# Association between oral microbiome and five types of respiratory infections: a two-sample Mendelian randomization study in east Asian population

**DOI:** 10.3389/fmicb.2024.1392473

**Published:** 2024-04-10

**Authors:** Jiawei He, Ningfeng Mao, Wenliang Lyu, Shuhan Zhou, Yang Zhang, Zhiyi Liu, Zixuan Xu

**Affiliations:** ^1^Institute of Epidemic Diseases, Hubei University of Chinese Medicine, Wuhan, Hubei, China; ^2^School of Traditional Chinese Medicine, Hubei University of Chinese Medicine, Wuhan, Hubei, China

**Keywords:** causal effect, Mendelian randomizations, oral microbiome, respiratory infection, respiratory tract diseases

## Abstract

**Objective:**

To explore the causal relationship between the oral microbiome and specific respiratory infections including tonsillitis, chronic sinusitis, bronchiectasis, bronchitis, and pneumonia, assessing the impact of genetic variations associated with the oral microbiome.

**Methods:**

Mendelian randomization was used to analyze genetic variations, leveraging data from genome-wide association studies in an East Asian cohort to identify connections between specific oral microbiota and respiratory infections.

**Results:**

Our analysis revealed that *Prevotella*, *Streptococcus*, *Fusobacterium*, *Pauljensenia*, and *Capnocytophaga* play crucial roles in influencing respiratory infections. *Prevotella* is associated with both promoting bronchitis and inhibiting pneumonia and tonsillitis, with a mixed effect on chronic sinusitis. *Streptococcus* and *Fusobacterium* show varied impacts on respiratory diseases, with *Fusobacterium* promoting chronic sinusitis, bronchiectasis, and bronchitis. Conversely, *Pauljensenia* and *Capnocytophaga* are linked to reduced bronchitis and tonsillitis, and inhibited pneumonia and bronchitis, respectively.

**Discussion:**

These findings underscore the significant impact of the oral microbiome on respiratory health, suggesting potential strategies for disease prevention and management through microbiome targeting. The study highlights the complexity of microbial influences on respiratory infections and the importance of further research to elucidate these relationships.

## 1 Introduction

The oral microbiome, possessing the second-highest level of diversity after the gut microbiome, encompasses a wide array of bacterial genera and families (Caselli et al., 2020). Anatomically, the oral cavity is directly linked to the airway via the oropharynx, which leads to the larynx and subsequently the trachea, facilitating not only breathing, speaking, and swallowing but also the potential transit of oral microorganisms into the respiratory tract. This anatomical pathway underscores the relevance of oral health in the context of respiratory infections. Its significant role in respiratory infections underscores the profound impact of oral health on respiratory diseases. Research demonstrates that oral bacteria can modulate the risk and severity of respiratory infections, highlighting the crucial role of oral hygiene in mitigating such conditions (Pathak et al., 2021; Dong et al., 2022). Furthermore, evidence supports a bidirectional association between the oral and lung microbiomes, suggesting implications for both the prevention and management of lung diseases (Pu et al., 2020). Compromised oral health has been linked to an elevated risk of respiratory infections, especially among high-risk groups, emphasizing the imperative of maintaining oral hygiene for respiratory well-being (Scannapieco, 1999; Mammen et al., 2020). Additionally, numerous clinical studies have validated the efficacy of oral probiotics, such as *Lactiplantibacillus plantarum* and *Lacticaseibacillus rhamnosus*, in decreasing the occurrence of ventilator-associated pneumonia, acute upper respiratory tract infections, and COVID-19 infections (Bo et al., 2014; Sundararaman et al., 2020; Zhao et al., 2022). Nonetheless, the complexity of conducting high-quality clinical trials has hindered the definitive establishment of this causal link in clinical settings. Moreover, identifying other specific in oral microbiome with potential causal roles in respiratory infections remains challenging with conventional methods alone.

Mendelian randomization (MR) represents a novel statistical approach for inferring causality, simulating the conditions of a randomized controlled trial through the random allocation of genetic variations during conception (Emdin et al., 2017). Utilizing single nucleotide polymorphisms (SNPs) as instrumental variables, MR models and infers causal effects, thereby eliminating confounding variables. Additionally, the irreversible nature of genetics helps to eliminate the possibility of reverse causation (Benn and Nordestgaard, 2018). This approach facilitates the evaluation of the microbiome’s influence on infections, significantly reducing the impact of confounding variables.

This study aims to investigate the causal relationship between the oral microbiome and various respiratory infections, conducting a comprehensive bidirectional MR analysis on conditions such as Tonsillitis, Chronic sinusitis, Bronchiectasis, Bronchitis, and Pneumonia.

## 2 Materials and methods

### 2.1 Data sources for the exposure

In our investigation, we utilized single nucleotide polymorphisms (SNPs) linked to the oral microbiota as instrumental variables (IVs) for a two-sample Mendelian Randomization (MR) analysis. This analysis leveraged summary statistics from a groundbreaking GWAS that explored the oral microbiome in an East Asian cohort. This particular GWAS, pioneering in its focus and scale for East Asian populations, analyzed data from 455 documented tongue dorsum microbiomes (*N* = 2,017) and 540 documented saliva microbiomes (*N* = 1,915).

For the microbiota of the tongue dorsum, our analysis revealed links to 1 phylum, 2 classes, 8 orders, 17 families, 15 genera, and 412 species. Regarding the saliva microbiota, we found associations with 2 phyla, 1 class, 4 orders, 13 families, 96 genera, and 424 species.

Further details on sample collection, sequencing methodologies, microbiome trait preparation, observational analyses, and genotyping methods are detailed in an accompanying publication (Li et al., 2022). This study marks a significant step in understanding the genetic determinants of the oral microbiota within East Asian populations, providing a comprehensive overview of its association with various microbial taxa down to the species level. The GWAS data is summarized in Table 1.

**Table 1:** Summary of the GWAS included in this Mendelian randomization study.

Exposures/outcomes	Consortium	Ethnicity	Sample sizes	Year
Oral microbiome	CNGBdb	East Asian	2,948 (Tongue *N* = 2017 Saliva *N* = 1914)	2021
Tonsillitis	NBDC Human Database	East Asian	152,266	2021
Chronic sinusitis	NBDC Human Database	East Asian	156,843	2021
Bronchiectasis	NBDC Human Database	East Asian	162,044	2021
Bronchitis	NBDC Human Database	East Asian	171,465	2021
Pneumonia	NBDC Human Database	East Asian	178,726	2021

GWAS, genome-wide association studies; SNPs, single nucleotide polymorphisms; IVs, instrumental variables; CNGBdb, China National GeneBank DataBase; NBDC, national bioscience database centre.

### 2.2 Data sources for the outcome

The GWAS data for this analysis, pertaining to five respiratory infections, was obtained from the publicly available National Bioscience Database Center (NBDC) Human Database.^1^ The data originated from a detailed GWAS of an East Asian cohort. This included 420 cases and 151,846 controls for Tonsillitis, 4,617 cases and 152,226 controls for Chronic Sinusitis, 241 cases and 161,803 controls for Bronchiectasis, 1,465 cases and 170,000 controls for Bronchitis, and 7,423 cases and 171,303 controls for Pneumonia. The GWAS data is summarized in Table 1.

### 2.3 Selection of instrumental variables

In this Mendelian randomization (MR) study, we utilized single nucleotide polymorphisms (SNPs) strongly linked with each oral microbiota taxon as instrumental variables (IVs). Due to the limited number of IVs identified using a stringent *p*-value threshold (*p* < 5 × 10–8), we adopted a more inclusive criterion (*p* < 1 × 10–5) to amass a larger set of IVs, thereby strengthening the validity of our findings. To ensure the independence of each IV and reduce the effects of linkage disequilibrium (LD), SNPs within a 10,000 kb radius were pruned using an r2 threshold of <0.001. The LD r2 values were estimated using the 1,000 Genomes Project data for East Asian populations (Auton et al., 2015).

To negate the influence of weak instrument bias on the estimation of association effects, we assessed the strength of the instruments using an F-statistic threshold greater than 10. The F-statistic was calculated using the formula: F = (R2 / (1 − R2)) * ((n − k − 1) / k), where R2 represents the proportion of variance explained by the instruments for each oral microbiota component, n is the sample size, and k is the number of instruments. The R2 was determined using the minor allele frequency (MAF) and effect estimates (β) as follows: R2 = 2 × MAF × (1 − MAF) × (β / SD)2. An F-statistic greater than 10 suggests that the results are not biased by weak IVs (Burgess et al., 2018).

### 2.4 MR analysis

**Figure 1:**
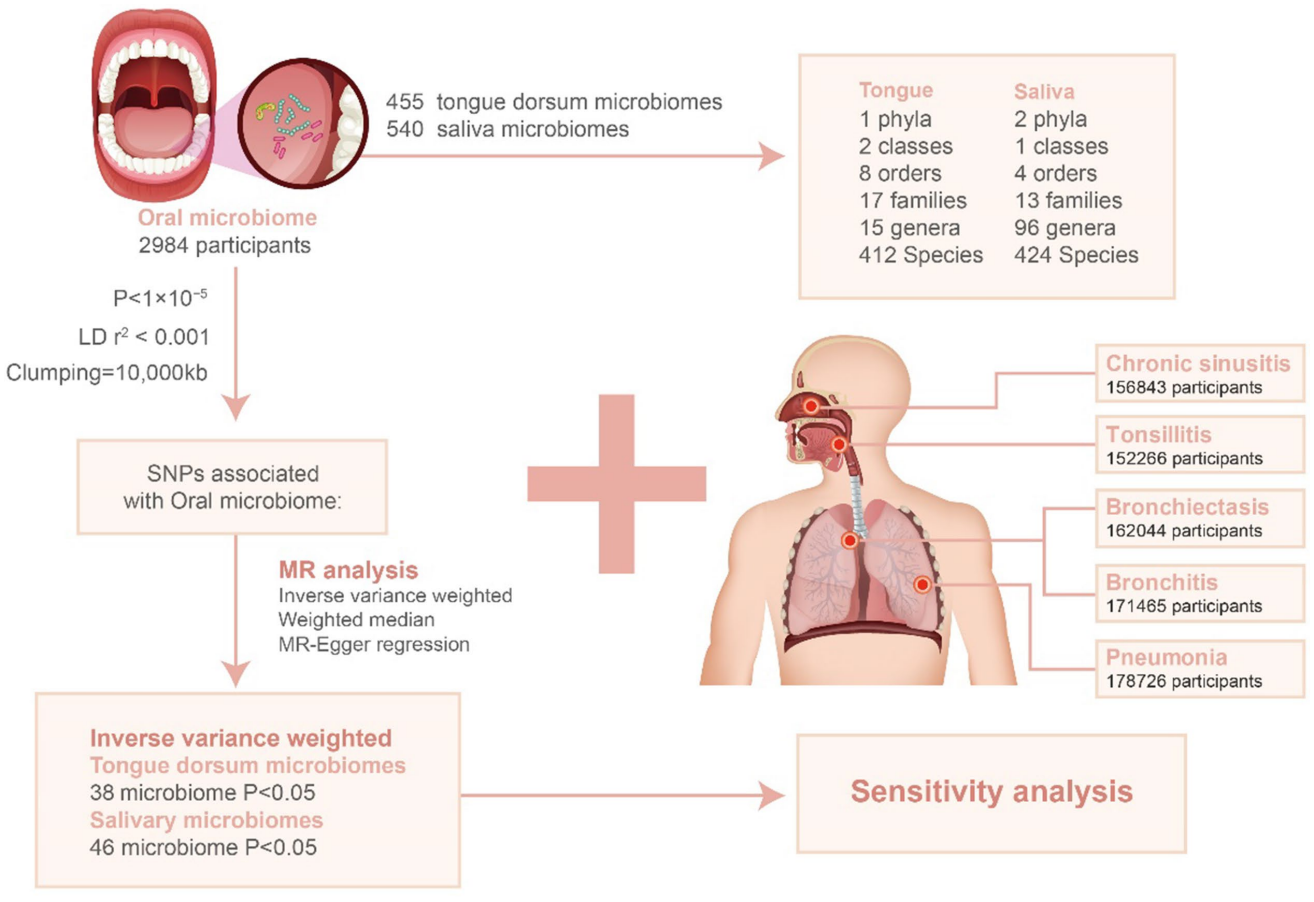
Flowchart for the study of the association between oral microbiota and respiratory infections. LD: linkage disequilibrium; SNP: single nucleotide polymorphism.

Mendelian randomization analysis was conducted to explore the potential causal link between oral microbiota exposure and respiratory infections. This analysis primarily utilized the inverse variance weighted (IVW) method (Burgess et al., 2018), supplemented by weighted median (WM) and MR-Egger regression as auxiliary tools (Bowden et al., 2015, 2016). Statistical significance was determined when *p*-values were below 0.05. The flowchart depicted in Figure 1 provides an overview of the MR analysis procedure.

### 2.5 Sensitivity analysis

Additionally, we conducted a suite of sensitivity analyses to ensure the accuracy and robustness of our findings. On one hand, the MR-PRESSO global test and the MR Egger intercept test were utilized to assess the pleiotropy at the aggregate level of the instrumental variables (IVs). Both methods yielded *p*-values greater than 0.05, indicating no evidence of pleiotropy (Verbanck et al., 2018). On the other hand, heterogeneity was evaluated using Cochran’s Q test, with a p-value greater than 0.05 suggesting the absence of heterogeneity (Hemani et al., 2018). Finally, a leave-one-out sensitivity analysis was employed to ascertain if any single SNP could influence the inference of the causal relationship.

All MR analyses were conducted using R (version 4.3.1) with the TwoSampleMR and MR-PRESSO packages.

## 3 Results

### 3.1 Instrumental variables selection

We performed clustering on 455 tongue dorsum microbiomes and 540 saliva microbiomes, identifying 17,544 and 22,553 independent SNPs (*p* < 1 × 10–5) associated with them, respectively, across six taxonomic levels: phylum, class, order, family, genus, and species. After adjusting for linkage disequilibrium (LD) by removing SNPs in LD, all retained SNPs exhibited an F-statistic greater than 10, indicating a low likelihood of weak instrumental bias affecting the estimates (Supplementary Tables S1, S2). Following the harmonization of exposure and outcome alleles and conducting Mendelian randomization (MR) analyses, significant associations were detected using the inverse variance weighted (IVW) method with a *p*-value <0.05. Consistent trends were observed with the weighted median (WM) and MR-Egger methods (Supplementary Tables S3, S4). This analysis resulted in 367 SNPs from 38 tongue dorsum microbiomes and 405 SNPs from 46 saliva microbiomes.

### 3.2 Causal effects of oral microbiota on five types of respiratory infections

#### 3.2.1 Tonsillitis

**Figure 2:**
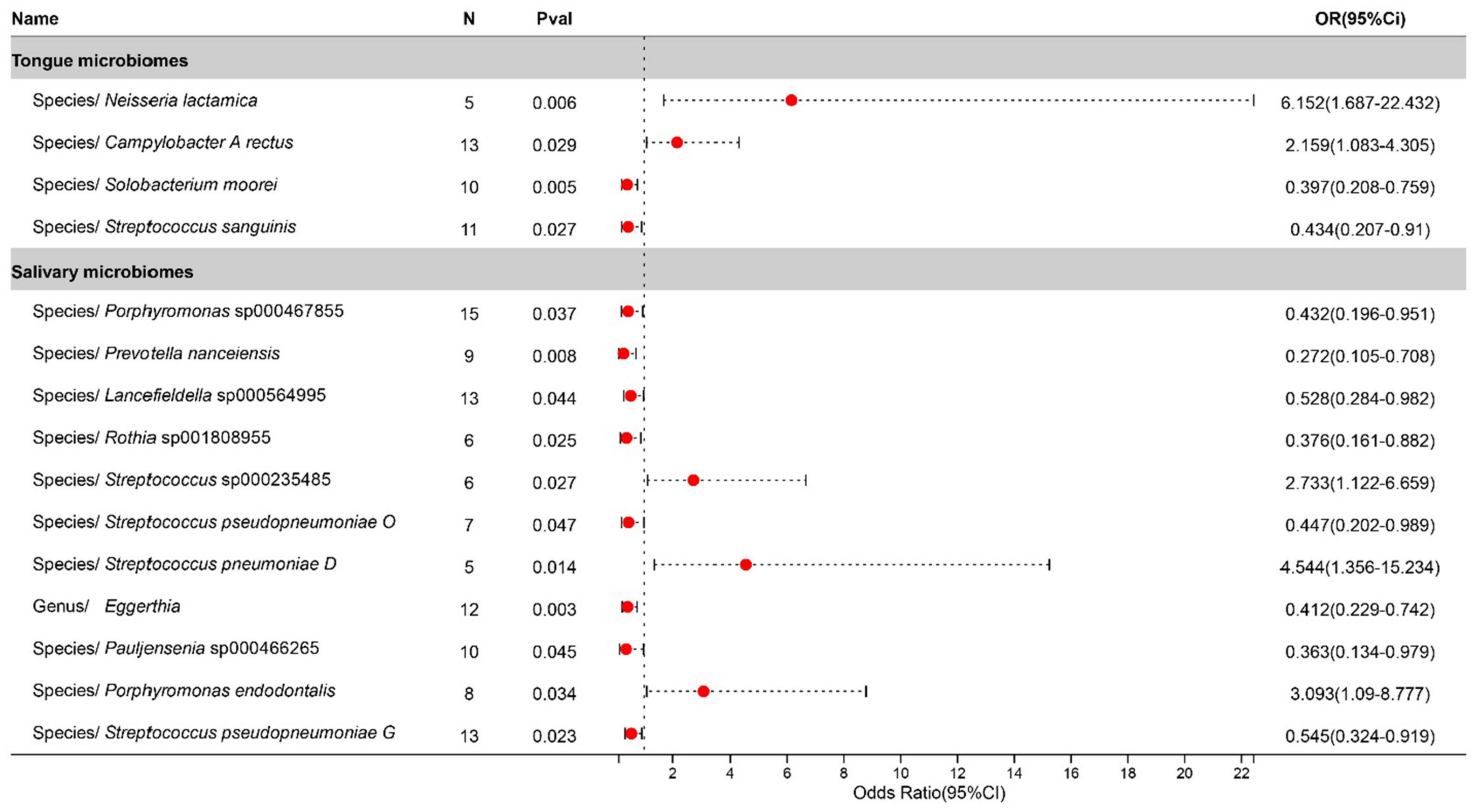
Forest plot of tongue dorsum microbiomes and salivary microbiomes taxa associated with Tonsillitis identified by IVW method.

Statistically significant associations with Tonsillitis were identified for 4 bacterial species in the tongue (across 4 genera and 4 families) and 11 in saliva (spanning 7 genera and 7 families), with the *Streptococcus* genus present in both. The causal impacts of these bacterial species on Tonsillitis are illustrated in Figure 2.

#### 3.2.2 Chronic sinusitis

**Figure 3:**
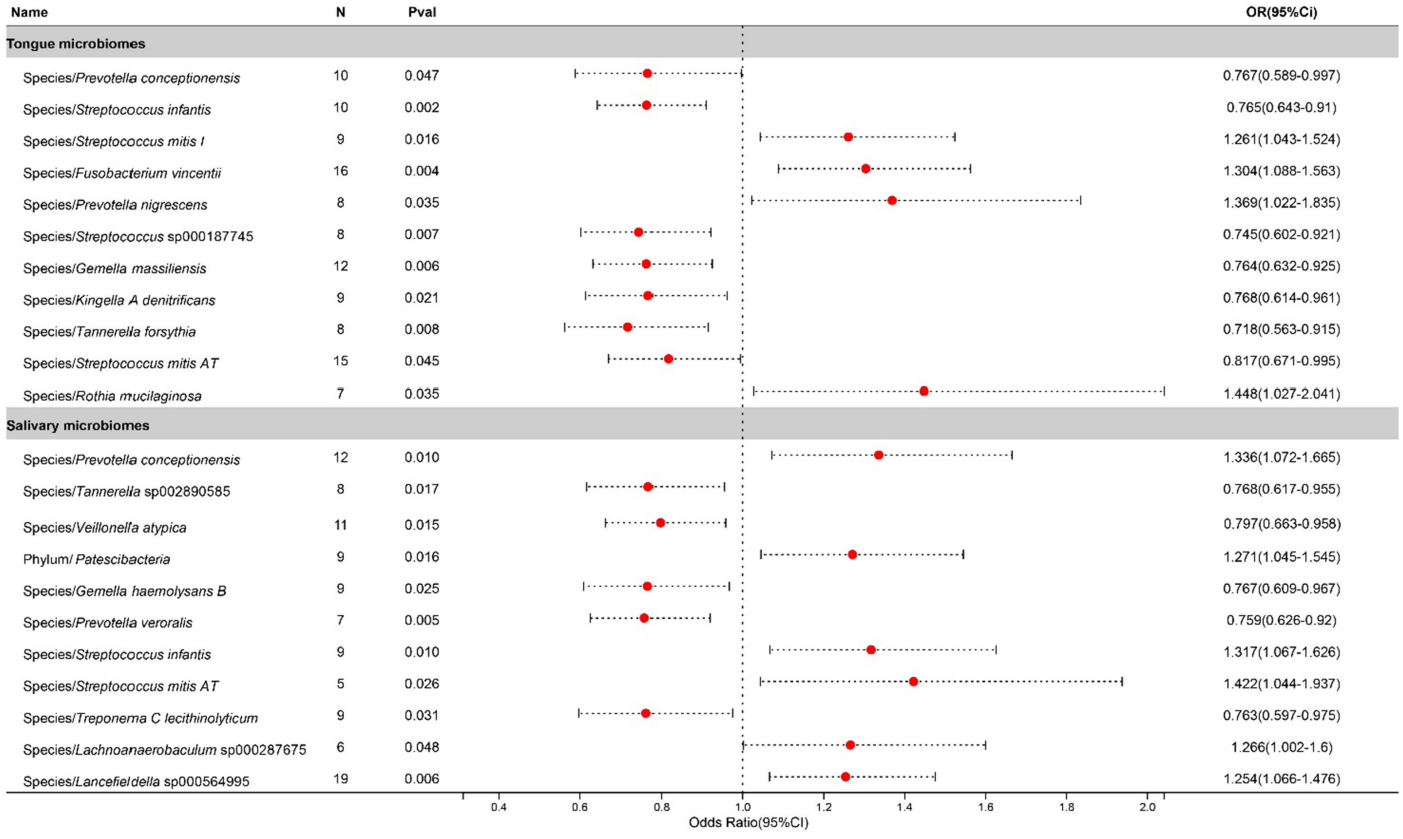
Forest plot of tongue dorsum microbiomes and salivary microbiomes taxa associated with Chronic sinusitis identified by IVW method.

In the study on chronic sinusitis, after excluding two unclassified bacterial species, statistically significant correlations were identified for 11 bacterial species located on the tongue (spanning 8 genera and 7 families) and 11 in saliva (8 genera and 7 families), with *Prevotella*, *Gemella*, *Tannerella*, and *Streptococcus* shared between both. To be specific, salivary *Streptococcus infantis* appears to exacerbate the condition, whereas its counterpart on the tongue exhibits a suppressive effect. Contrastingly, *Streptococcus mitis AT* found in saliva contributes to the progression of the disease, while the same species present on the tongue plays an inhibitory role. Causal effects on Chronic sinusitis by these bacteria are illustrated in Figure 3.

#### 3.2.3 Bronchiectasis

**Figure 4:**
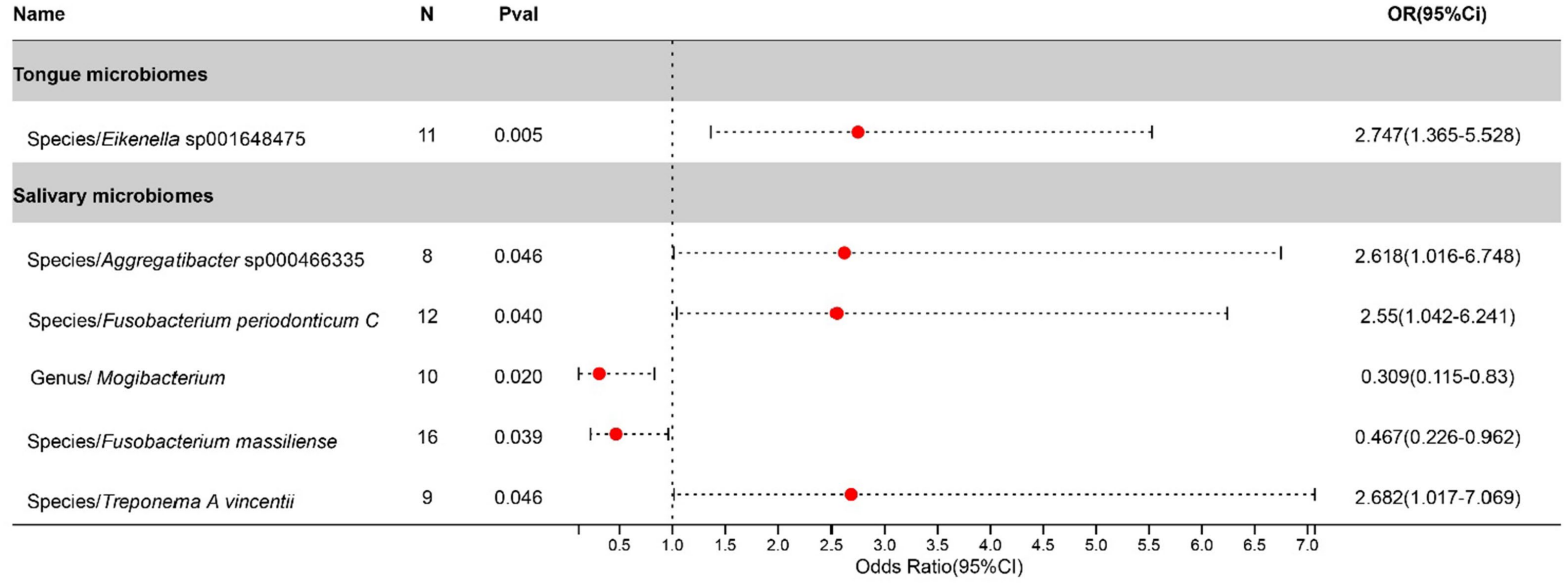
Forest plot of tongue dorsum microbiomes and salivary microbiomes taxa associated with Bronchiectasis identified by IVW method.

After excluding three unclassified bacterial species, statistically significant associations with Bronchiectasis were identified for 1 bacterial species in the tongue (1 genera and 1 families) and 5 in saliva (4 genera and 4 families). The causal impacts of these bacterial species on Bronchiectasis are detailed in Figure 4.

#### 3.2.4 Bronchitis

**Figure 5:**
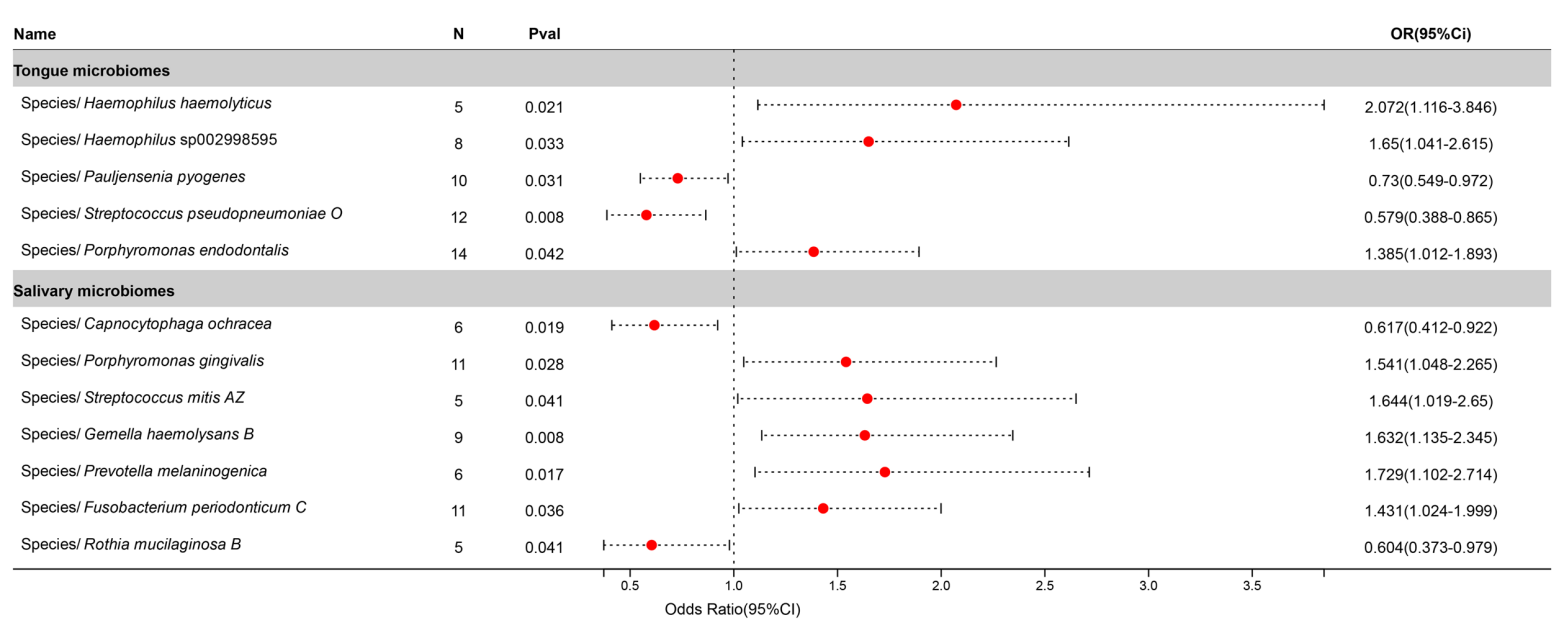
Forest plot of tongue dorsum microbiomes and salivary microbiomes taxa associated with Bronchitis identified by IVW method.

After excluding three unclassified bacterial species, statistically significant relationships with Bronchitis were found for 5 bacterial species in the tongue (5 genera and 5 families) and 7 in saliva (7 genera and 5 families), with *Porphyromonas* and *Streptococcus* being common to both. The causal impacts of these bacterial species on Bronchitis are illustrated in Figure 5.

#### 3.2.5 Pneumonia

**Figure 6:**
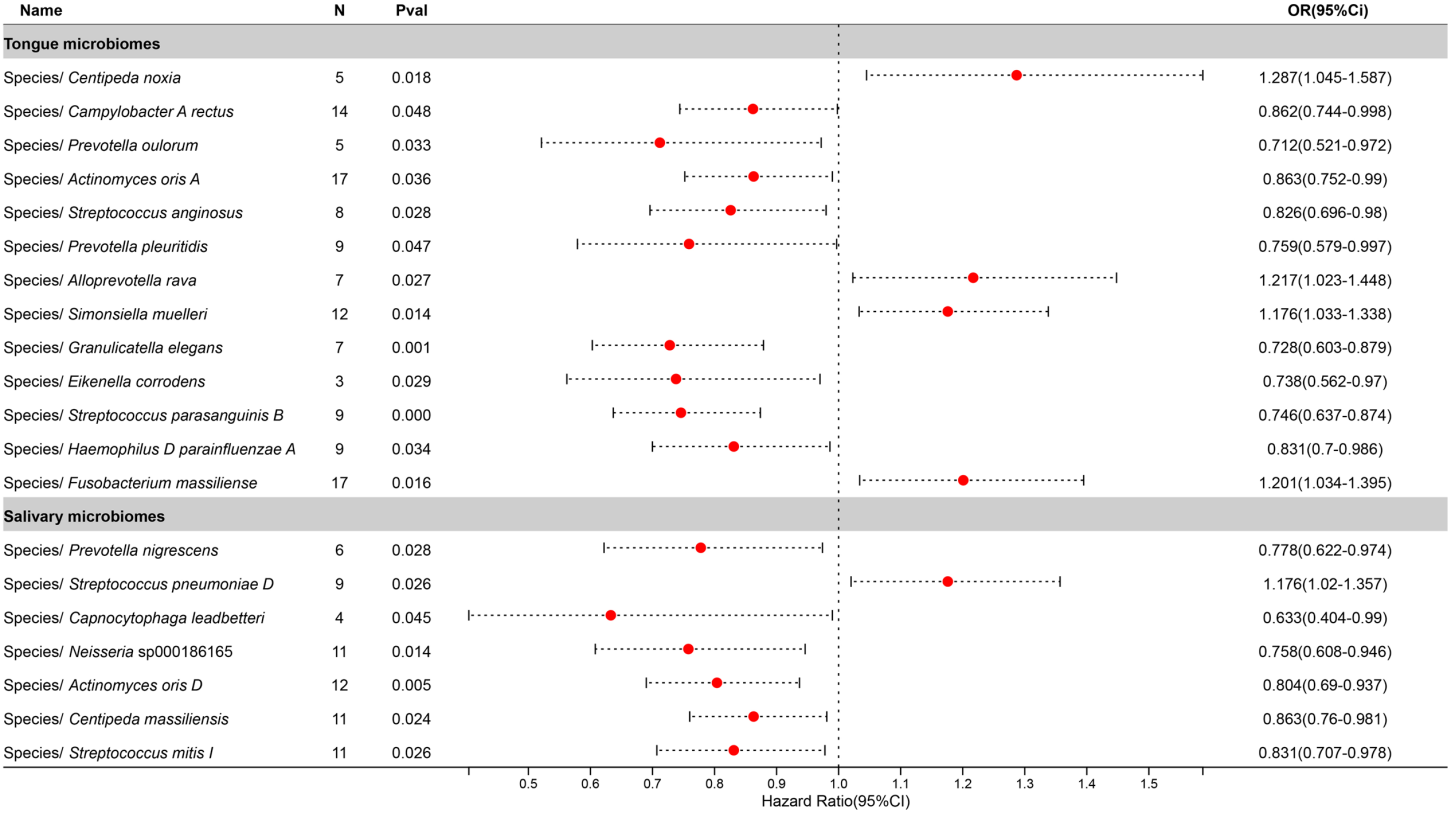
Forest plot of tongue dorsum microbiomes and salivary microbiomes taxa associated with Pneumonia identified by IVW method.

After excluding three unclassified bacterial species, statistically significant associations with Pneumonia were identified for 13 bacterial species in the tongue (11 genera and 9 families) and 7 in saliva (6 genera and 6 families), with *Prevotella*, *Actinomyces*, *Streptococcus*, and *Centipeda* shared between both. The causal impacts of these bacterial species on Pneumonia are depicted in Figure 6.

### 3.3 Sensitivity analysis

Heterogeneity was assessed using Cochran’s Q statistic for both IVW and MR-Egger methods, with their corresponding Q_pval greater than 0.05, indicating the absence of heterogeneity, thereby justifying the use of fixed-effect models for IVW (Supplementary Tables S5, S6). Outlier detection through the MR-PRESSO method revealed no outliers, as evidenced by a *p*-value greater than 0.05 (Supplementary Tables S7, S8). Furthermore, the stability of MR results was assessed through a leave-one-out analysis, which showed that no single SNP exerted a significant influence on the stability of the study outcomes (Supplementary Tables S9, S10). Therefore, it is concluded that the MR analysis results between the oral microbiome and various respiratory infection phenotypes are stable. Additionally, detailed forest plots, funnel plots, scatter plots, and leave-one-out plots can be found in the Supplementary materials.

### 3.4 Comprehensive relationship between specific oral microbiota genera and respiratory infections

**Figure 7:**
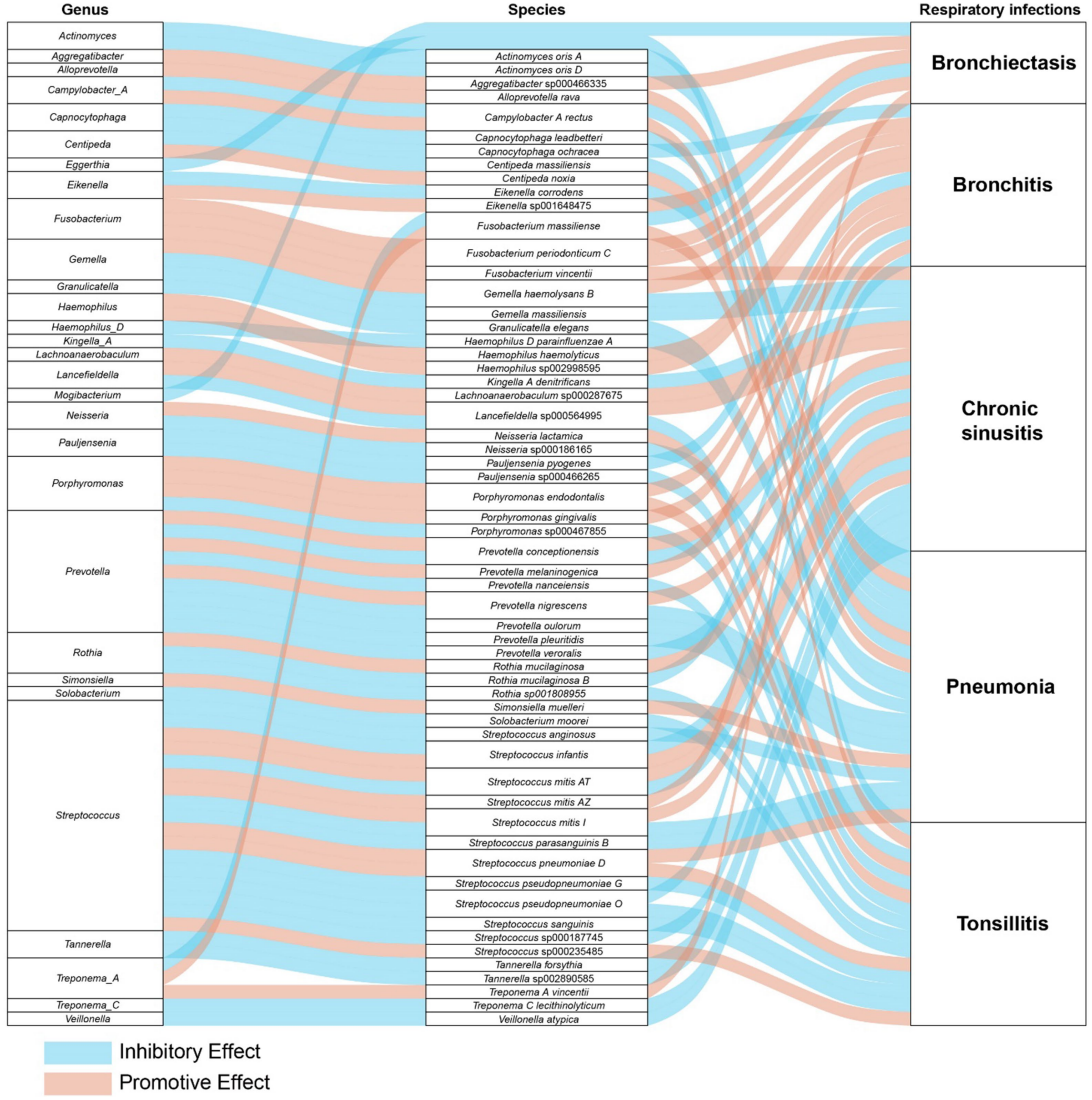
The comprehensive relationships between oral microflora at the genus-species level and five types of respiratory infections.

In a detailed examination of the oral microbiome, certain bacterial genera have emerged as key players influencing a range of respiratory infections, based on extensive analysis of data from saliva and tongue samples. *Streptococcus* exhibits a varied influence on chronic sinusitis, pneumonia, tonsillitis, and bronchitis. Specifically, *Streptococcus pneumoniae* was found to contribute to the aggravation of pneumonia and tonsillitis. Conversely, *Streptococcus pseudopneumoniae* demonstrated an inhibitory effect on the development of tonsillitis and bronchitis. Furthermore, our research indicates that *Streptococcus infantis* and *Streptococcus mitis* exhibit bidirectional effects on chronic sinusitis. Additionally, *Streptococcus mitis* I was observed to exacerbate chronic sinusitis; however, it exerts an inhibitory effect on the progression of pneumonia. The genus *Prevotella* is noted for its complex role: it appears to promote bronchitis, while it inhibits pneumonia and tonsillitis, and shows a mixed effect on chronic sinusitis. *Fusobacterium* is recognized for promoting chronic sinusitis, bronchiectasis, and bronchitis. Additionally, *Pauljensenia* is linked to a reduction in bronchitis and tonsillitis cases. *Capnocytophaga* is found to play a role in inhibiting pneumonia and bronchitis. Table 2 offers a concise overview of the associations between different respiratory infections and oral microbiota genera. Figure 7 details the hierarchy from oral microflora genera to species and their links to respiratory infections.

**Table 2:** Summary of multiple respiratory infections in relation to bacteria (genera) present in both tongue and saliva.

Outcomes	Mechanism	Quantity	Bacterial (genus)
Chronic sinusitis	Inhibit	5	*Gemella,Kingella_A, Tannerella,Veillonella, Treponema_C*
	Promote	4	*Fusobacterium, Rothia, Lachnoanaerobaculum, Lancefieldella*
	Mix	3	*Streptococcus,Prevotella*
Pneumonia	Inhibit	9	*Campylobacter_A, Prevotella, Actinomyces,Granulicatella, Eikenella, Haemophilus_D, Capnocytophaga, Neisseria*
	Promote	5	*Alloprevotella,Simonsiella, Treponema_A*
	Mix	2	*Centipeda, Streptococcus*
Tonsillitis	Inhibit	6	*Solobacterium, Prevotella, Pauljensenia, Eggerthia, Rothia, Lancefieldella*
	Promote	2	*Neisseria, Campylobacter_A*
	Mix	2	*Porphyromonas, Streptococcus*
Bronchiectasis	Inhibit	2	*Mogibacterium*
	Promote	3	*Eikenella, Fusobacterium, Aggregatibacter*
	Mix	1	*Treponema_A*
Bronchitis	Inhibit	5	*Pauljensenia, Capnocytophaga, Rothia*
	Promote	6	*Haemophilus, Porphyromonas, Gemella, Prevotella, Fusobacterium*
	Mix	1	*Streptococcus*

## 4 Discussion

To our knowledge, this represents the first endeavor to utilize publicly available genetic databases to delve into the causal relationship between the oral microbiome and respiratory infections. In our study, we conducted a comprehensive Mendelian randomization (MR) analysis of GWAS data involving 995 taxa, aiming to investigate the potential role of the oral microbiome in the development of respiratory infections. Drawing on extensive genetic data from 2,948 East Asian participants, we identified 80 types of oral microbiota that may play a significant role in the development of respiratory infections.

Recent studies have increasingly focused on the connection between the oral microbiome and respiratory infections, highlighting the complex ecosystem of the oral microenvironment that includes the microbiota, anatomical structures, saliva, and their interactions with the host. A pivotal insight derived from existing studies highlights the significance of oral microbes—located in dental plaques, periodontal pockets, or within saliva—as foundational sources for respiratory infections. These microbes can be inhaled into the lower respiratory tract, potentially causing or exacerbating diseases such as aspiration pneumonia and COPD. The pathogenicity of these microorganisms involves various mechanisms, notably immunoregulation involving Th1 and Th2 immune responses. Studies have shown that oral bacteria can trigger the release of proinflammatory cytokines in a manner similar to respiratory pathogens, significantly affecting the immune response (Scannapieco et al., 2001; Petelin et al., 2004; Schaub et al., 2006). Additionally, certain periodontopathic bacteria like *Fusobacterium nucleatum* have been identified to induce IL-6 and IL-8 production, exacerbating conditions such as COPD (Hayata et al., 2019). Moreover, interactions between oral microbes and respiratory pathogens can enhance the virulence of the latter, as demonstrated in studies involving oral *Streptococci* and *P. aeruginosa*, further emphasizing the intricate relationship between oral health and respiratory diseases (Duan et al., 2003; Parkins et al., 2008; Sibley et al., 2008; Whiley et al., 2014, 2015).

Tonsillitis research increasingly points to the oral microbiome’s critical role, where dysbiosis significantly contributes to the pathogenesis of tonsillar diseases (Nagasawa et al., 2021; Park et al., 2023). *Neisseria lactamica*, traditionally viewed as a commensal organism, is now linked to an elevated risk of tonsillitis due to its potential to modulate immune responses in the mucosa, suggesting its role in increasing susceptibility to this condition (Vaughan et al., 2010). Similarly, *Campylobacter rectus*, often associated with periodontal disease, has been found in the tonsillar crypts of both affected and unaffected individuals, with this study confirming its consistent association with a heightened risk of tonsillitis (Nomura et al., 2023). *Porphyromonas endodontalis*, known for its presence in periodontal infections, has been detected in those with recurrent tonsillitis, underscoring the interconnectedness between oral health and tonsillar pathology, with findings here aligning with its role in exacerbating the condition (Jensen et al., 2013). Conversely, *Streptococcus sanguinis*, a normal component of the oral microbiota, has been identified as a protective factor against recurrent streptococcal tonsillitis, likely through competitive inhibition or immune modulation, a finding that matches our study’s observations on its beneficial role in the tonsillar environment (Murakata and Harabuchi, 1999).

Chronic sinusitis, a widespread inflammatory condition, is increasingly linked to the oral microbiome. Research by Siu et al. reveals that antibiotic treatment-induced modifications in the oral and gut microbiomes, due to drug distribution, may influence chronic sinusitis treatment outcomes (Siu et al., 2019, 2021). Furthermore, Mehra underscores the significant role of odontogenic factors in chronic sinusitis, suggesting that dental conditions like periodontitis may impact sinus health via alterations in the oral microbiome’s composition (Mehra and Jeong, 2009). The detection of *Streptococcus mitis* in chronic sinusitis patients’ sinuses corroborates its contributory role in the condition’s inflammation and pathogenesis as an oral microbiota component (Paju et al., 2003; Cariati and Deng, 2014). Likewise, the enrichment of *Rothia mucilaginosa* in these patients’ sinus microbiota indicates its potential involvement in the disease’s pathology, aligning with our research findings (Yuan et al., 2020). In contrast, while *Tannerella forsythia* is known as a periodontal pathogen associated with a higher incidence of chronic sinusitis, implying a connection between oral health and upper respiratory tract diseases (Uraz et al., 2019), our research suggests its association with a decreased risk of chronic sinusitis, prompting further investigation to clarify this relationship.

Bronchiectasis has been increasingly recognized for its complex interplay with the oral microbiome, shedding light on the potential mechanisms influencing pulmonary disease progression and management. The significance of oral-lung microbiome interactions in lung pathologies, including bronchiectasis and cystic fibrosis, underscores the potential impact on disease progression and management strategies (Mammen et al., 2020). Furthermore, the pivotal role of the oral microecosystem as a primary source for lung microbiota, influencing respiratory health outcomes, has been emphasized (Dong et al., 2022). These insights suggest that targeting the oral microbiome could offer novel therapeutic interventions and preventive measures for managing bronchiectasis, highlighting a significant area for future research and clinical application.

Bronchitis, a respiratory condition marked by the inflammation of the bronchial tubes, has been increasingly studied in the context of the oral microbiome. Research indicates that oral purified bacterial extracts may alleviate symptoms in patients with chronic bronchitis and COPD, although evidence on their preventive capabilities remains inconclusive (Steurer-Stey et al., 2004).

Pneumonia, often linked to dental plaque bacteria like *Streptococcus pneumoniae*, significantly impacts community-acquired pneumonia (CAP) severity and hospitalization rates (Sumi et al., 2007; Yamasaki et al., 2013). Oral care is crucial in reducing pneumonia risk, especially in hospital settings (Quagliarello et al., 2005; Heo et al., 2008). The COVID-19 pandemic has further highlighted saliva’s role in viral pneumonia transmission and diagnosis (Chen et al., 2020; To et al., 2020). *Streptococcus pneumoniae*, a Gram-positive bacterium, is widely recognized as a major pathogen causing community-acquired pneumonia (CAP). *Pneumococcus* can cause various infections, including otitis media, sinusitis, meningitis, and the most severe form, pneumonia. Pneumococcal pneumonia poses a significant challenge to global public health, especially in children and the elderly. Studies have shown that community-acquired pneumonia is the primary manifestation of severe pneumococcal disease in adults (Grant et al., 2024), consistent with the findings of our study.

In essence, a singular bacterium can play a dichotomous role in the dynamics of respiratory infections, oscillating between inhibiting and promoting the progression of such infections via a myriad of mechanisms. The correlation between the oral microbiome and respiratory infections underscores substantial implications for both public health and clinical practices. There exists a significant potential in mitigating the risk of respiratory infections through diligent oral hygiene and the strategic regulation of the oral microbiome’s equilibrium. Thus, forthcoming research endeavors should focus on delineating the specific influence of the oral microbiome on respiratory well-being and harnessing this insight to forge novel prophylactic and therapeutic interventions aimed at countering respiratory infections and associated ailments.

Our study showcases several significant strengths. First and foremost, it incorporates the latest GWAS datasets related to the oral microbiome and employs Mendelian Randomization (MR) to delineate causal relationships. The intricate network of associations between oral microbiota and the development of respiratory infections underscores the necessity for an in-depth comprehension of this complex system. As we delve further into this domain, our findings offer novel insights for subsequent research endeavors and potential therapeutic strategies. Secondly, this research represents the inaugural Mendelian Randomization study probing the association between oral microbiota and multiple respiratory infections within an East Asian demographic. Additionally, our research’s ability to precisely identify microbial species elevates the depth and specificity of our understanding of the oral microbiome’s impact on respiratory health. By advancing beyond generic genus-level analyses to detailed species-level identification, we illuminate the nuanced pathways through which specific oral bacteria influence respiratory infections. This meticulous approach allows us to map the intricate connections from the genus and species of oral microbiota to their roles in disease processes, revealing the unique effects individual bacterial species have on respiratory health. Moreover, our study sheds light on the bidirectional influences of multiple bacterial genera on respiratory diseases. This detailed exploration underscores the complexity of microbial interactions within the oral cavity and their profound implications for respiratory disease mechanisms and potential therapeutic interventions.

Nonetheless, it is critical to acknowledge several limitations within our study. The possibility of horizontal pleiotropy might affect the selection of instrumental variables in MR analyses. The oral microbiome’s composition can be influenced by numerous factors, such as genetic predispositions, lifestyle habits, dietary modifications, and environmental influences. Instrumental variables might capture only a fraction of the observed variability, necessitating further studies to unravel the complex dynamics of oral microbiota changes fully. Moreover, given that our MR analysis was primarily concentrated on Asian ancestries, the applicability of our findings to populations of European descent may be limited.

## 5 Conclusion

Utilizing sophisticated GWAS datasets and Mendelian randomization techniques, our study enhances the comprehension of the oral microbiome’s crucial influence on respiratory infections, uncovering likely causal links between particular oral microorganisms and five distinct respiratory ailments. Our results uncover the intricate functions of the oral microbiome, with these organisms playing varied roles that can either dampen or intensify the progression of infections. This underscores the significance of the oral microbiome as a key target for groundbreaking preventive and therapeutic approaches in the management of respiratory health.

## Data availability statement

The original contributions presented in the study are included in the article/Supplementary material, further inquiries can be directed to the corresponding authors.

## Author contributions

JH: Writing – original draft, Visualization, Methodology, Formal analysis, Data curation, Conceptualization. NM: Writing – original draft, Formal analysis, Data curation. WL: Writing – review & editing, Supervision. SZ: Writing – review & editing, Supervision. YZ: Writing – review & editing. ZL: Writing – review & editing. ZX: Writing – original draft.

## Footnotes

^1^ https://humandbs.biosciencedbc.jp/en/
