# Supplementary figures and images for: Association between oral microbiome and five types of respiratory infections: a two-sample Mendelian randomization study in east Asian population (part 2 of 2)
Source: Front Microbiol. 2024 Apr 10;15:1392473. doi: 10.3389/fmicb.2024.1392473 (PMC11039966; doi:10.3389/fmicb.2024.1392473)

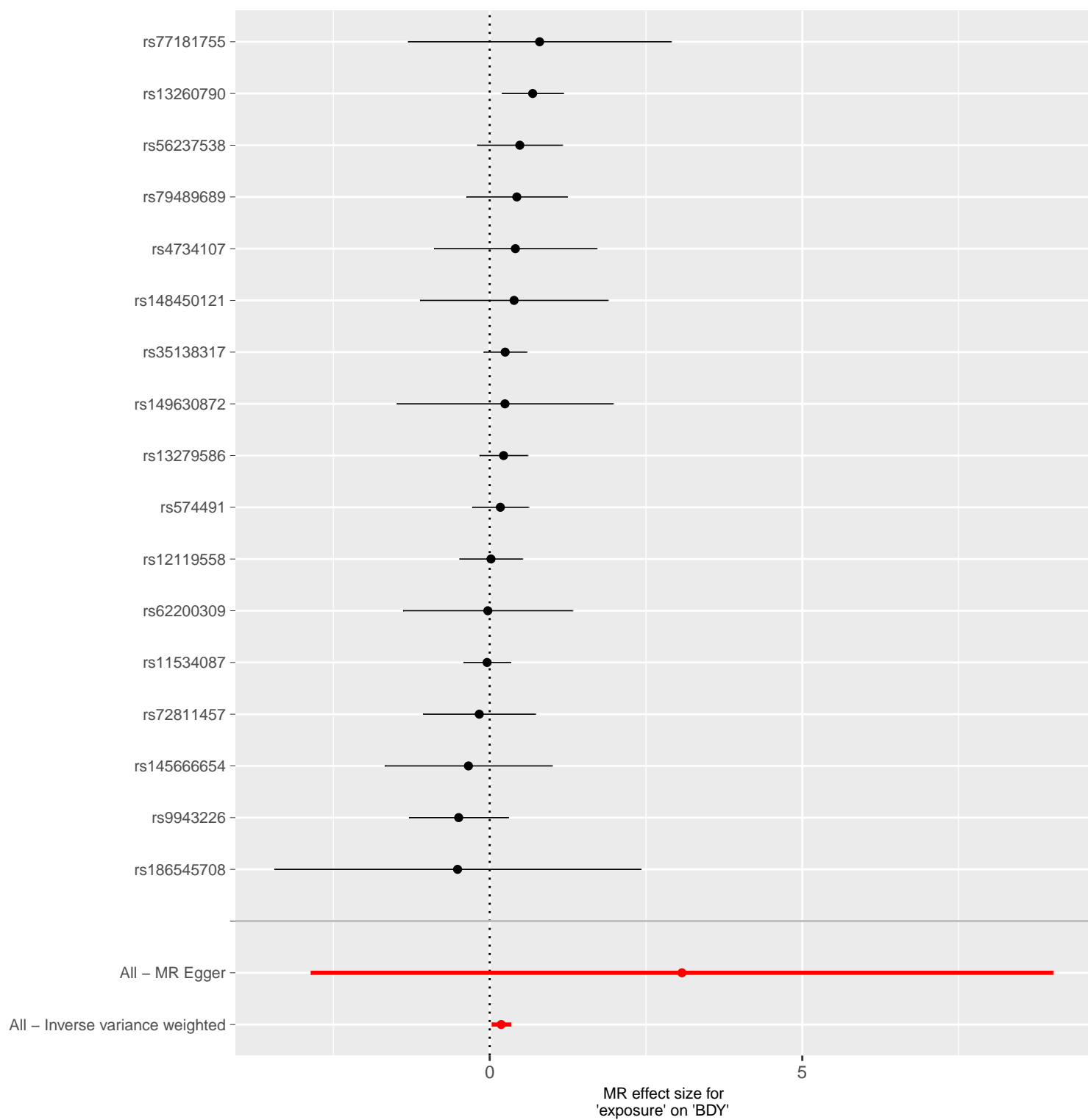

Supplement: Supplementary file 1 [file Data_Sheet_1.zip › Supplementary Materials/MR plots for tongue/Pneumonia/s__Fusobacterium_massiliense_mgs_586/forest.pdf]

# MR Method

- Inverse variance weighted
- MR Egger

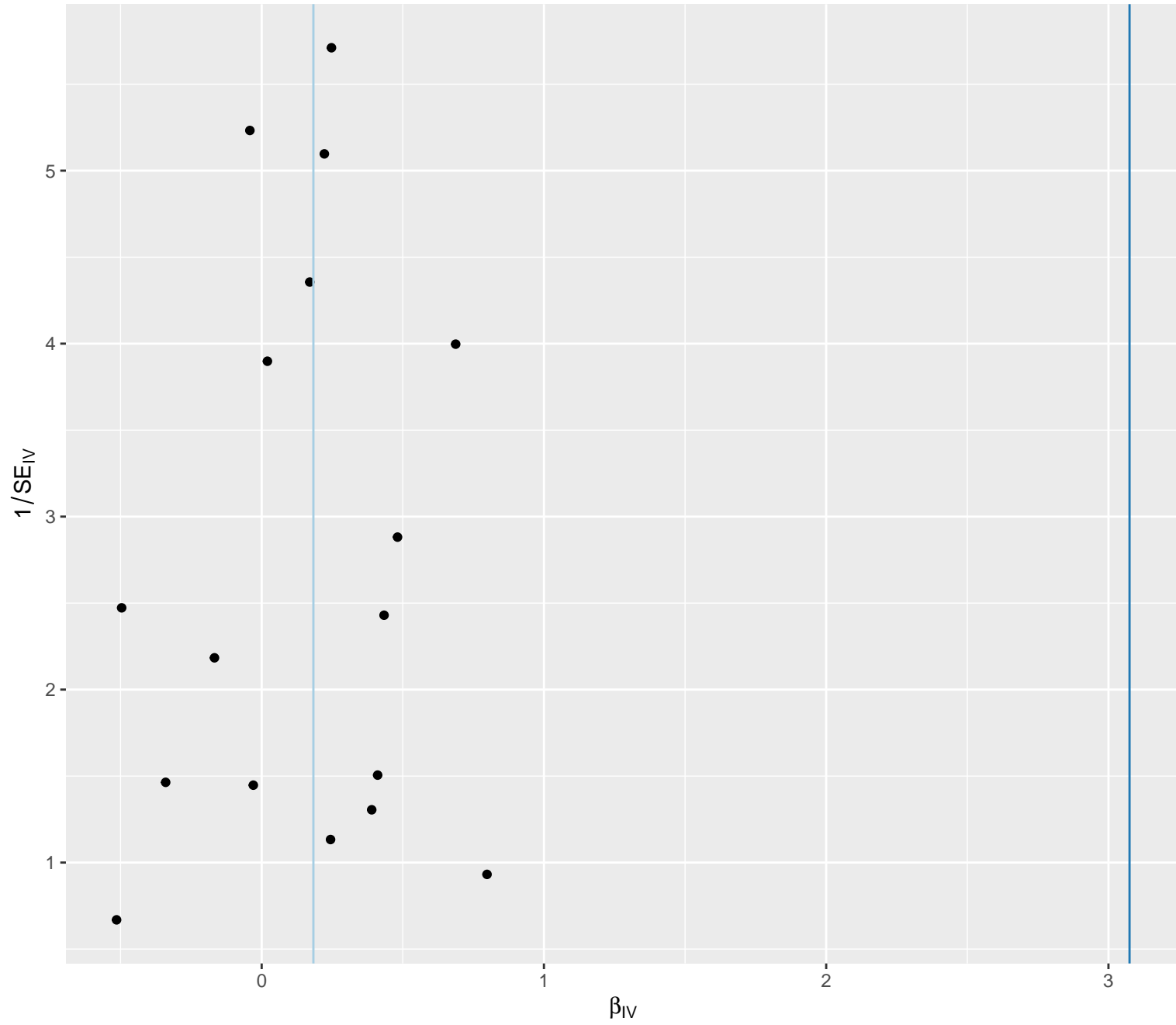

Supplement: Supplementary file 1 [file Data_Sheet_1.zip › Supplementary Materials/MR plots for tongue/Pneumonia/s__Fusobacterium_massiliense_mgs_586/funnel.pdf]

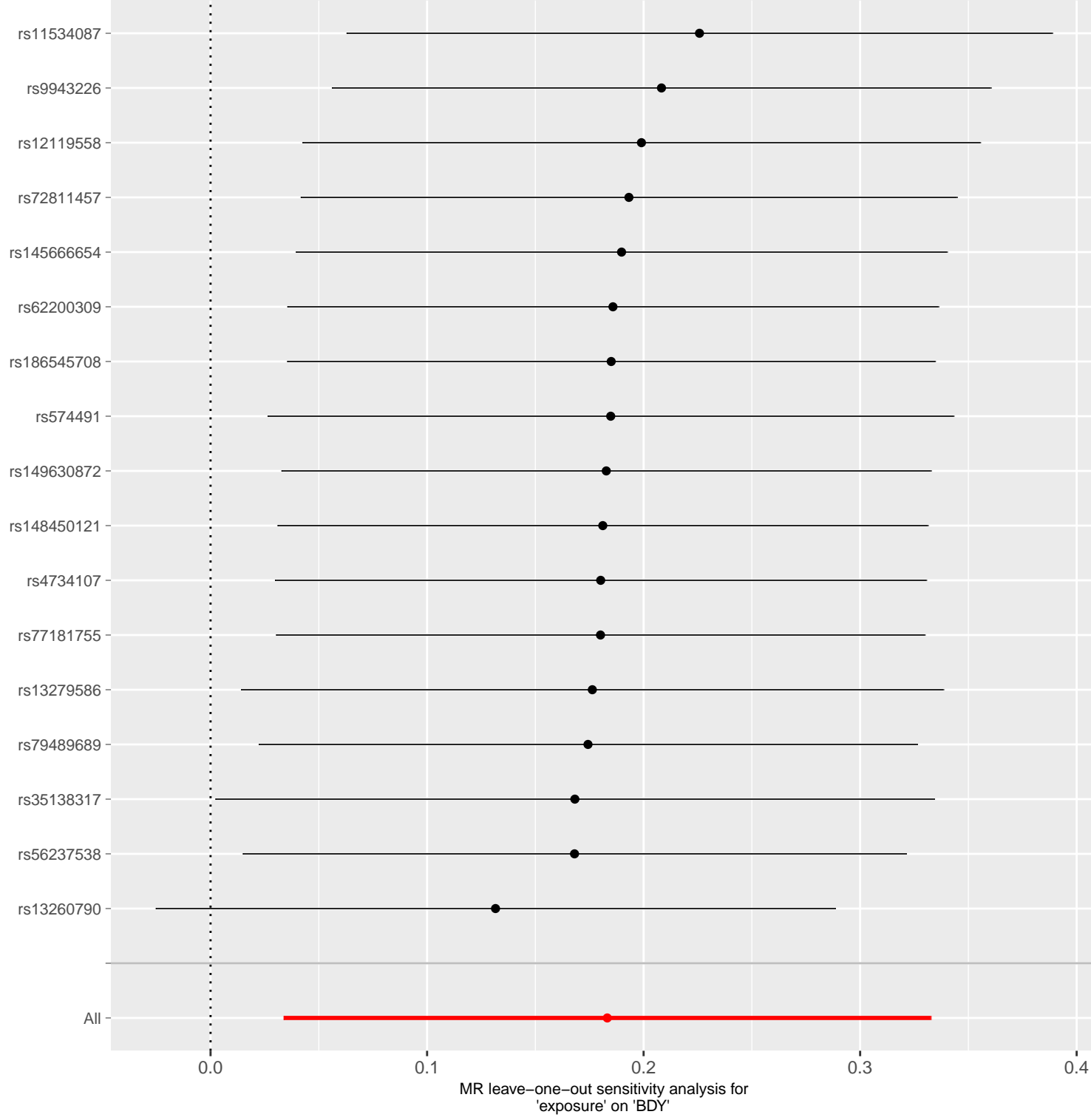

Supplement: Supplementary file 1 [file Data_Sheet_1.zip › Supplementary Materials/MR plots for tongue/Pneumonia/s__Fusobacterium_massiliense_mgs_586/leave_one_out.pdf]

# MR Test

- Inverse variance weighted
- MR Egger
- Weighted median

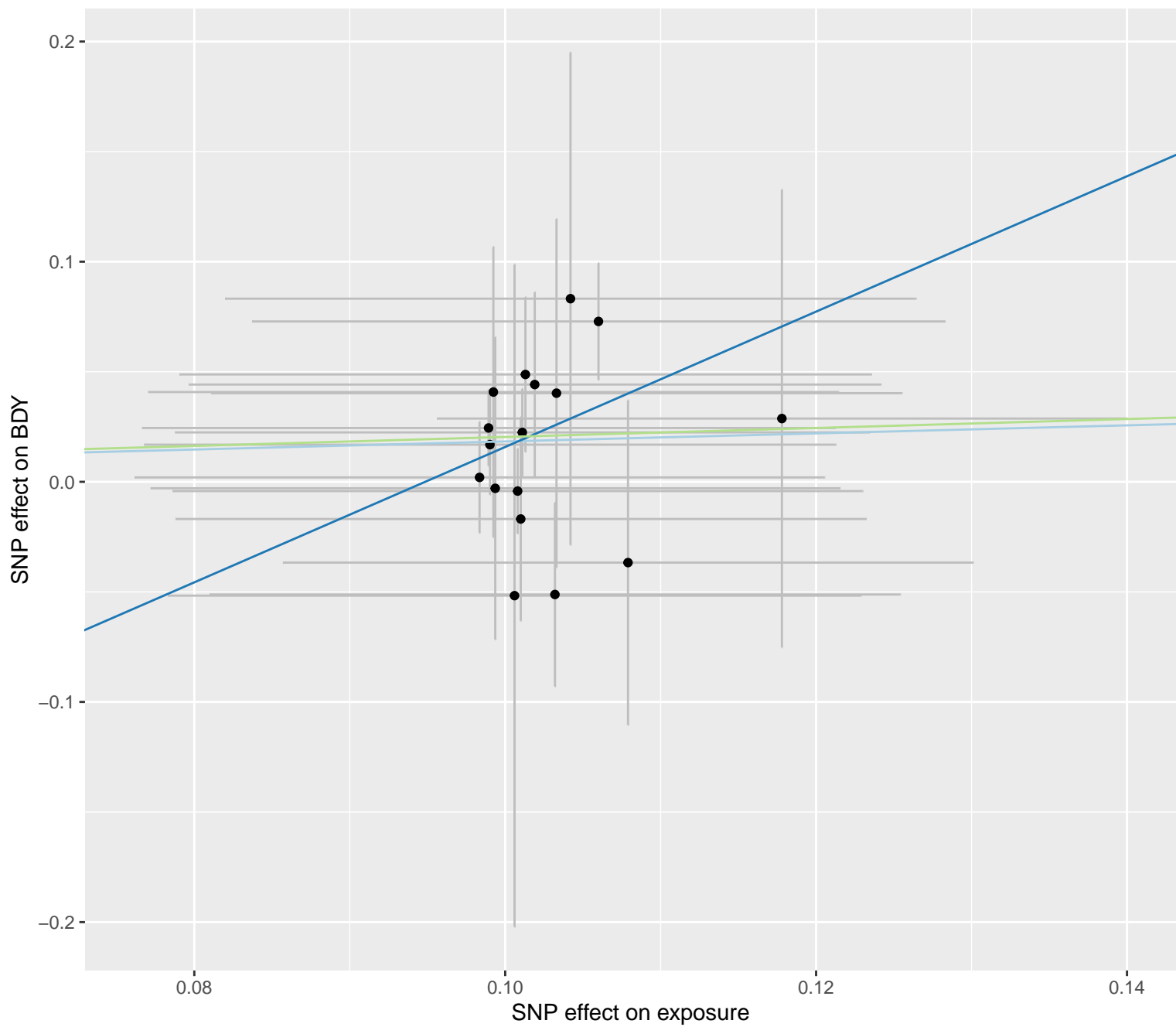

Supplement: Supplementary file 1 [file Data_Sheet_1.zip › Supplementary Materials/MR plots for tongue/Pneumonia/s__Fusobacterium_massiliense_mgs_586/scatter.pdf]

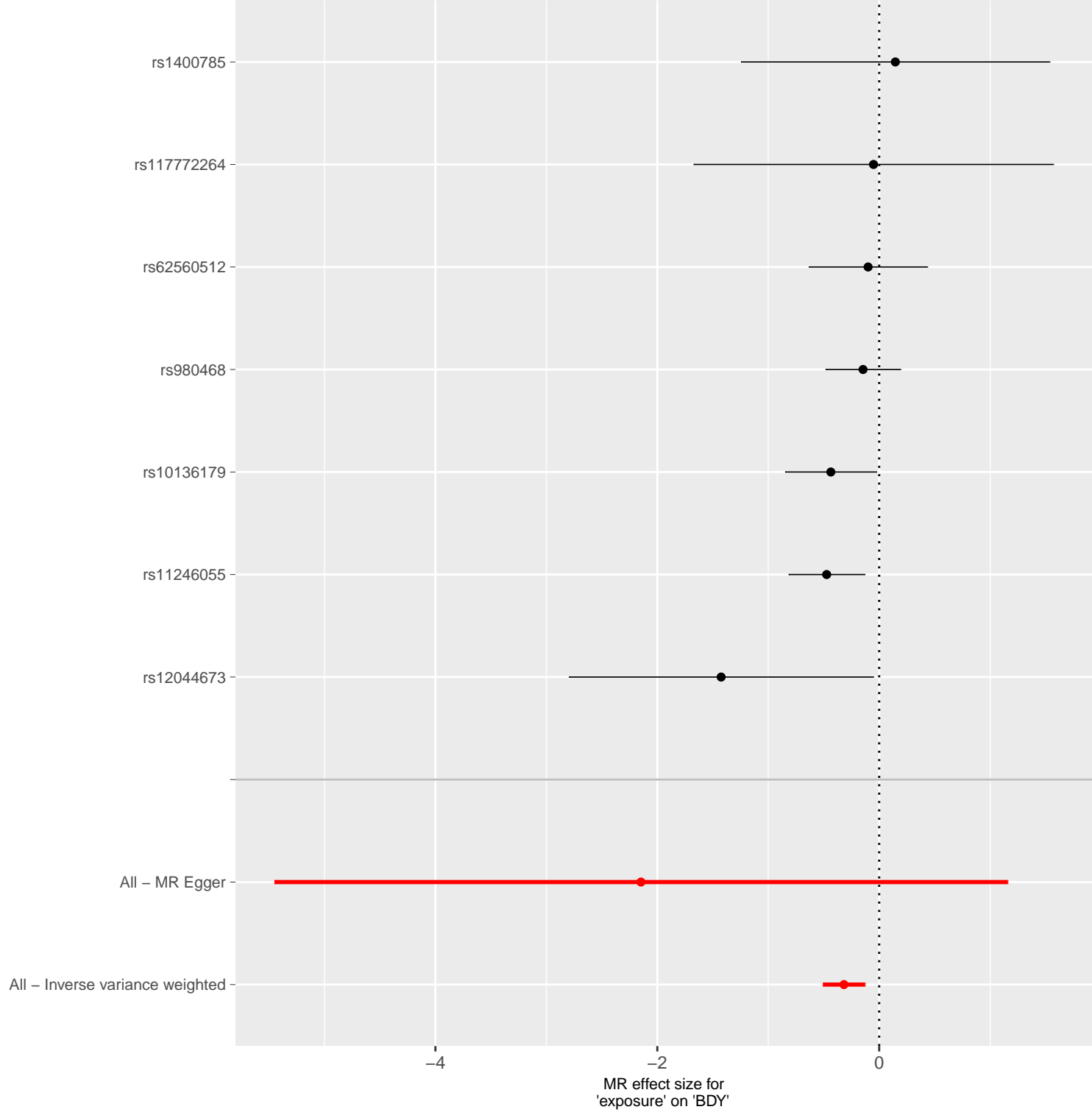

Supplement: Supplementary file 1 [file Data_Sheet_1.zip › Supplementary Materials/MR plots for tongue/Pneumonia/s__Granulicatella_elegans_mgs_2285/forest.pdf]

# MR Method

- Inverse variance weighted
- MR Egger

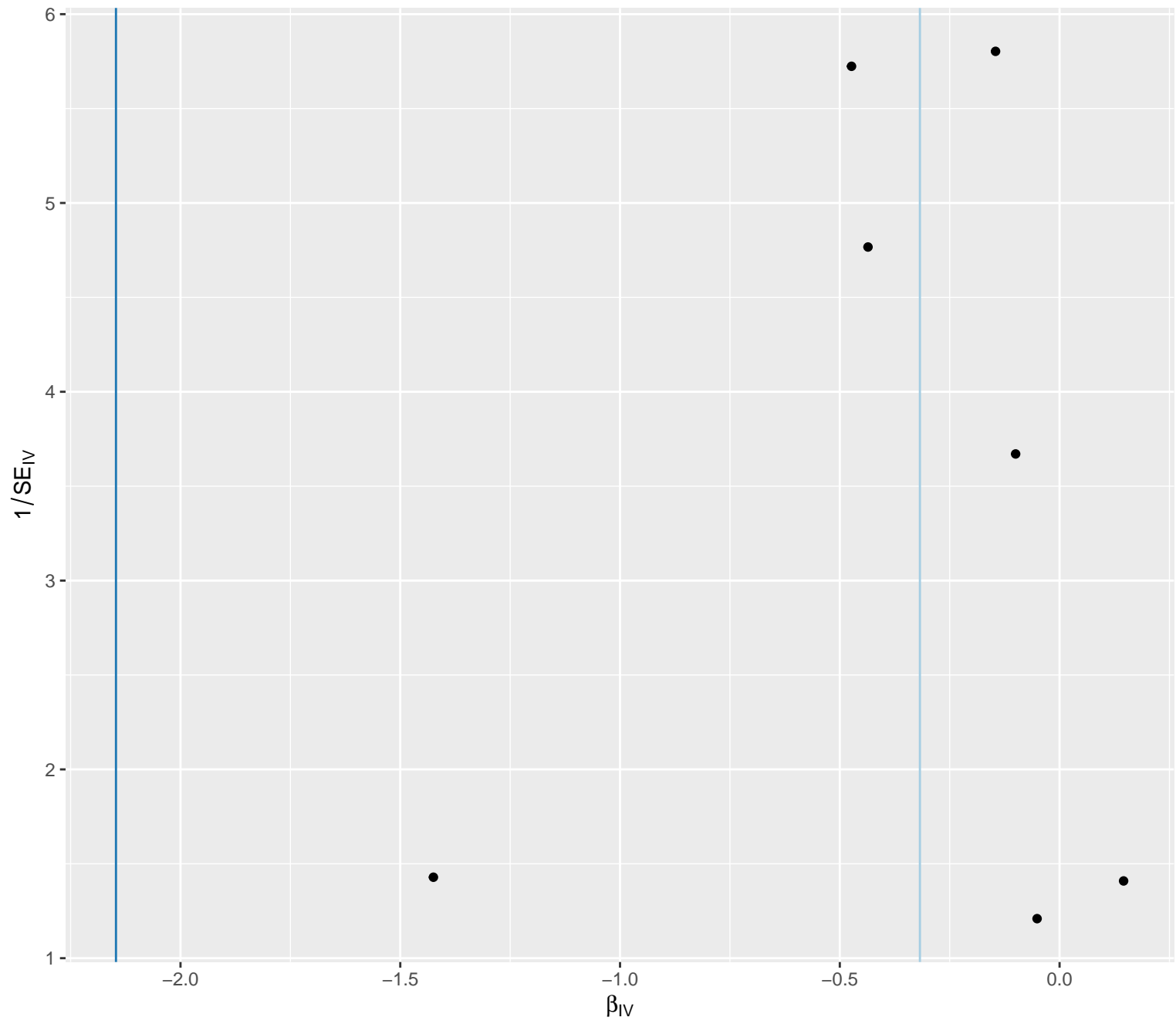

Supplement: Supplementary file 1 [file Data_Sheet_1.zip › Supplementary Materials/MR plots for tongue/Pneumonia/s__Granulicatella_elegans_mgs_2285/funnel.pdf]

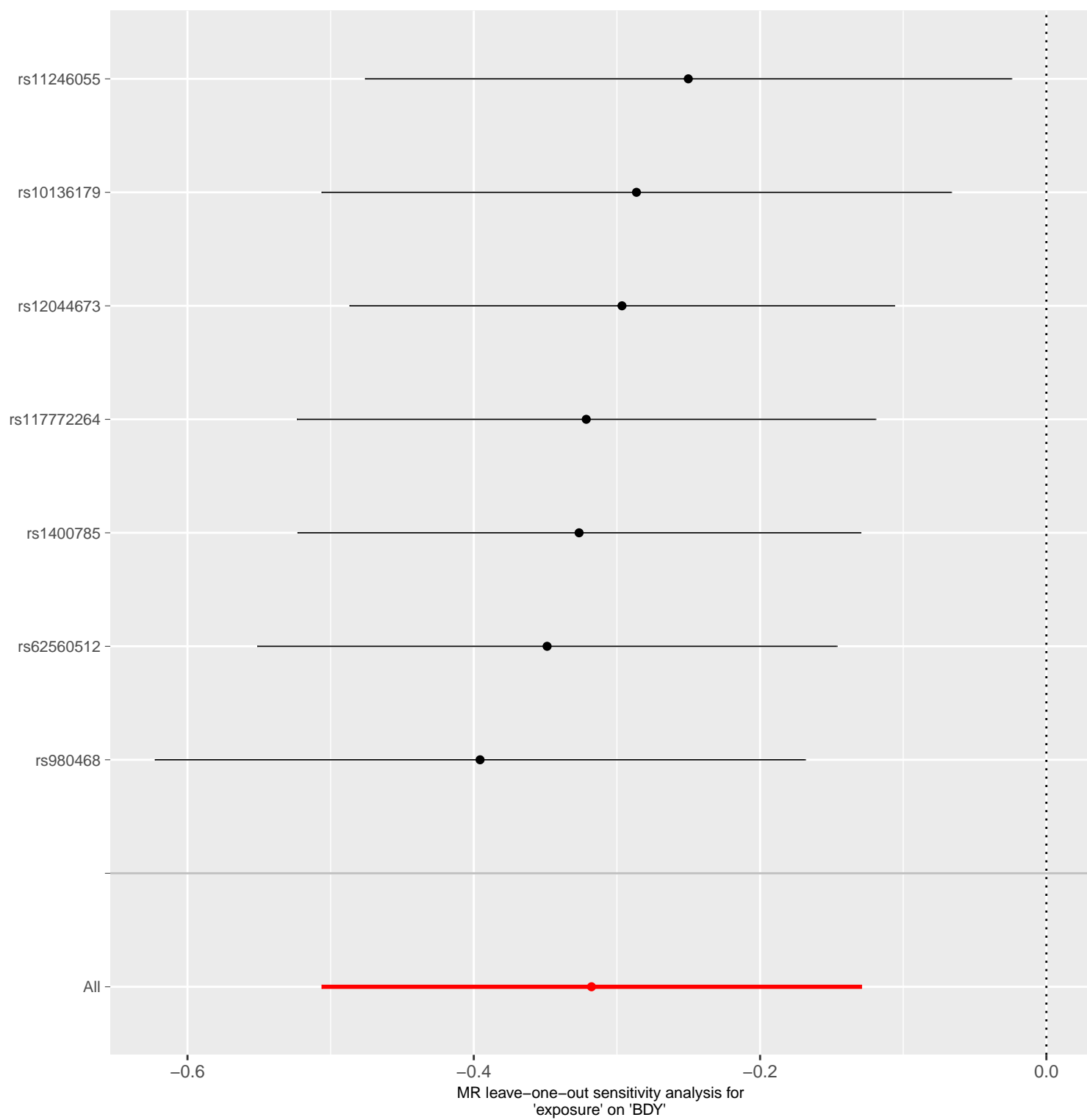

Supplement: Supplementary file 1 [file Data_Sheet_1.zip › Supplementary Materials/MR plots for tongue/Pneumonia/s__Granulicatella_elegans_mgs_2285/leave_one_out.pdf]

# MR Test

- Inverse variance weighted
- MR Egger
- Weighted median

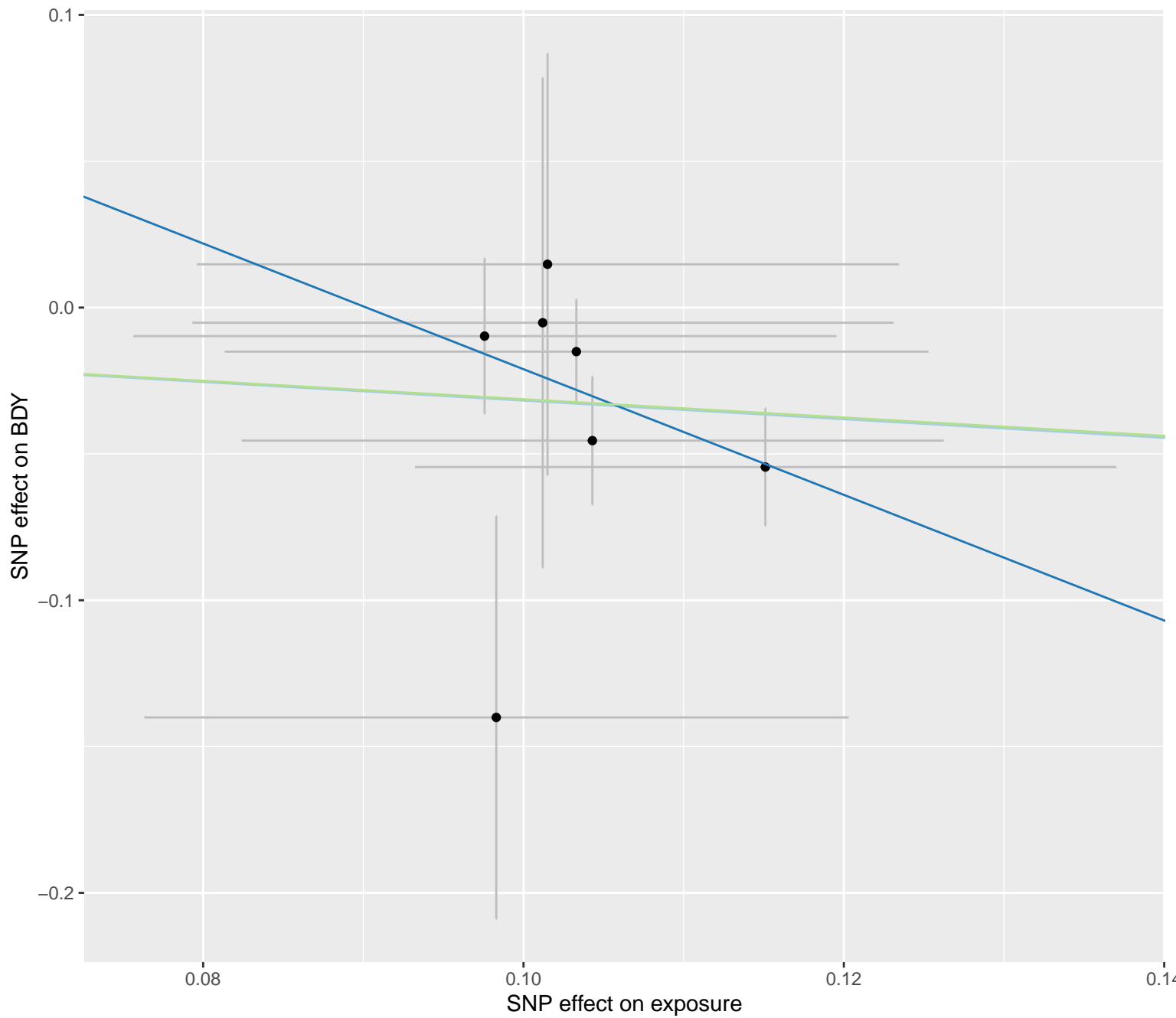

Supplement: Supplementary file 1 [file Data_Sheet_1.zip › Supplementary Materials/MR plots for tongue/Pneumonia/s__Granulicatella_elegans_mgs_2285/scatter.pdf]

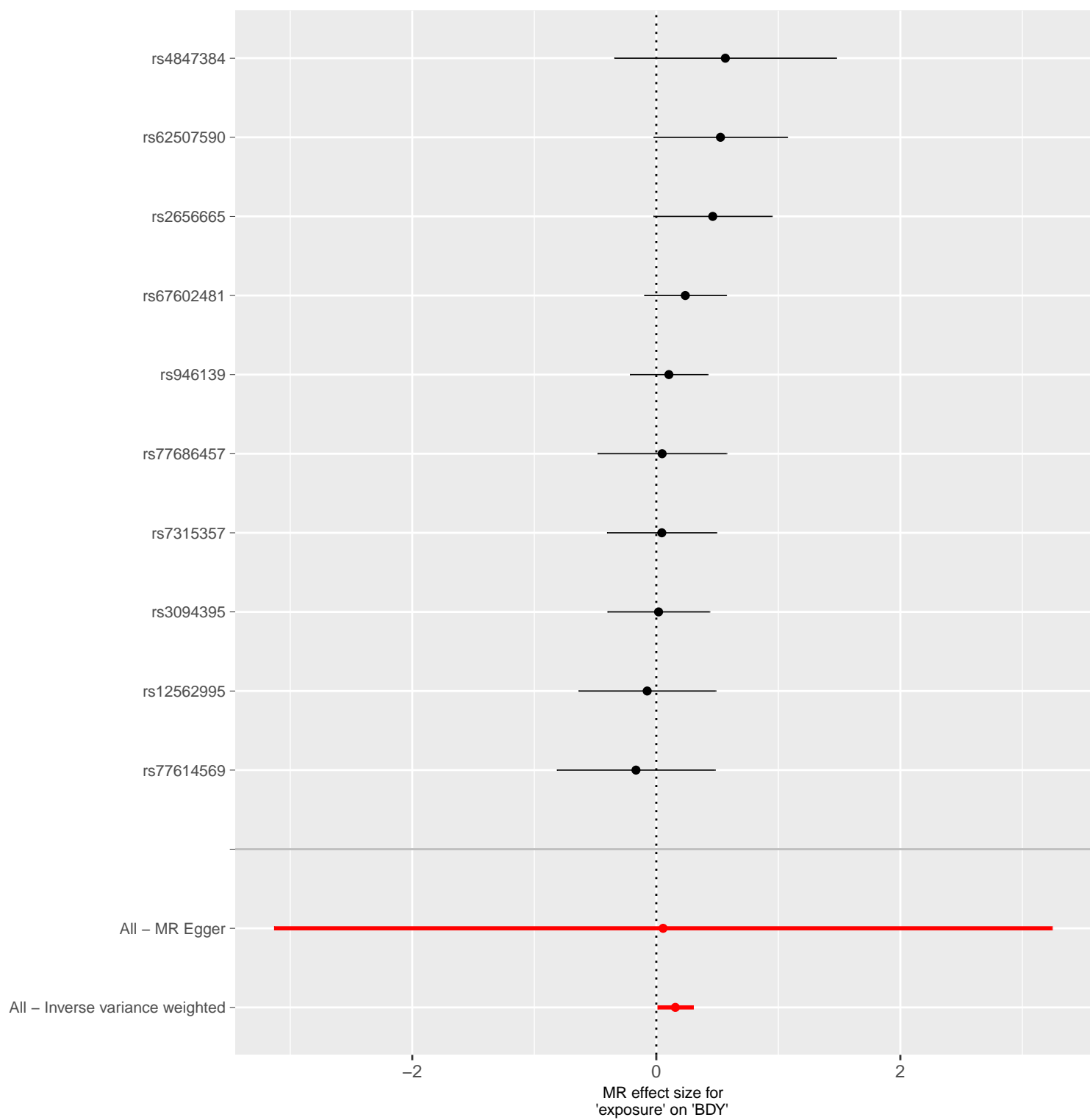

Supplement: Supplementary file 1 [file Data_Sheet_1.zip › Supplementary Materials/MR plots for tongue/Pneumonia/s__HOT-345_sp003260355_mgs_213/forest.pdf]

# MR Method

- Inverse variance weighted
- MR Egger

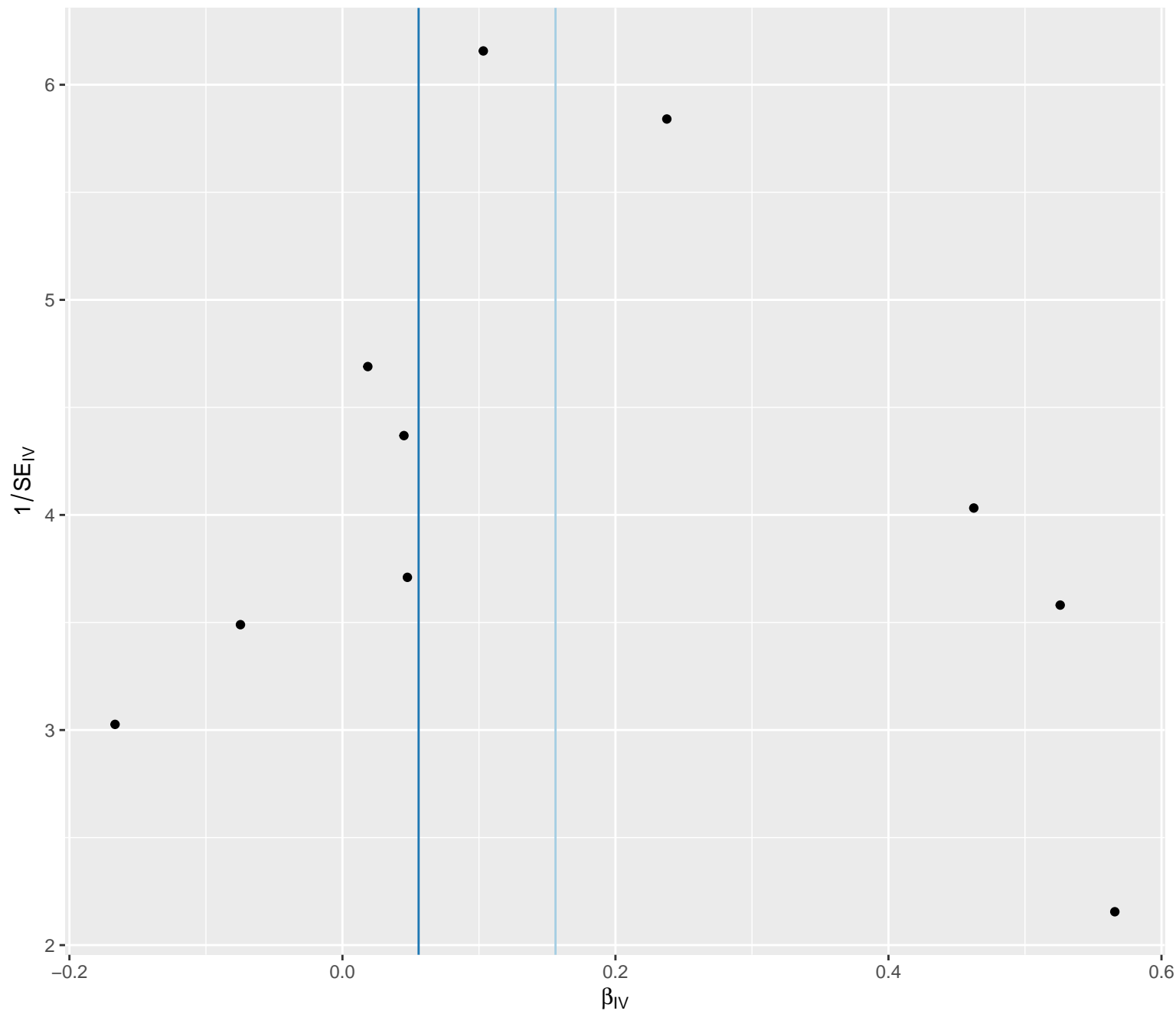

Supplement: Supplementary file 1 [file Data_Sheet_1.zip › Supplementary Materials/MR plots for tongue/Pneumonia/s__HOT-345_sp003260355_mgs_213/funnel.pdf]

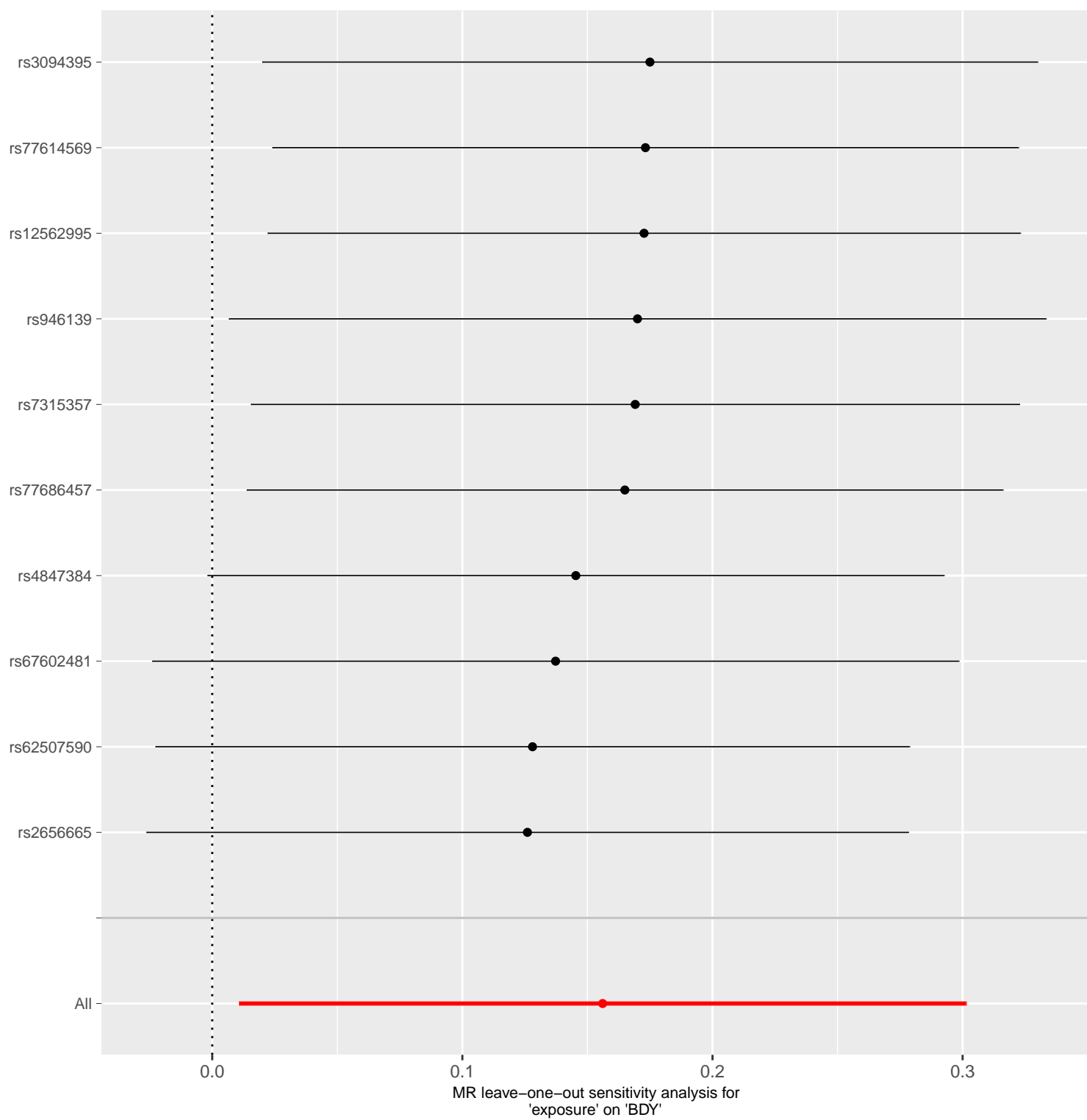

Supplement: Supplementary file 1 [file Data_Sheet_1.zip › Supplementary Materials/MR plots for tongue/Pneumonia/s__HOT-345_sp003260355_mgs_213/leave_one_out.pdf]

# MR Test

- Inverse variance weighted
- MR Egger
- Weighted median

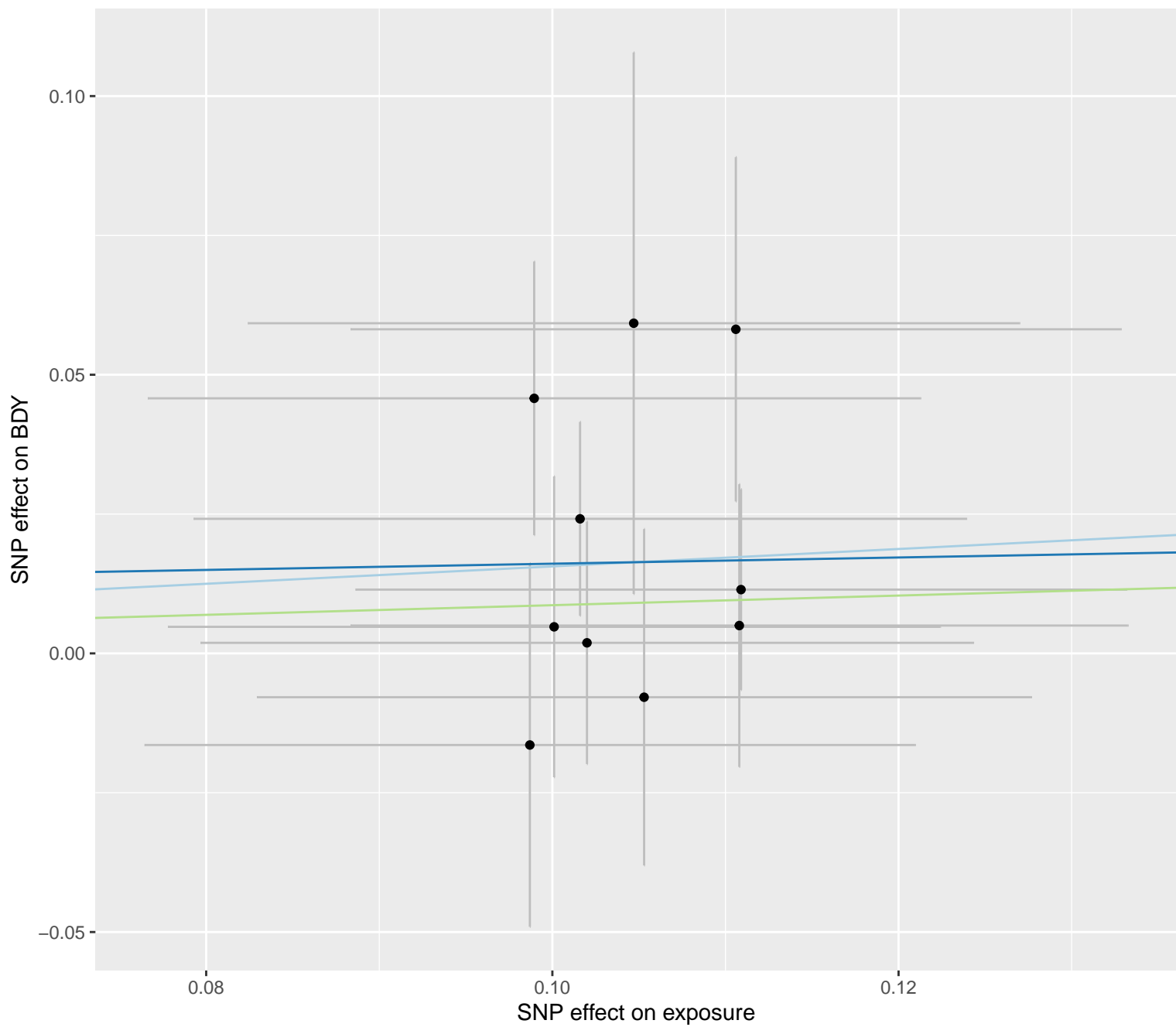

Supplement: Supplementary file 1 [file Data_Sheet_1.zip › Supplementary Materials/MR plots for tongue/Pneumonia/s__HOT-345_sp003260355_mgs_213/scatter.pdf]

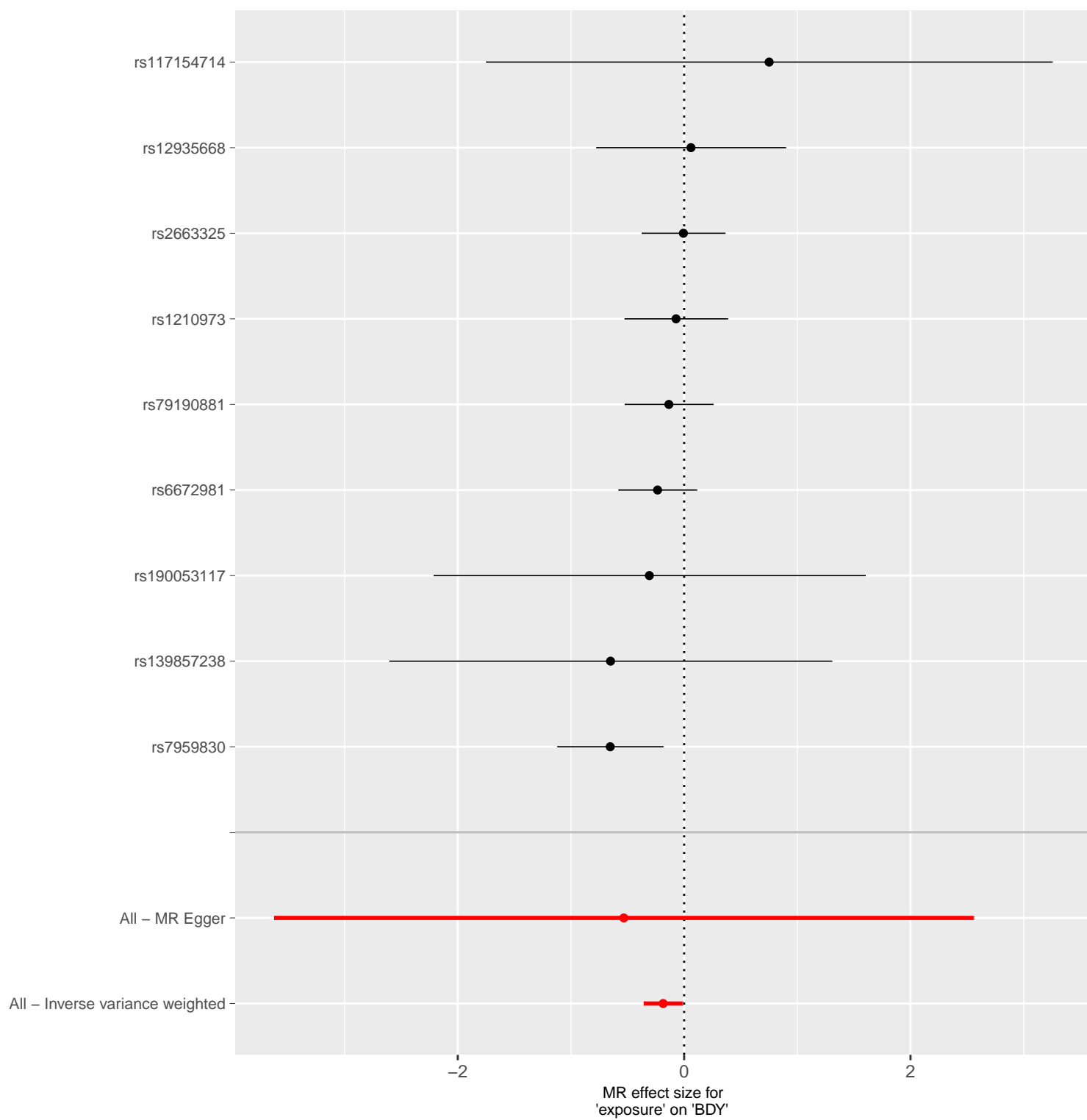

Supplement: Supplementary file 1 [file Data_Sheet_1.zip › Supplementary Materials/MR plots for tongue/Pneumonia/s__Haemophilus_D_parainfluenzae_A_mgs_2988/forest.pdf]

# MR Method

- Inverse variance weighted
- MR Egger

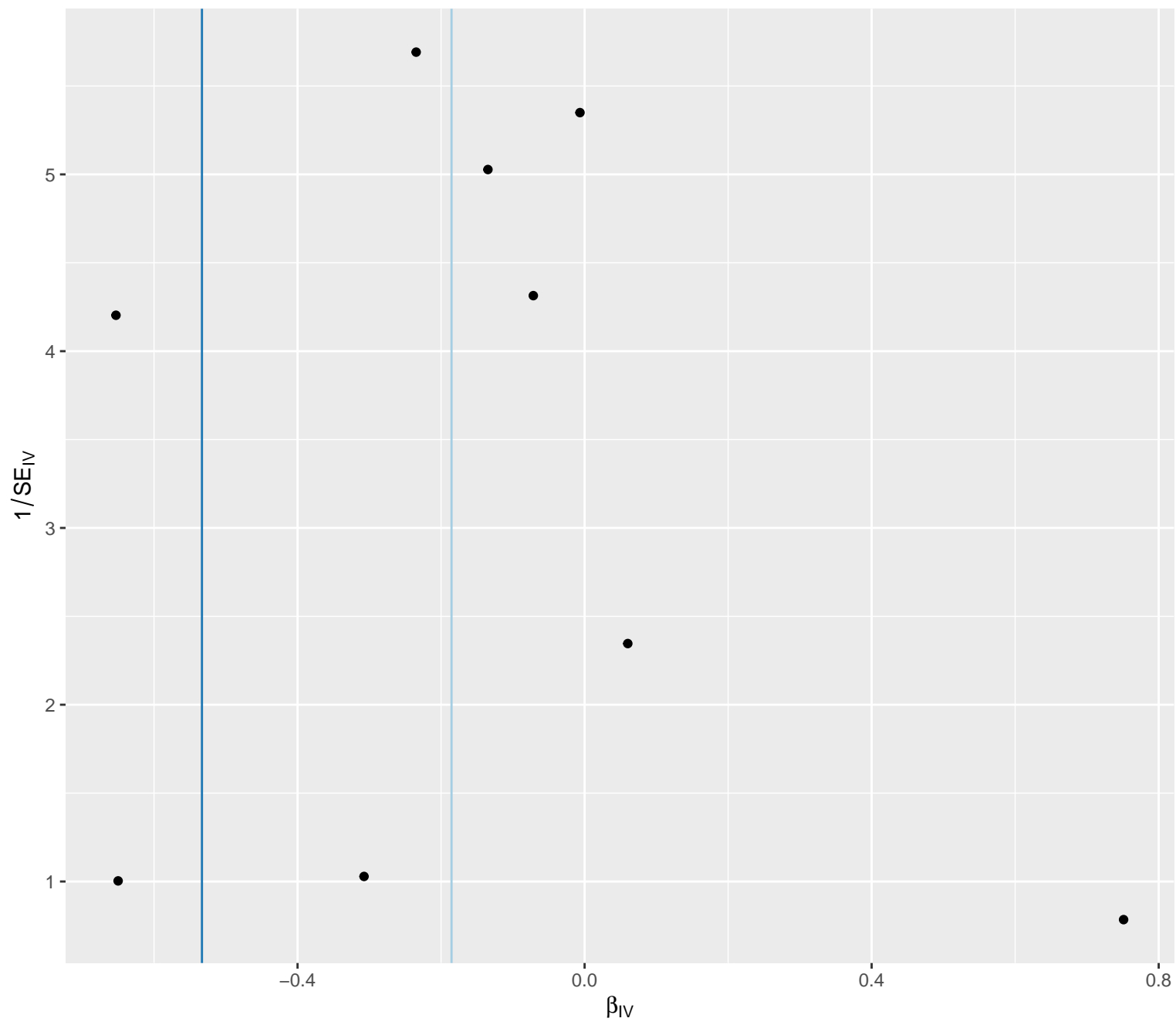

Supplement: Supplementary file 1 [file Data_Sheet_1.zip › Supplementary Materials/MR plots for tongue/Pneumonia/s__Haemophilus_D_parainfluenzae_A_mgs_2988/funnel.pdf]

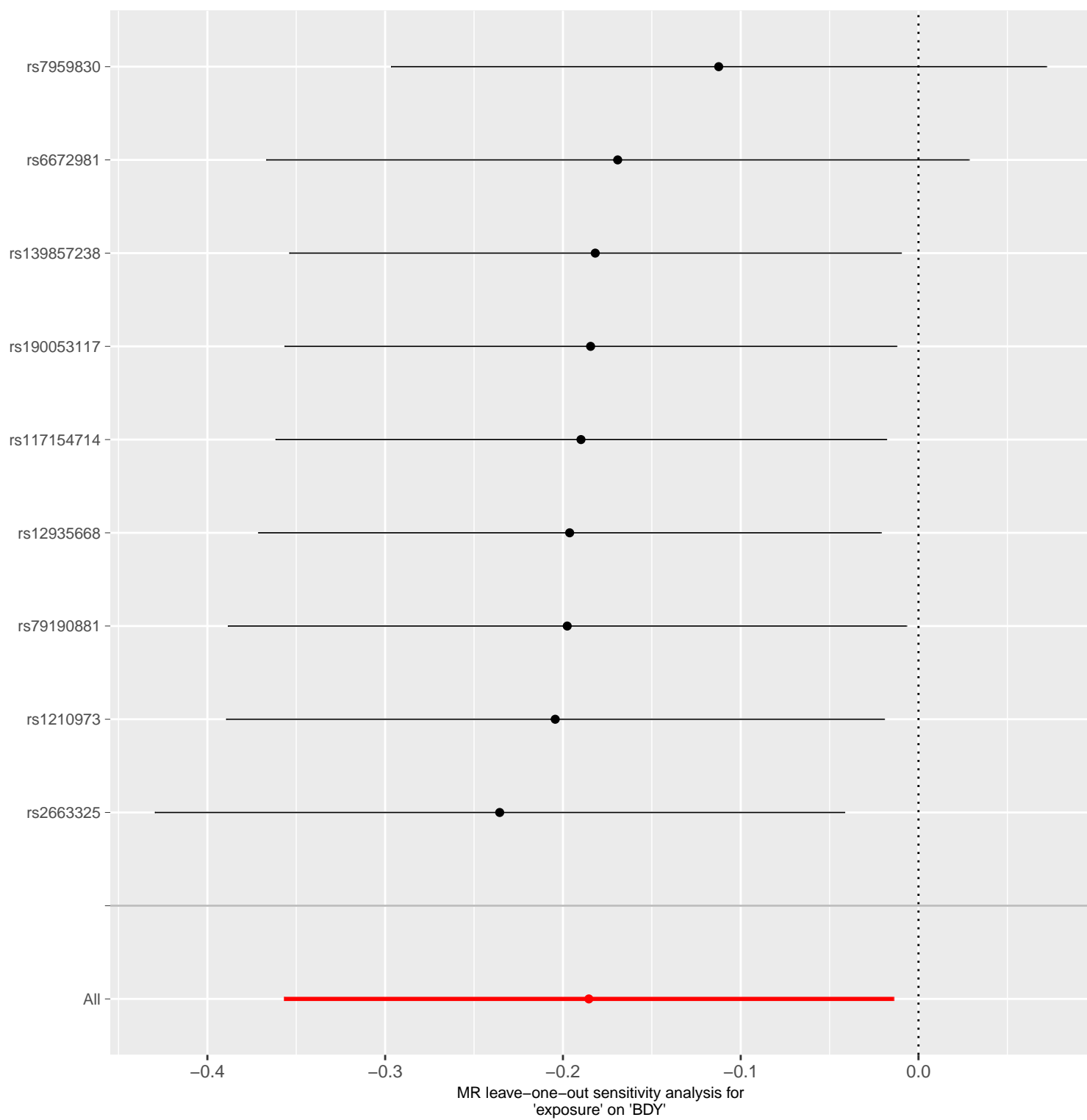

Supplement: Supplementary file 1 [file Data_Sheet_1.zip › Supplementary Materials/MR plots for tongue/Pneumonia/s__Haemophilus_D_parainfluenzae_A_mgs_2988/leave_one_out.pdf]

# MR Test

- Inverse variance weighted
- MR Egger
- Weighted median

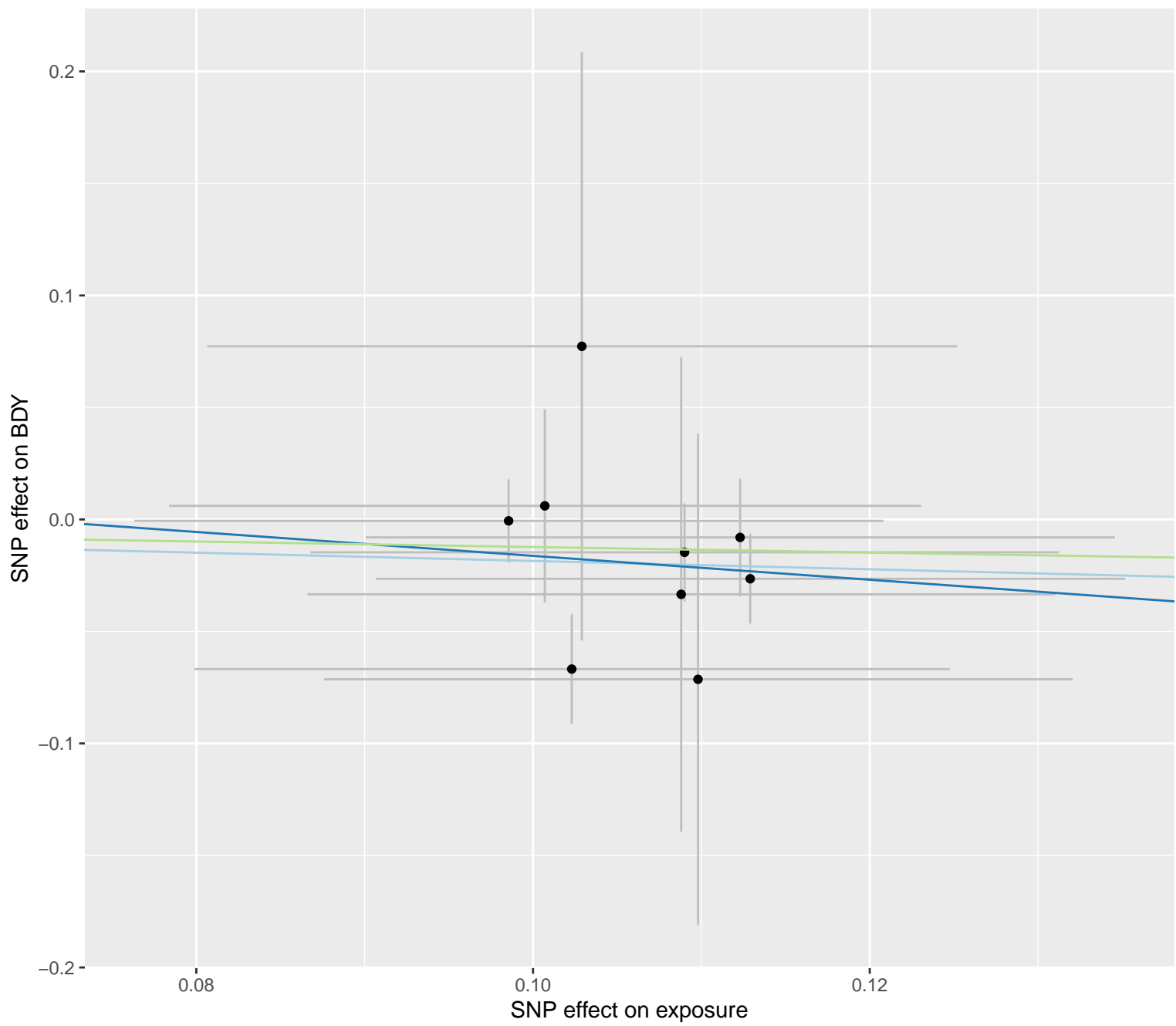

Supplement: Supplementary file 1 [file Data_Sheet_1.zip › Supplementary Materials/MR plots for tongue/Pneumonia/s__Haemophilus_D_parainfluenzae_A_mgs_2988/scatter.pdf]

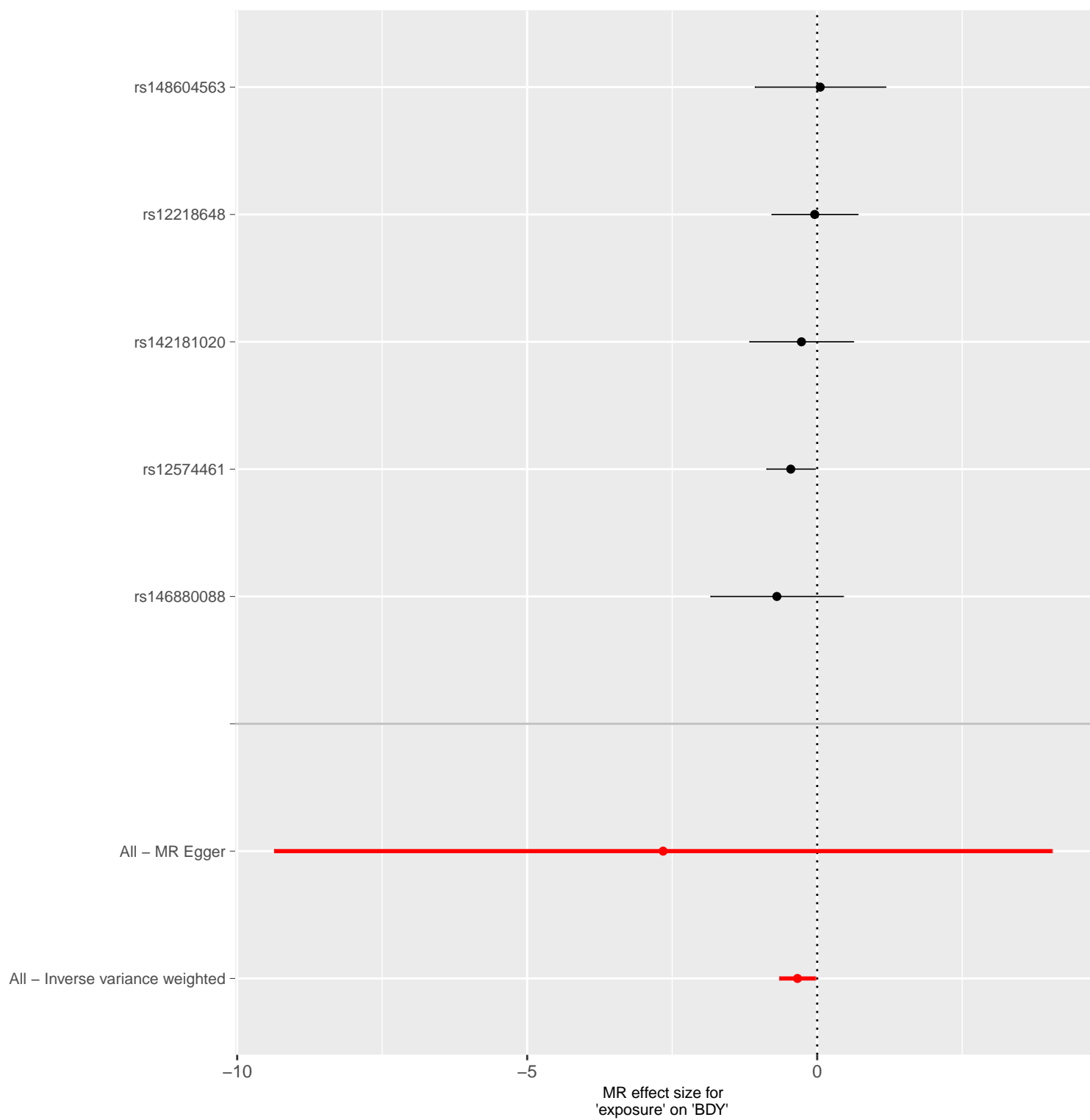

Supplement: Supplementary file 1 [file Data_Sheet_1.zip › Supplementary Materials/MR plots for tongue/Pneumonia/s__Prevotella_oulorum_mgs_3240/forest.pdf]

# MR Method

- Inverse variance weighted
- MR Egger

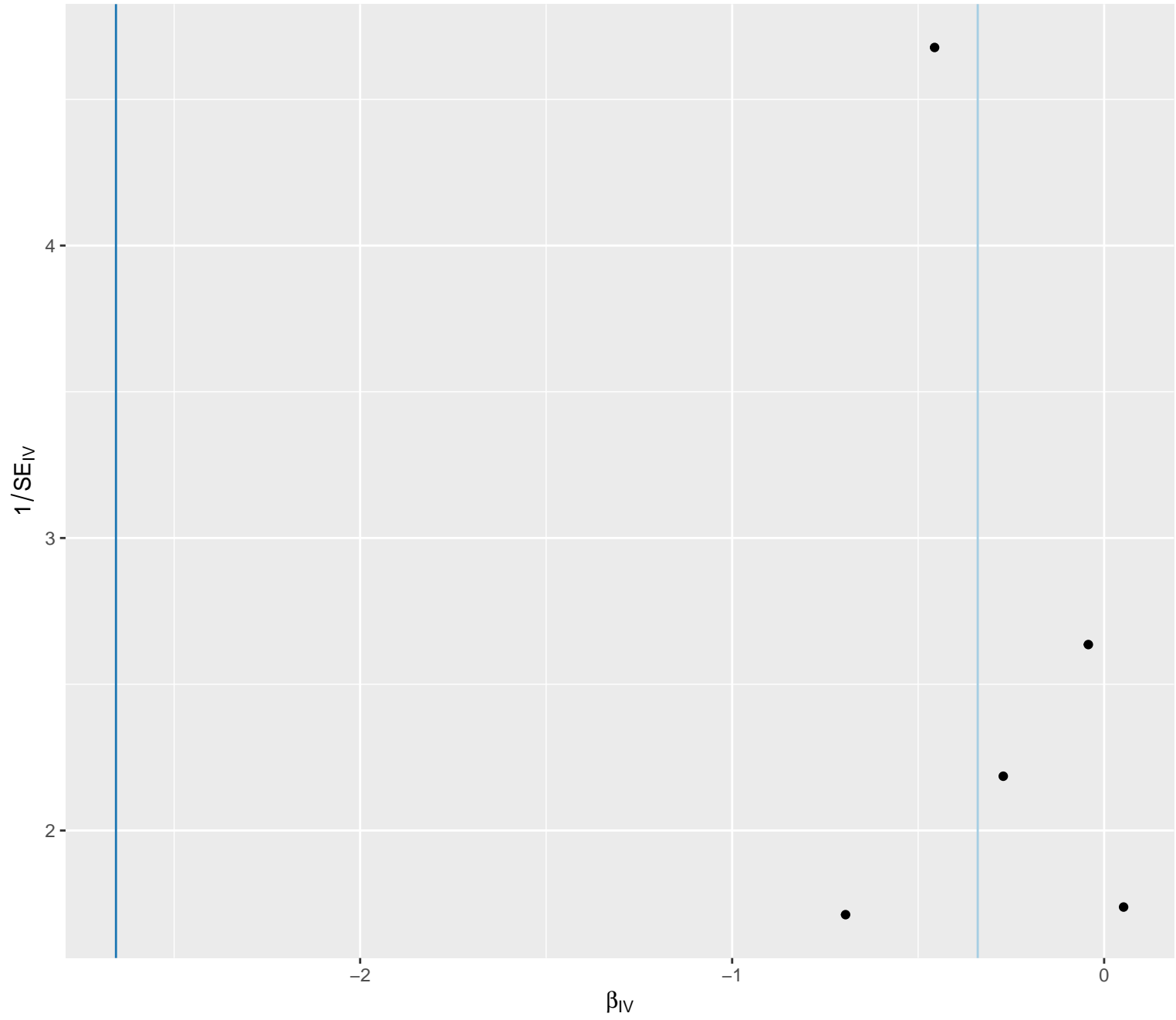

Supplement: Supplementary file 1 [file Data_Sheet_1.zip › Supplementary Materials/MR plots for tongue/Pneumonia/s__Prevotella_oulorum_mgs_3240/funnel.pdf]

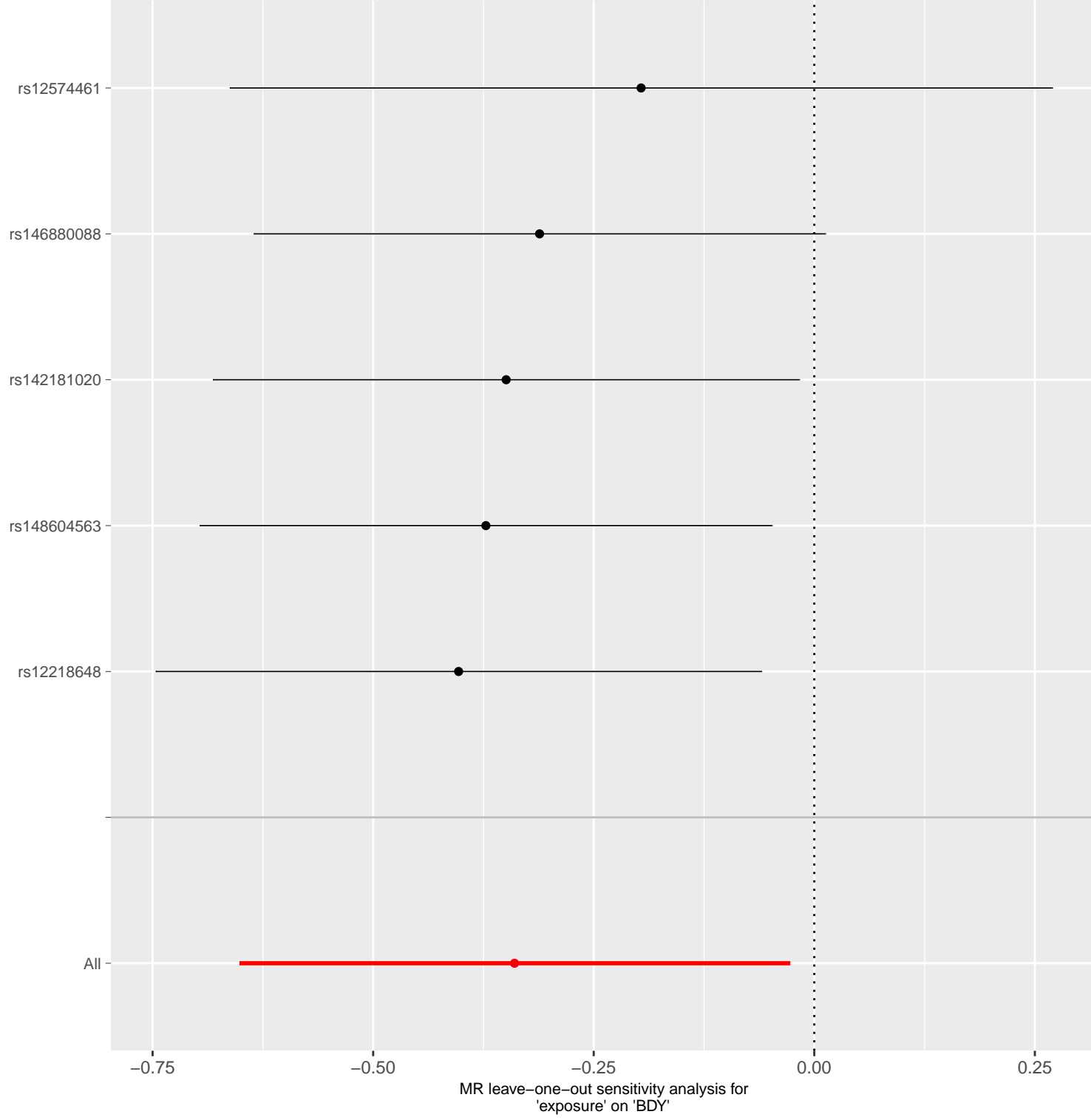

Supplement: Supplementary file 1 [file Data_Sheet_1.zip › Supplementary Materials/MR plots for tongue/Pneumonia/s__Prevotella_oulorum_mgs_3240/leave_one_out.pdf]

# MR Test

- Inverse variance weighted
- MR Egger
- Weighted median

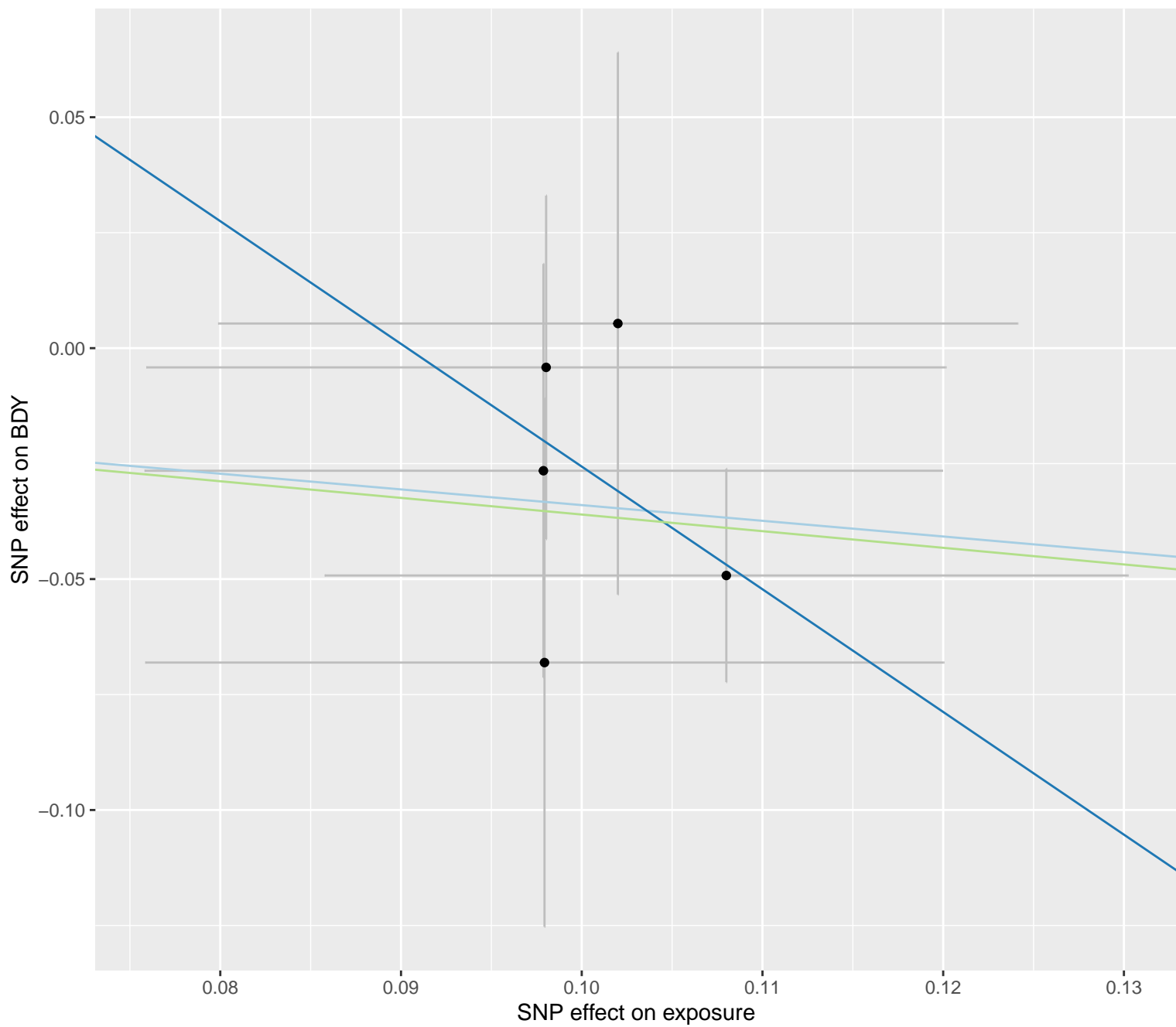

Supplement: Supplementary file 1 [file Data_Sheet_1.zip › Supplementary Materials/MR plots for tongue/Pneumonia/s__Prevotella_oulorum_mgs_3240/scatter.pdf]

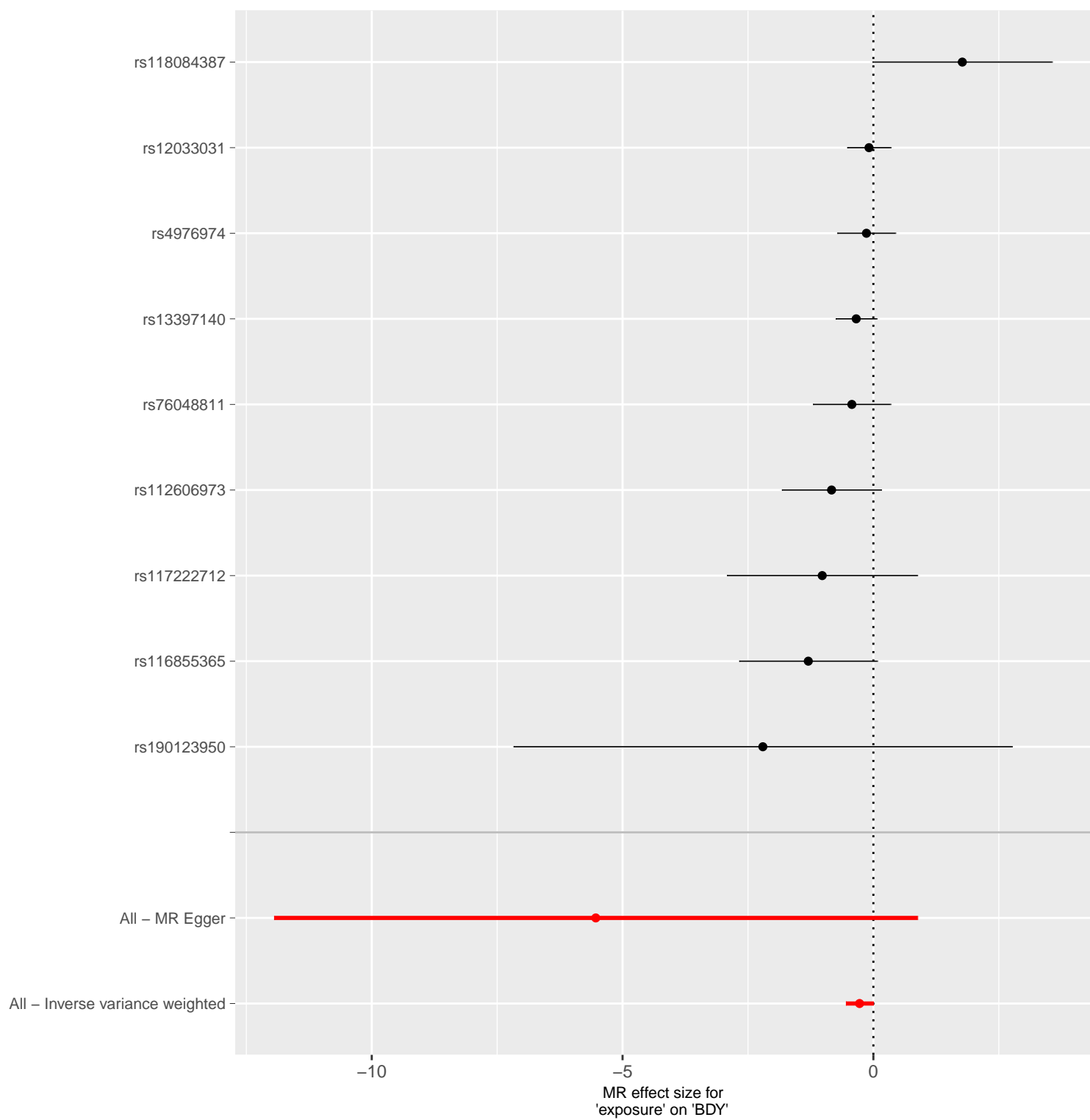

Supplement: Supplementary file 1 [file Data_Sheet_1.zip › Supplementary Materials/MR plots for tongue/Pneumonia/s__Prevotella_pleuritidis_mgs_61/forest.pdf]

# MR Method

- Inverse variance weighted
- MR Egger

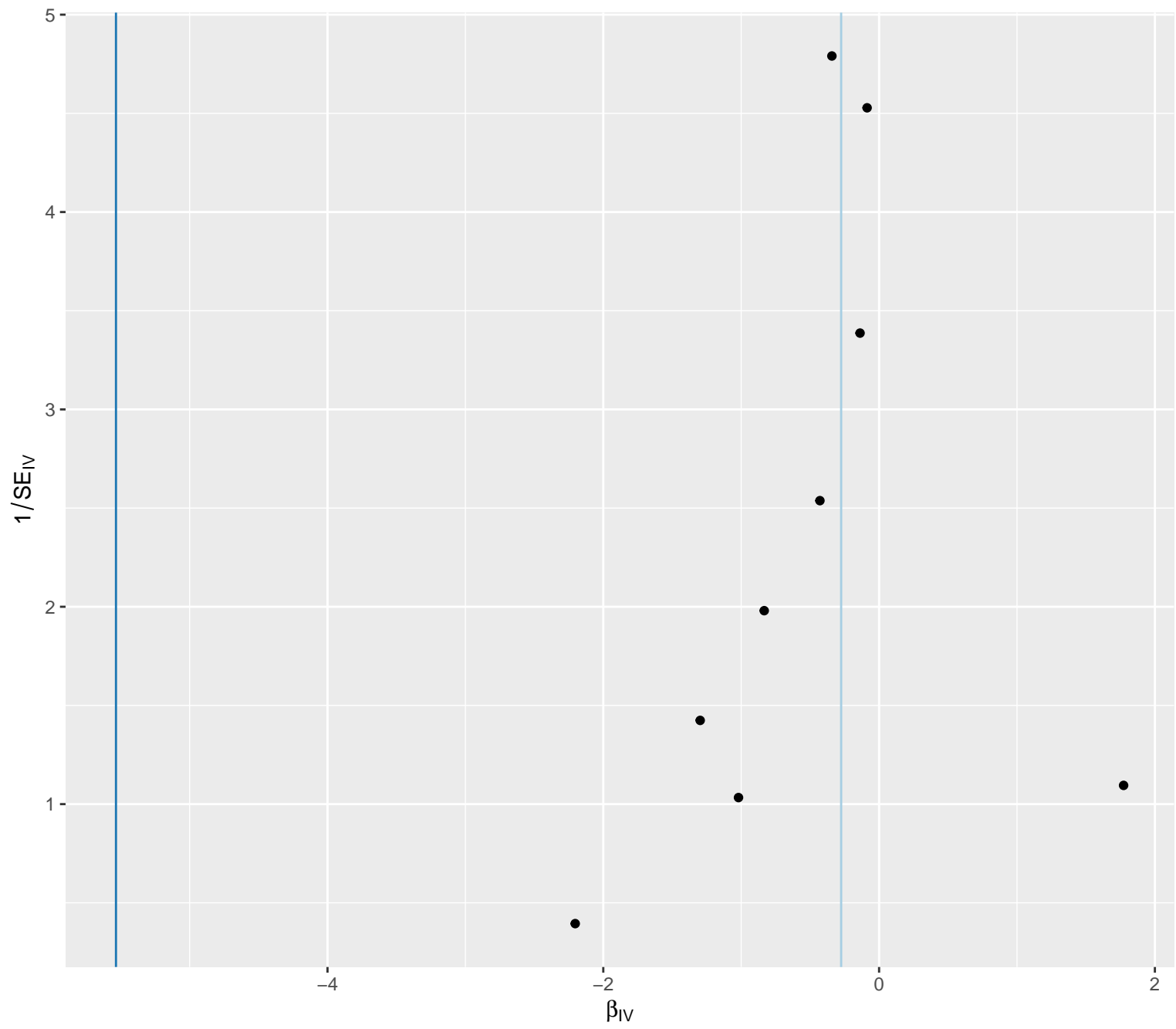

Supplement: Supplementary file 1 [file Data_Sheet_1.zip › Supplementary Materials/MR plots for tongue/Pneumonia/s__Prevotella_pleuritidis_mgs_61/funnel.pdf]

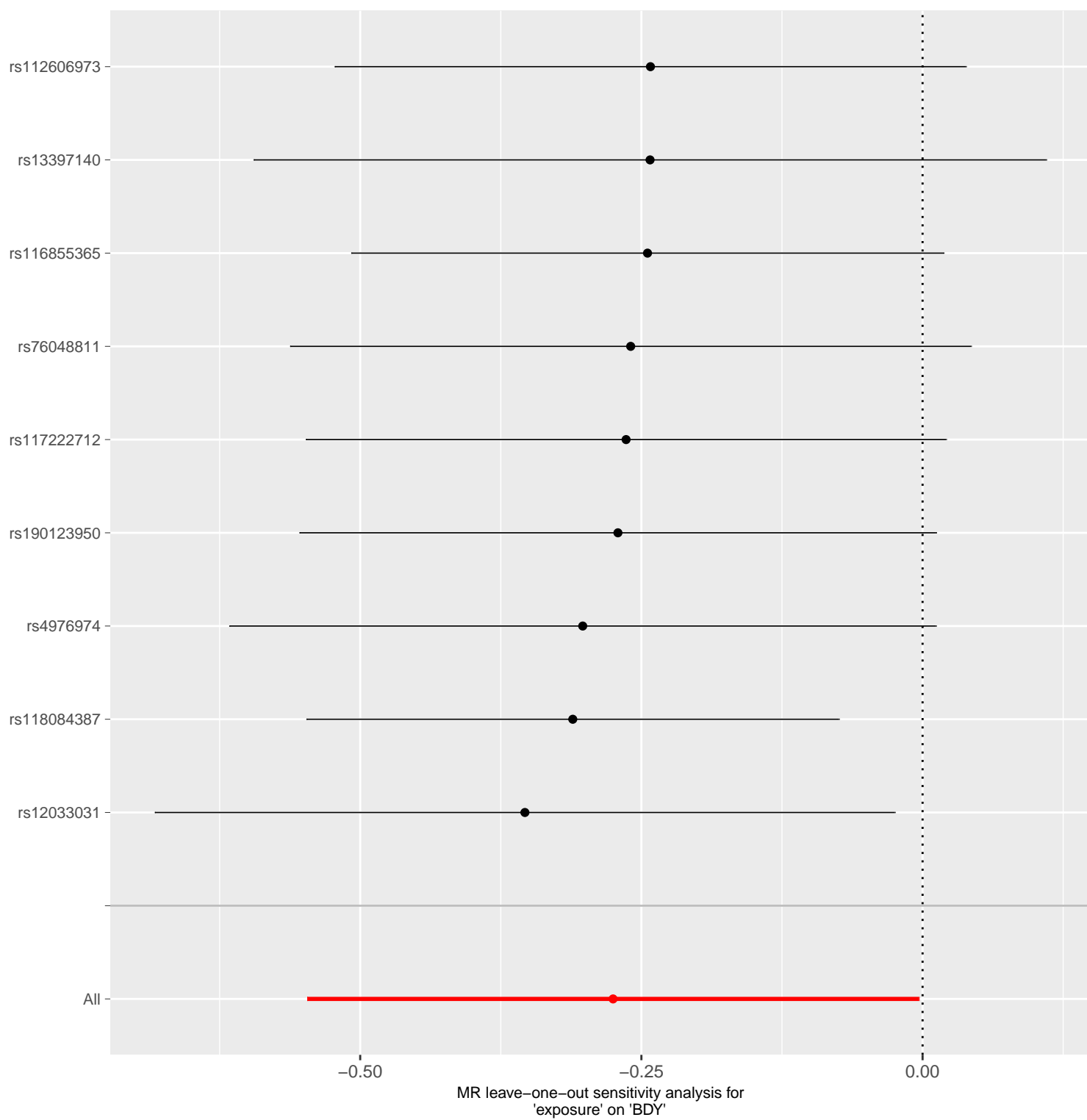

Supplement: Supplementary file 1 [file Data_Sheet_1.zip › Supplementary Materials/MR plots for tongue/Pneumonia/s__Prevotella_pleuritidis_mgs_61/leave_one_out.pdf]

# MR Test

- Inverse variance weighted
- Weighted median
- MR Egger

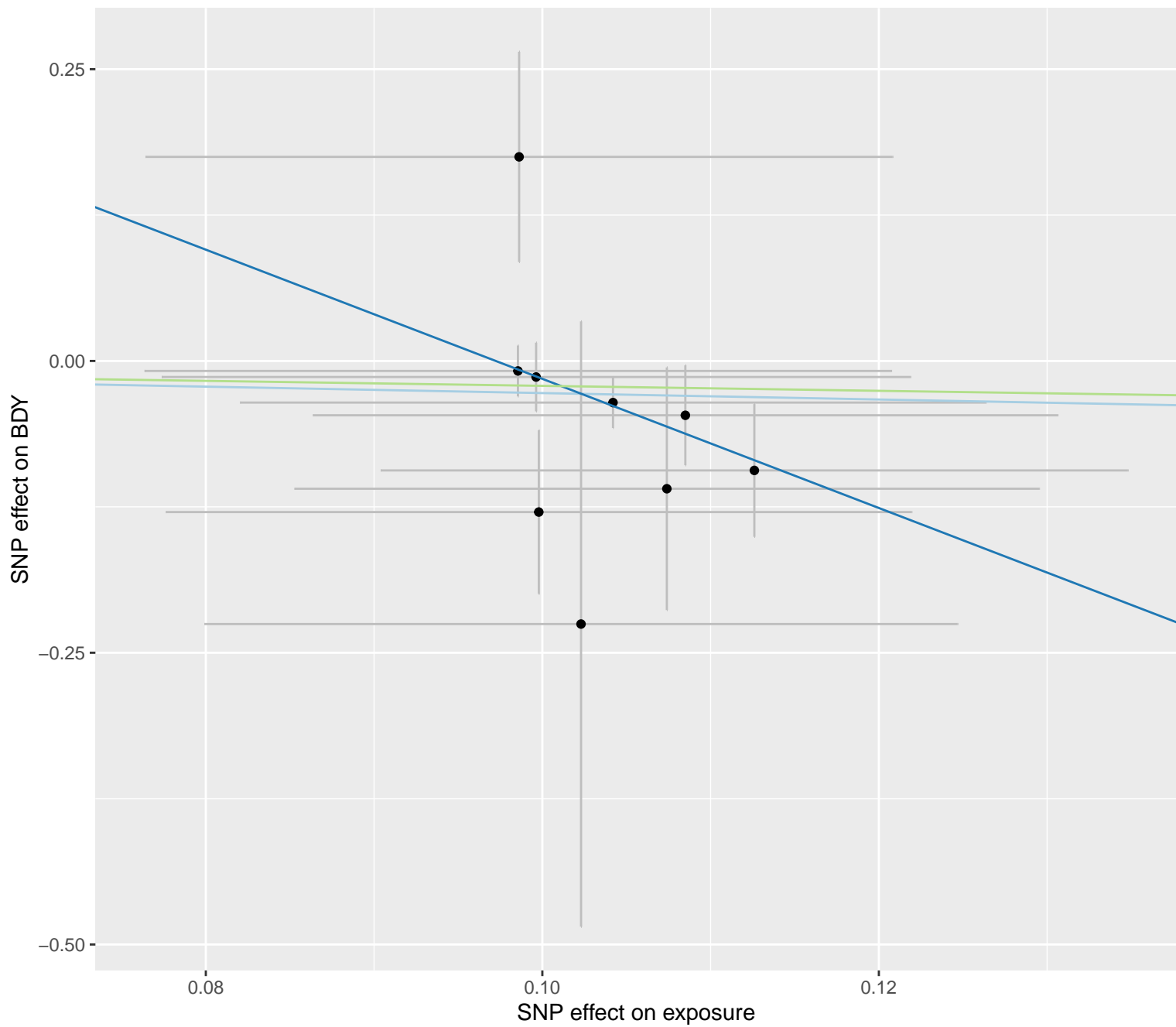

Supplement: Supplementary file 1 [file Data_Sheet_1.zip › Supplementary Materials/MR plots for tongue/Pneumonia/s__Prevotella_pleuritidis_mgs_61/scatter.pdf]

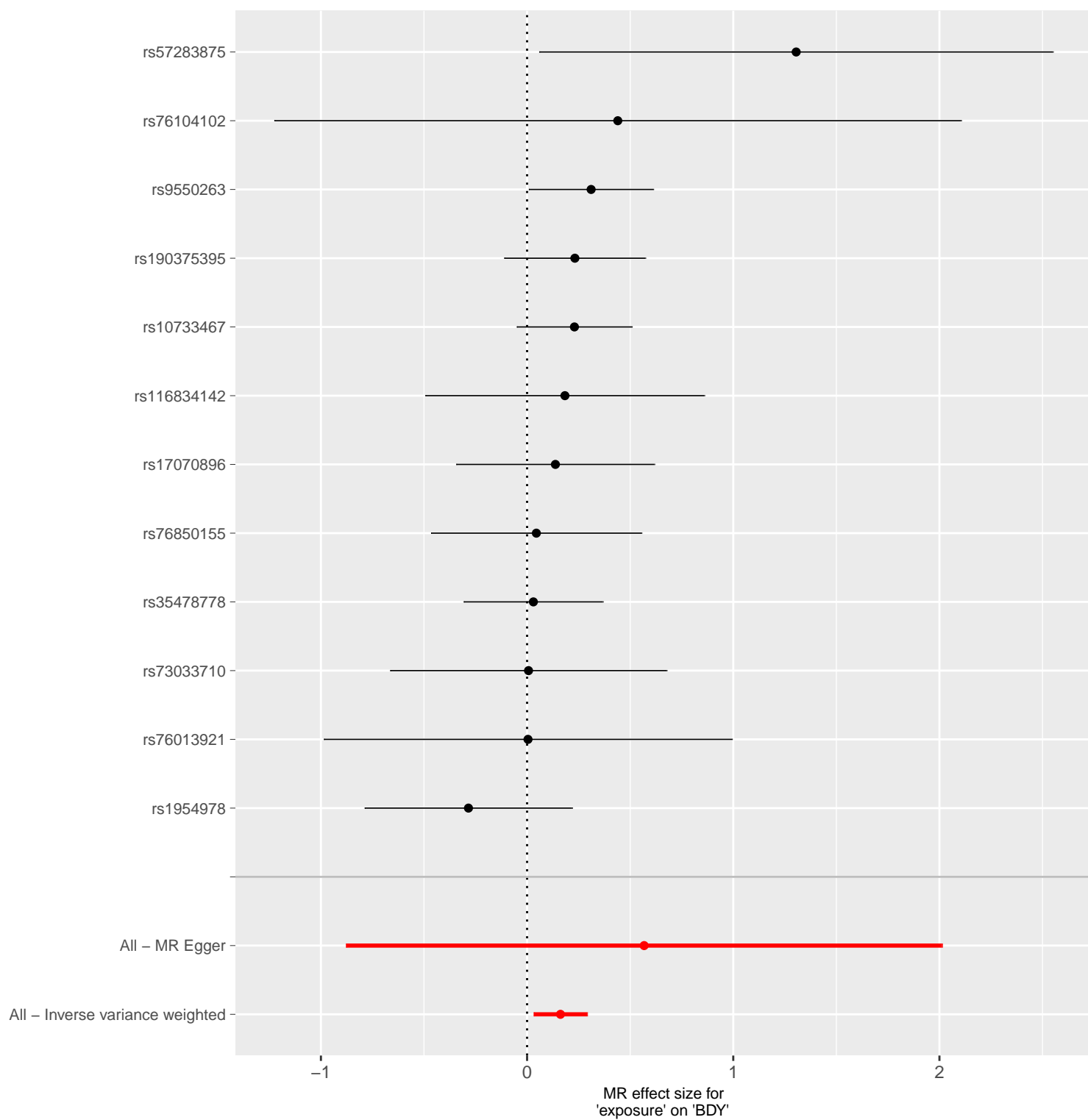

Supplement: Supplementary file 1 [file Data_Sheet_1.zip › Supplementary Materials/MR plots for tongue/Pneumonia/s__Simonsiella_muelleri_mgs_3058/forest.pdf]

# MR Method

- Inverse variance weighted
- MR Egger

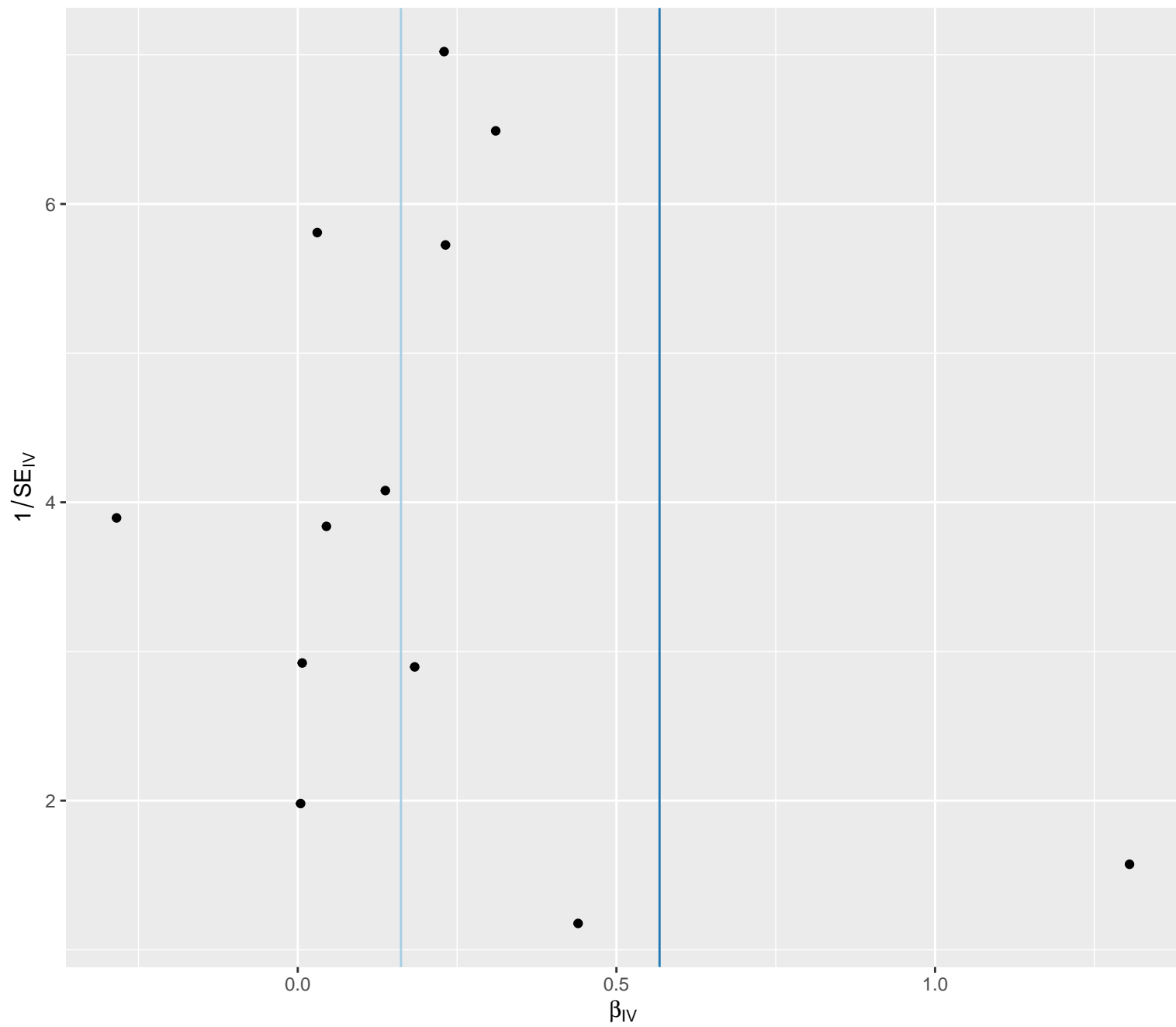

Supplement: Supplementary file 1 [file Data_Sheet_1.zip › Supplementary Materials/MR plots for tongue/Pneumonia/s__Simonsiella_muelleri_mgs_3058/funnel.pdf]

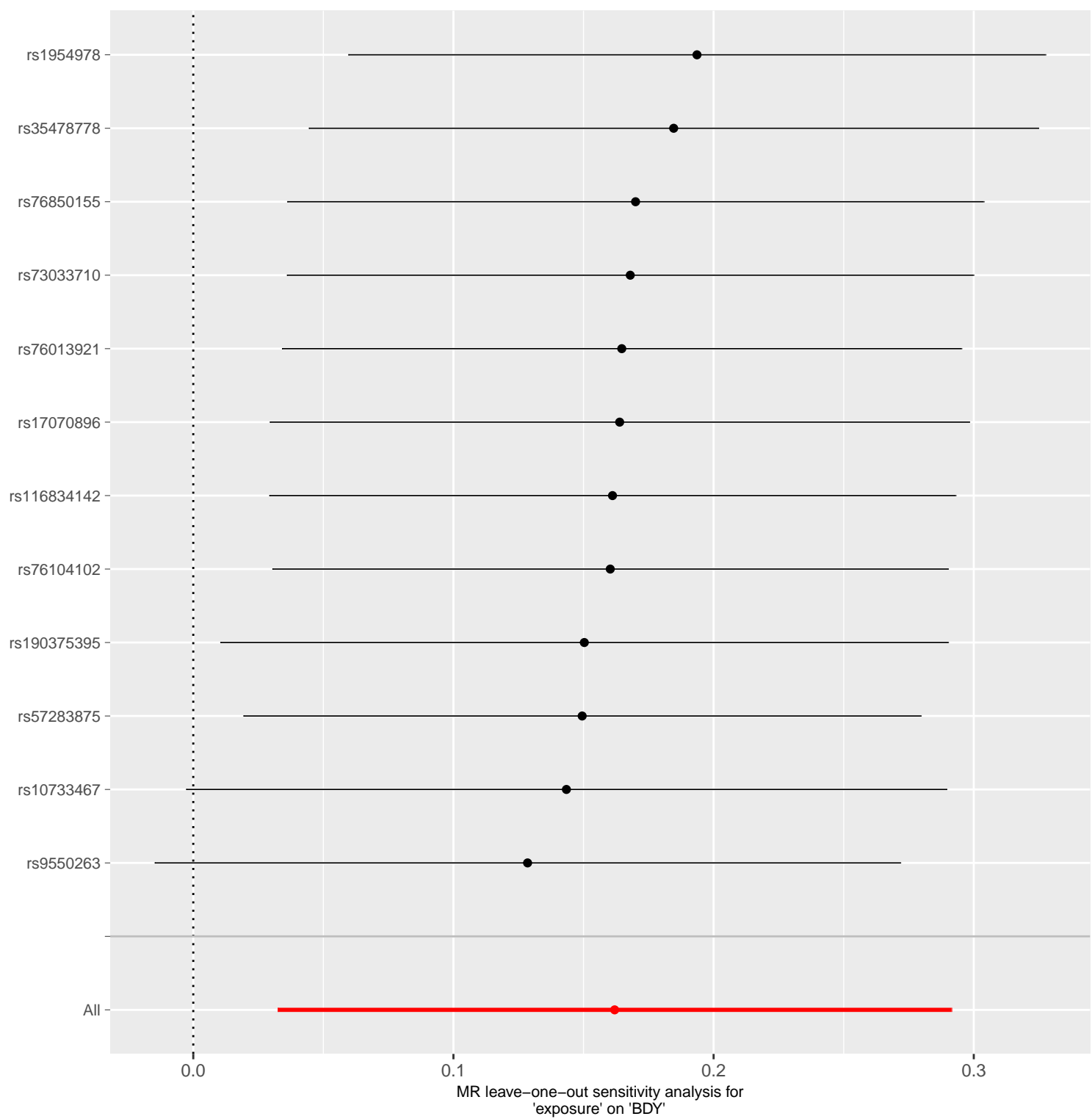

Supplement: Supplementary file 1 [file Data_Sheet_1.zip › Supplementary Materials/MR plots for tongue/Pneumonia/s__Simonsiella_muelleri_mgs_3058/leave_one_out.pdf]

# MR Test

- Inverse variance weighted
- MR Egger
- Weighted median

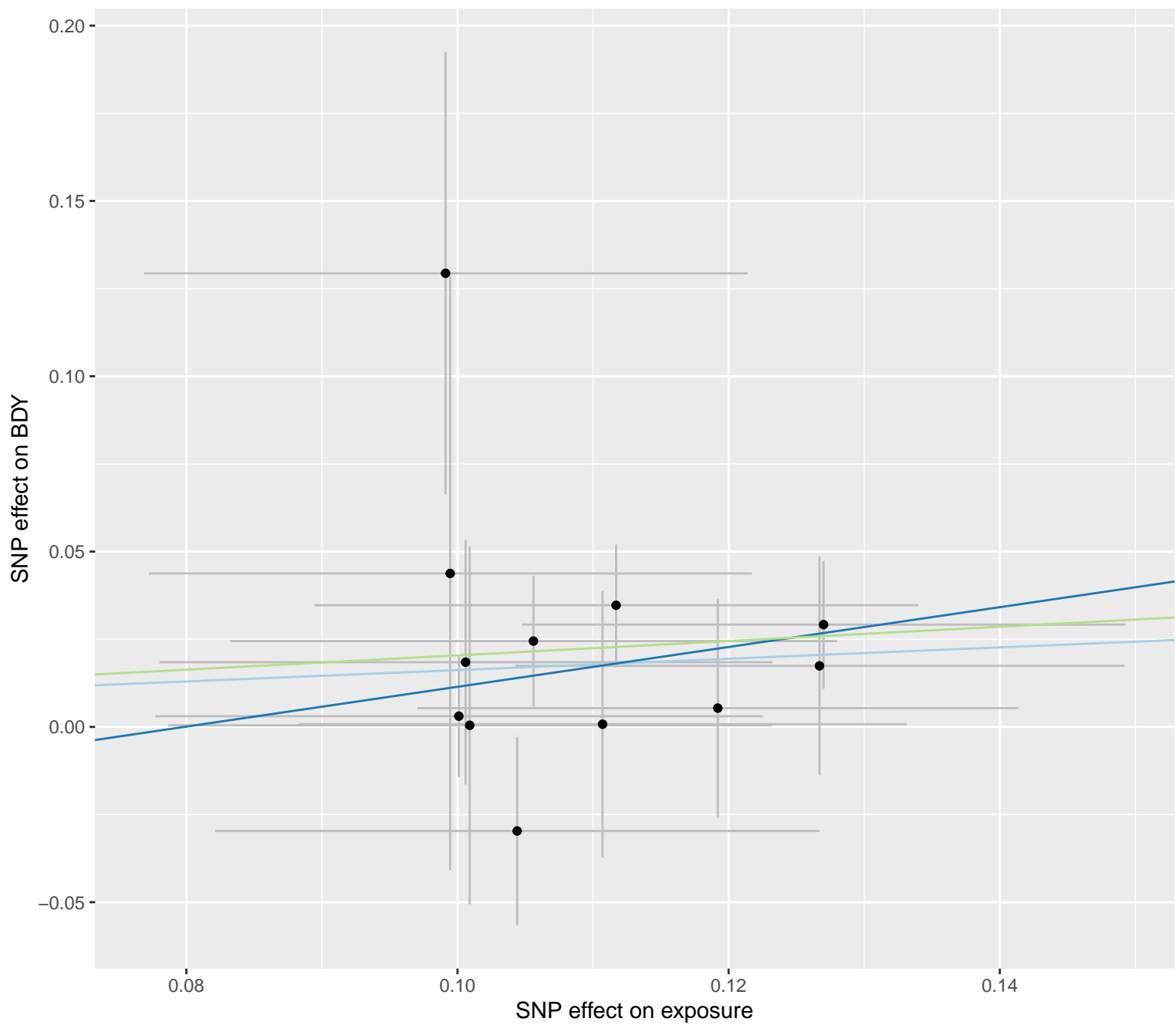

Supplement: Supplementary file 1 [file Data_Sheet_1.zip › Supplementary Materials/MR plots for tongue/Pneumonia/s__Simonsiella_muelleri_mgs_3058/scatter.pdf]

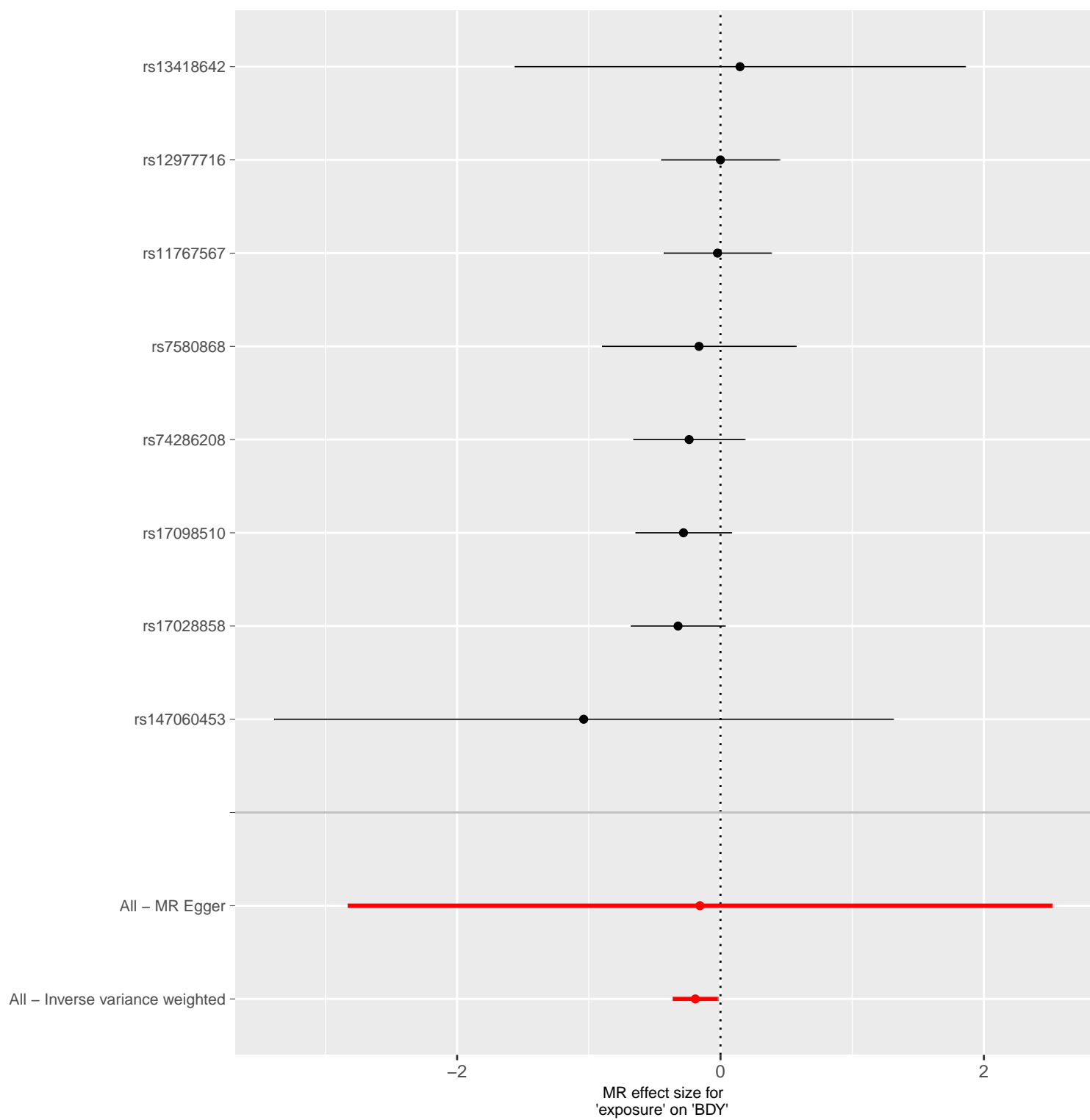

Supplement: Supplementary file 1 [file Data_Sheet_1.zip › Supplementary Materials/MR plots for tongue/Pneumonia/s__Streptococcus_anginosus_mgs_295/forest.pdf]

# MR Method

- Inverse variance weighted
- MR Egger

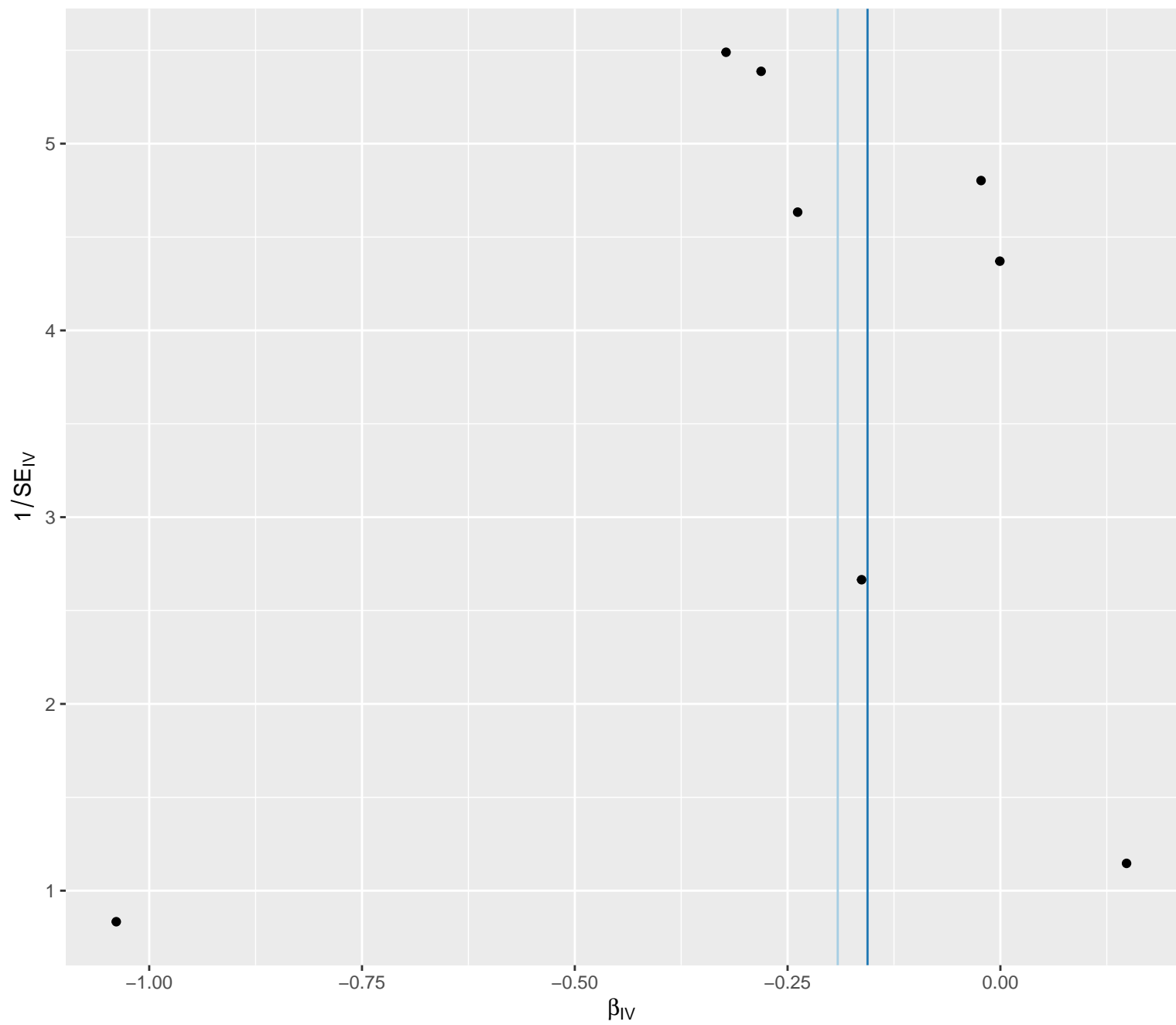

Supplement: Supplementary file 1 [file Data_Sheet_1.zip › Supplementary Materials/MR plots for tongue/Pneumonia/s__Streptococcus_anginosus_mgs_295/funnel.pdf]

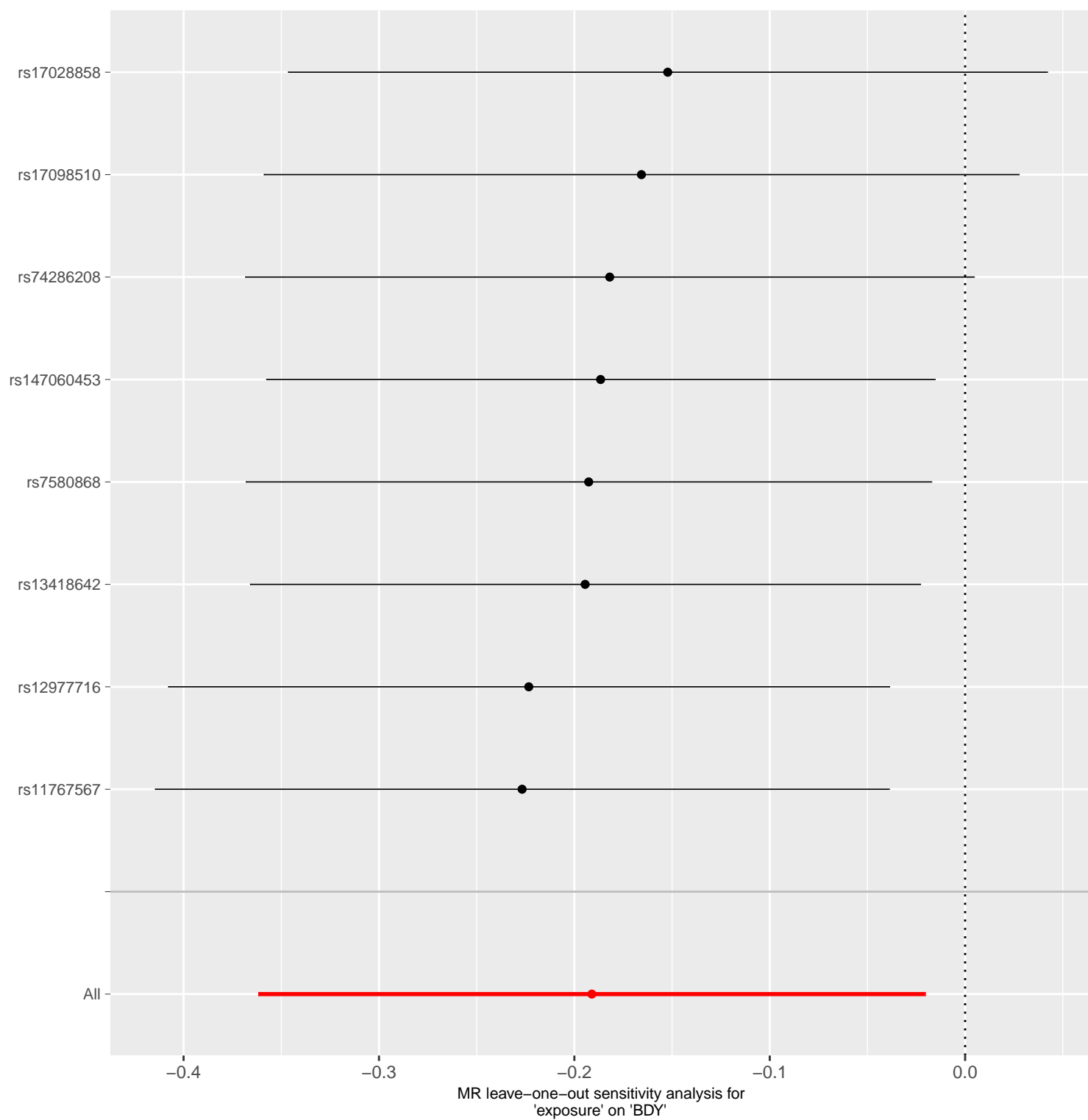

Supplement: Supplementary file 1 [file Data_Sheet_1.zip › Supplementary Materials/MR plots for tongue/Pneumonia/s__Streptococcus_anginosus_mgs_295/leave_one_out.pdf]

# MR Test

- Inverse variance weighted
- MR Egger
- Weighted median

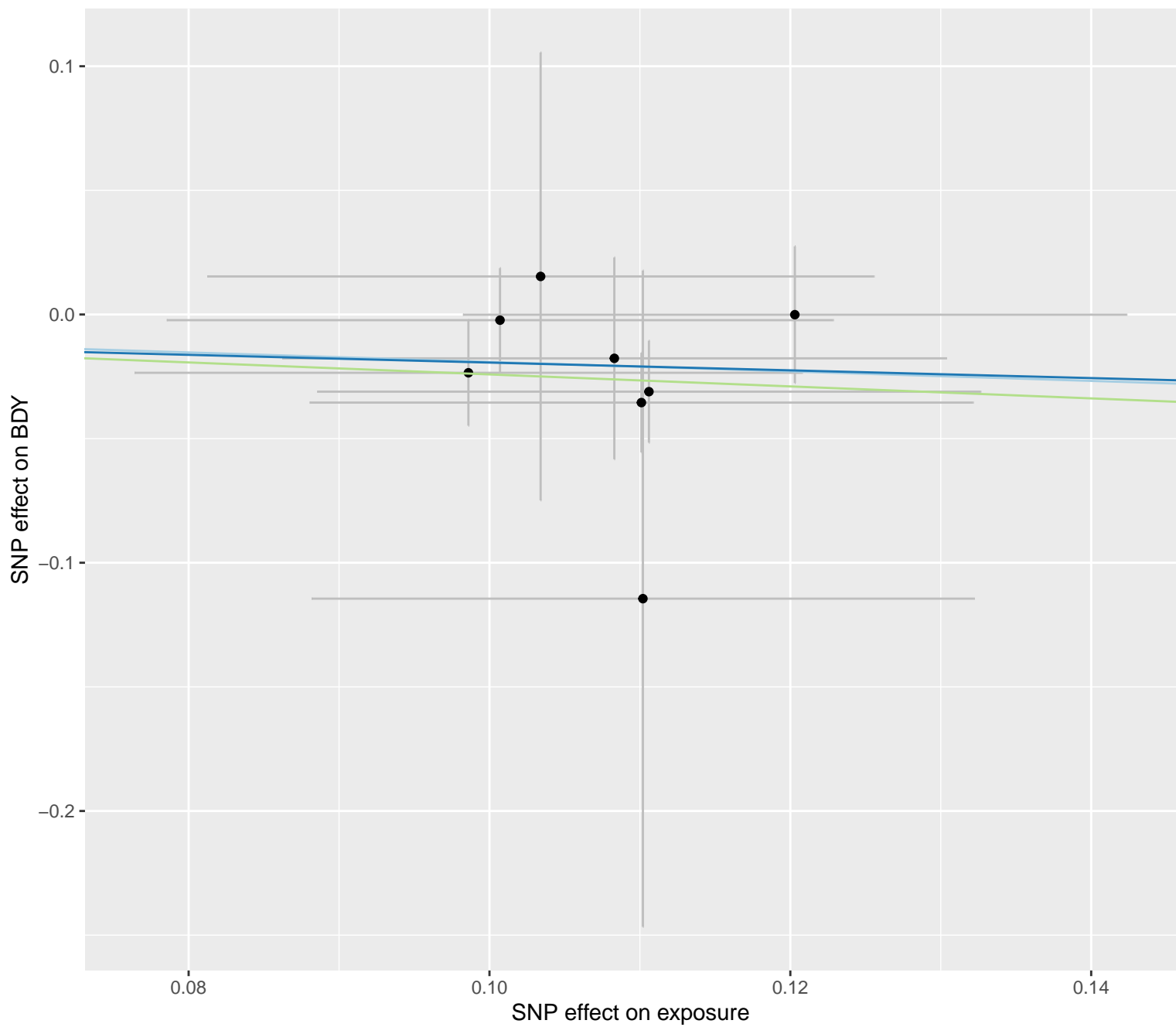

Supplement: Supplementary file 1 [file Data_Sheet_1.zip › Supplementary Materials/MR plots for tongue/Pneumonia/s__Streptococcus_anginosus_mgs_295/scatter.pdf]

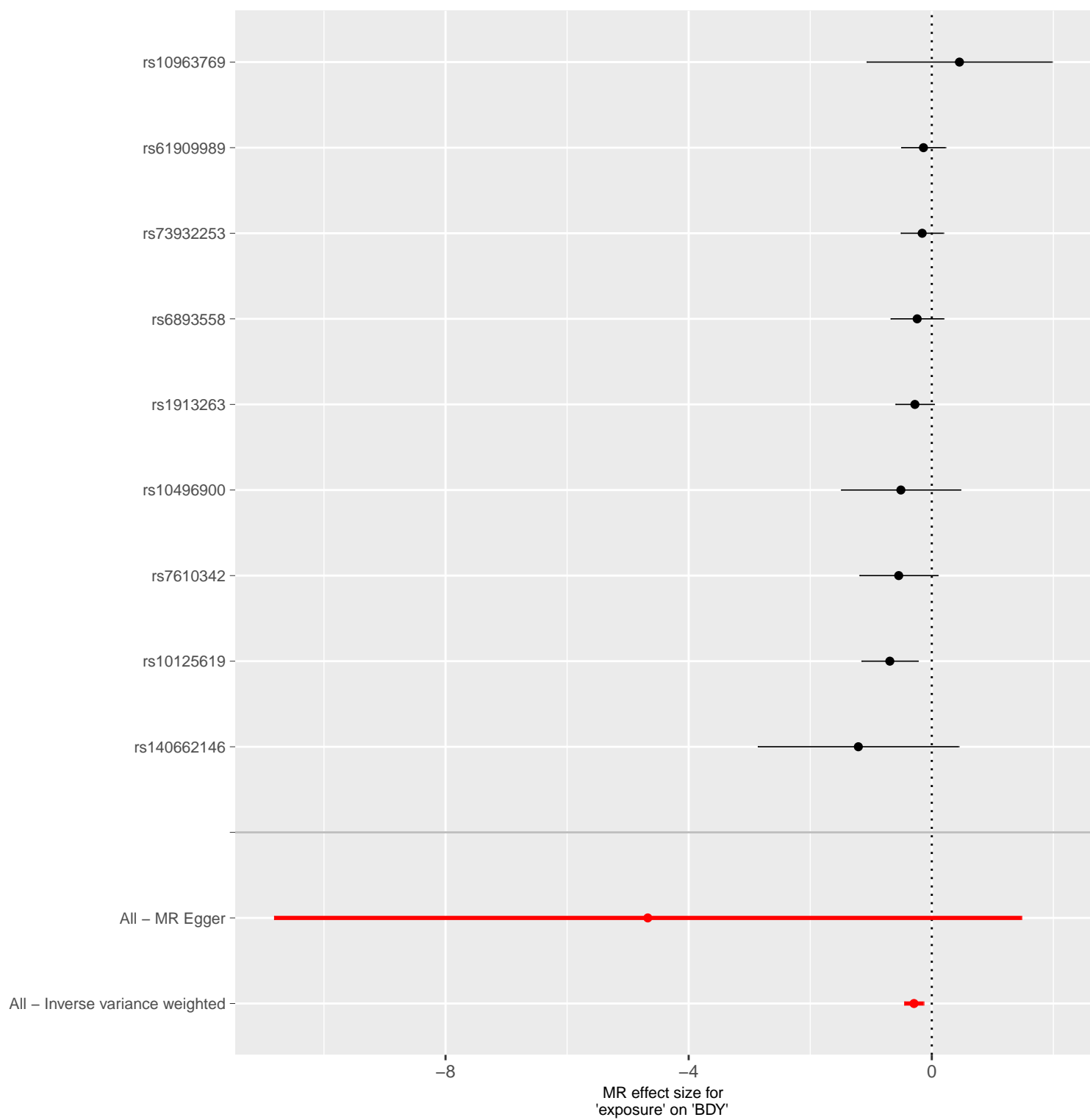

Supplement: Supplementary file 1 [file Data_Sheet_1.zip › Supplementary Materials/MR plots for tongue/Pneumonia/s__Streptococcus_parasanguinis_B_mgs_3506/forest.pdf]

# MR Method

- Inverse variance weighted
- MR Egger

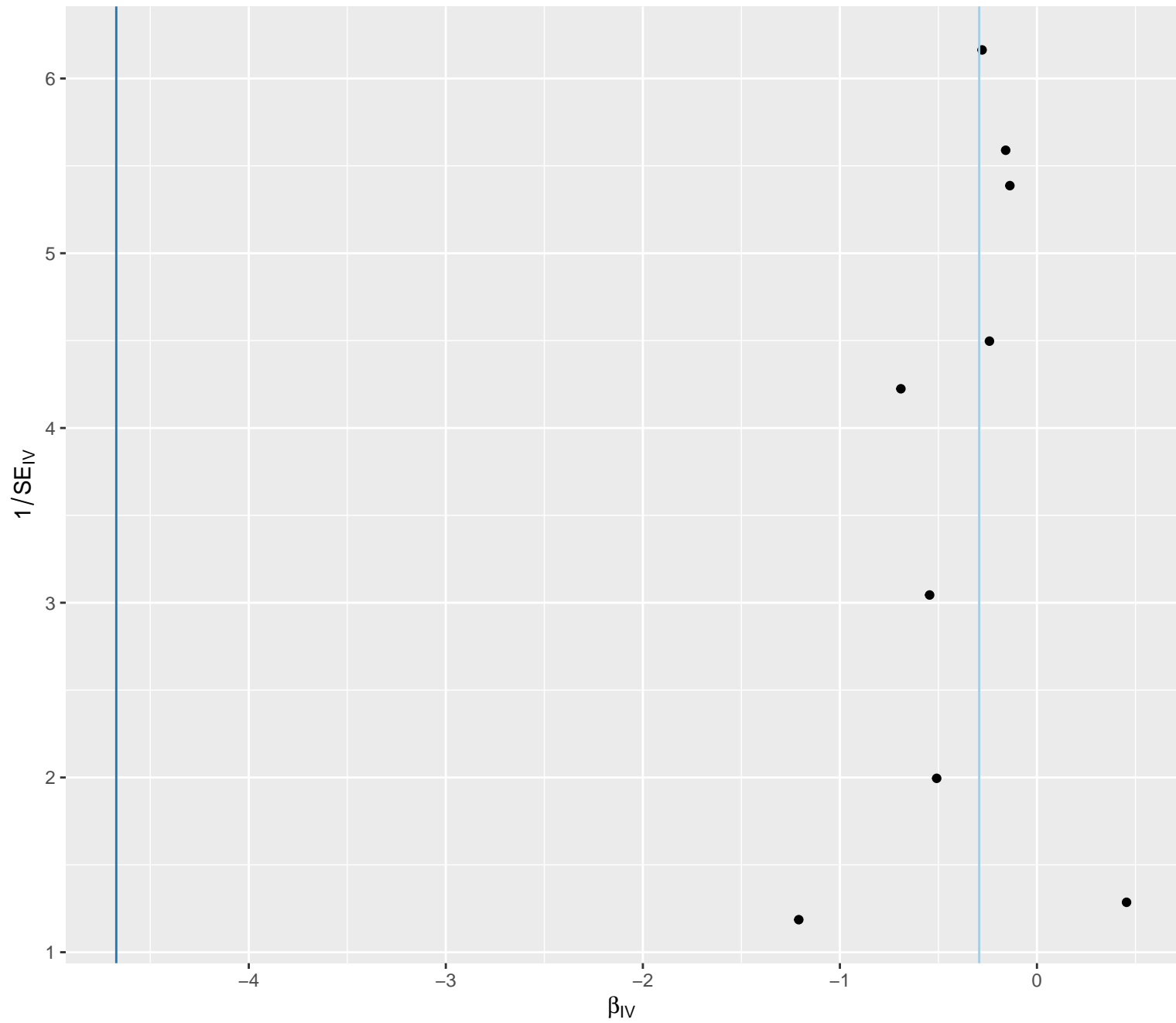

Supplement: Supplementary file 1 [file Data_Sheet_1.zip › Supplementary Materials/MR plots for tongue/Pneumonia/s__Streptococcus_parasanguinis_B_mgs_3506/funnel.pdf]

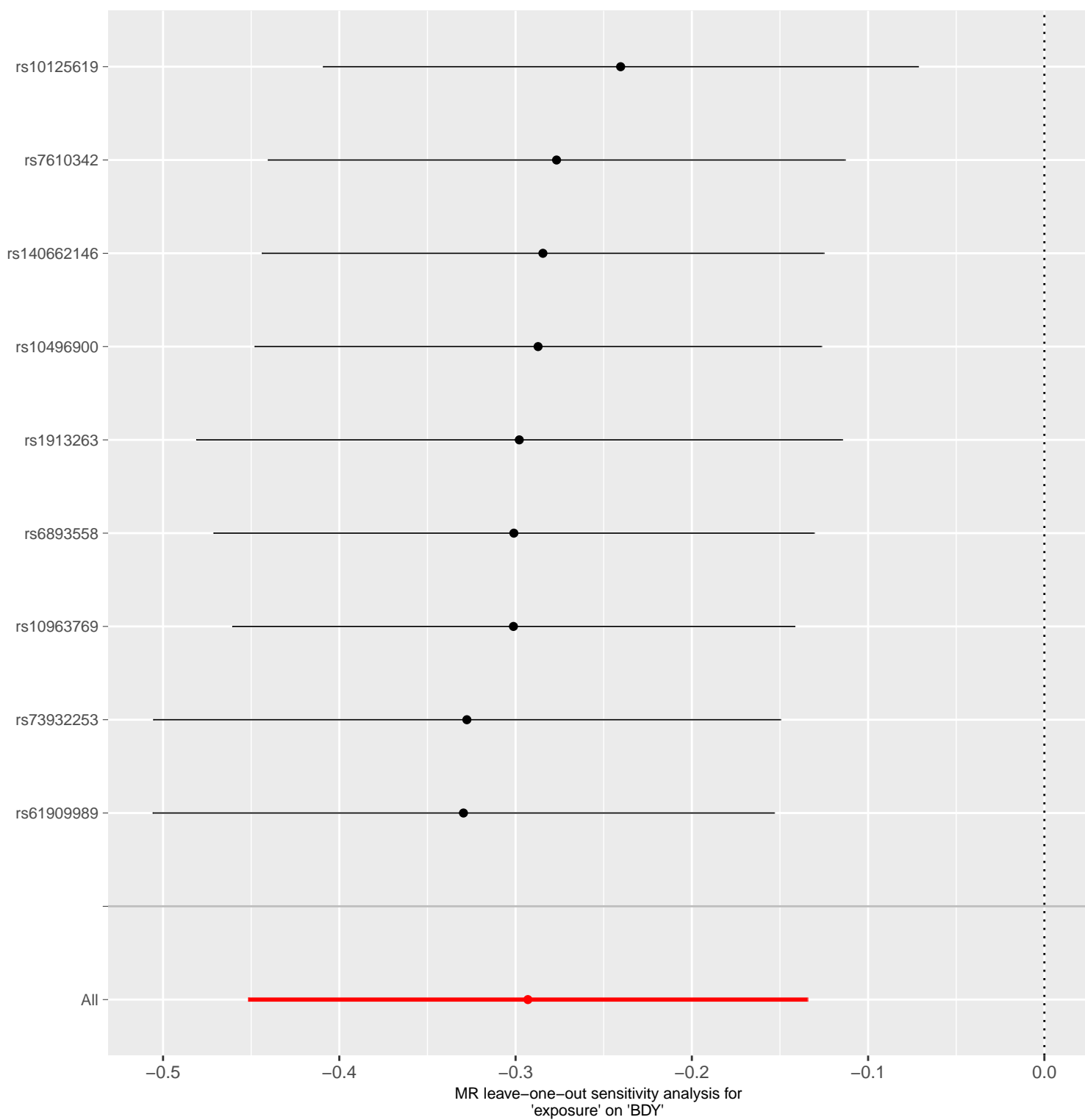

Supplement: Supplementary file 1 [file Data_Sheet_1.zip › Supplementary Materials/MR plots for tongue/Pneumonia/s__Streptococcus_parasanguinis_B_mgs_3506/leave_one_out.pdf]

# MR Test

- Inverse variance weighted
- MR Egger
- Weighted median

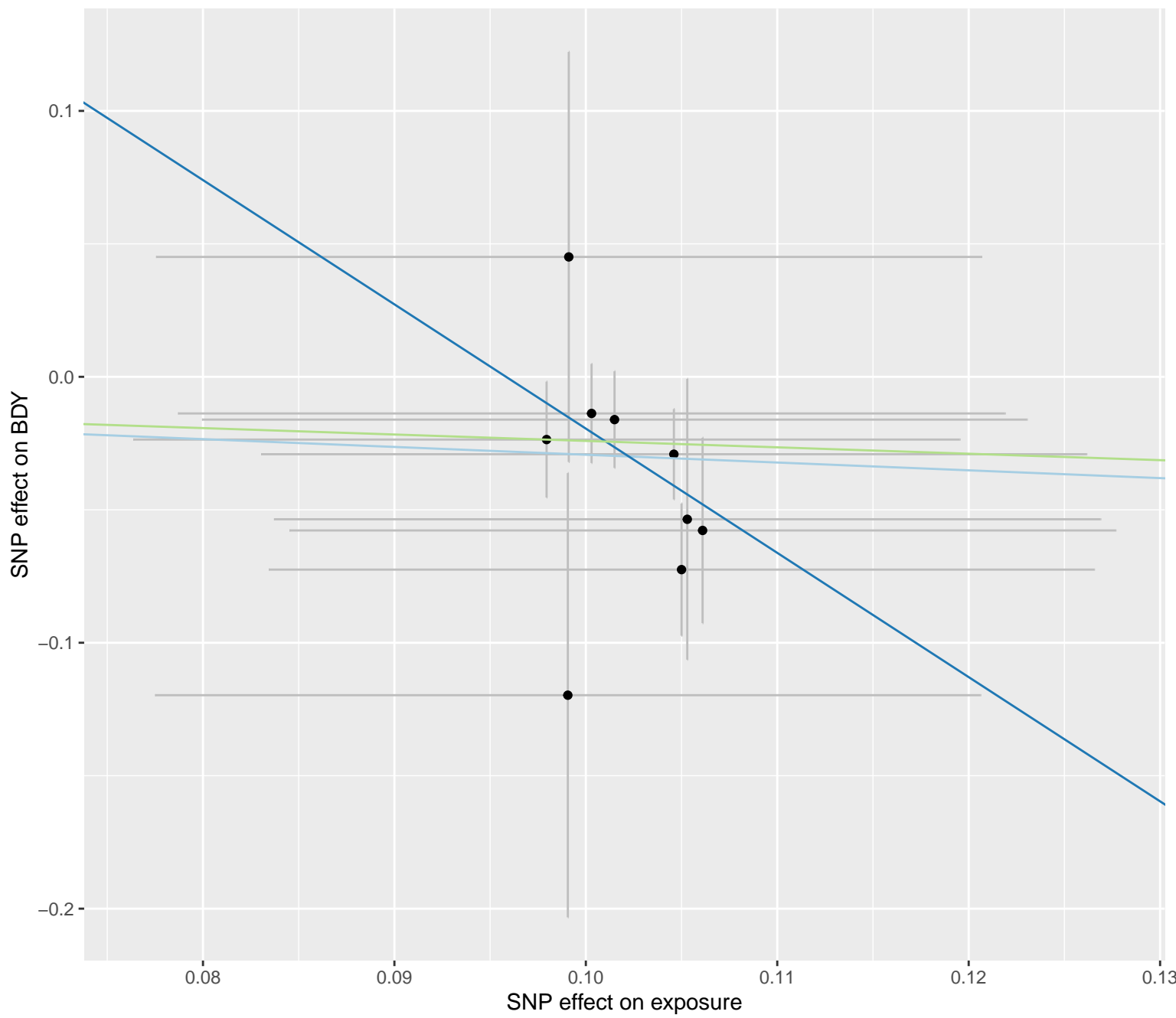

Supplement: Supplementary file 1 [file Data_Sheet_1.zip › Supplementary Materials/MR plots for tongue/Pneumonia/s__Streptococcus_parasanguinis_B_mgs_3506/scatter.pdf]

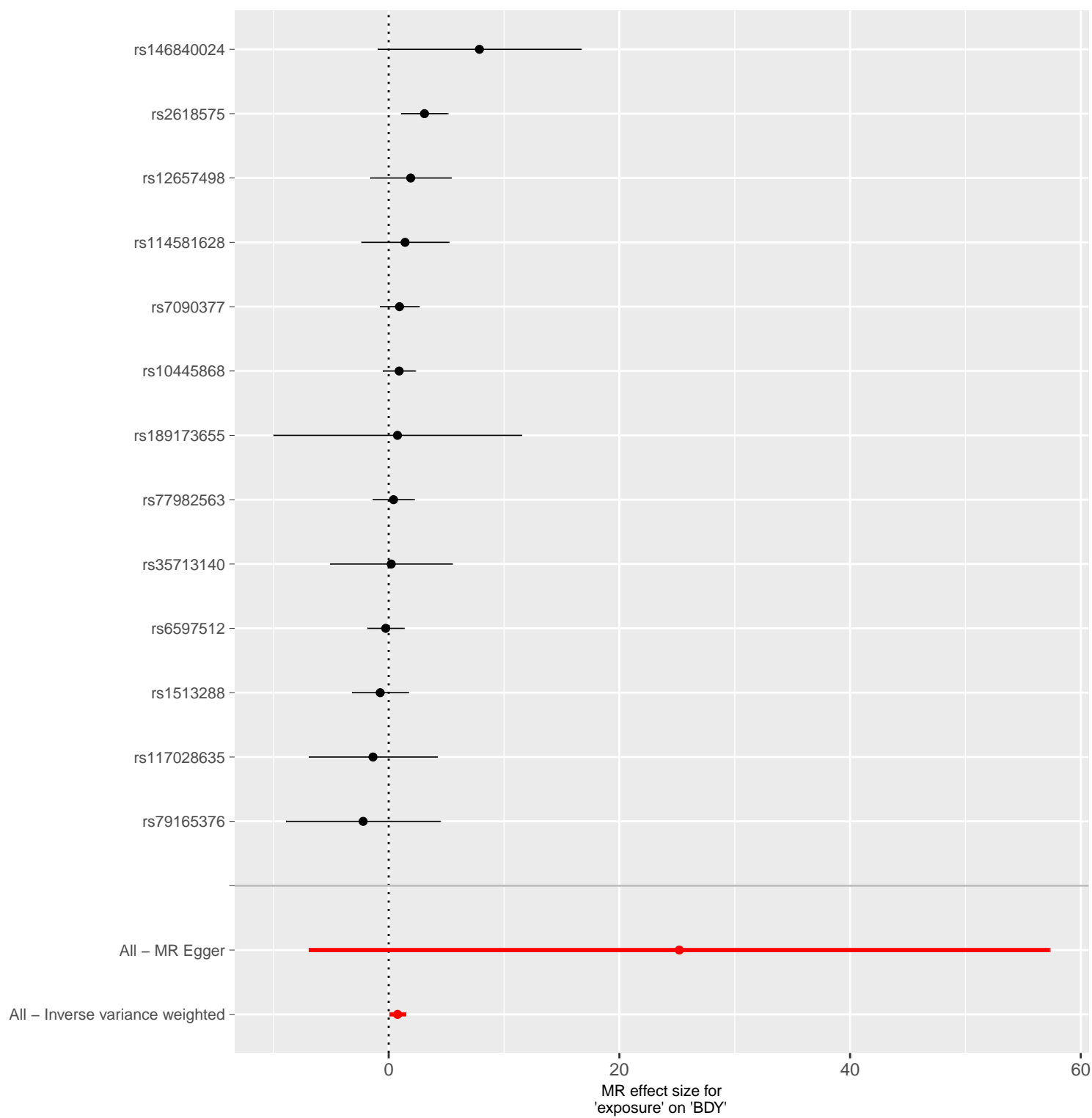

Supplement: Supplementary file 1 [file Data_Sheet_1.zip › Supplementary Materials/MR plots for tongue/Tonsillitis/s__Campylobacter_A_rectus_mgs_1402/forest.pdf]

# MR Method

- Inverse variance weighted
- MR Egger

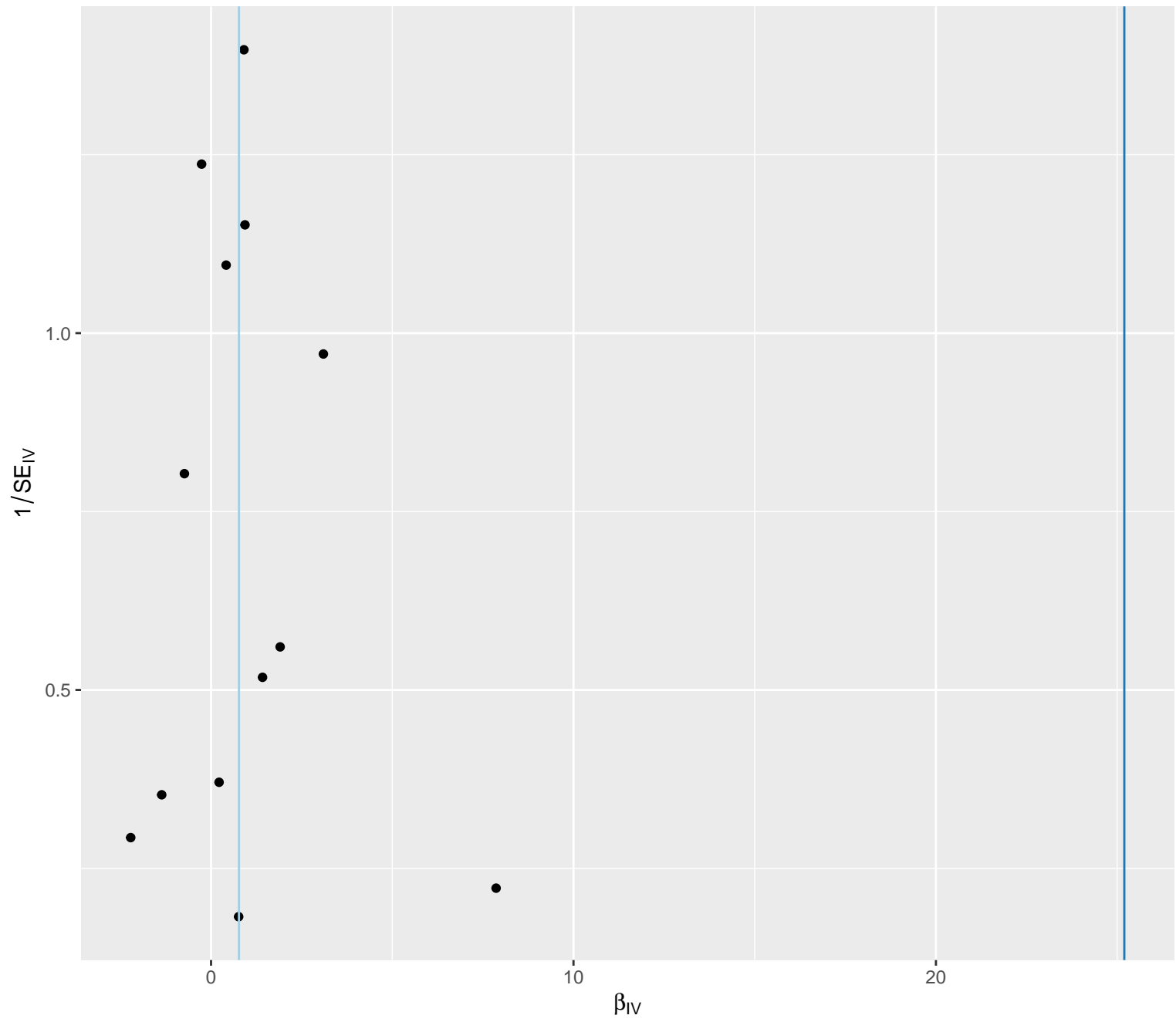

Supplement: Supplementary file 1 [file Data_Sheet_1.zip › Supplementary Materials/MR plots for tongue/Tonsillitis/s__Campylobacter_A_rectus_mgs_1402/funnel.pdf]

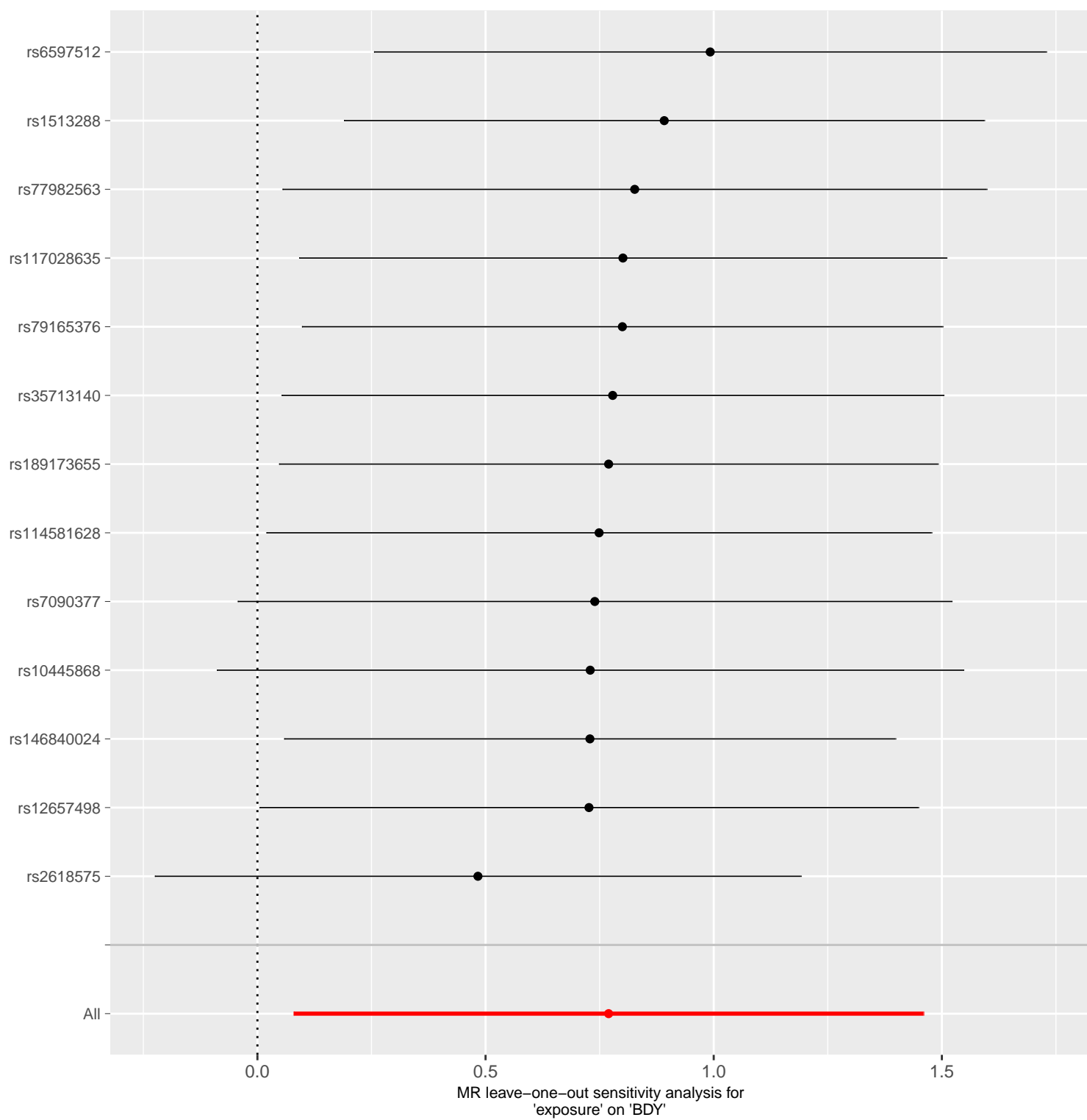

Supplement: Supplementary file 1 [file Data_Sheet_1.zip › Supplementary Materials/MR plots for tongue/Tonsillitis/s__Campylobacter_A_rectus_mgs_1402/leave_one_out.pdf]

# MR Test

- Inverse variance weighted
- MR Egger
- Weighted median

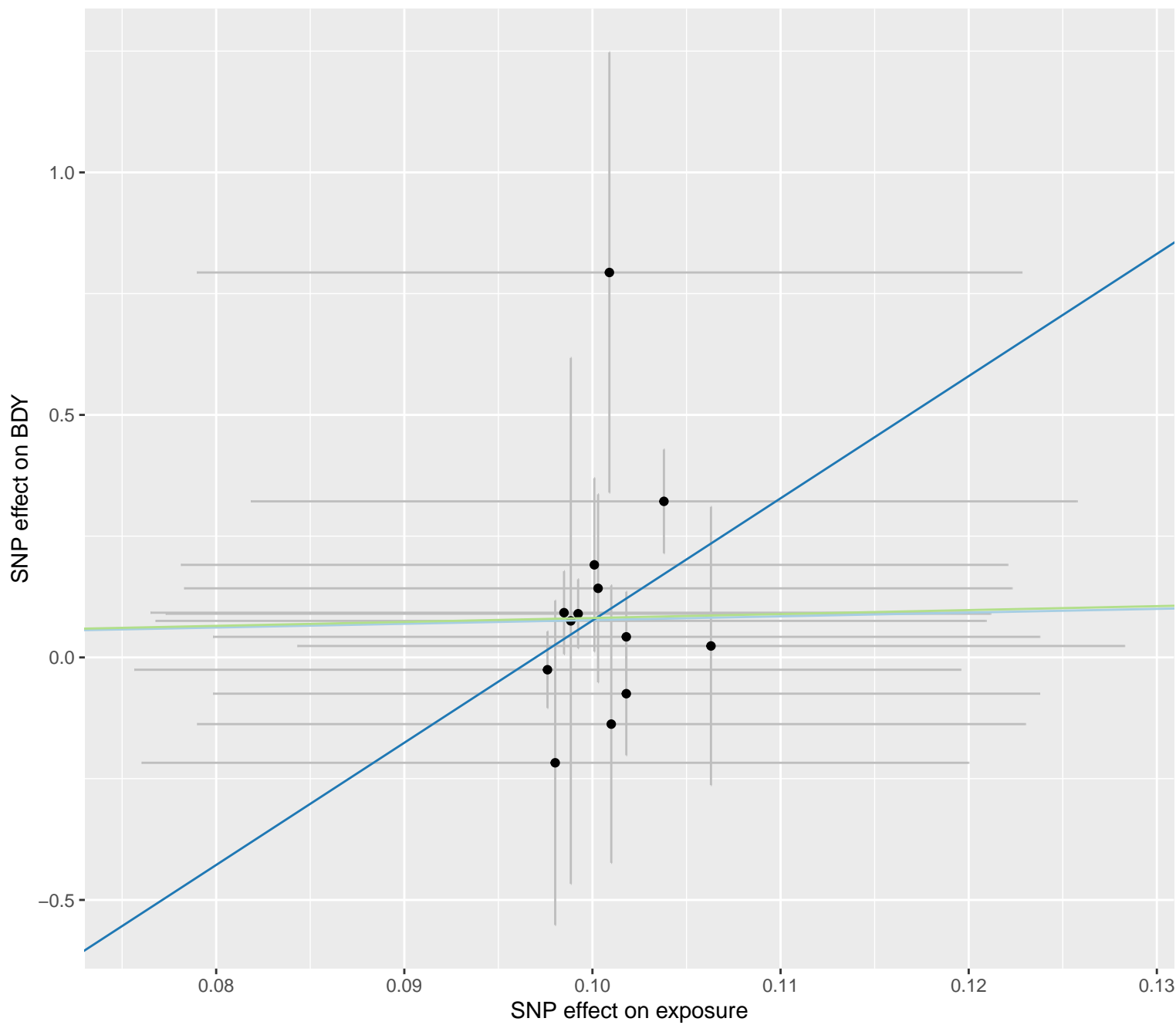

Supplement: Supplementary file 1 [file Data_Sheet_1.zip › Supplementary Materials/MR plots for tongue/Tonsillitis/s__Campylobacter_A_rectus_mgs_1402/scatter.pdf]

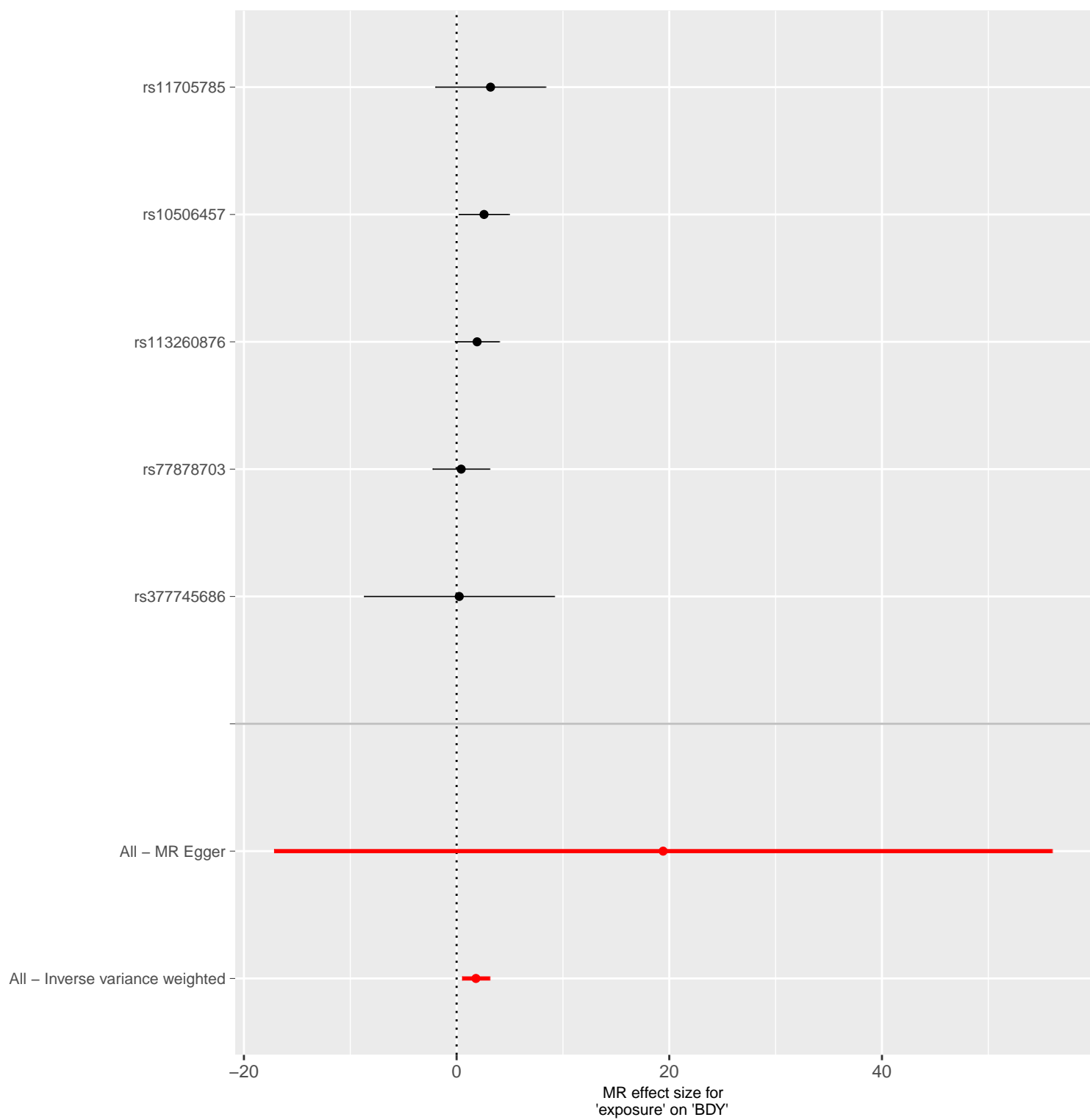

Supplement: Supplementary file 1 [file Data_Sheet_1.zip › Supplementary Materials/MR plots for tongue/Tonsillitis/s__Neisseria_lactamica_mgs_343/forest.pdf]

# MR Method

- Inverse variance weighted
- MR Egger

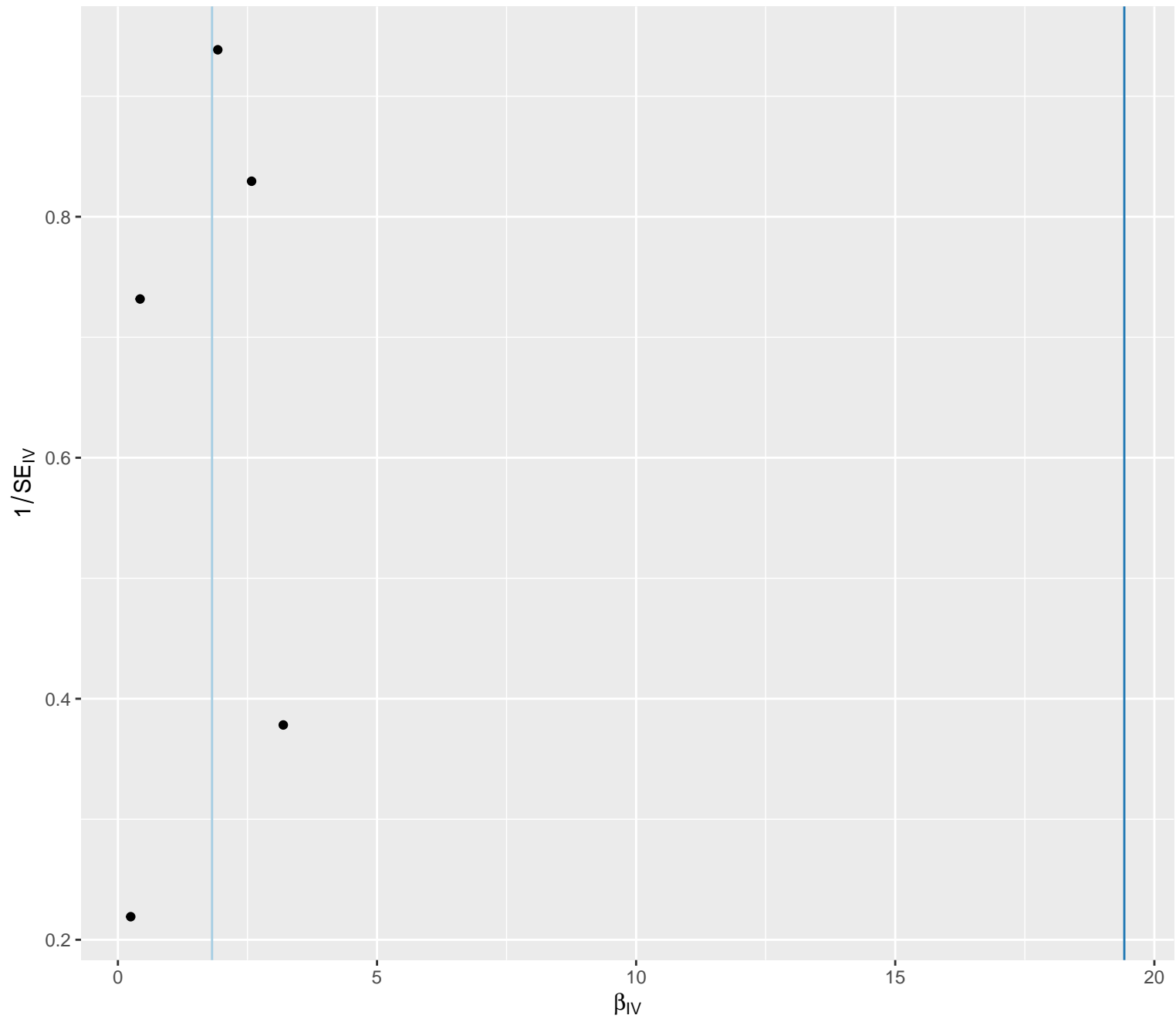

Supplement: Supplementary file 1 [file Data_Sheet_1.zip › Supplementary Materials/MR plots for tongue/Tonsillitis/s__Neisseria_lactamica_mgs_343/funnel.pdf]

rs77878703

rs377745686

rs113260876

rs11705785

rs10506457

All

0

1

2

3

MR leave-one-out sensitivity analysis for  
'exposure' on 'BDY'

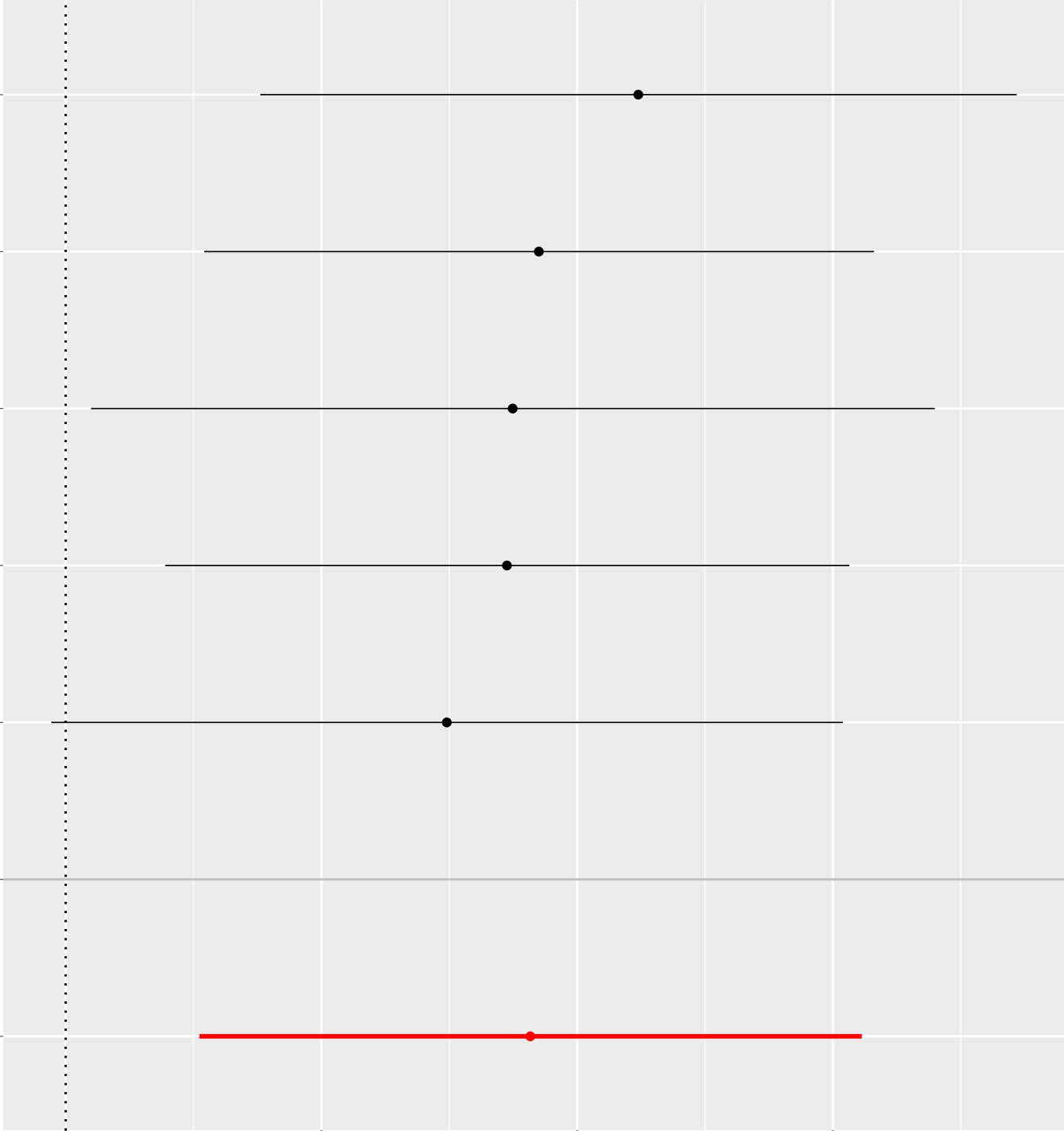

Supplement: Supplementary file 1 [file Data_Sheet_1.zip › Supplementary Materials/MR plots for tongue/Tonsillitis/s__Neisseria_lactamica_mgs_343/leave_one_out.pdf]

# MR Test

- Inverse variance weighted
- MR Egger
- Weighted median

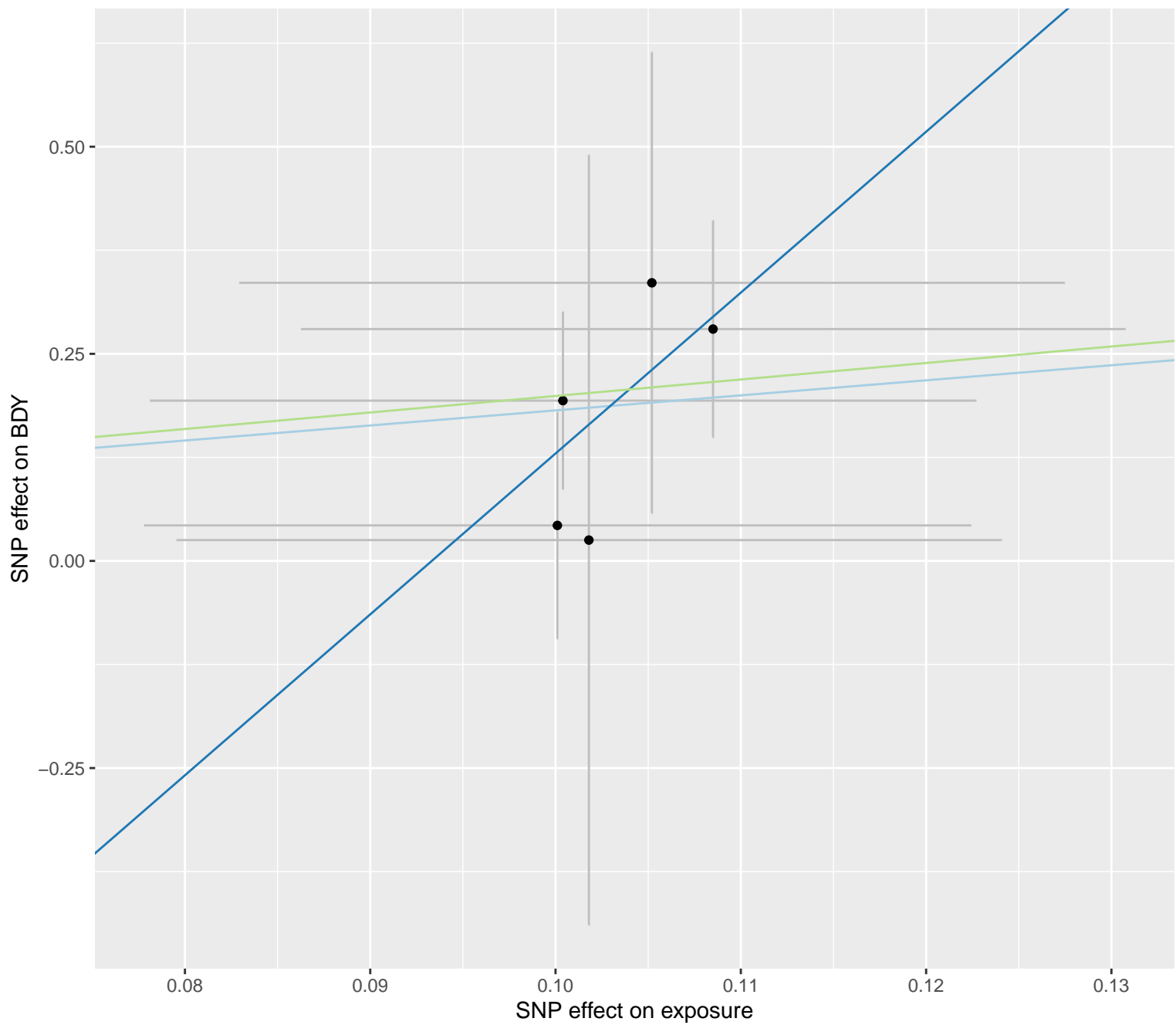

Supplement: Supplementary file 1 [file Data_Sheet_1.zip › Supplementary Materials/MR plots for tongue/Tonsillitis/s__Neisseria_lactamica_mgs_343/scatter.pdf]

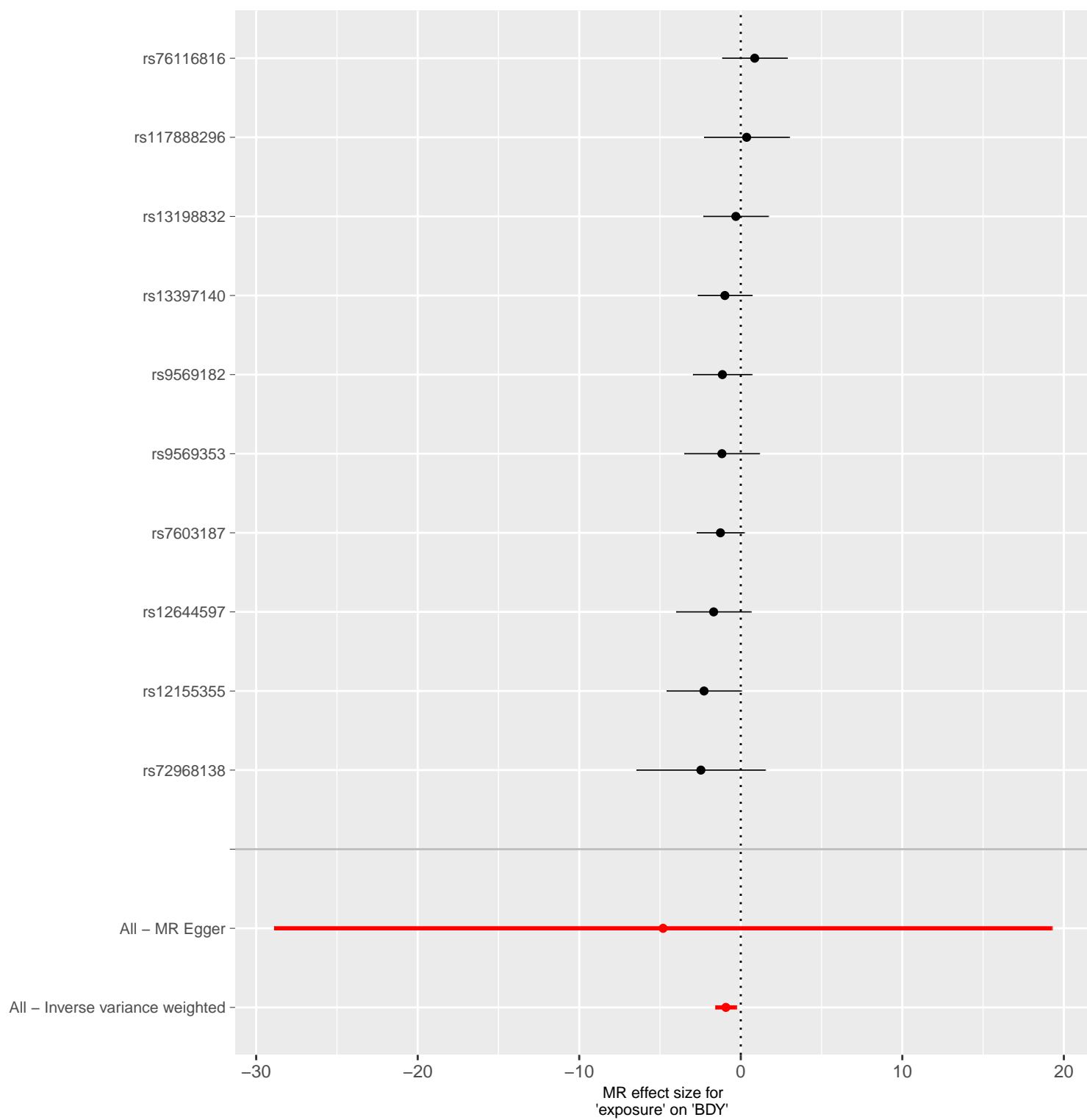

Supplement: Supplementary file 1 [file Data_Sheet_1.zip › Supplementary Materials/MR plots for tongue/Tonsillitis/s__Solobacterium_moorei_mgs_709/forest.pdf]

# MR Method

- Inverse variance weighted
- MR Egger

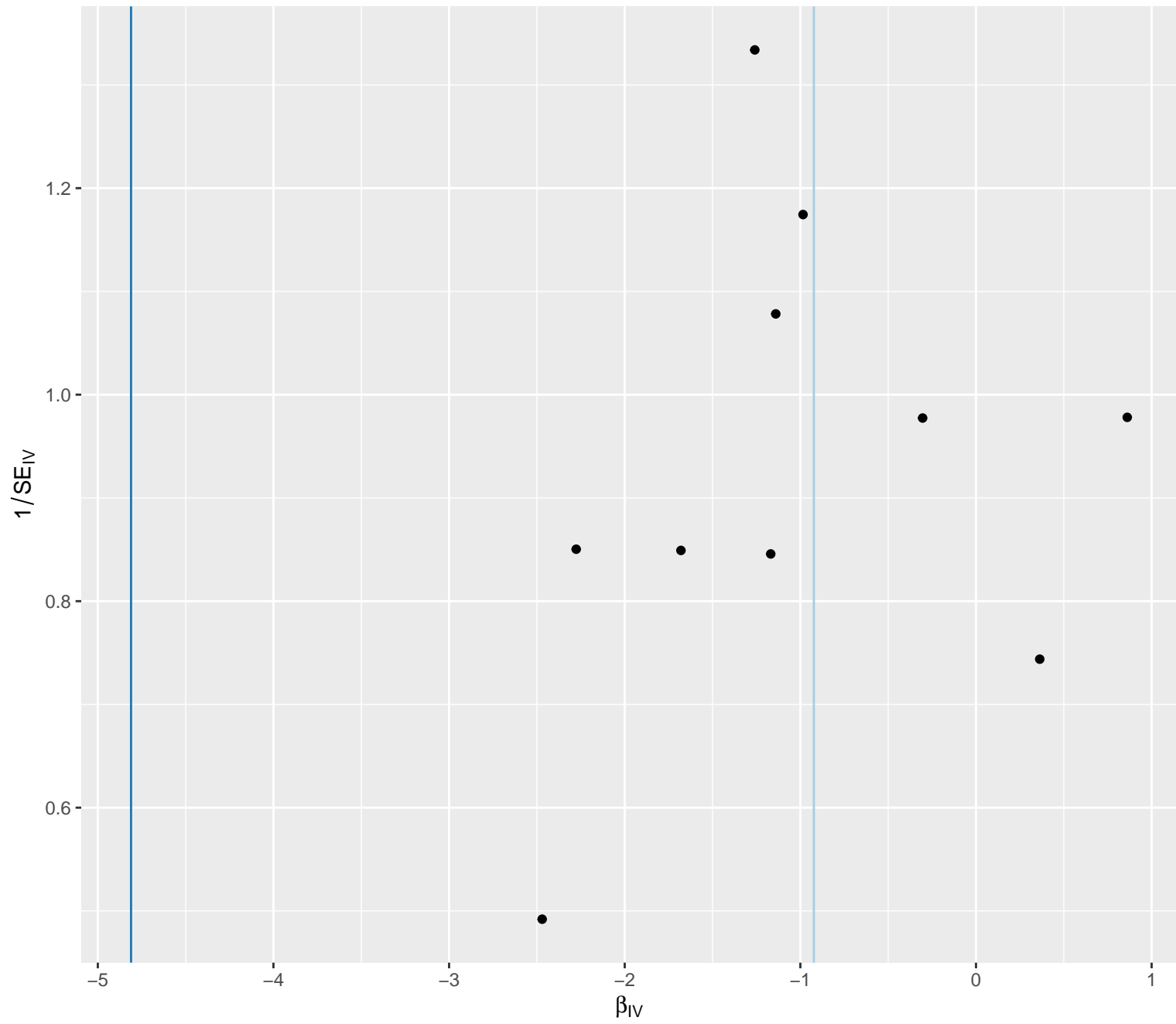

Supplement: Supplementary file 1 [file Data_Sheet_1.zip › Supplementary Materials/MR plots for tongue/Tonsillitis/s__Solobacterium_moorei_mgs_709/funnel.pdf]

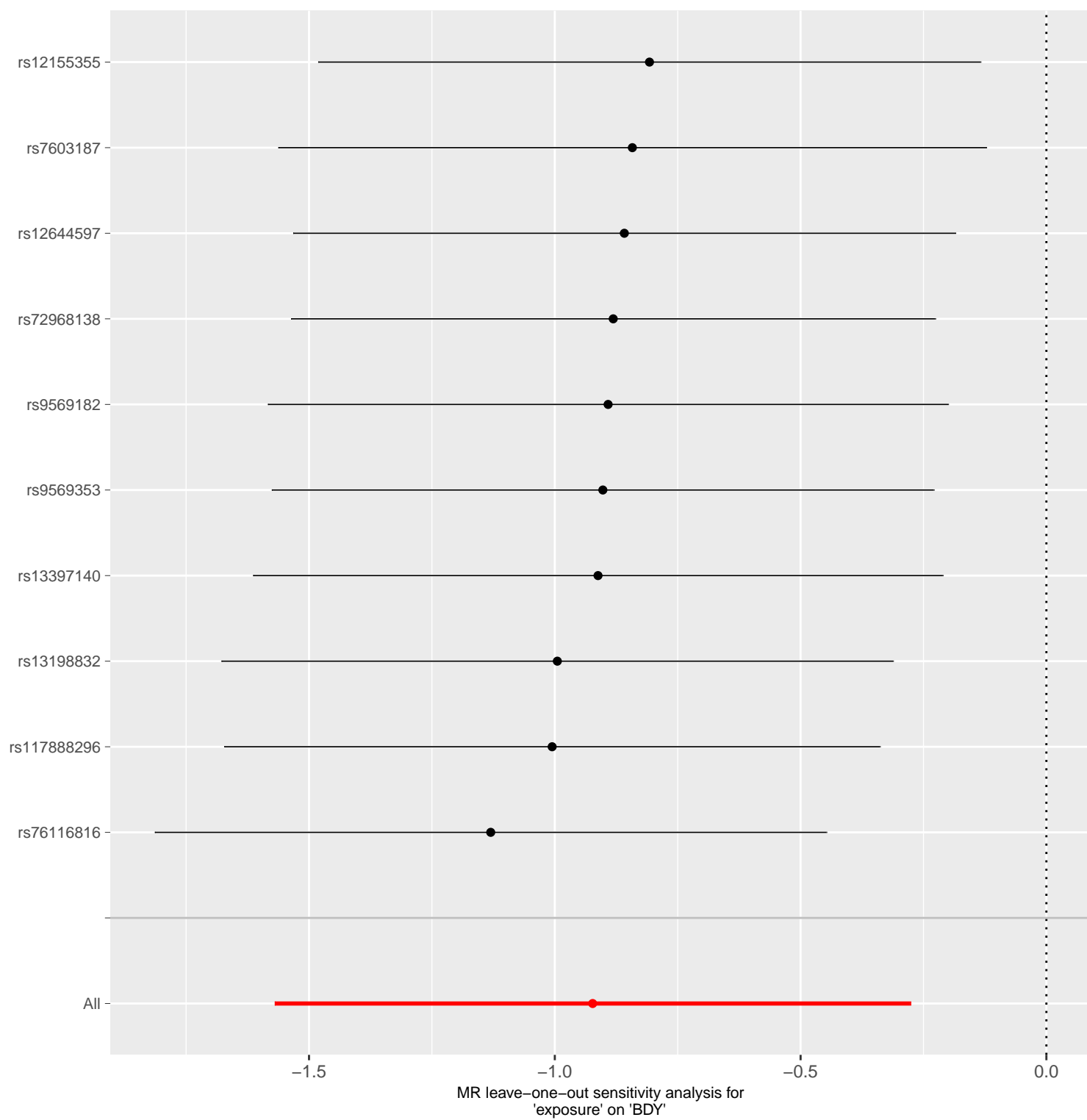

Supplement: Supplementary file 1 [file Data_Sheet_1.zip › Supplementary Materials/MR plots for tongue/Tonsillitis/s__Solobacterium_moorei_mgs_709/leave_one_out.pdf]

# MR Test

- Inverse variance weighted
- MR Egger
- Weighted median

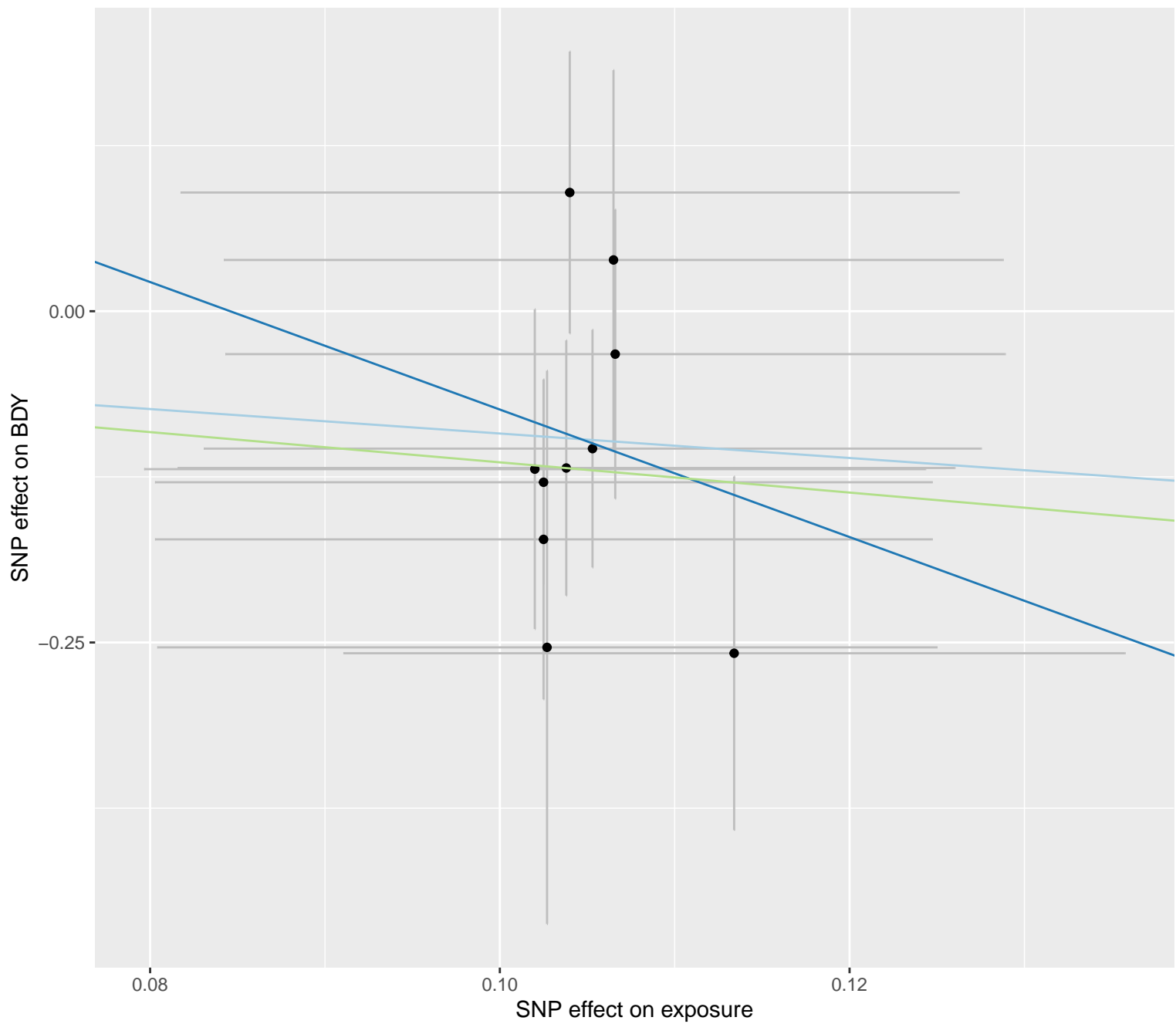

Supplement: Supplementary file 1 [file Data_Sheet_1.zip › Supplementary Materials/MR plots for tongue/Tonsillitis/s__Solobacterium_moorei_mgs_709/scatter.pdf]

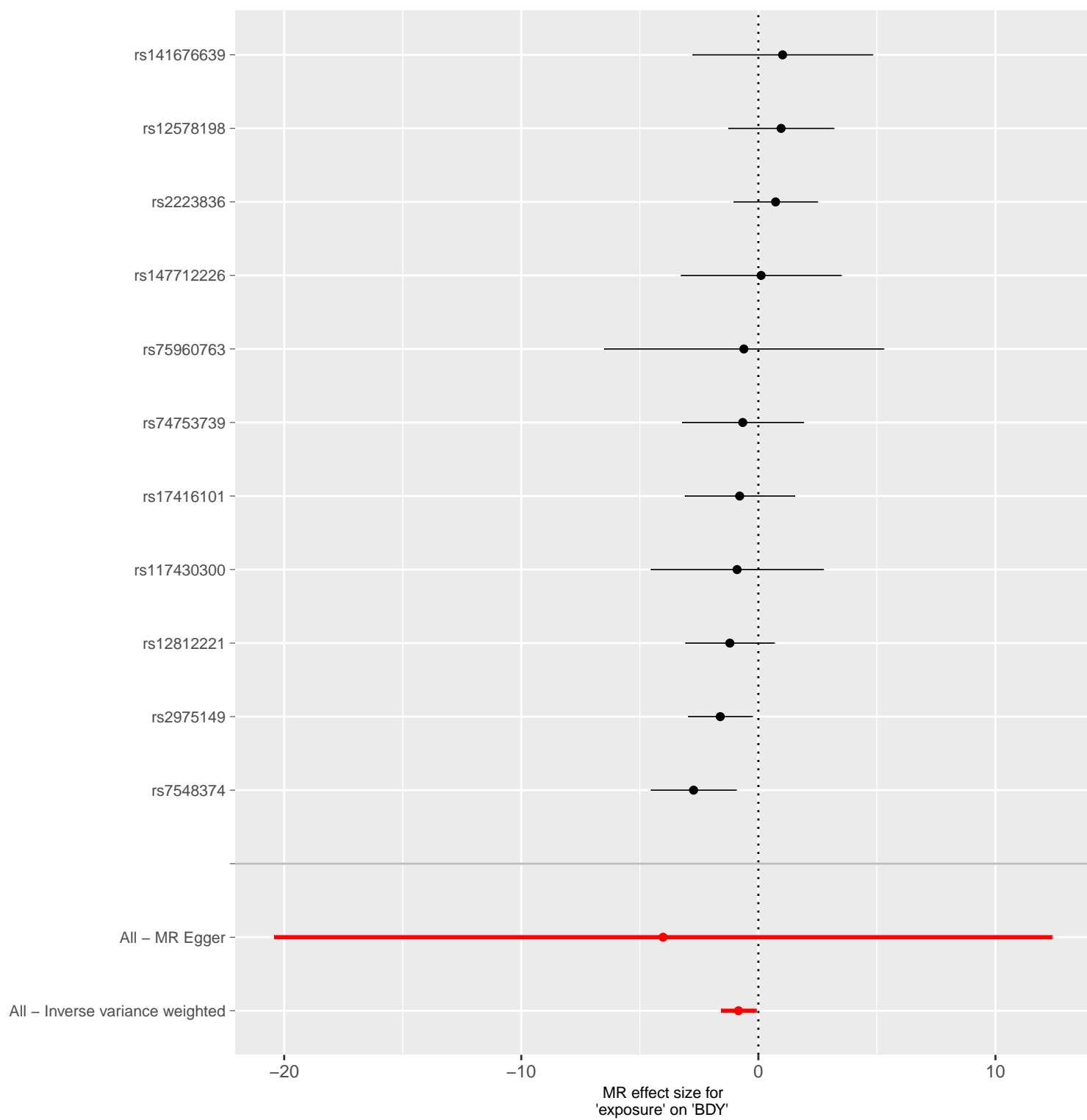

Supplement: Supplementary file 1 [file Data_Sheet_1.zip › Supplementary Materials/MR plots for tongue/Tonsillitis/s__Streptococcus_sanguinis_mgs_1844/forest.pdf]

# MR Method

- Inverse variance weighted
- MR Egger

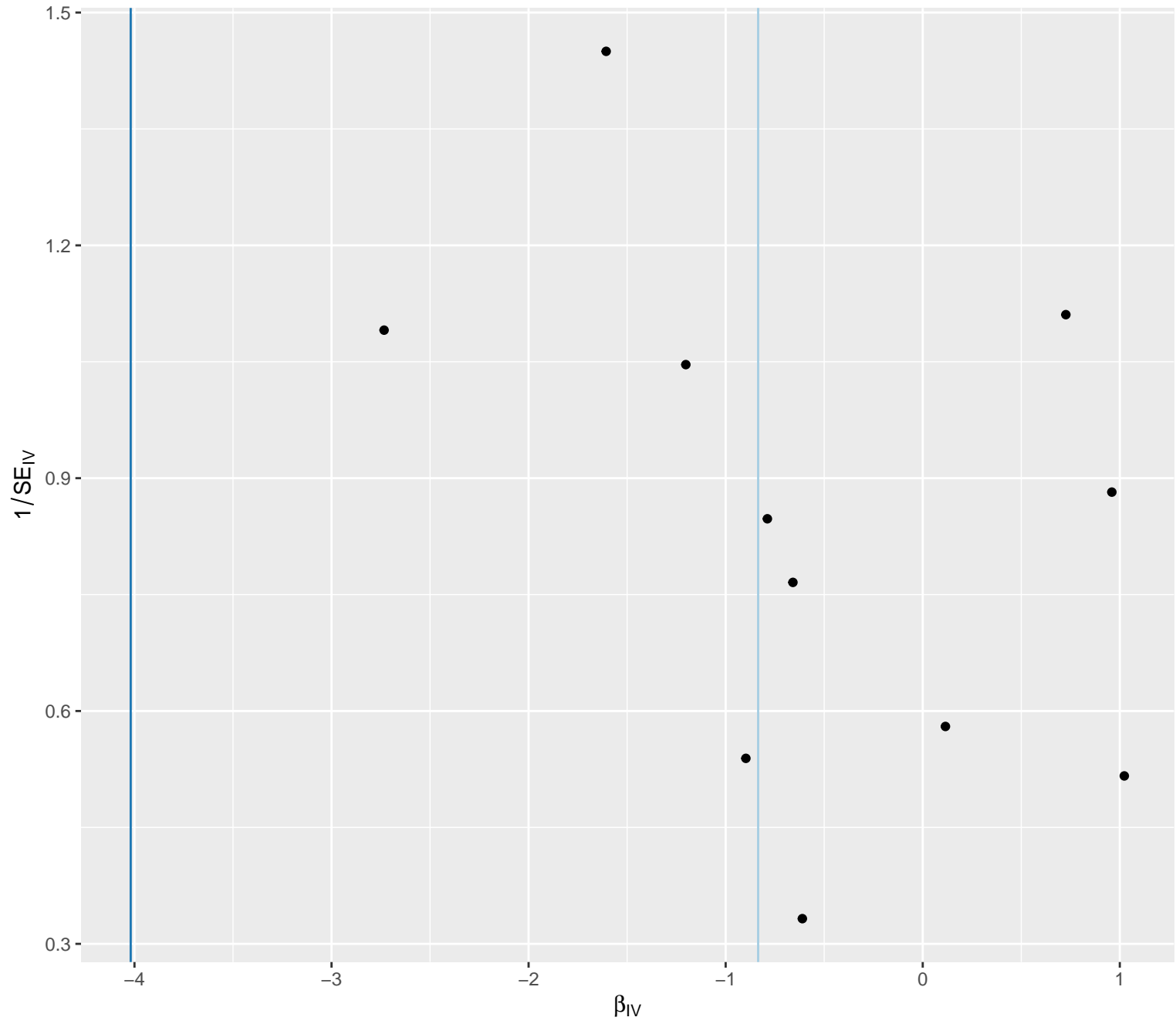

Supplement: Supplementary file 1 [file Data_Sheet_1.zip › Supplementary Materials/MR plots for tongue/Tonsillitis/s__Streptococcus_sanguinis_mgs_1844/funnel.pdf]

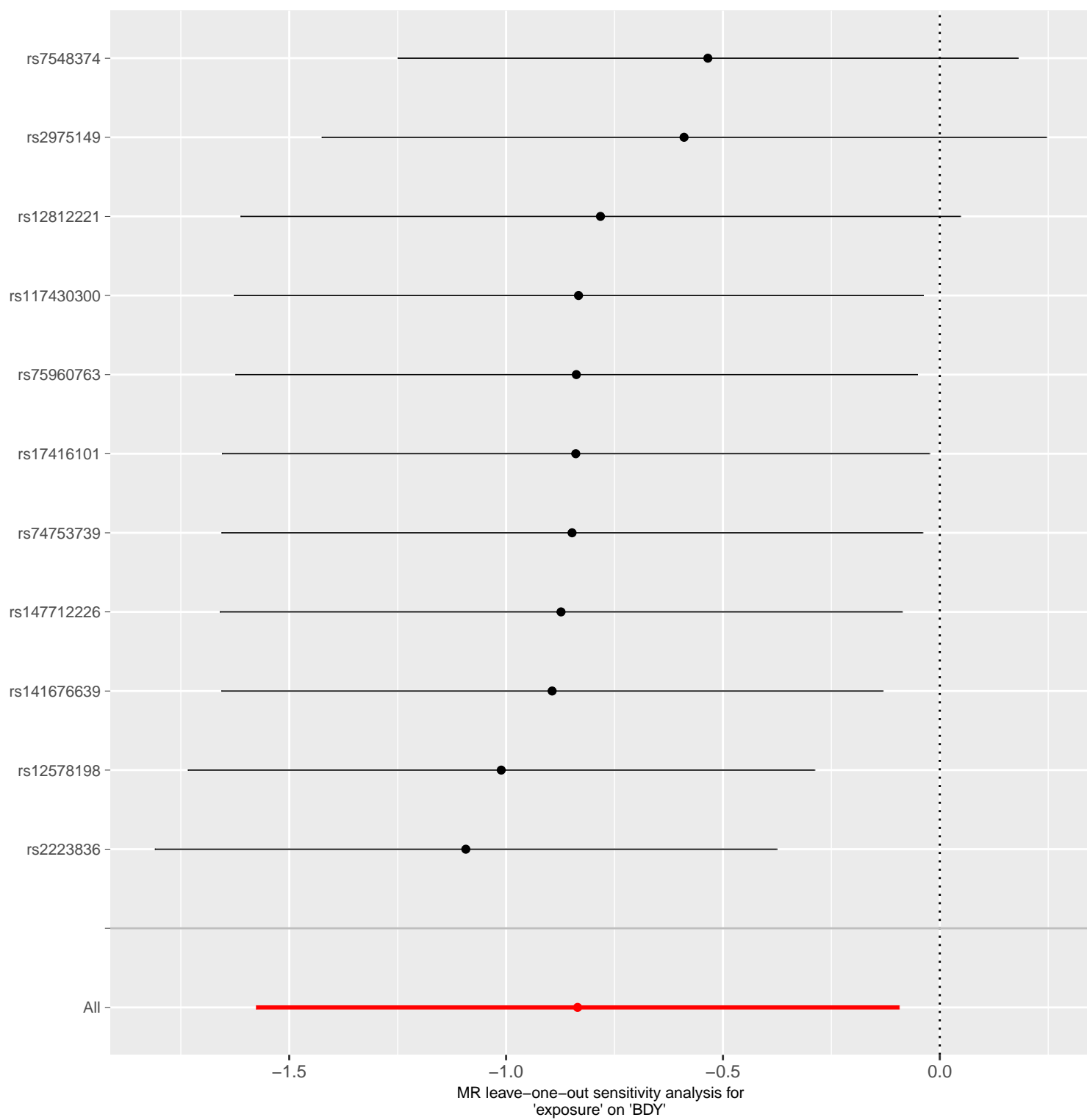

Supplement: Supplementary file 1 [file Data_Sheet_1.zip › Supplementary Materials/MR plots for tongue/Tonsillitis/s__Streptococcus_sanguinis_mgs_1844/leave_one_out.pdf]

# MR Test

- Inverse variance weighted
- MR Egger
- Weighted median

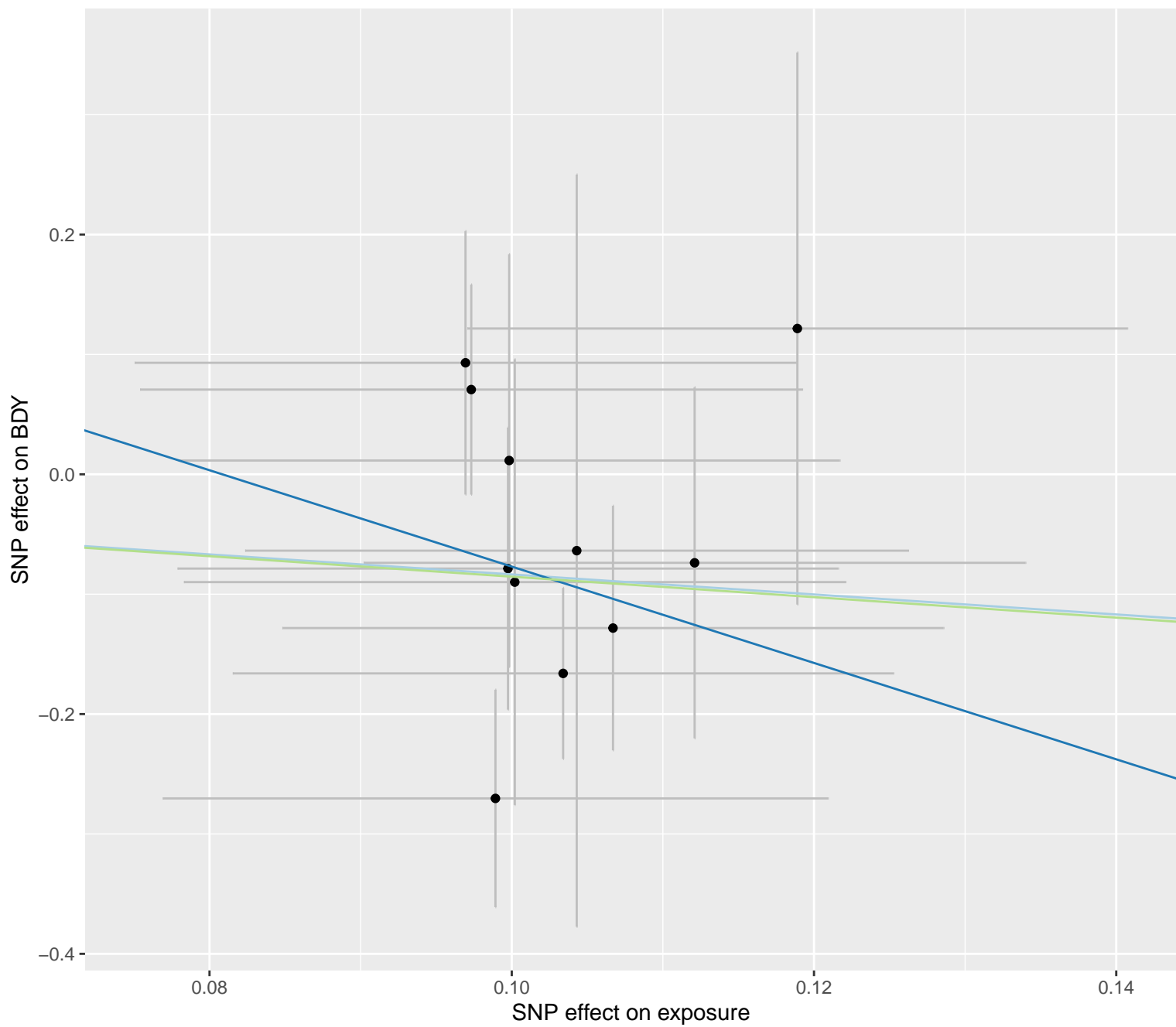

Supplement: Supplementary file 1 [file Data_Sheet_1.zip › Supplementary Materials/MR plots for tongue/Tonsillitis/s__Streptococcus_sanguinis_mgs_1844/scatter.pdf]

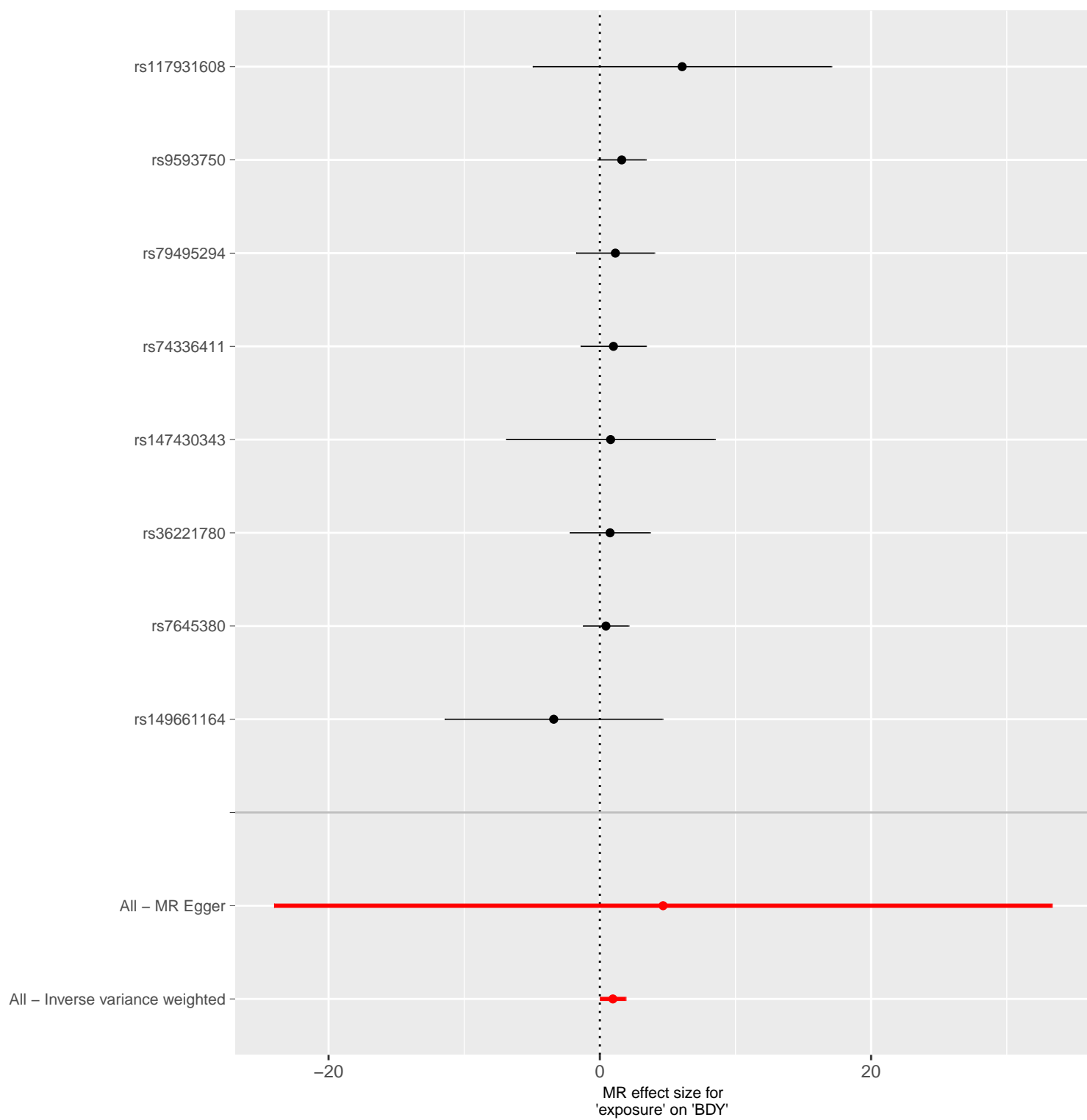

Supplement: Supplementary file 1 [file Data_Sheet_1.zip › Supplementary Materials/MR plots of saliva/Bronchiectasis/s__Aggregatibacter_sp000466335_mgs_2199/forest.pdf]

# MR Method

- Inverse variance weighted
- MR Egger

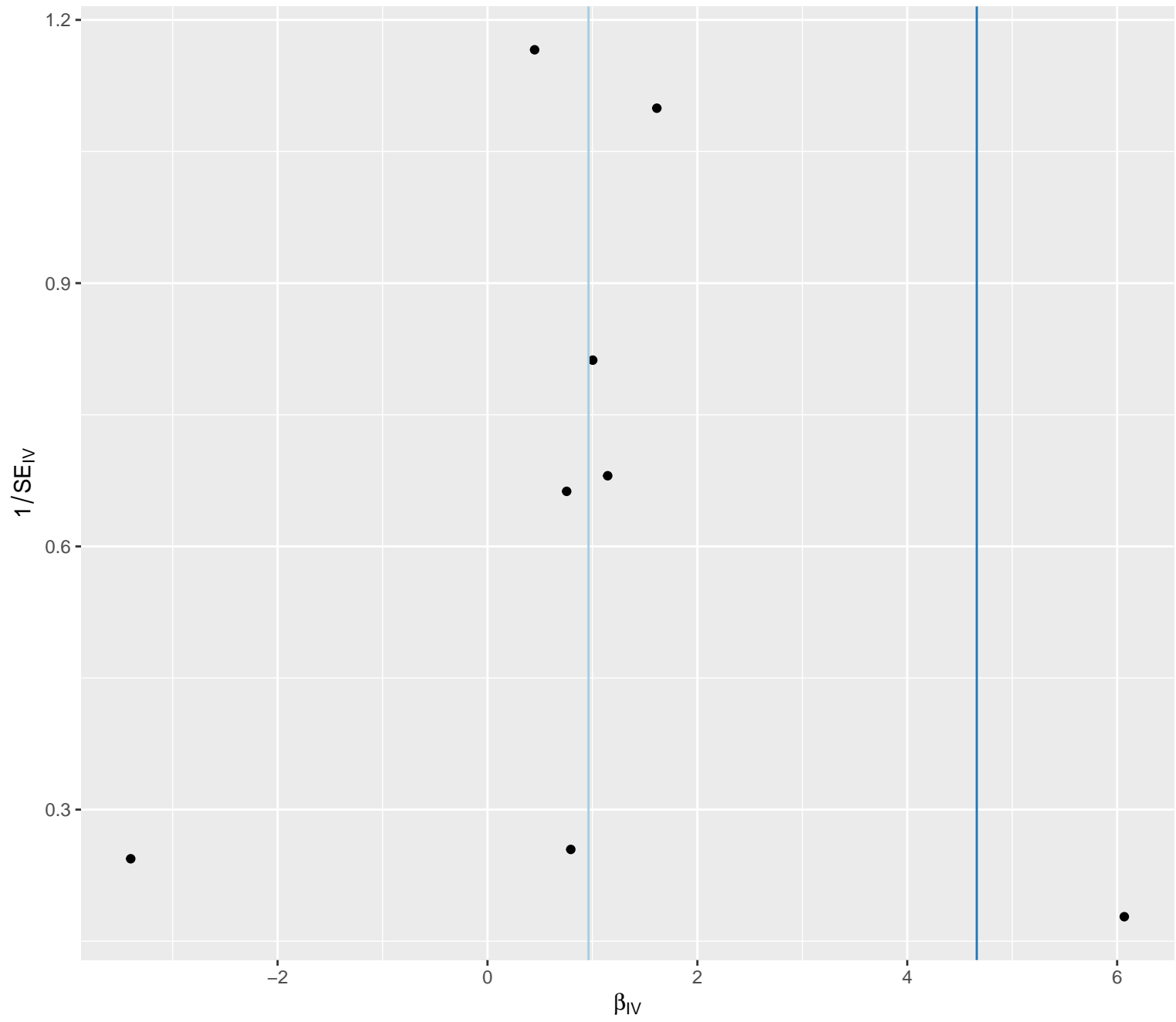

Supplement: Supplementary file 1 [file Data_Sheet_1.zip › Supplementary Materials/MR plots of saliva/Bronchiectasis/s__Aggregatibacter_sp000466335_mgs_2199/funnel.pdf]

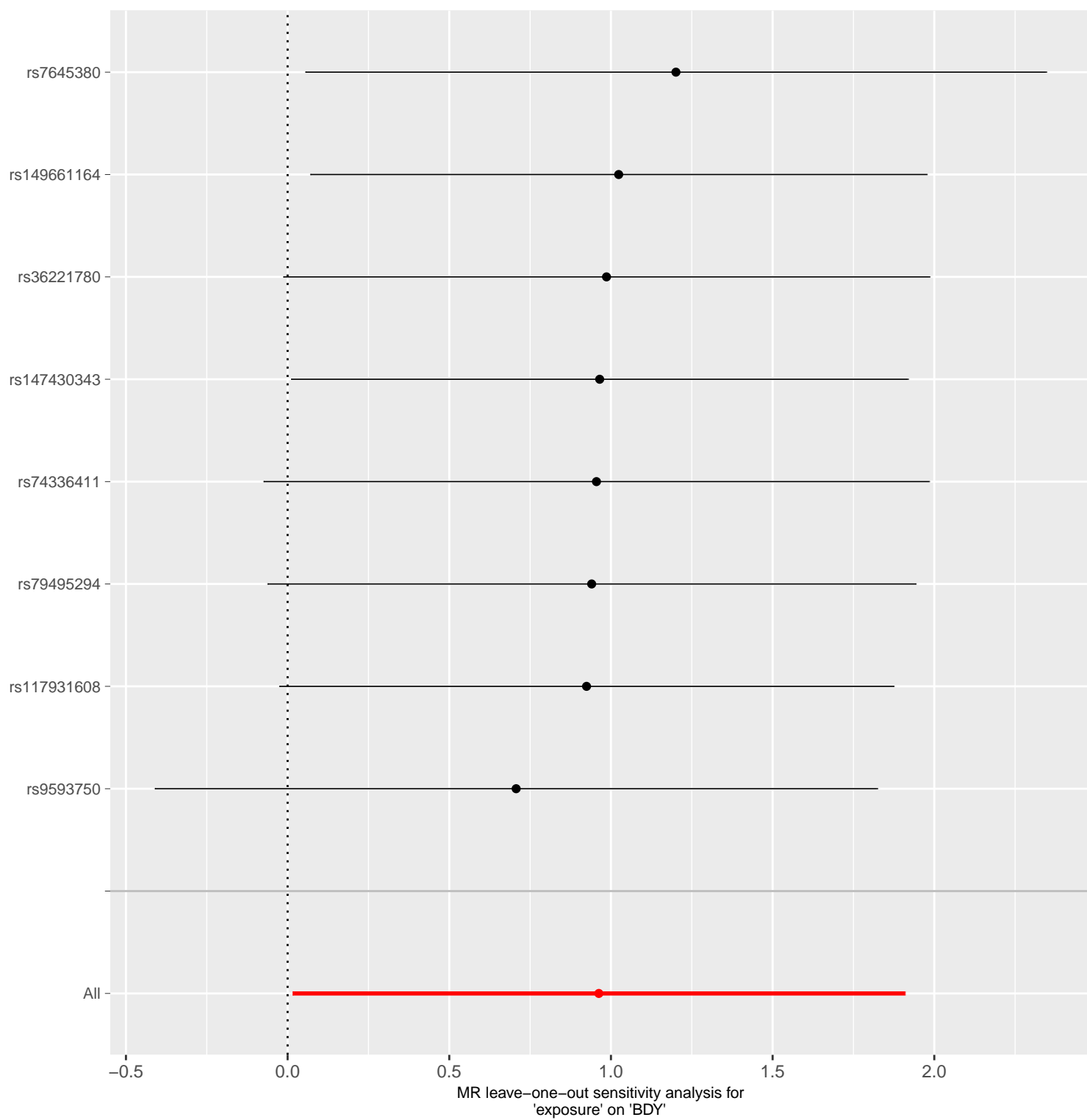

Supplement: Supplementary file 1 [file Data_Sheet_1.zip › Supplementary Materials/MR plots of saliva/Bronchiectasis/s__Aggregatibacter_sp000466335_mgs_2199/leave_one_out.pdf]

# MR Test

- Inverse variance weighted
- MR Egger
- Weighted median

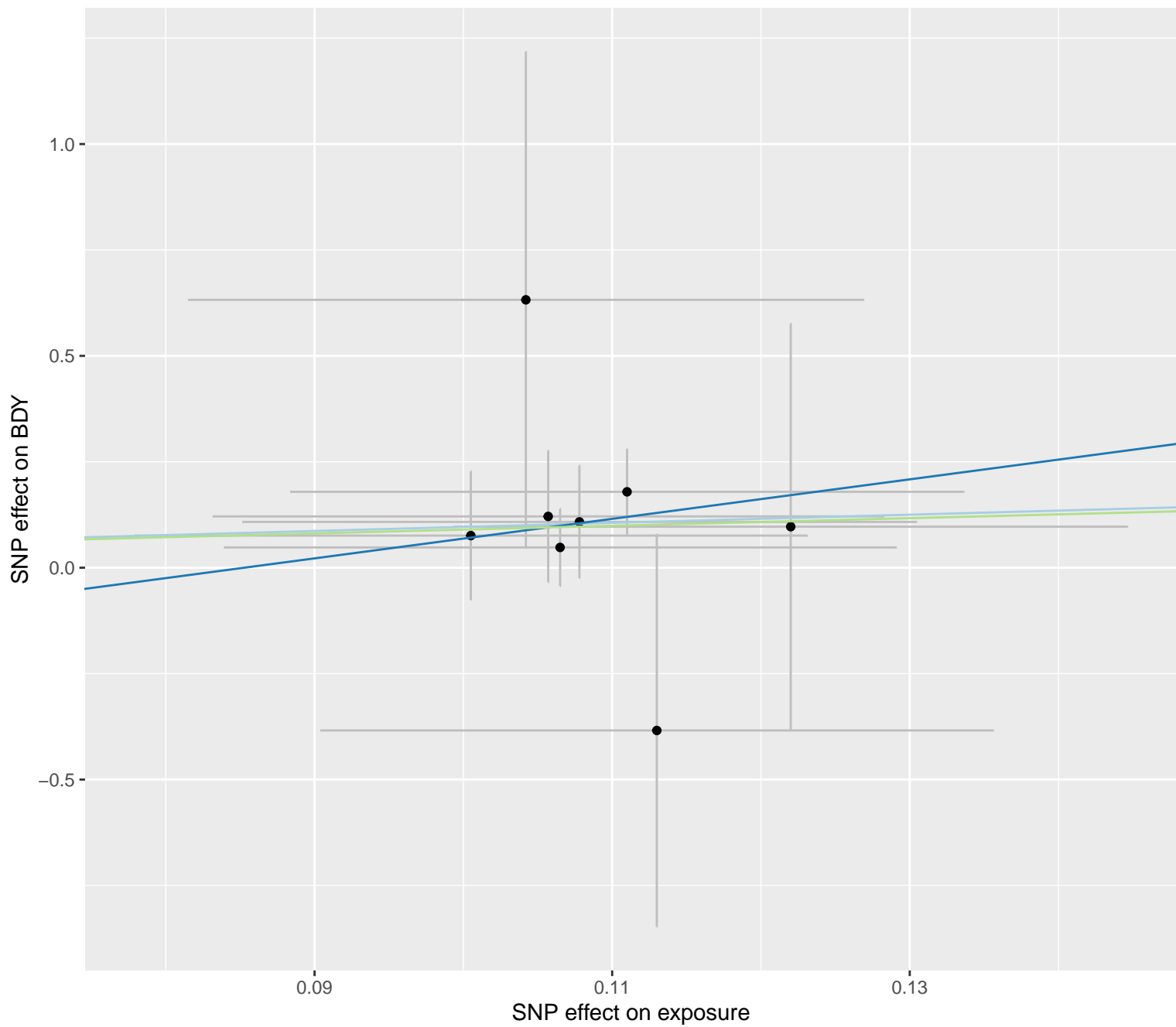

Supplement: Supplementary file 1 [file Data_Sheet_1.zip › Supplementary Materials/MR plots of saliva/Bronchiectasis/s__Aggregatibacter_sp000466335_mgs_2199/scatter.pdf]

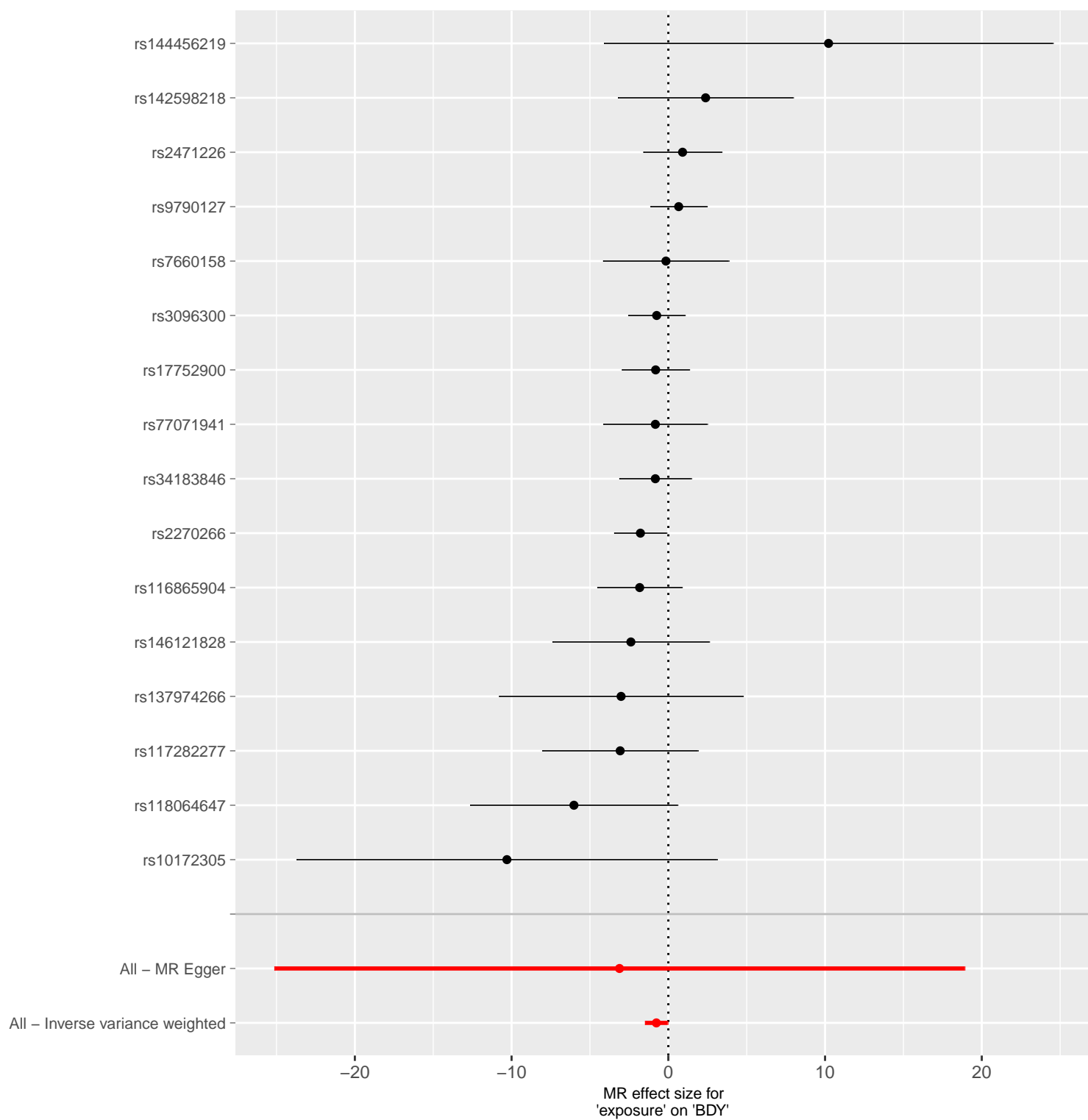

Supplement: Supplementary file 1 [file Data_Sheet_1.zip › Supplementary Materials/MR plots of saliva/Bronchiectasis/s__Aggregatibacter_sp000466335_mgs_2199_44/forest.pdf]

# MR Method

- Inverse variance weighted
- MR Egger

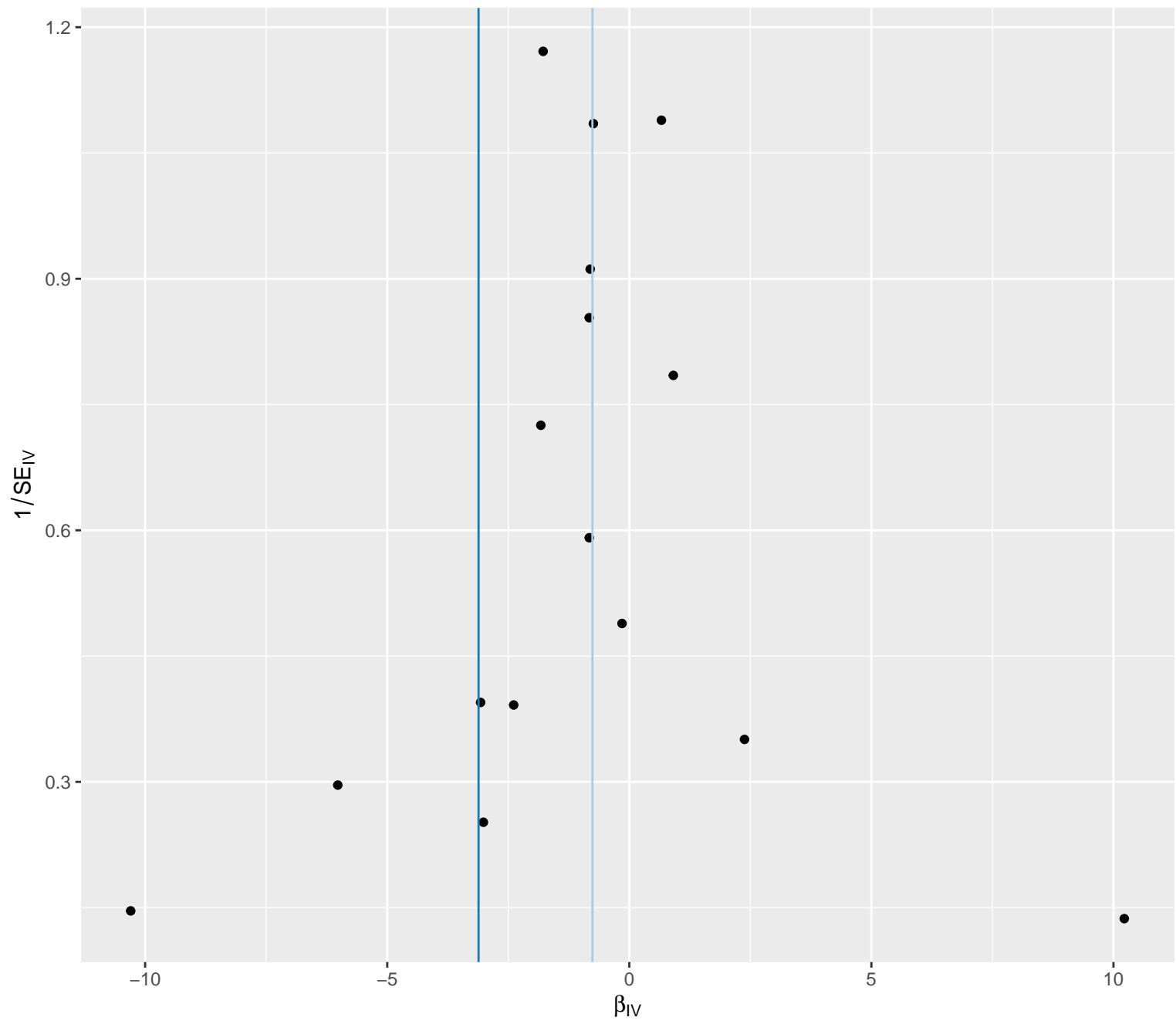

Supplement: Supplementary file 1 [file Data_Sheet_1.zip › Supplementary Materials/MR plots of saliva/Bronchiectasis/s__Aggregatibacter_sp000466335_mgs_2199_44/funnel.pdf]

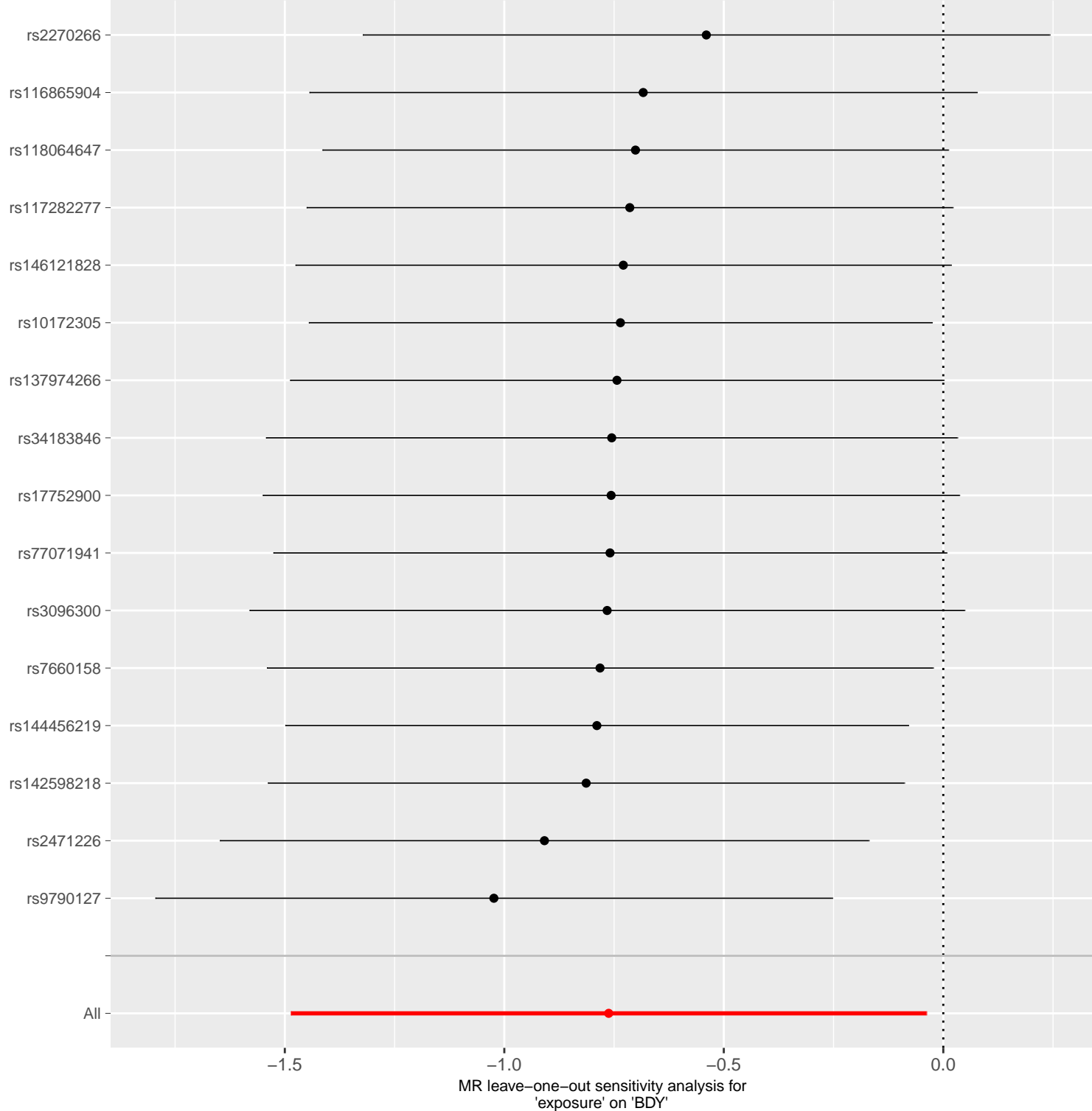

Supplement: Supplementary file 1 [file Data_Sheet_1.zip › Supplementary Materials/MR plots of saliva/Bronchiectasis/s__Aggregatibacter_sp000466335_mgs_2199_44/leave_one_out.pdf]

# MR Test

- Inverse variance weighted
- MR Egger
- Weighted median

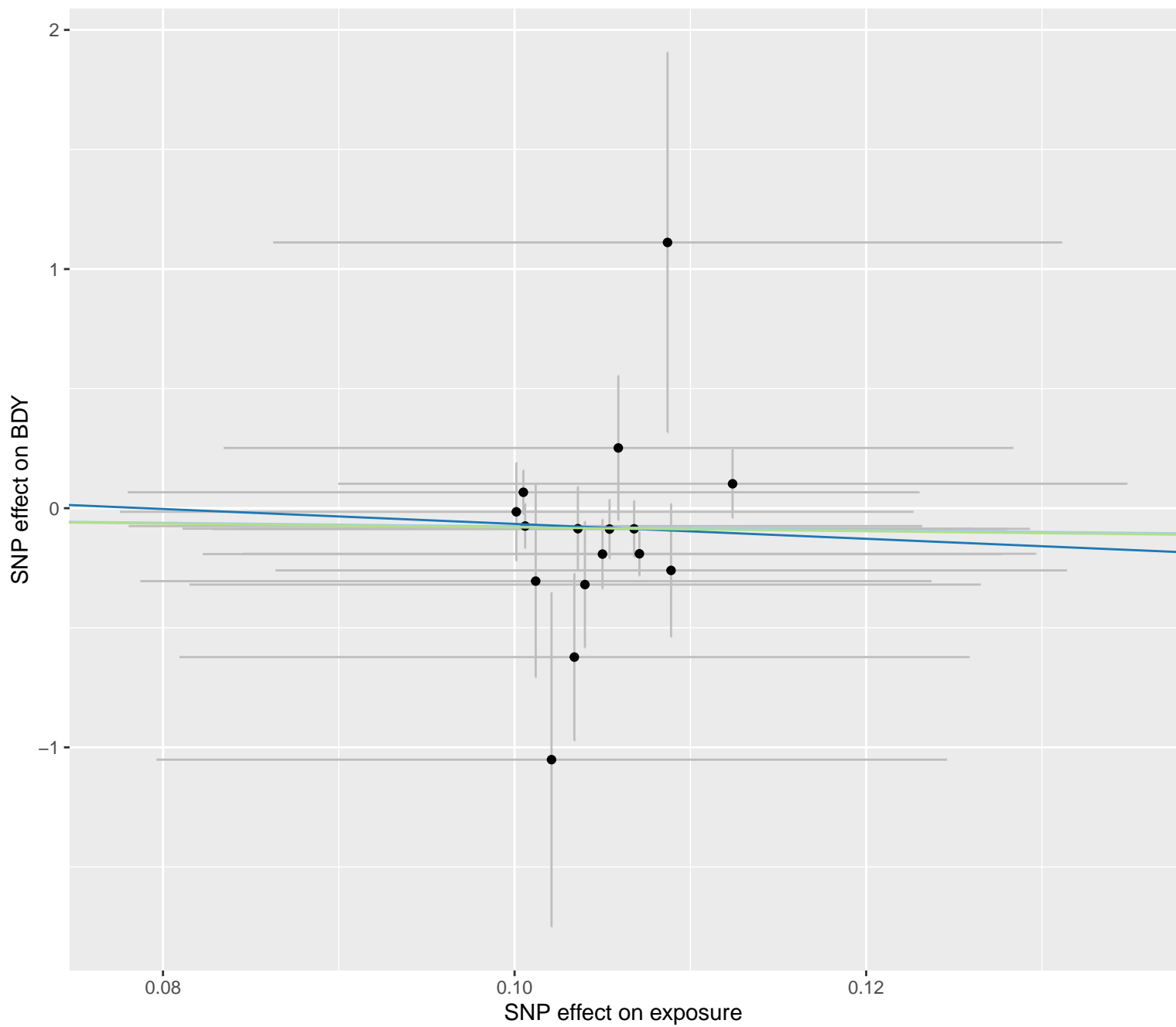

Supplement: Supplementary file 1 [file Data_Sheet_1.zip › Supplementary Materials/MR plots of saliva/Bronchiectasis/s__Aggregatibacter_sp000466335_mgs_2199_44/scatter.pdf]

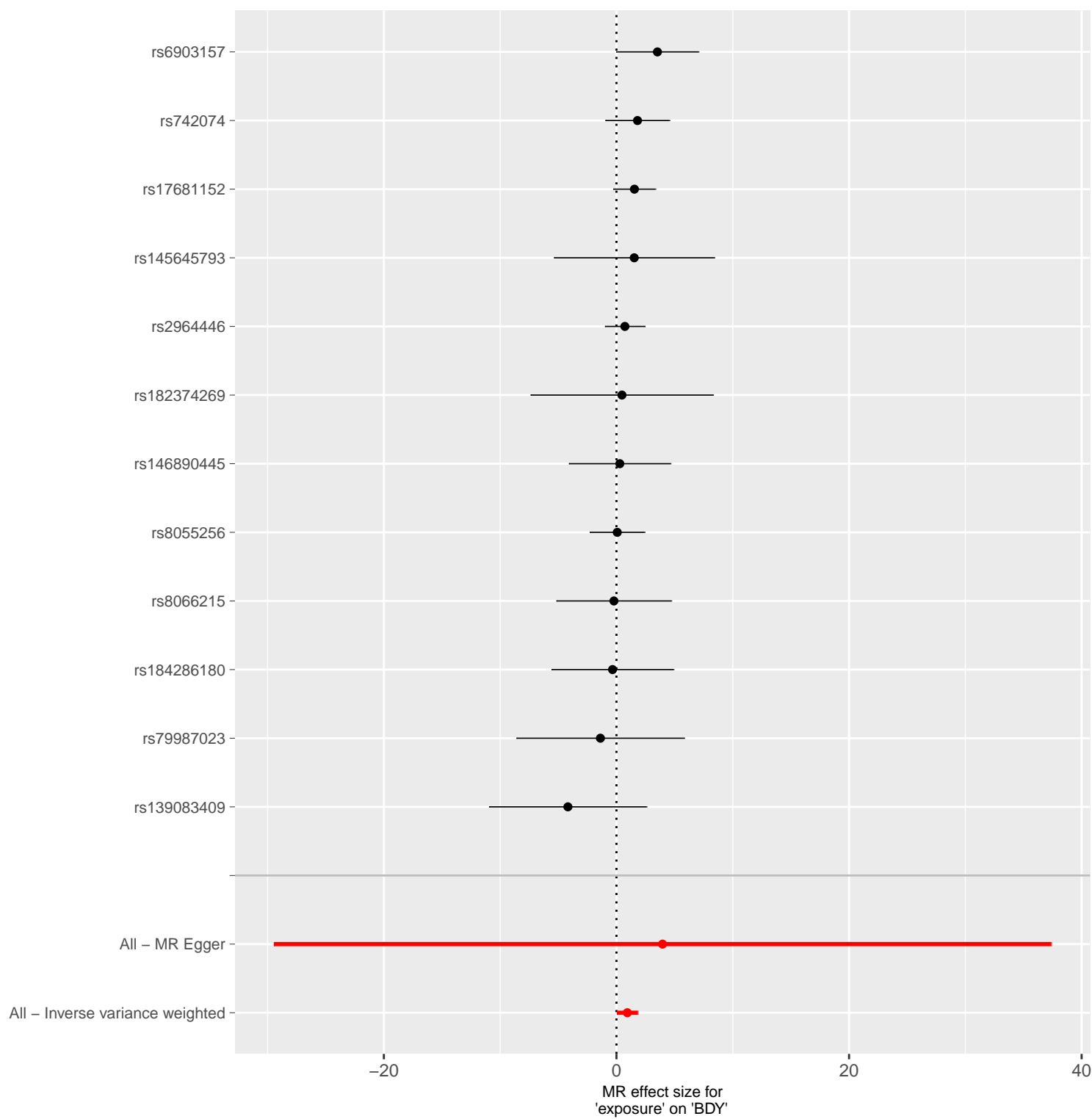

Supplement: Supplementary file 1 [file Data_Sheet_1.zip › Supplementary Materials/MR plots of saliva/Bronchiectasis/s__Fusobacterium_periodonticum_C_mgs_2270/forest.pdf]

# MR Method

- Inverse variance weighted
- MR Egger

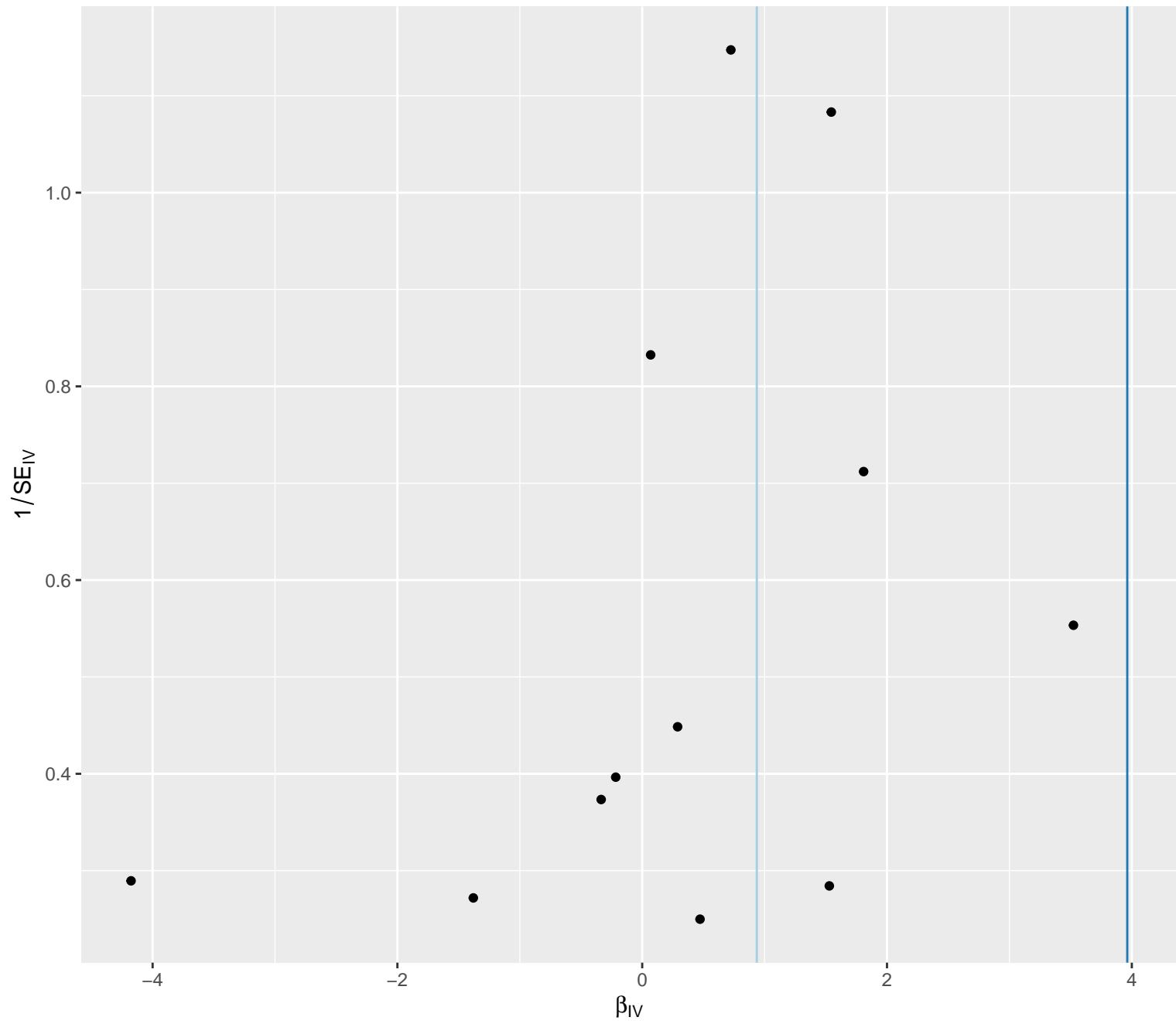

Supplement: Supplementary file 1 [file Data_Sheet_1.zip › Supplementary Materials/MR plots of saliva/Bronchiectasis/s__Fusobacterium_periodonticum_C_mgs_2270/funnel.pdf]

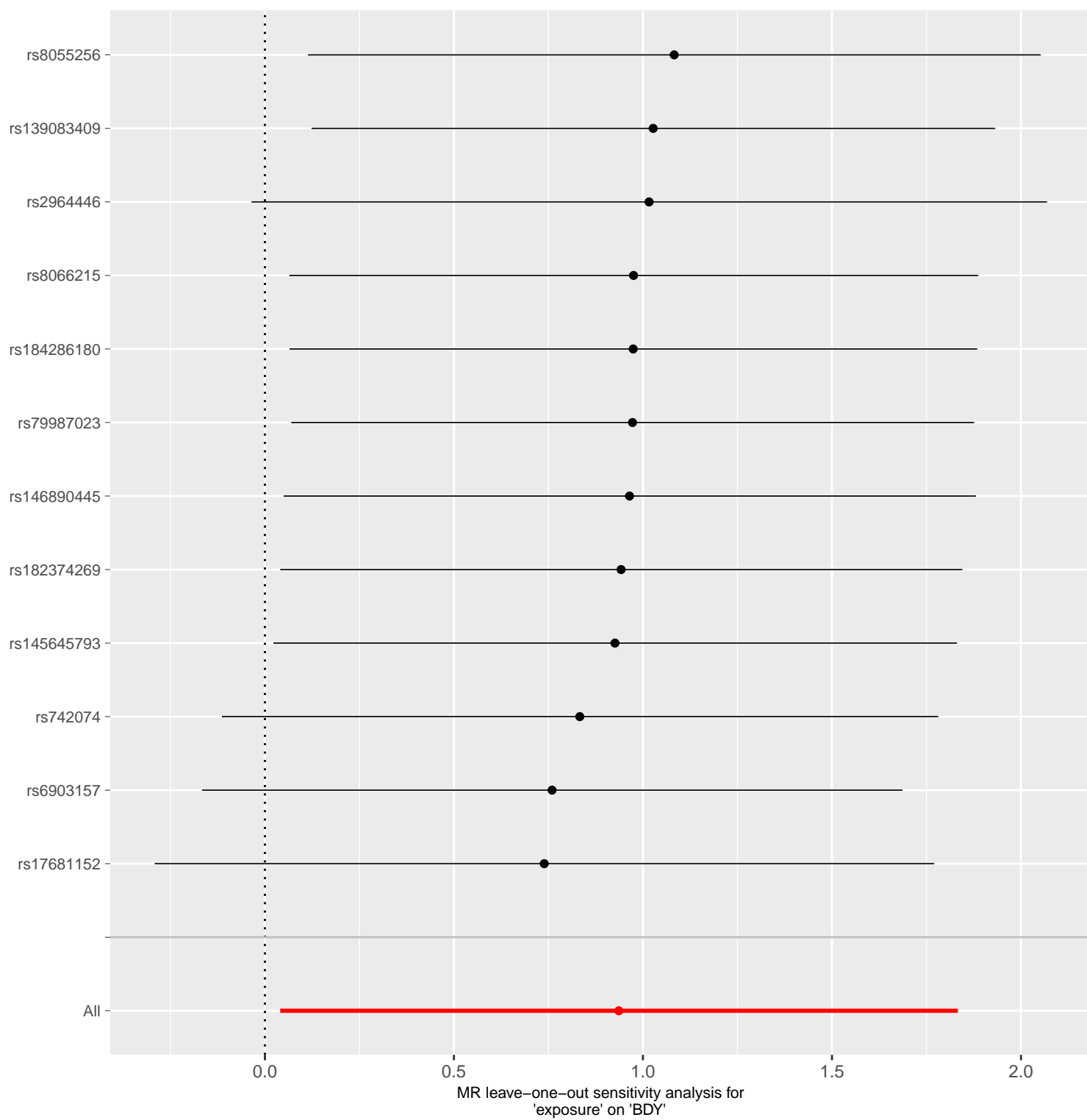

Supplement: Supplementary file 1 [file Data_Sheet_1.zip › Supplementary Materials/MR plots of saliva/Bronchiectasis/s__Fusobacterium_periodonticum_C_mgs_2270/leave_one_out.pdf]

# MR Test

- Inverse variance weighted
- MR Egger
- Weighted median

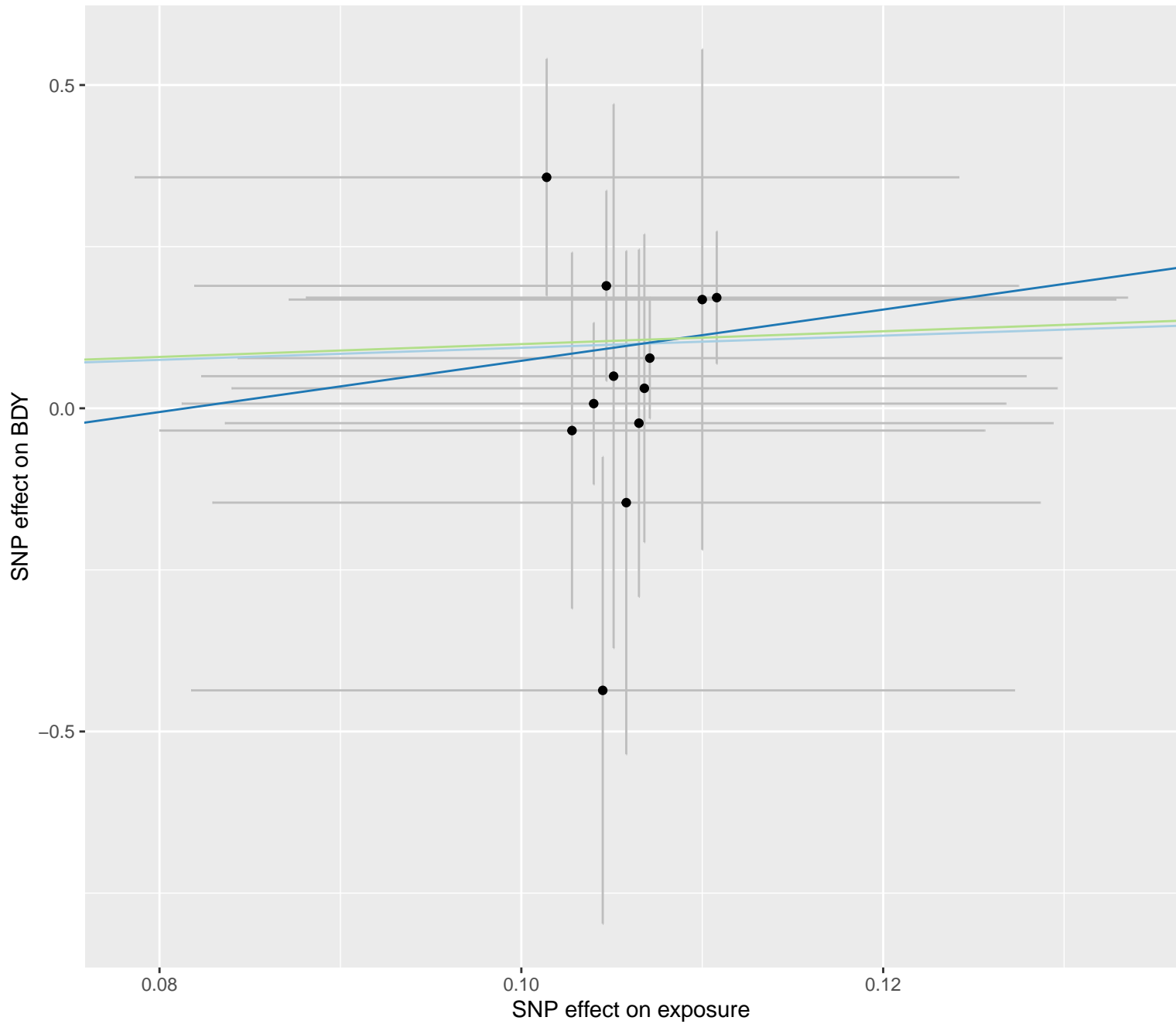

Supplement: Supplementary file 1 [file Data_Sheet_1.zip › Supplementary Materials/MR plots of saliva/Bronchiectasis/s__Fusobacterium_periodonticum_C_mgs_2270/scatter.pdf]

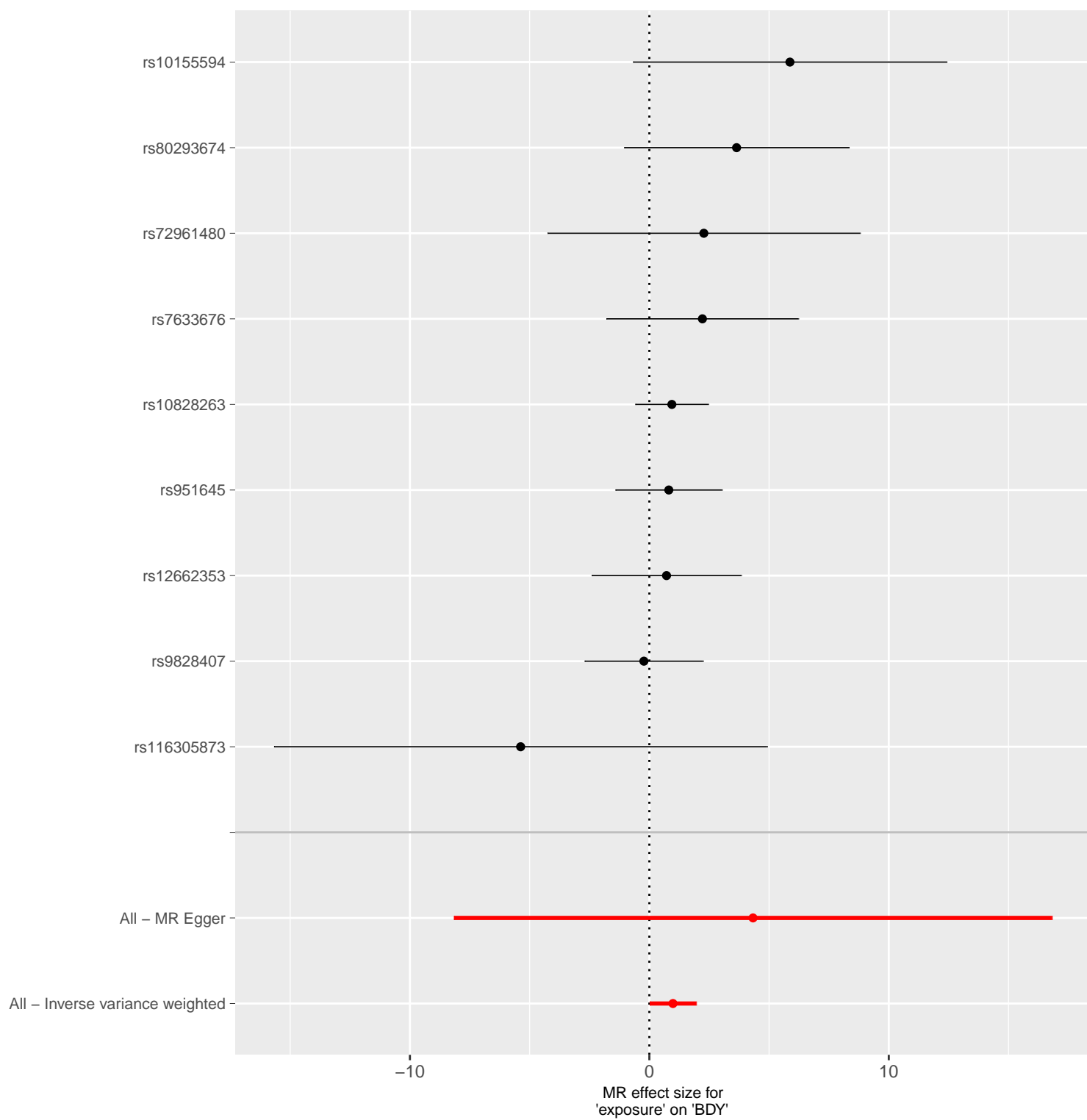

Supplement: Supplementary file 1 [file Data_Sheet_1.zip › Supplementary Materials/MR plots of saliva/Bronchiectasis/s__Fusobacterium_periodonticum_C_mgs_2270_43/forest.pdf]

# MR Method

- Inverse variance weighted
- MR Egger

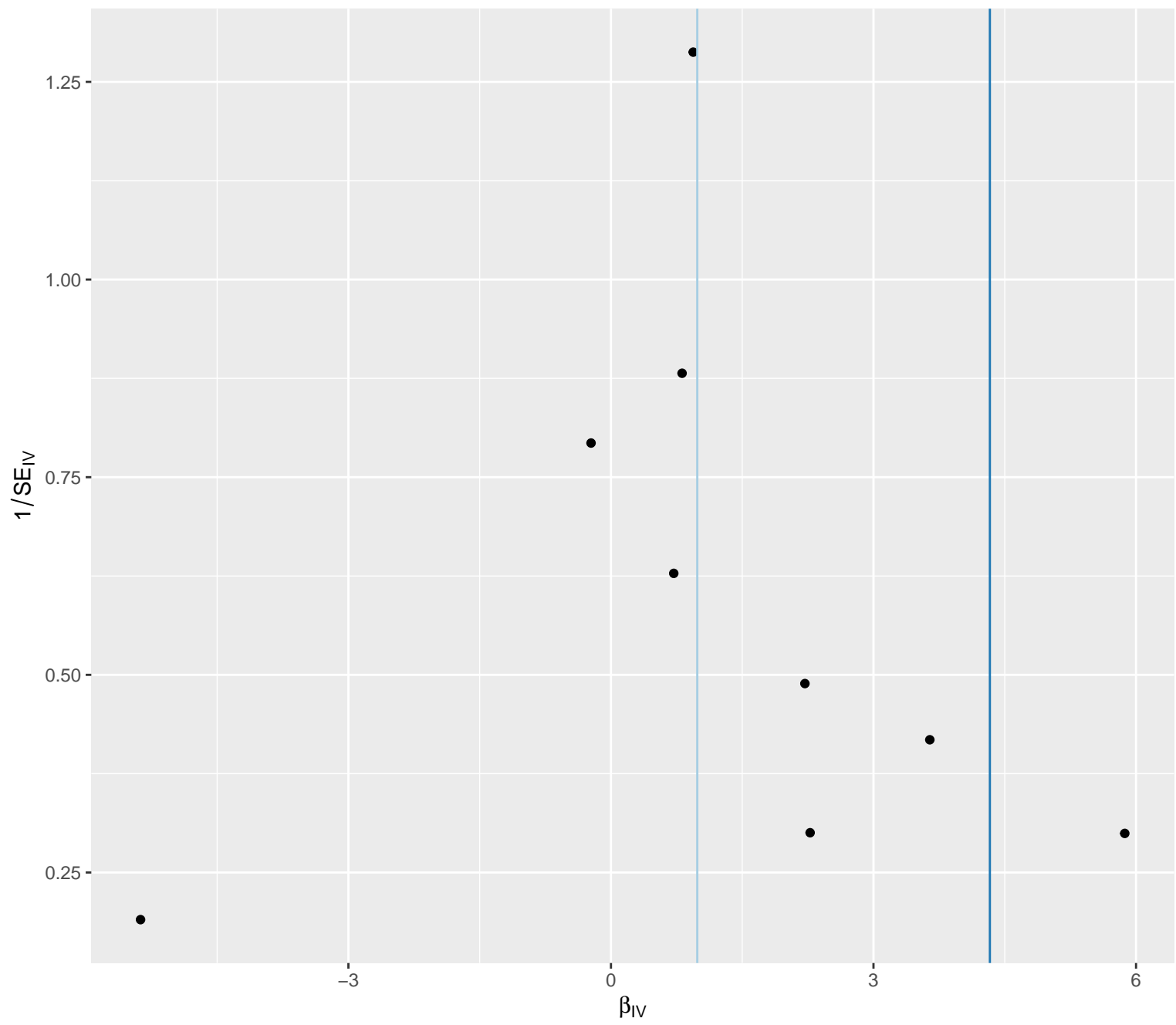

Supplement: Supplementary file 1 [file Data_Sheet_1.zip › Supplementary Materials/MR plots of saliva/Bronchiectasis/s__Fusobacterium_periodonticum_C_mgs_2270_43/funnel.pdf]

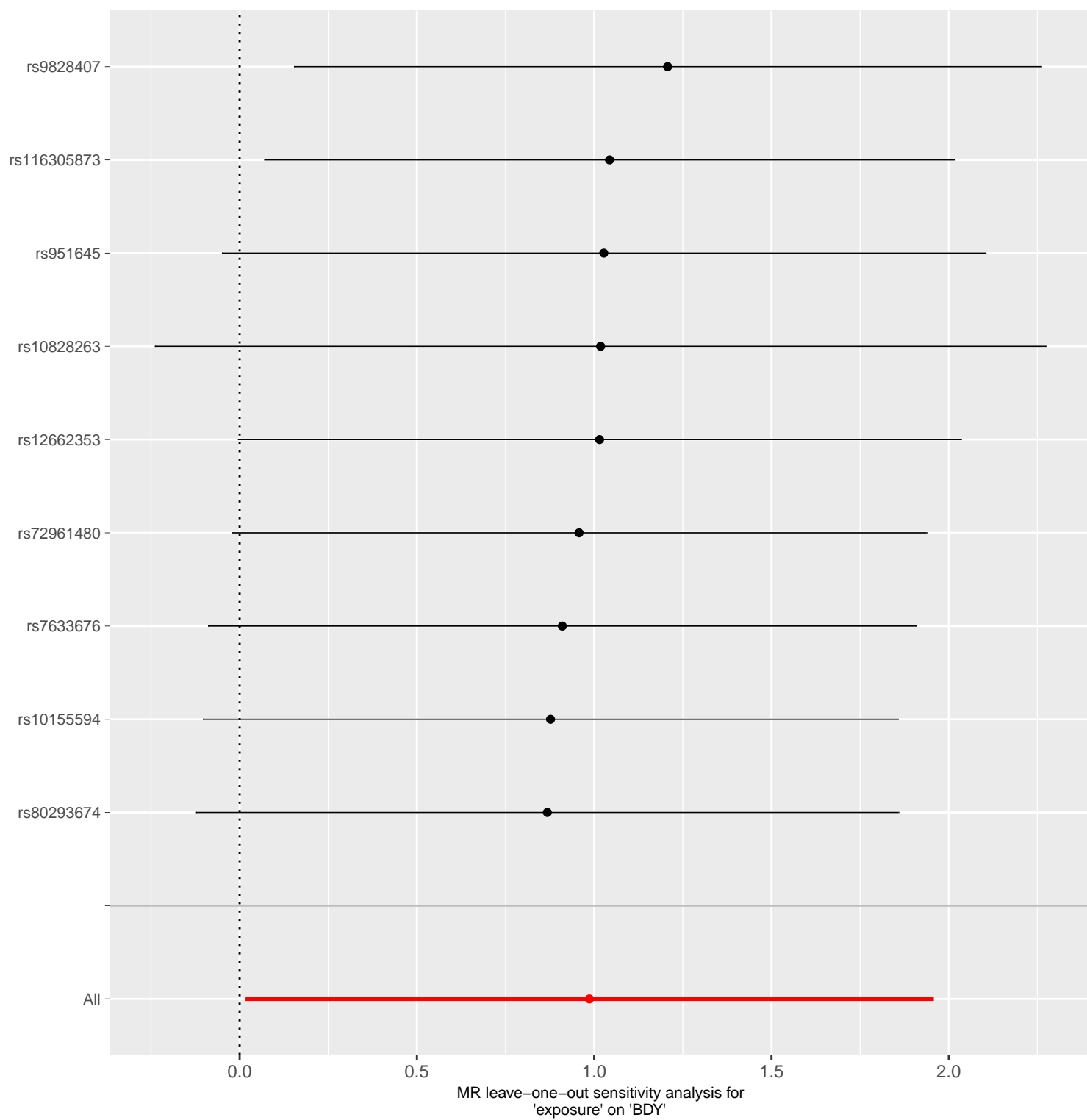

Supplement: Supplementary file 1 [file Data_Sheet_1.zip › Supplementary Materials/MR plots of saliva/Bronchiectasis/s__Fusobacterium_periodonticum_C_mgs_2270_43/leave_one_out.pdf]

# MR Test

- Inverse variance weighted
- MR Egger
- Weighted median

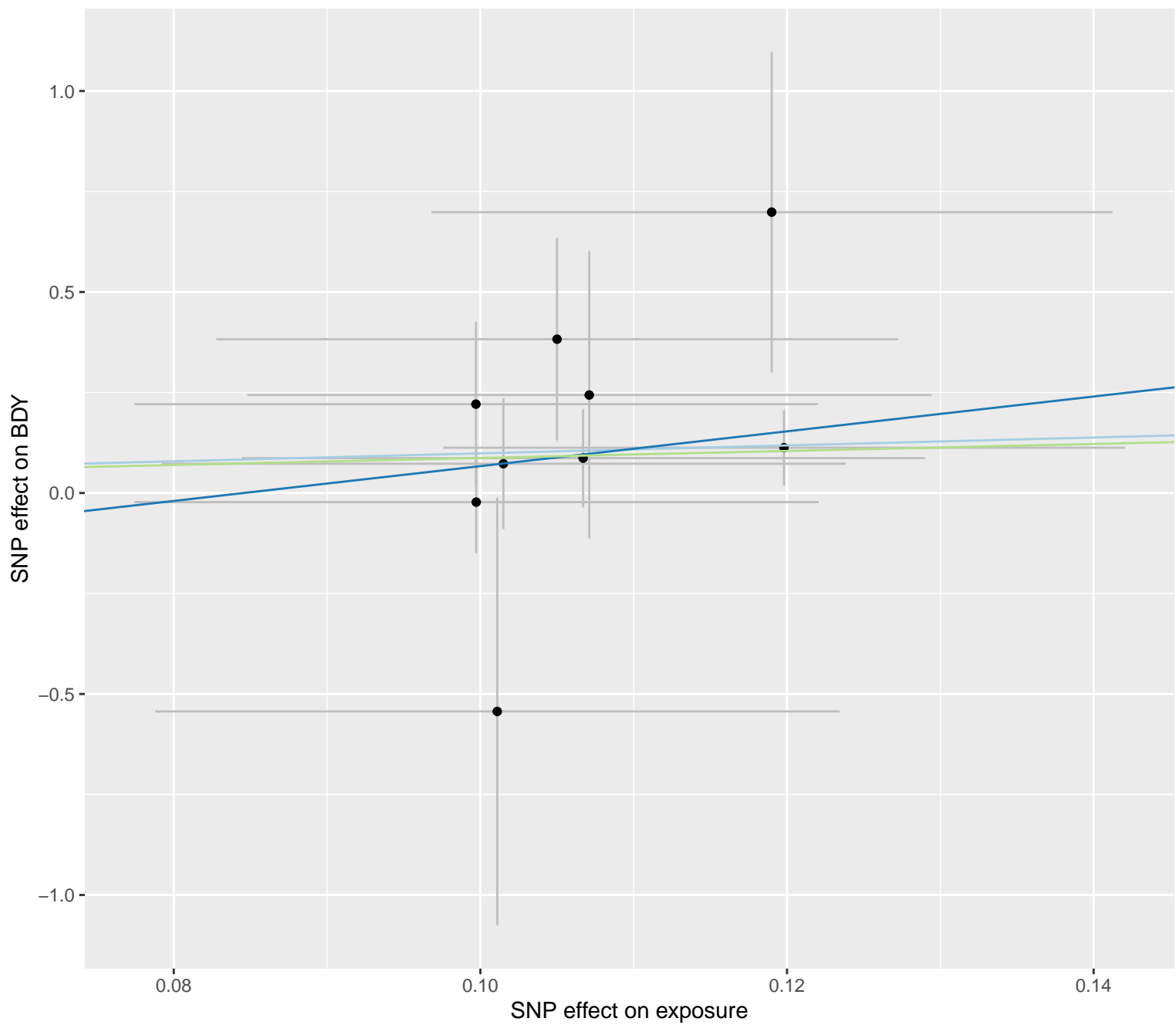

Supplement: Supplementary file 1 [file Data_Sheet_1.zip › Supplementary Materials/MR plots of saliva/Bronchiectasis/s__Fusobacterium_periodonticum_C_mgs_2270_43/scatter.pdf]

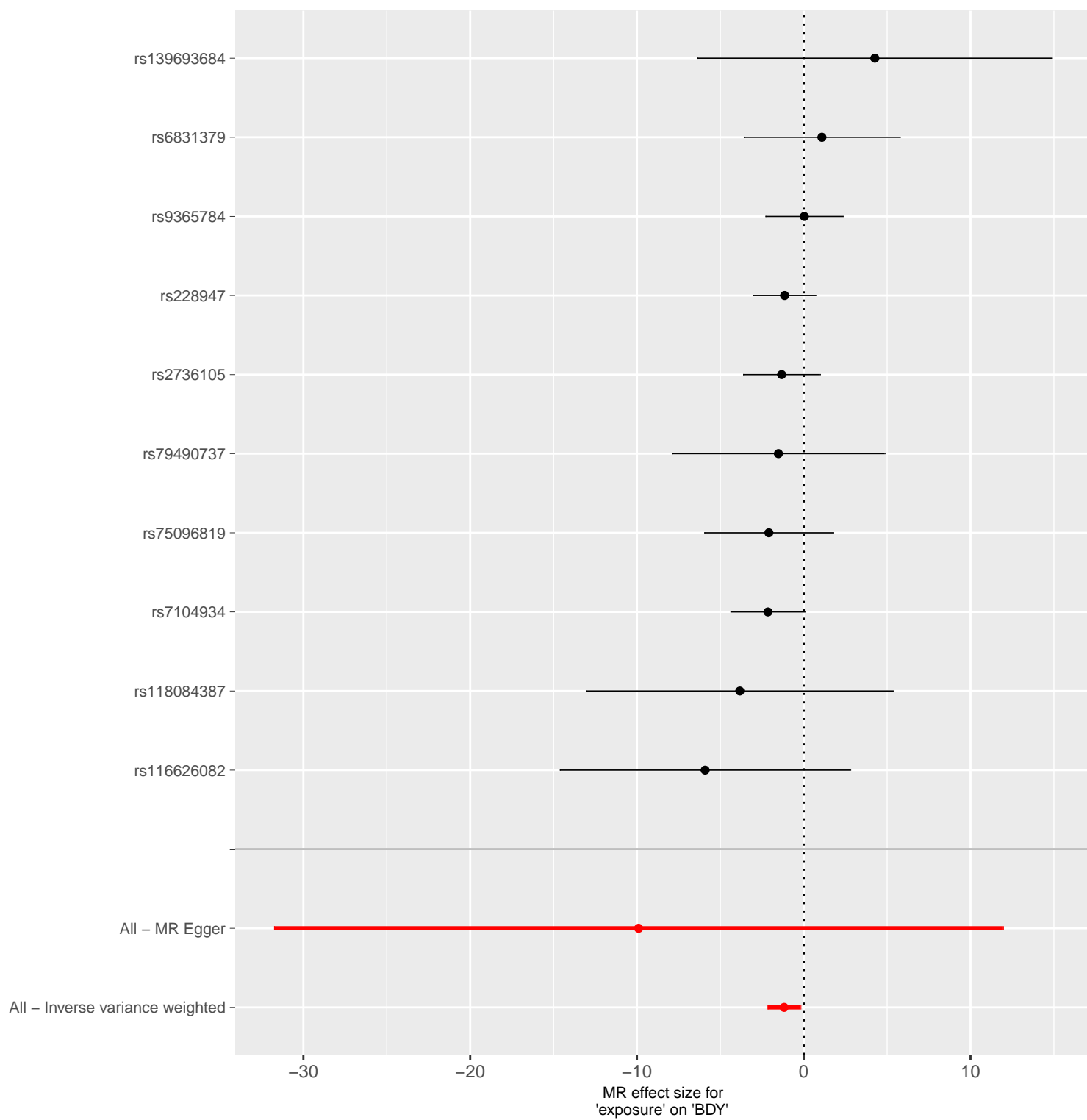

Supplement: Supplementary file 1 [file Data_Sheet_1.zip › Supplementary Materials/MR plots of saliva/Bronchiectasis/s__Fusobacterium_periodonticum_C_mgs_3047_45/forest.pdf]

# MR Method

- Inverse variance weighted
- MR Egger

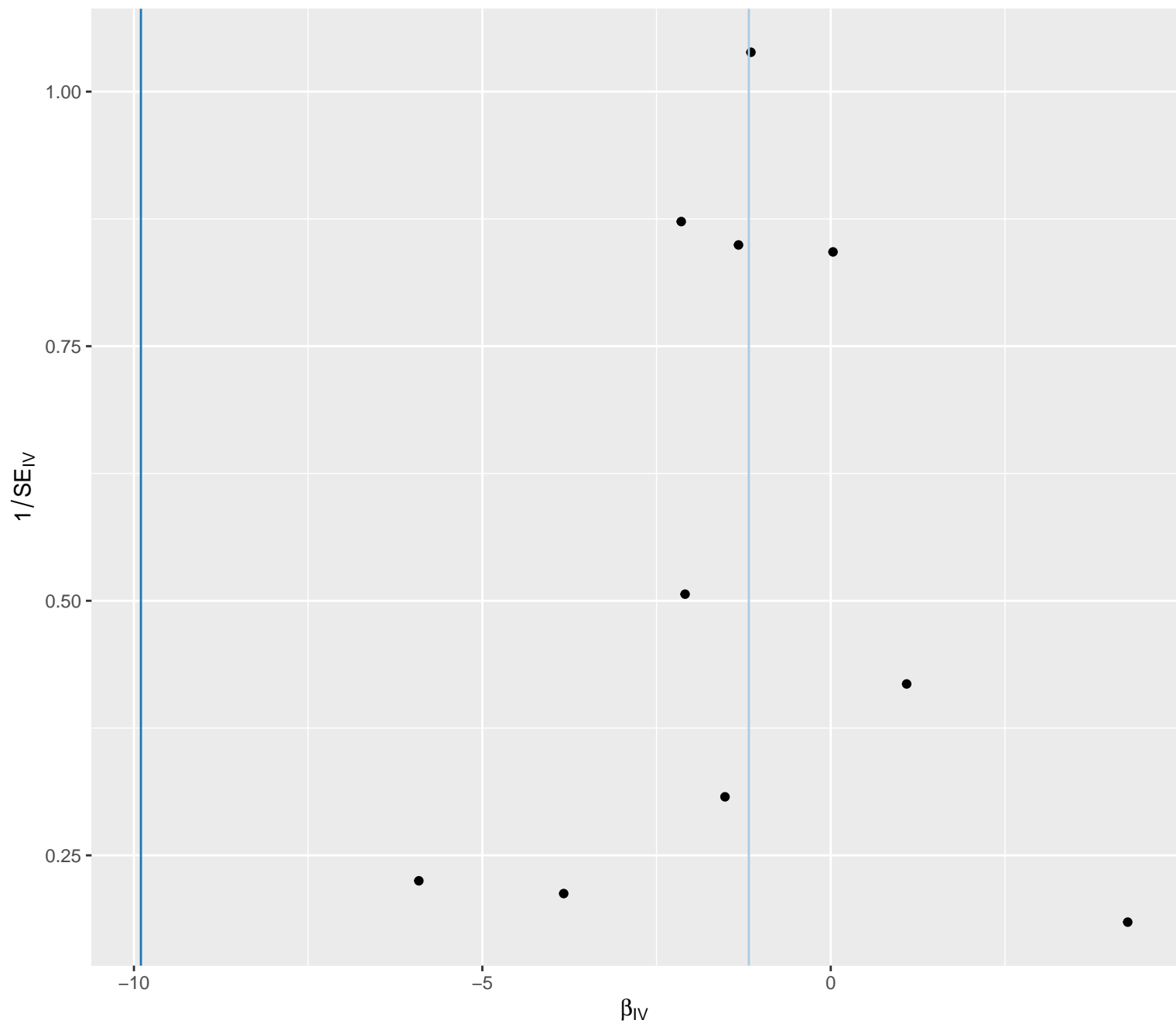

Supplement: Supplementary file 1 [file Data_Sheet_1.zip › Supplementary Materials/MR plots of saliva/Bronchiectasis/s__Fusobacterium_periodonticum_C_mgs_3047_45/funnel.pdf]

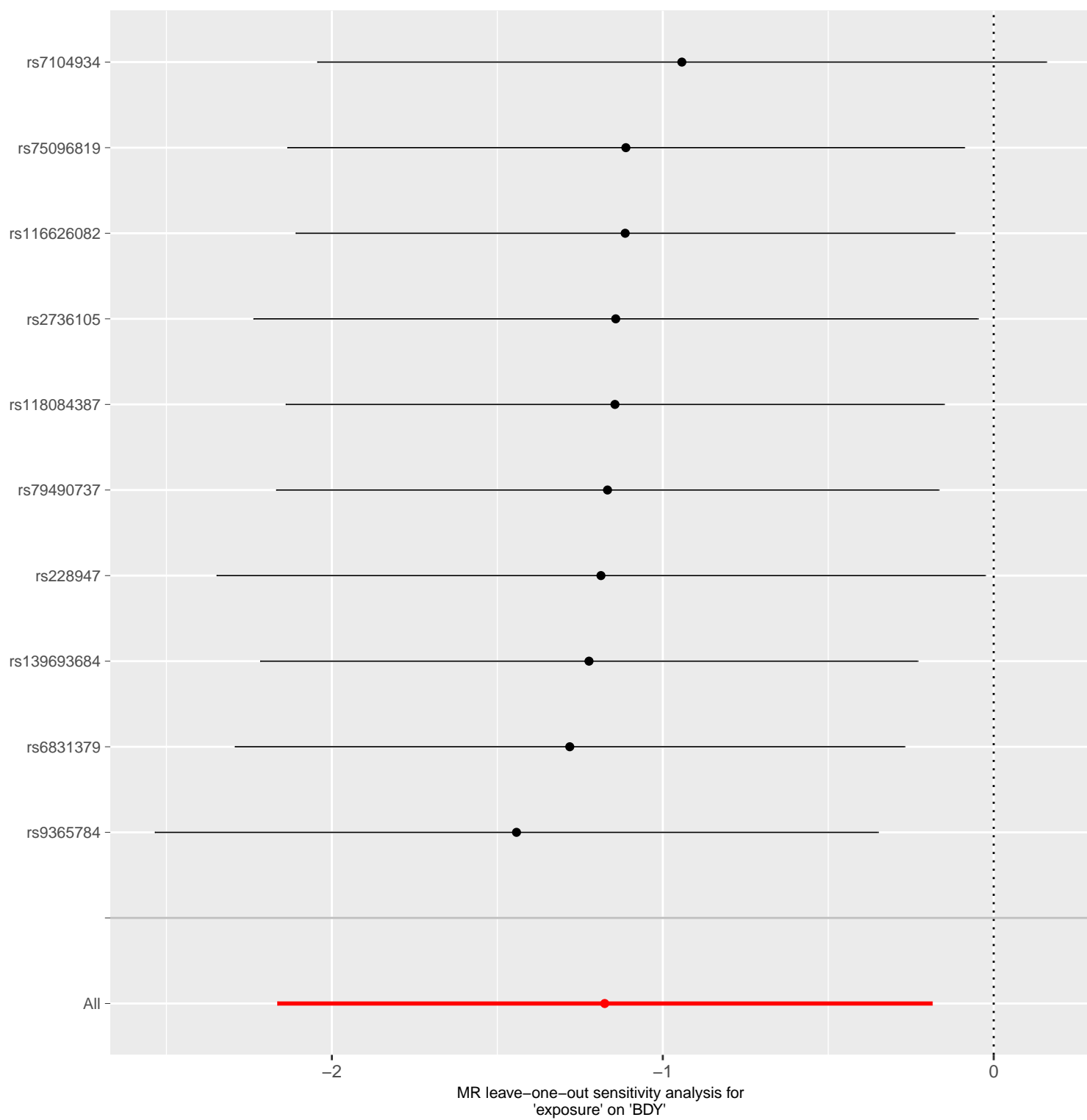

Supplement: Supplementary file 1 [file Data_Sheet_1.zip › Supplementary Materials/MR plots of saliva/Bronchiectasis/s__Fusobacterium_periodonticum_C_mgs_3047_45/leave_one_out.pdf]

# MR Test

- Inverse variance weighted
- MR Egger
- Weighted median

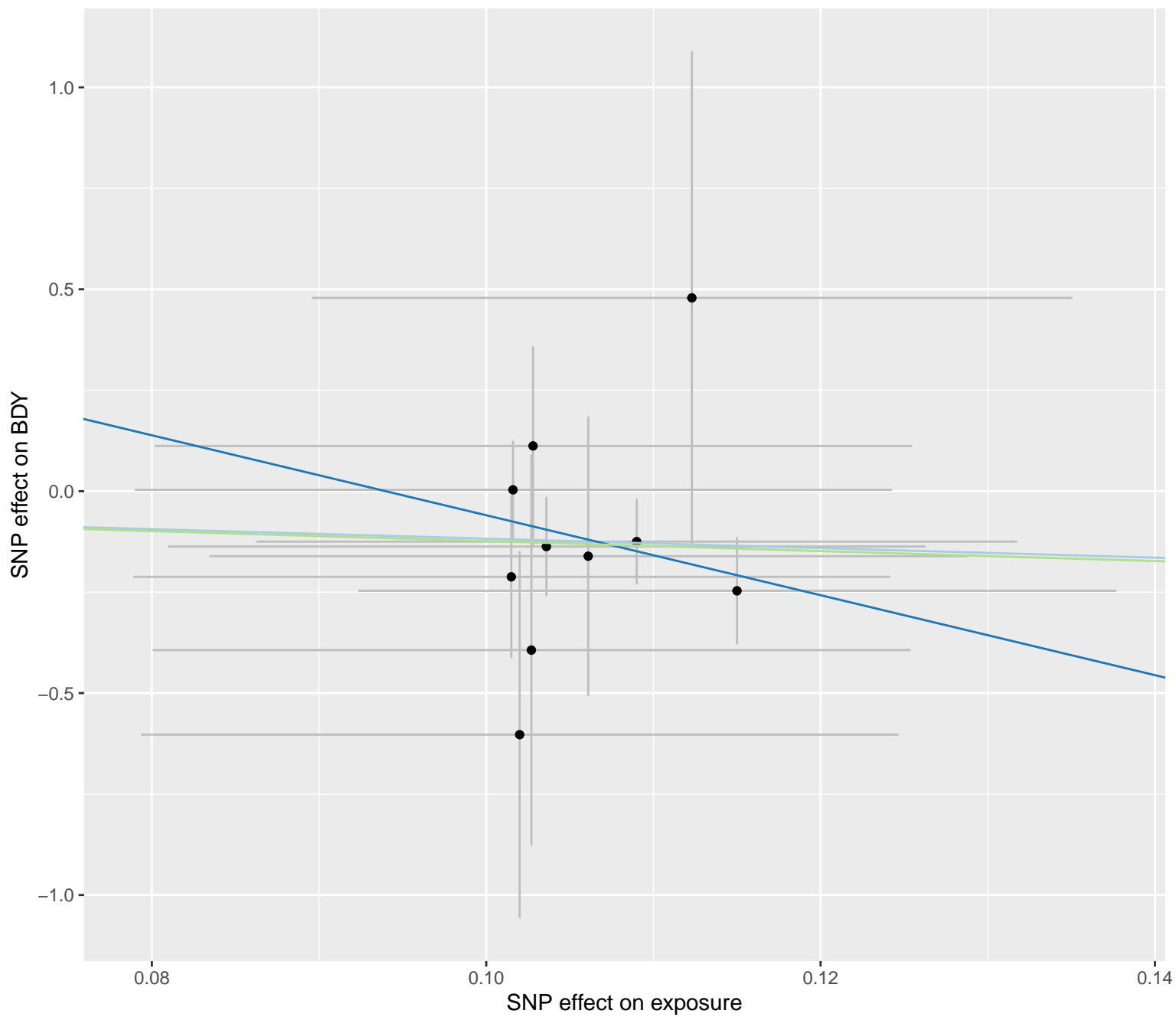

Supplement: Supplementary file 1 [file Data_Sheet_1.zip › Supplementary Materials/MR plots of saliva/Bronchiectasis/s__Fusobacterium_periodonticum_C_mgs_3047_45/scatter.pdf]

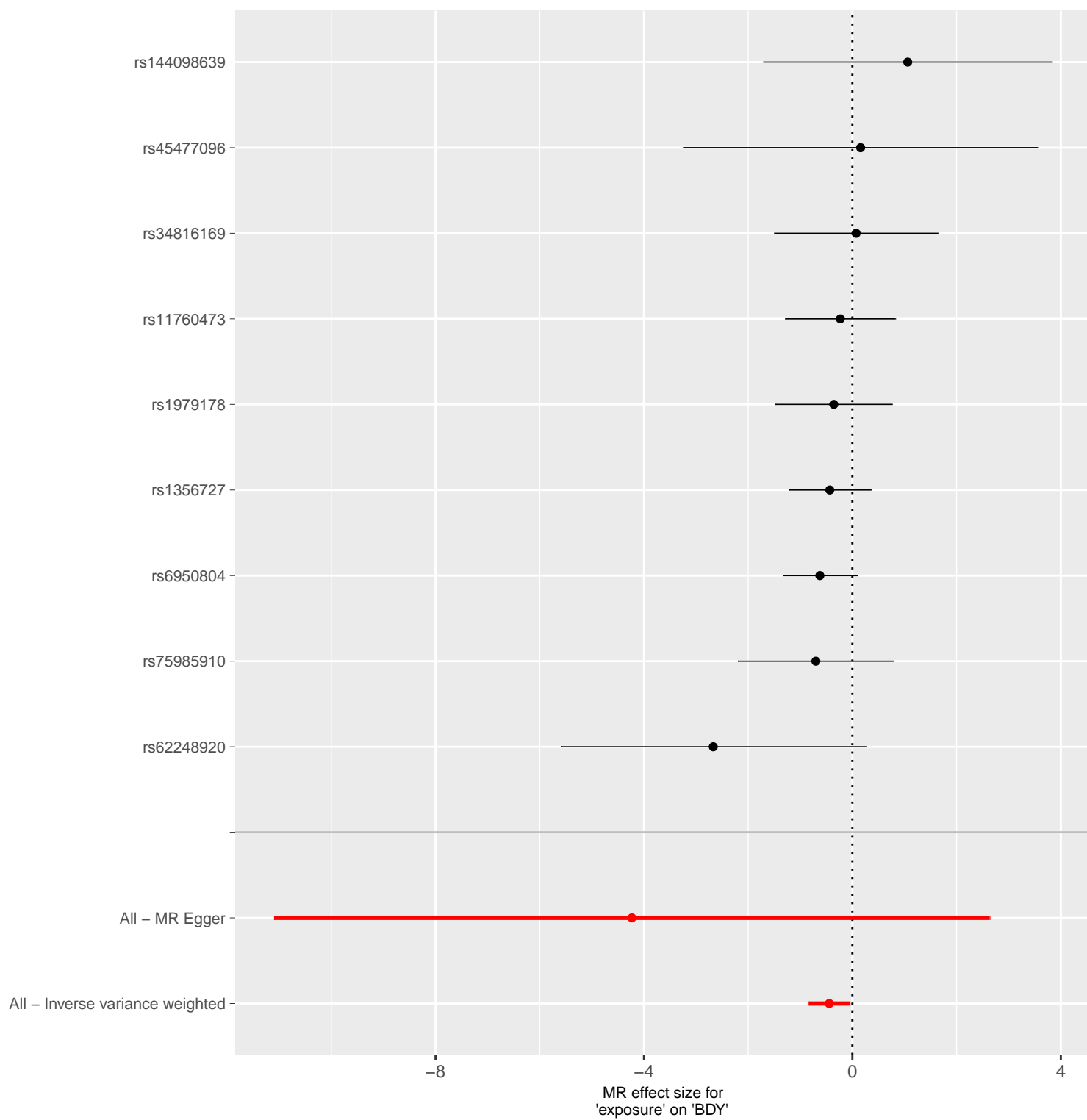

Supplement: Supplementary file 1 [file Data_Sheet_1.zip › Supplementary Materials/MR plots of saliva/Bronchitis/g__TM7x/forest.pdf]

# MR Method

- Inverse variance weighted
- MR Egger

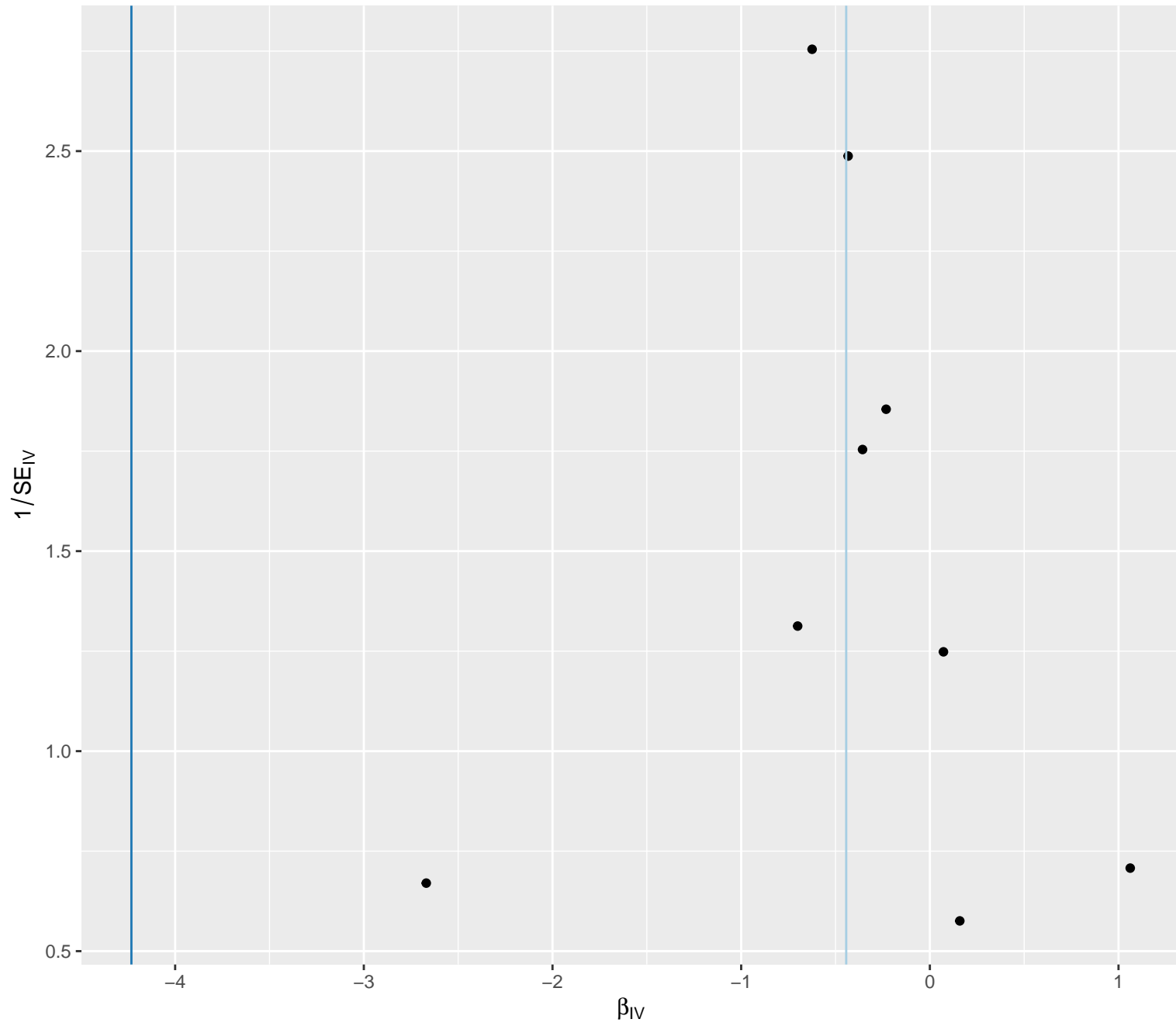

Supplement: Supplementary file 1 [file Data_Sheet_1.zip › Supplementary Materials/MR plots of saliva/Bronchitis/g__TM7x/funnel.pdf]

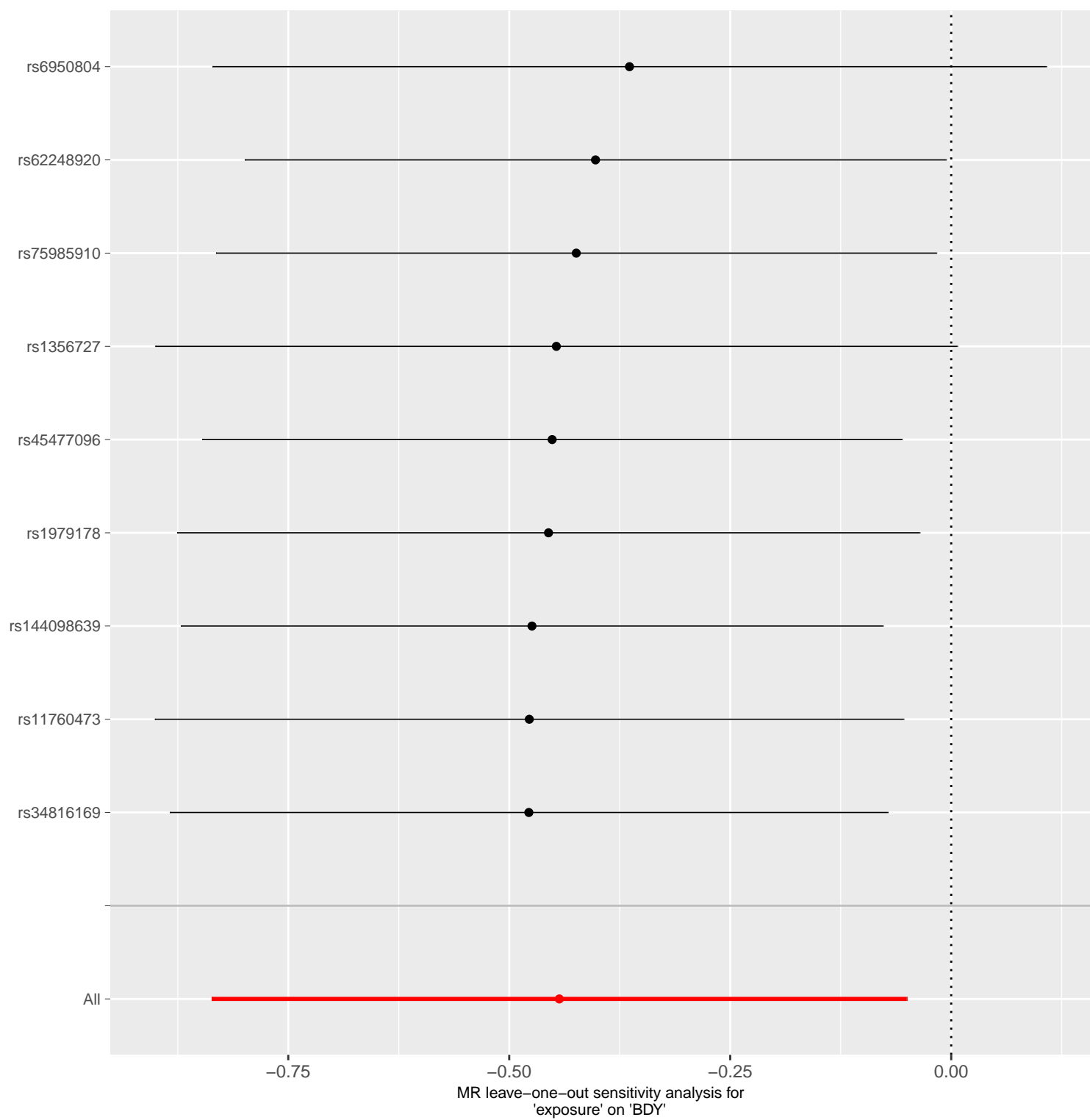

Supplement: Supplementary file 1 [file Data_Sheet_1.zip › Supplementary Materials/MR plots of saliva/Bronchitis/g__TM7x/leave_one_out.pdf]

# MR Test

- Inverse variance weighted
- MR Egger
- Weighted median

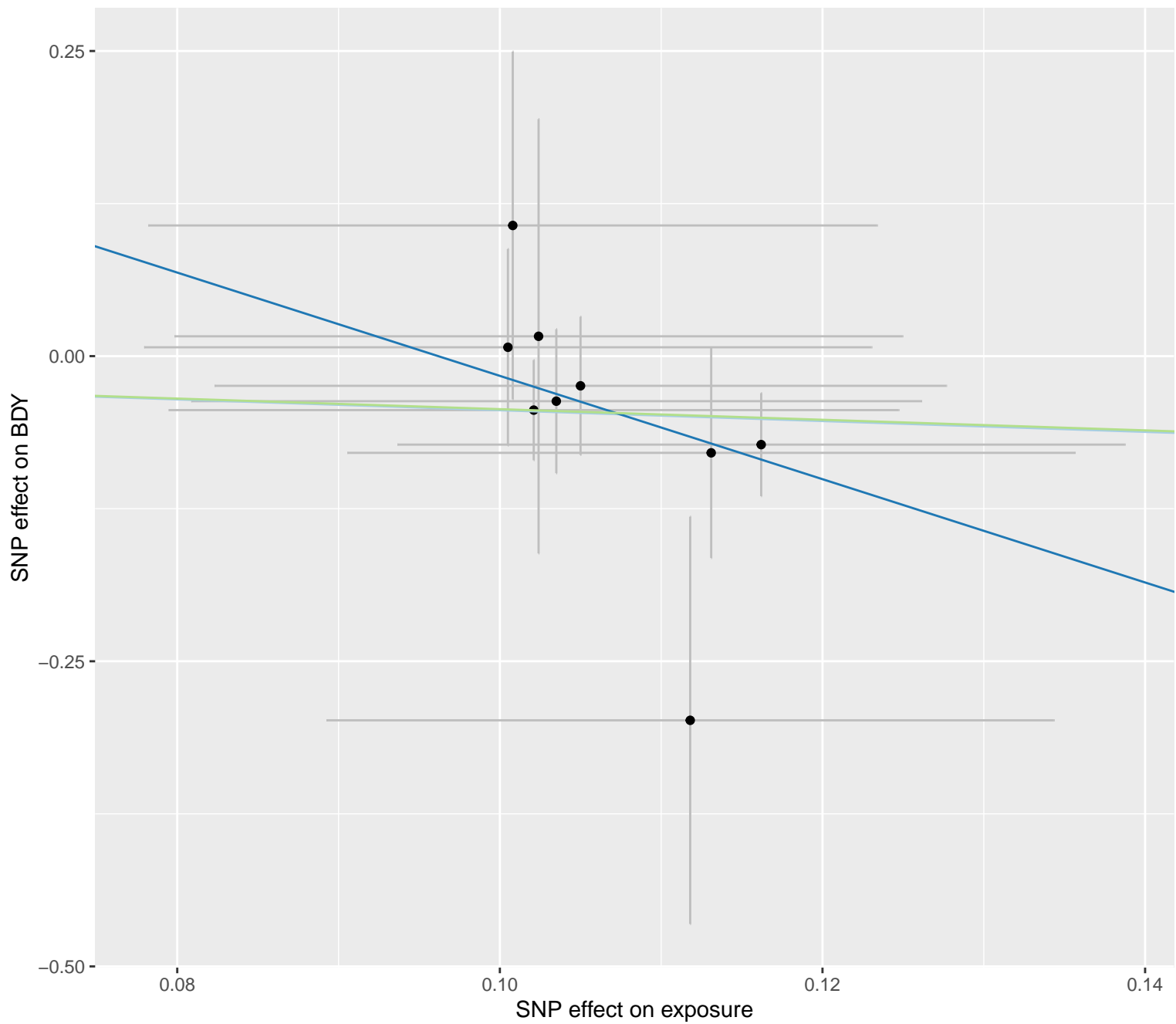

Supplement: Supplementary file 1 [file Data_Sheet_1.zip › Supplementary Materials/MR plots of saliva/Bronchitis/g__TM7x/scatter.pdf]

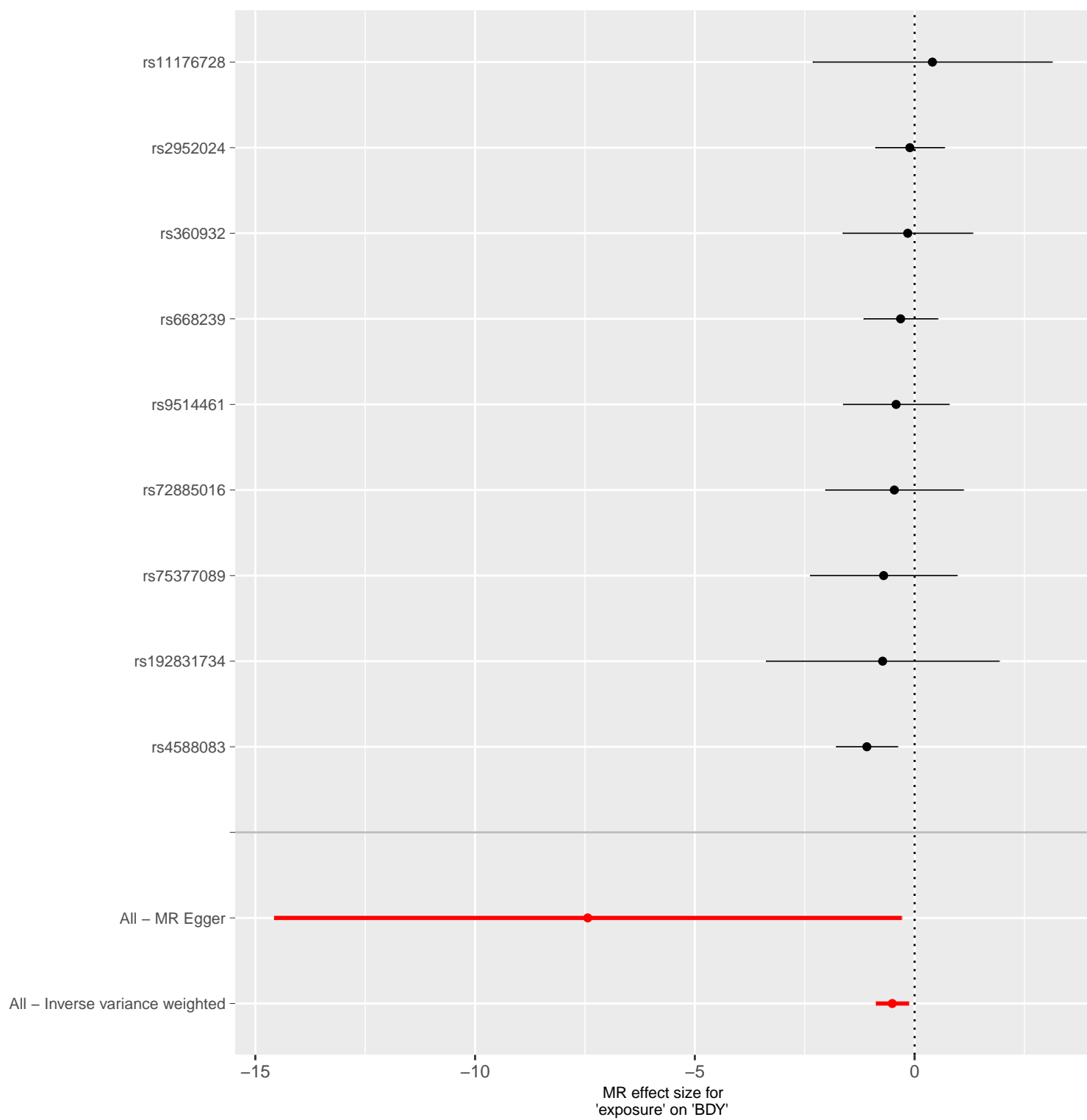

Supplement: Supplementary file 1 [file Data_Sheet_1.zip › Supplementary Materials/MR plots of saliva/Bronchitis/g__unclassified_mgs_377/forest.pdf]

# MR Method

- Inverse variance weighted
- MR Egger

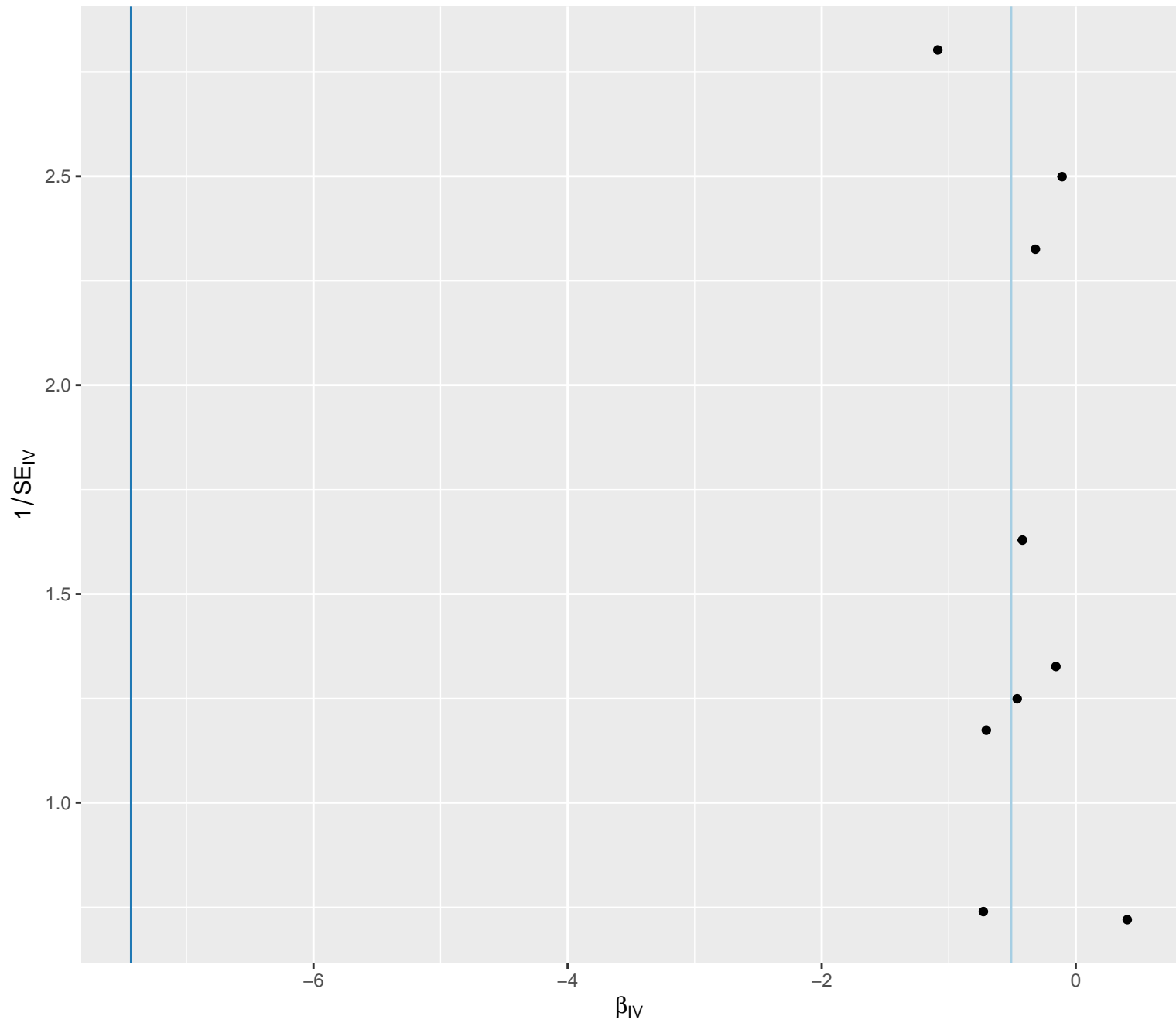

Supplement: Supplementary file 1 [file Data_Sheet_1.zip › Supplementary Materials/MR plots of saliva/Bronchitis/g__unclassified_mgs_377/funnel.pdf]

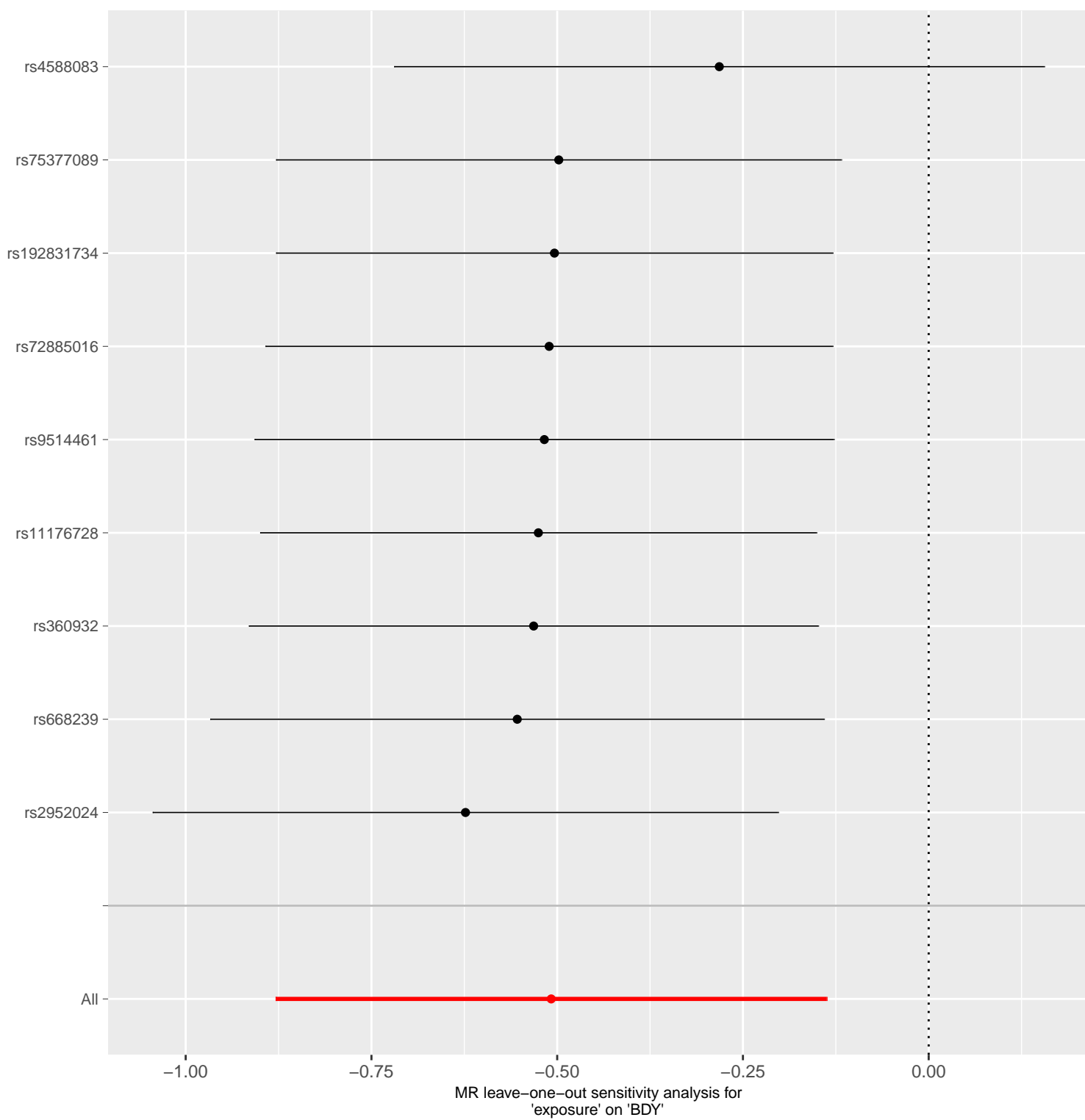

Supplement: Supplementary file 1 [file Data_Sheet_1.zip › Supplementary Materials/MR plots of saliva/Bronchitis/g__unclassified_mgs_377/leave_one_out.pdf]

# MR Test

- Inverse variance weighted
- MR Egger
- Weighted median

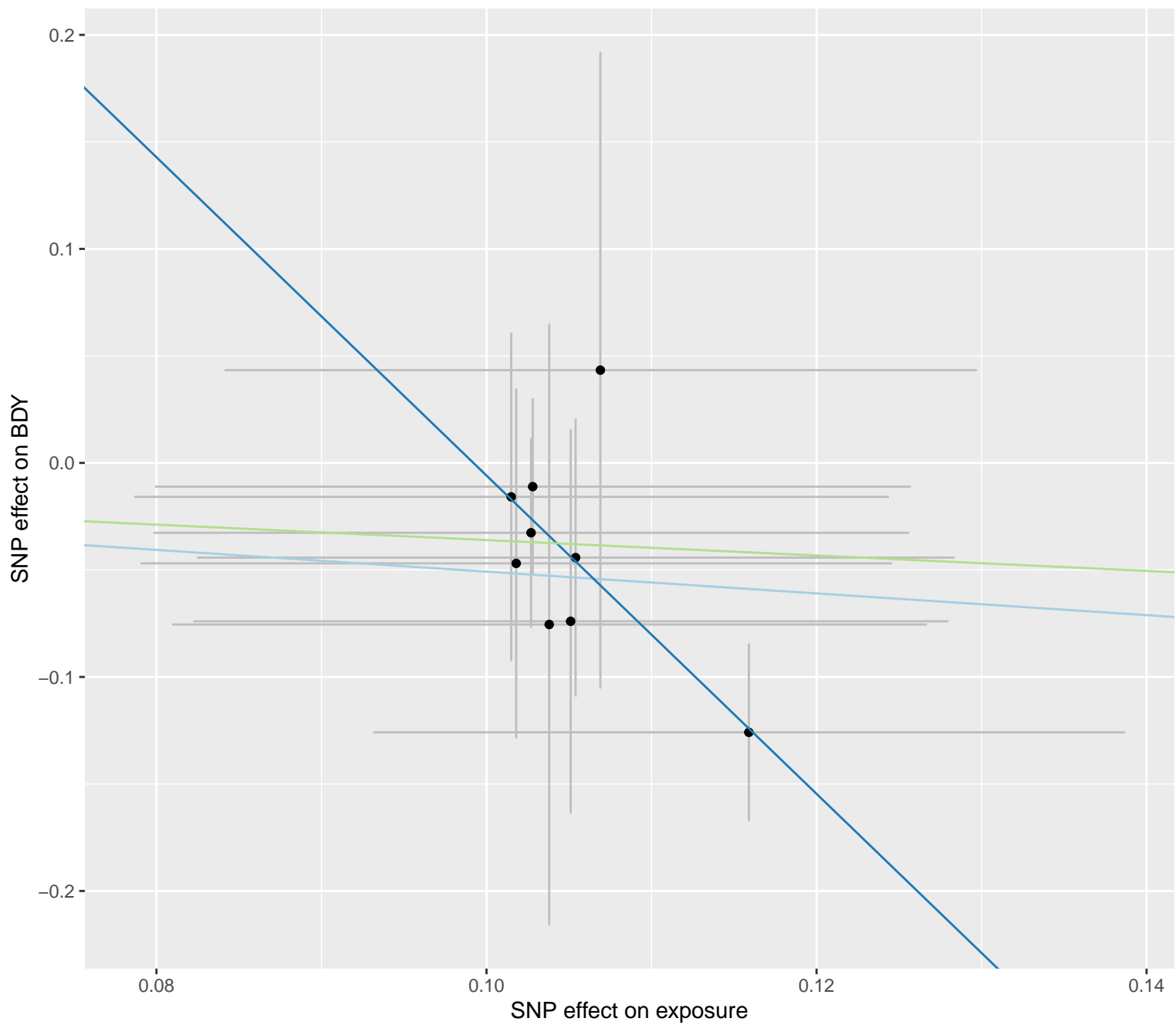

Supplement: Supplementary file 1 [file Data_Sheet_1.zip › Supplementary Materials/MR plots of saliva/Bronchitis/g__unclassified_mgs_377/scatter.pdf]

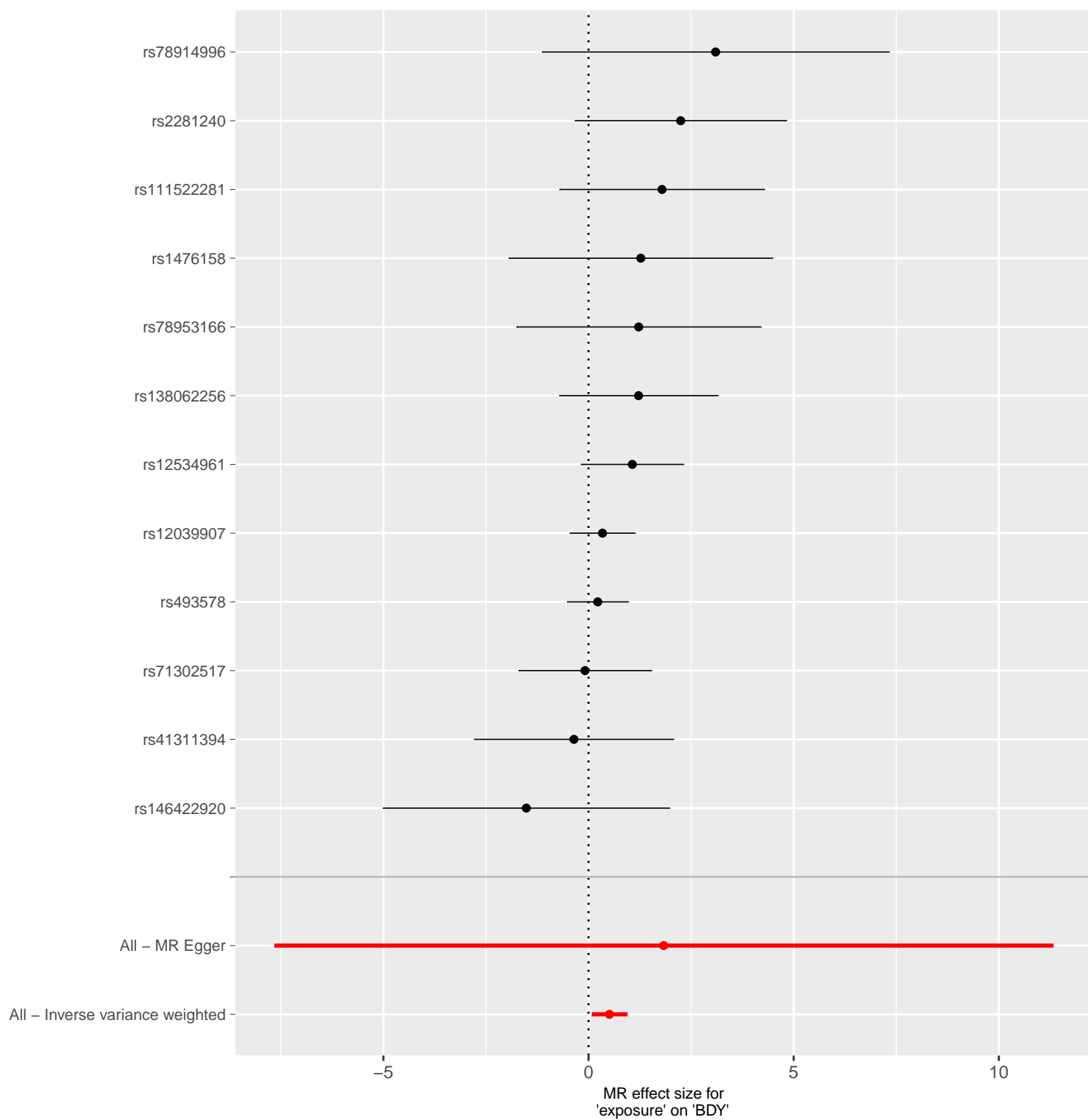

Supplement: Supplementary file 1 [file Data_Sheet_1.zip › Supplementary Materials/MR plots of saliva/Bronchitis/g__unclassified_mgs_947/forest.pdf]

# MR Method

- Inverse variance weighted
- MR Egger

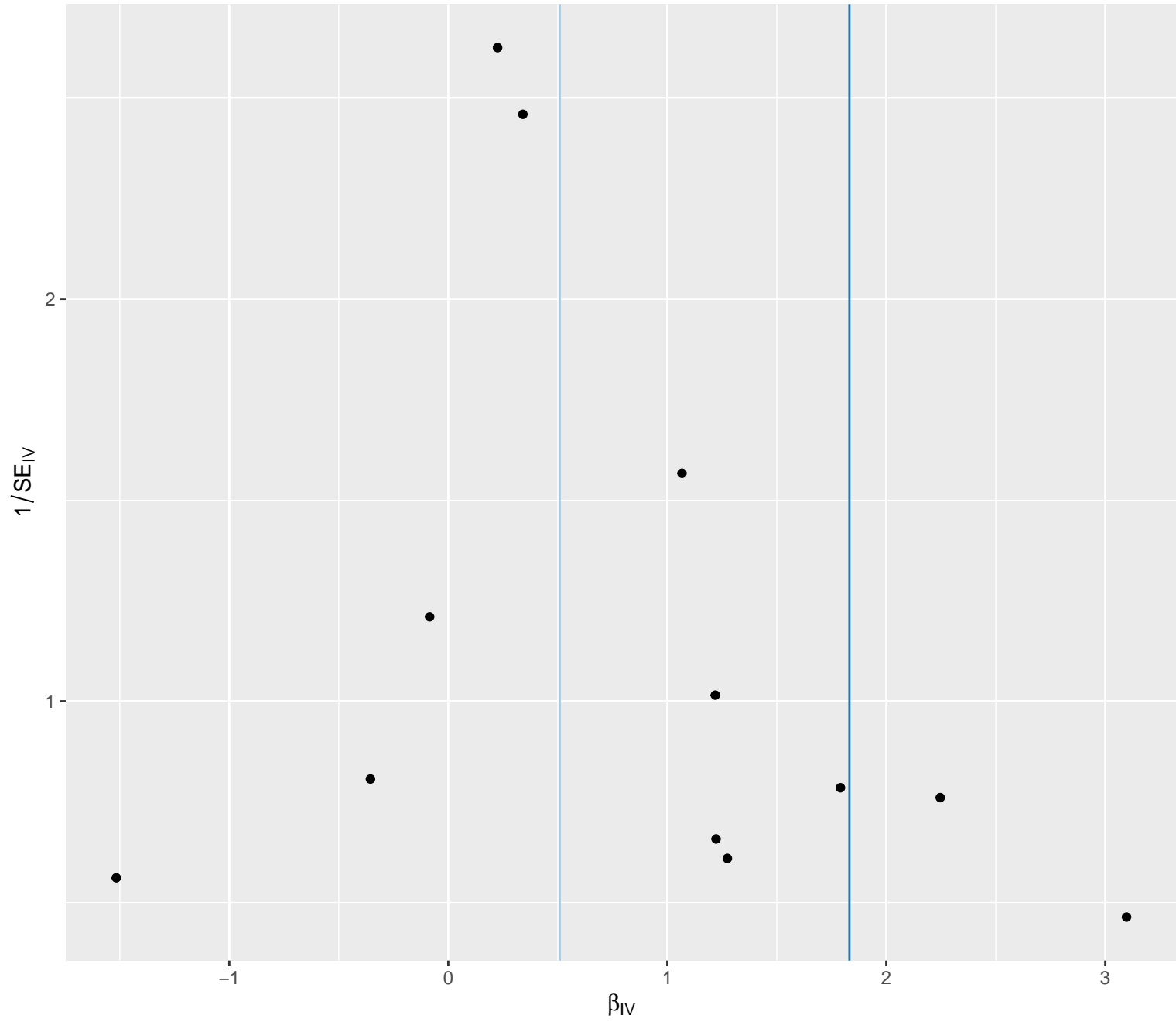

Supplement: Supplementary file 1 [file Data_Sheet_1.zip › Supplementary Materials/MR plots of saliva/Bronchitis/g__unclassified_mgs_947/funnel.pdf]

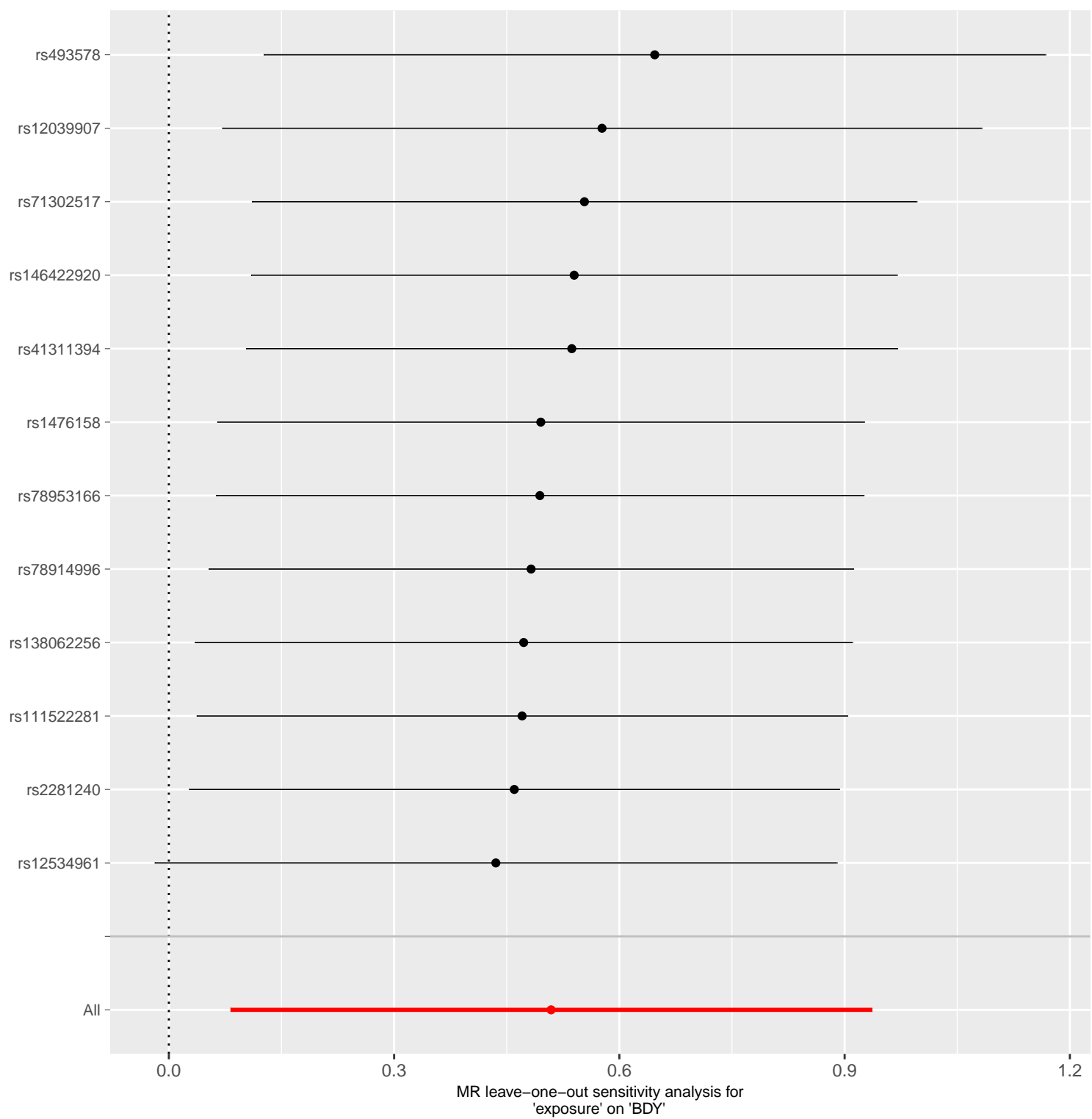

Supplement: Supplementary file 1 [file Data_Sheet_1.zip › Supplementary Materials/MR plots of saliva/Bronchitis/g__unclassified_mgs_947/leave_one_out.pdf]

# MR Test

- Inverse variance weighted
- MR Egger
- Weighted median

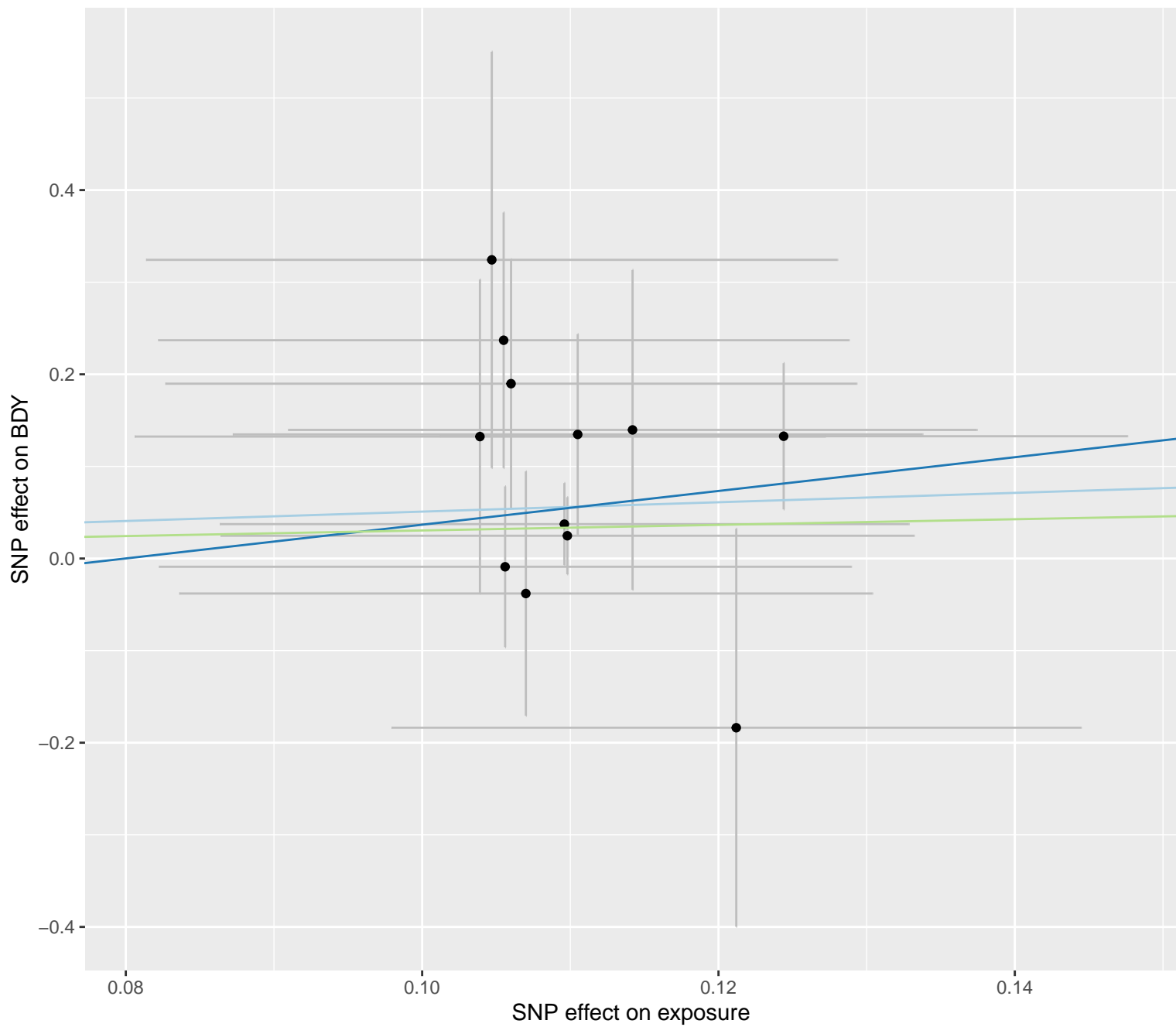

Supplement: Supplementary file 1 [file Data_Sheet_1.zip › Supplementary Materials/MR plots of saliva/Bronchitis/g__unclassified_mgs_947/scatter.pdf]

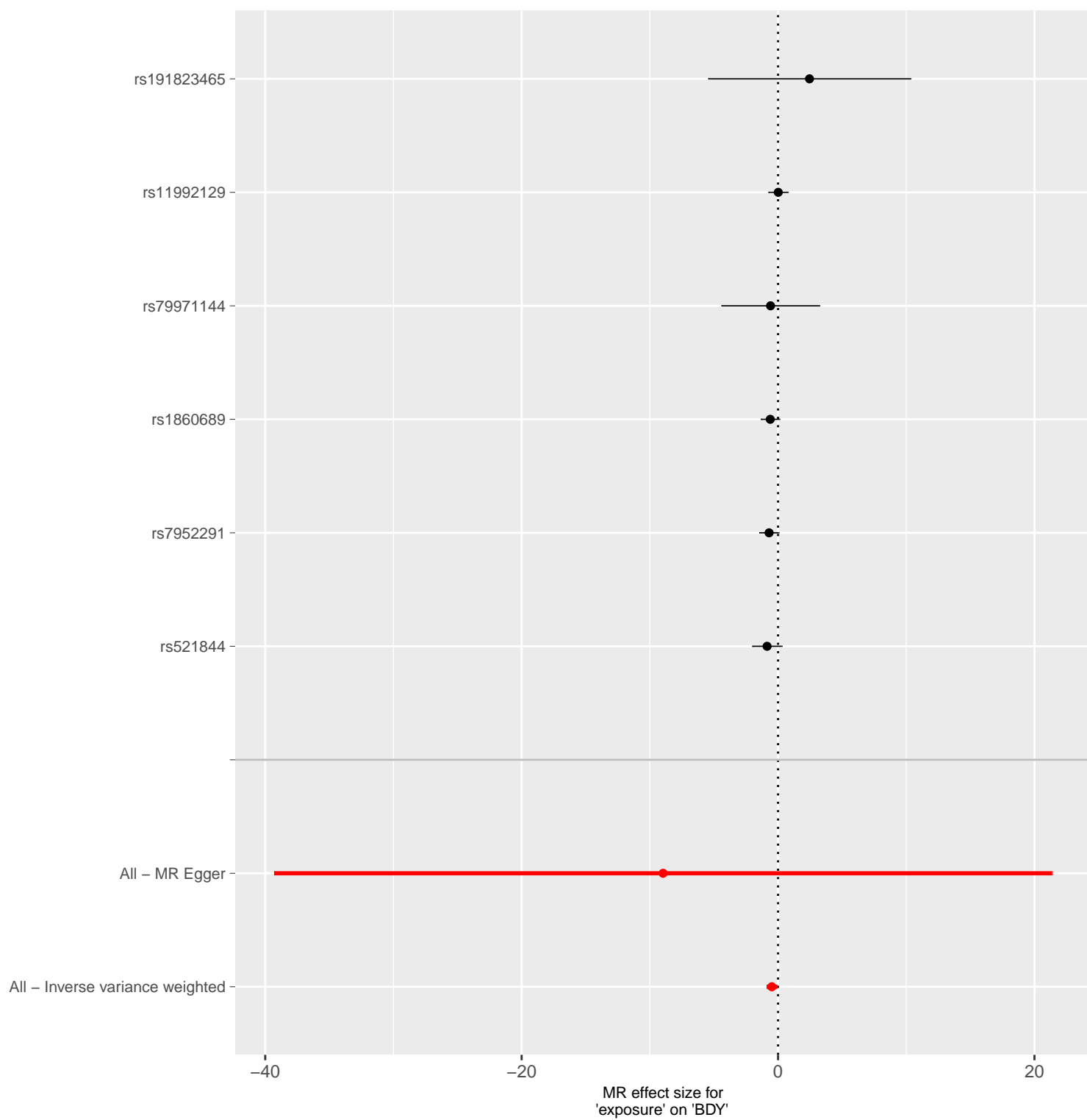

Supplement: Supplementary file 1 [file Data_Sheet_1.zip › Supplementary Materials/MR plots of saliva/Bronchitis/s__Capnocytophaga_ochracea_mgs_2502/forest.pdf]

# MR Method

- Inverse variance weighted
- MR Egger

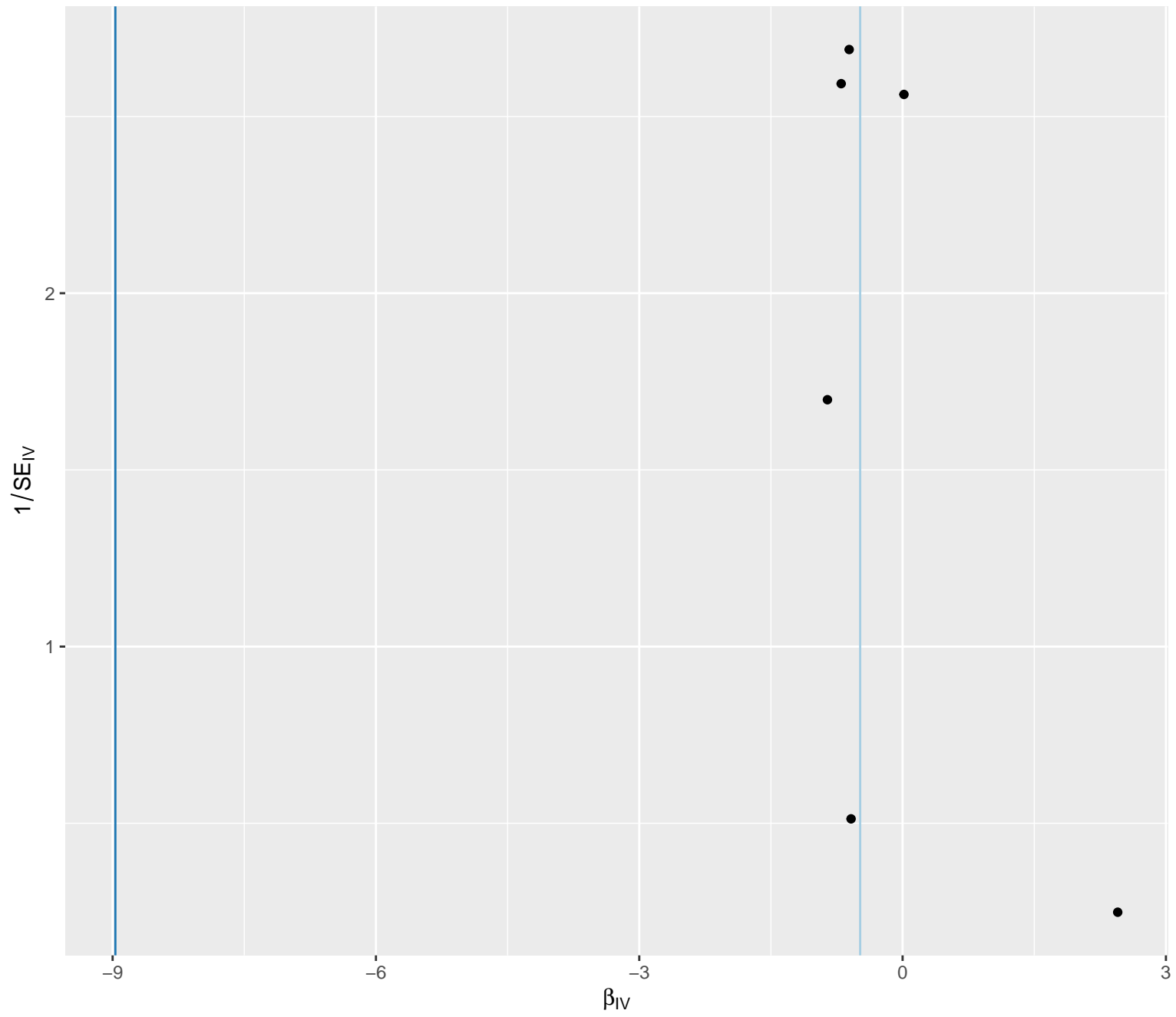

Supplement: Supplementary file 1 [file Data_Sheet_1.zip › Supplementary Materials/MR plots of saliva/Bronchitis/s__Capnocytophaga_ochracea_mgs_2502/funnel.pdf]

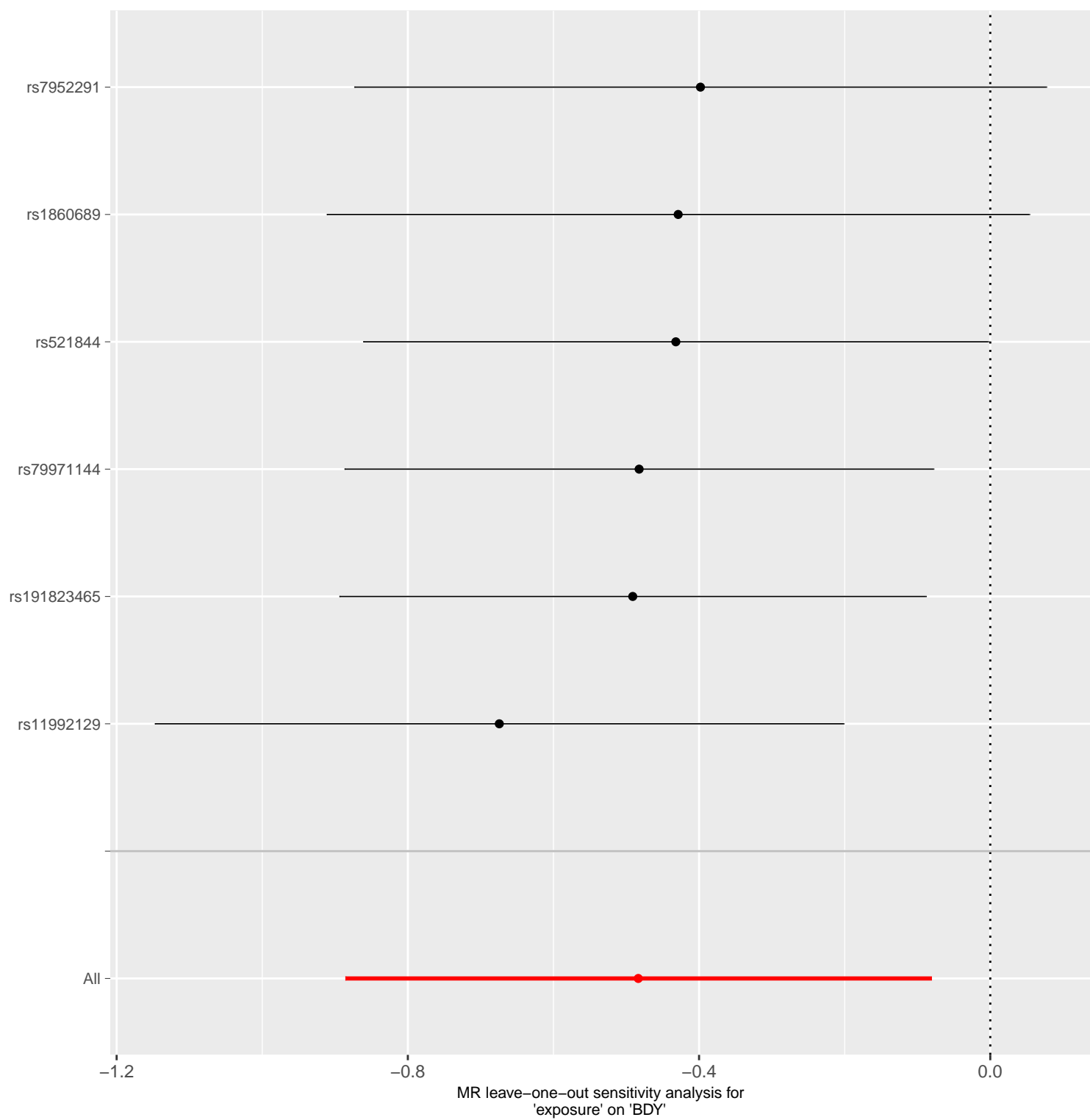

Supplement: Supplementary file 1 [file Data_Sheet_1.zip › Supplementary Materials/MR plots of saliva/Bronchitis/s__Capnocytophaga_ochracea_mgs_2502/leave_one_out.pdf]

# MR Test

- Inverse variance weighted
- MR Egger
- Weighted median

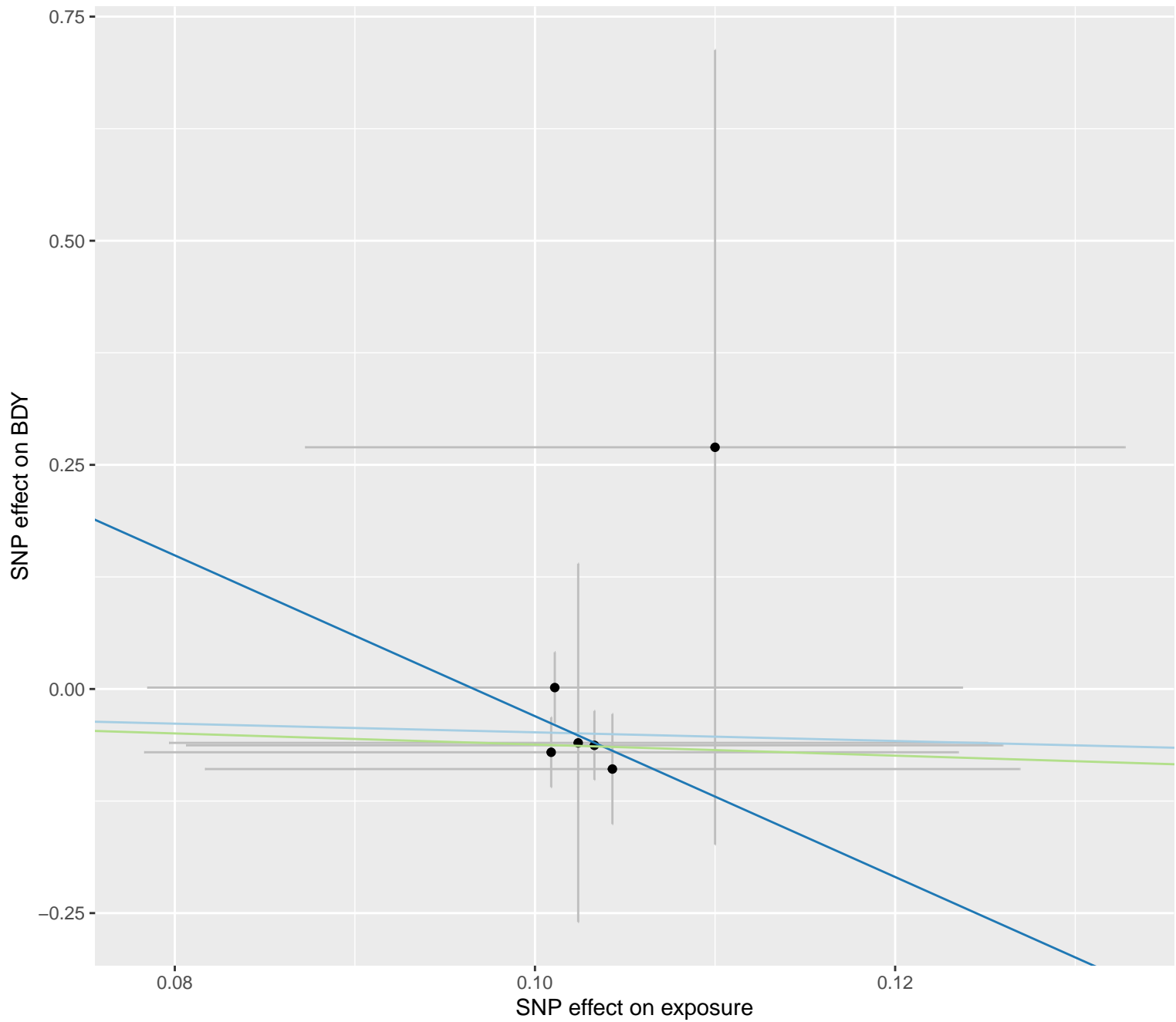

Supplement: Supplementary file 1 [file Data_Sheet_1.zip › Supplementary Materials/MR plots of saliva/Bronchitis/s__Capnocytophaga_ochracea_mgs_2502/scatter.pdf]

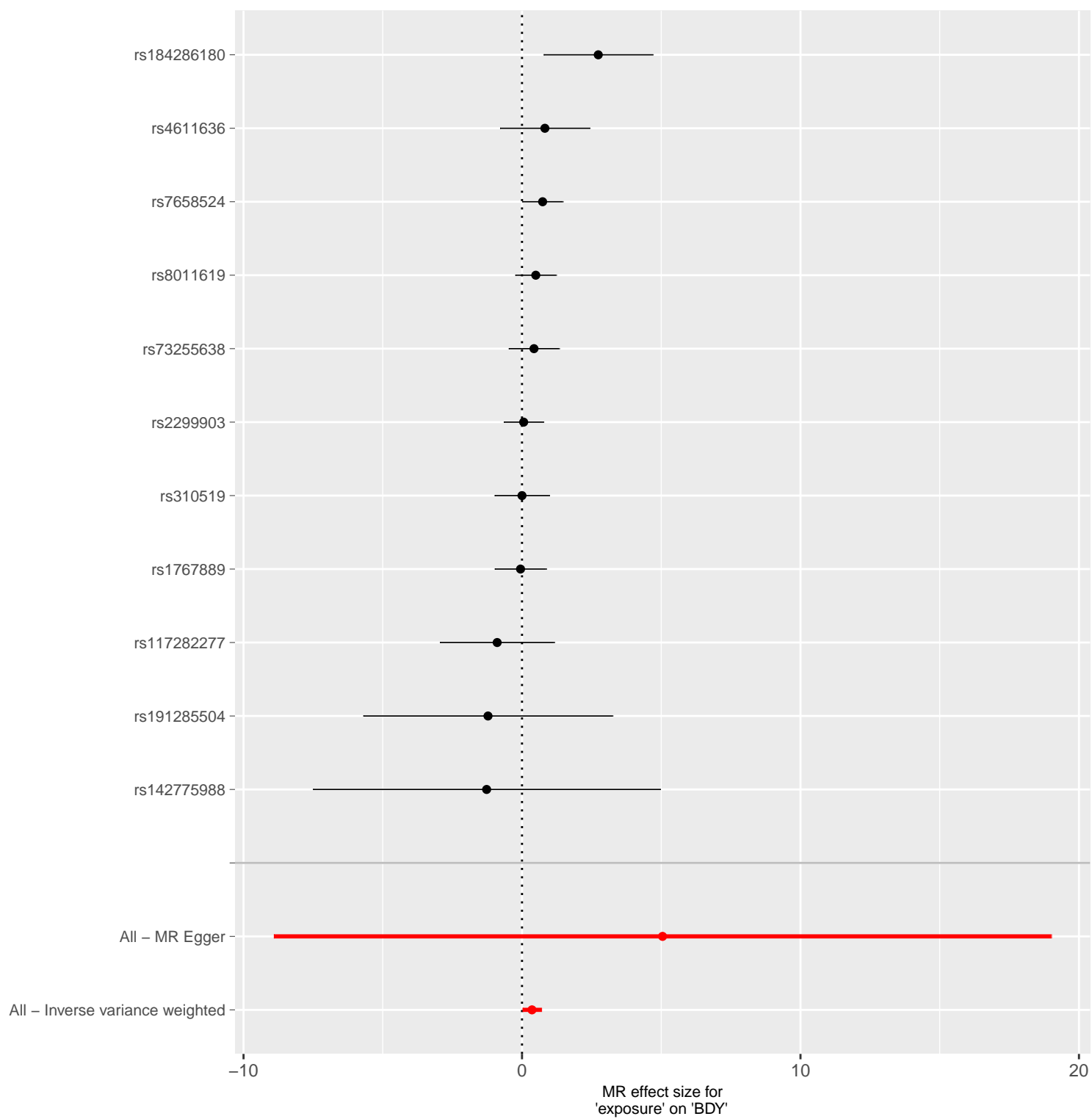

Supplement: Supplementary file 1 [file Data_Sheet_1.zip › Supplementary Materials/MR plots of saliva/Bronchitis/s__Fusobacterium_periodonticum_C_mgs_3047/forest.pdf]

# MR Method

- Inverse variance weighted
- MR Egger

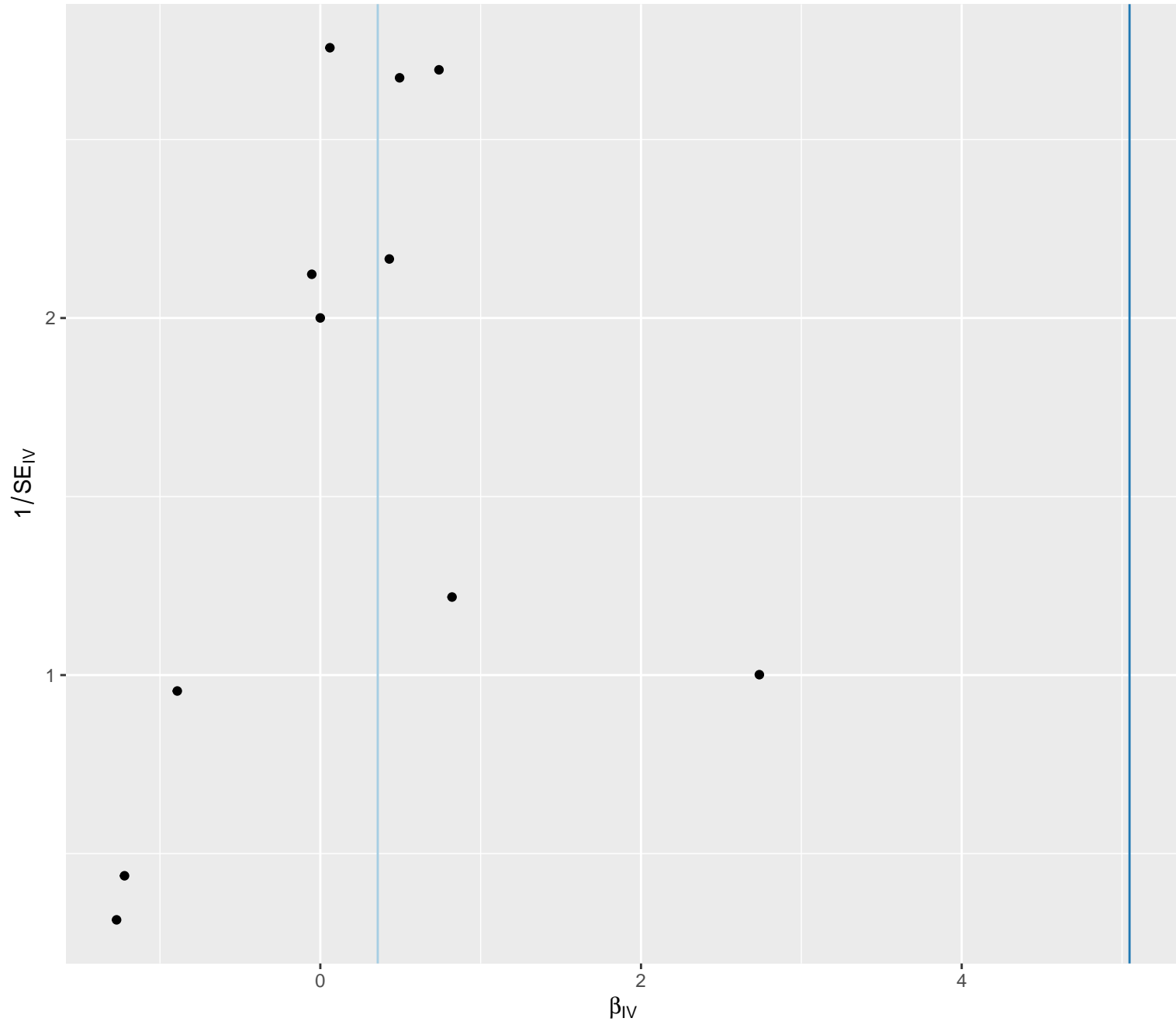

Supplement: Supplementary file 1 [file Data_Sheet_1.zip › Supplementary Materials/MR plots of saliva/Bronchitis/s__Fusobacterium_periodonticum_C_mgs_3047/funnel.pdf]

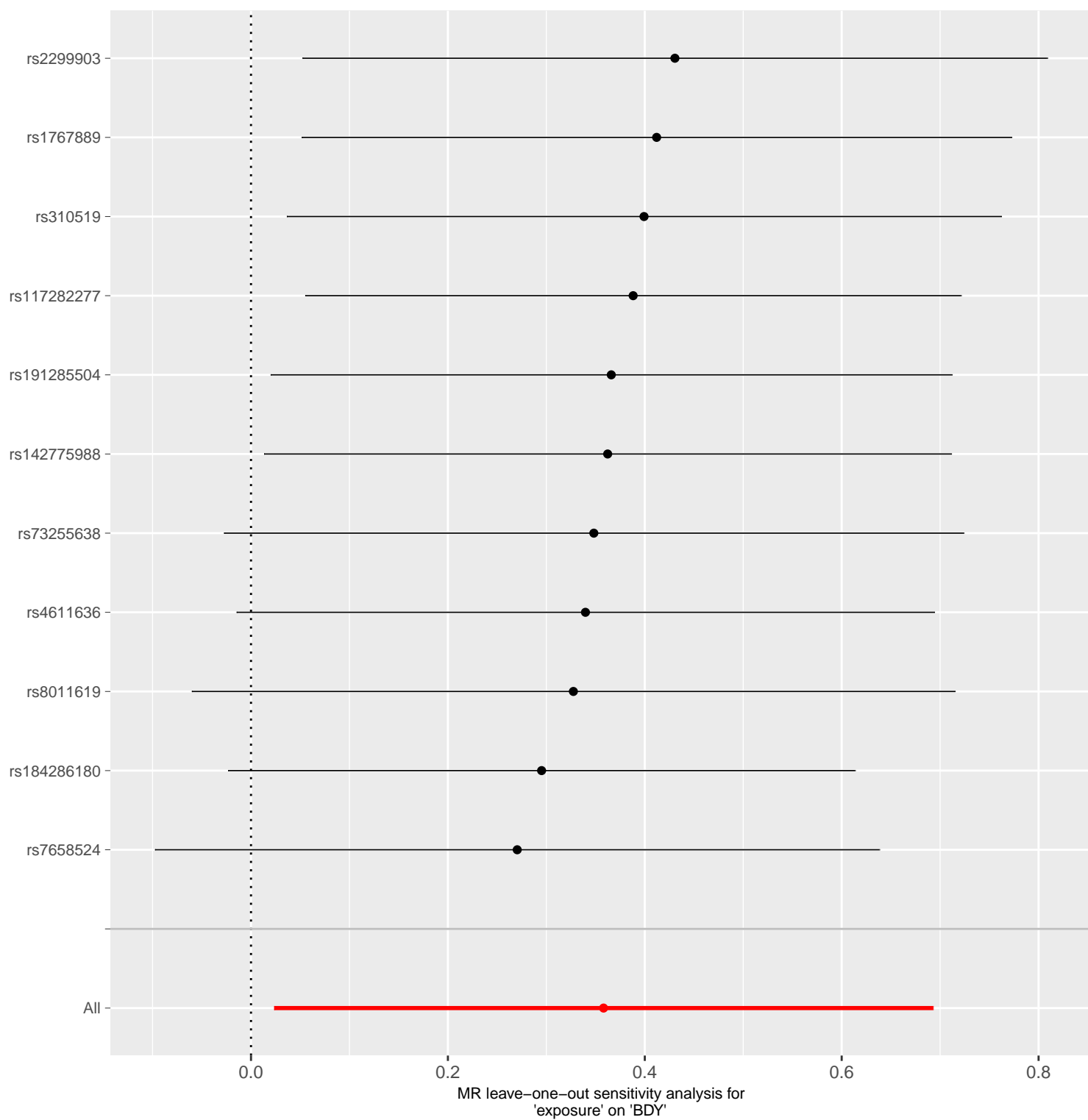

Supplement: Supplementary file 1 [file Data_Sheet_1.zip › Supplementary Materials/MR plots of saliva/Bronchitis/s__Fusobacterium_periodonticum_C_mgs_3047/leave_one_out.pdf]

# MR Test

- Inverse variance weighted
- MR Egger
- Weighted median

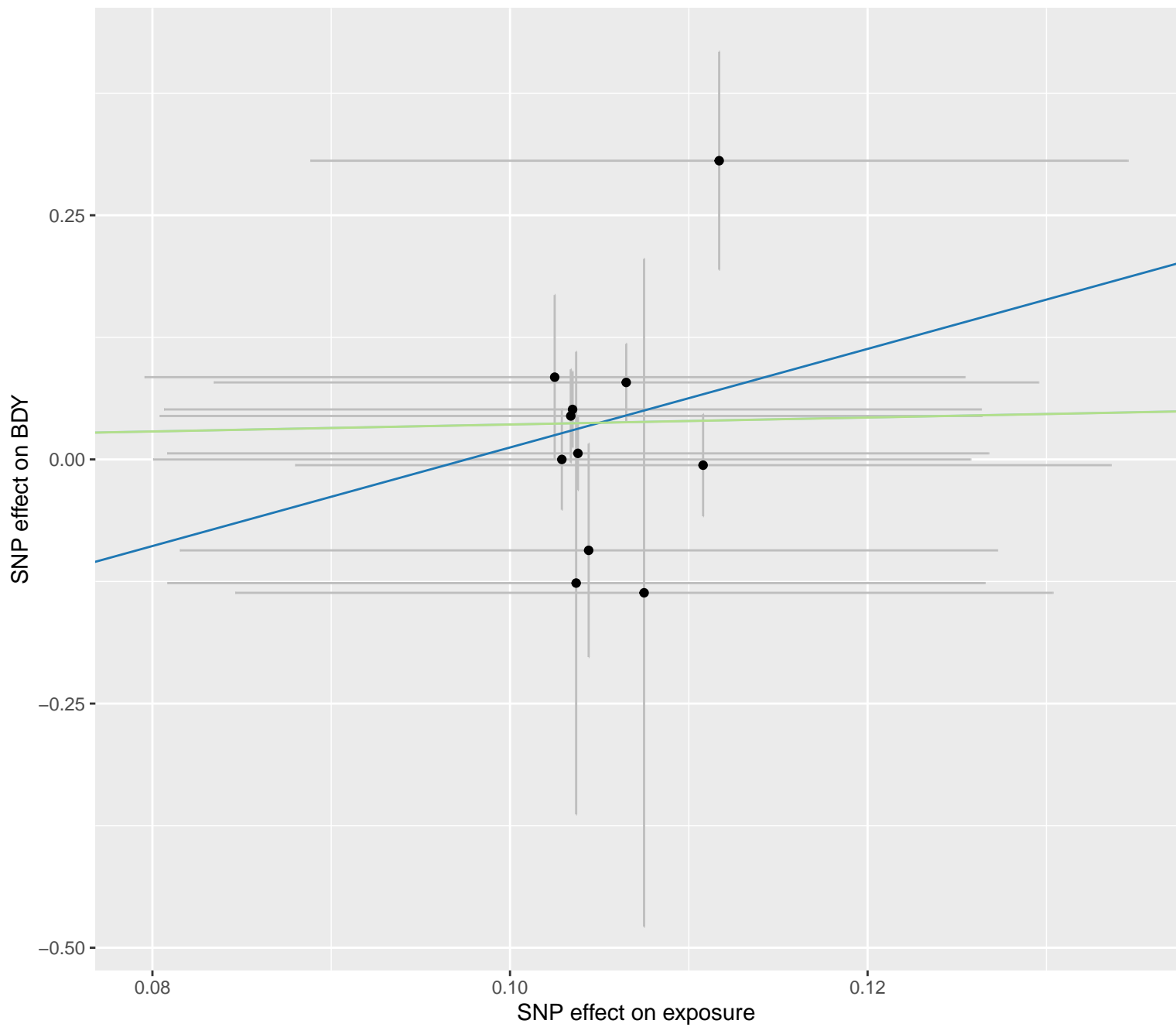

Supplement: Supplementary file 1 [file Data_Sheet_1.zip › Supplementary Materials/MR plots of saliva/Bronchitis/s__Fusobacterium_periodonticum_C_mgs_3047/scatter.pdf]

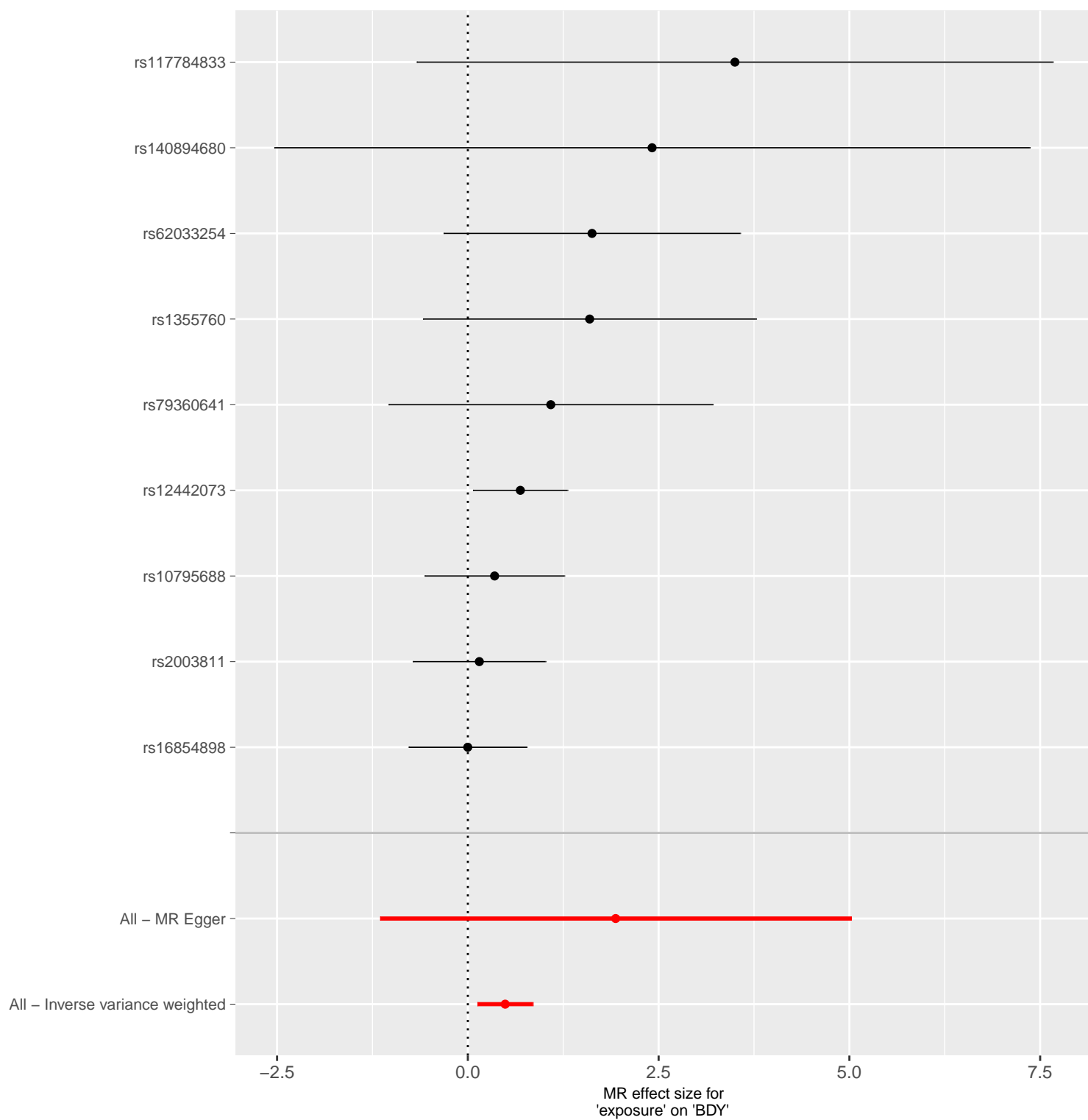

Supplement: Supplementary file 1 [file Data_Sheet_1.zip › Supplementary Materials/MR plots of saliva/Bronchitis/s__Gemella_haemolysans_B_mgs_2903/forest.pdf]

# MR Method

- Inverse variance weighted
- MR Egger

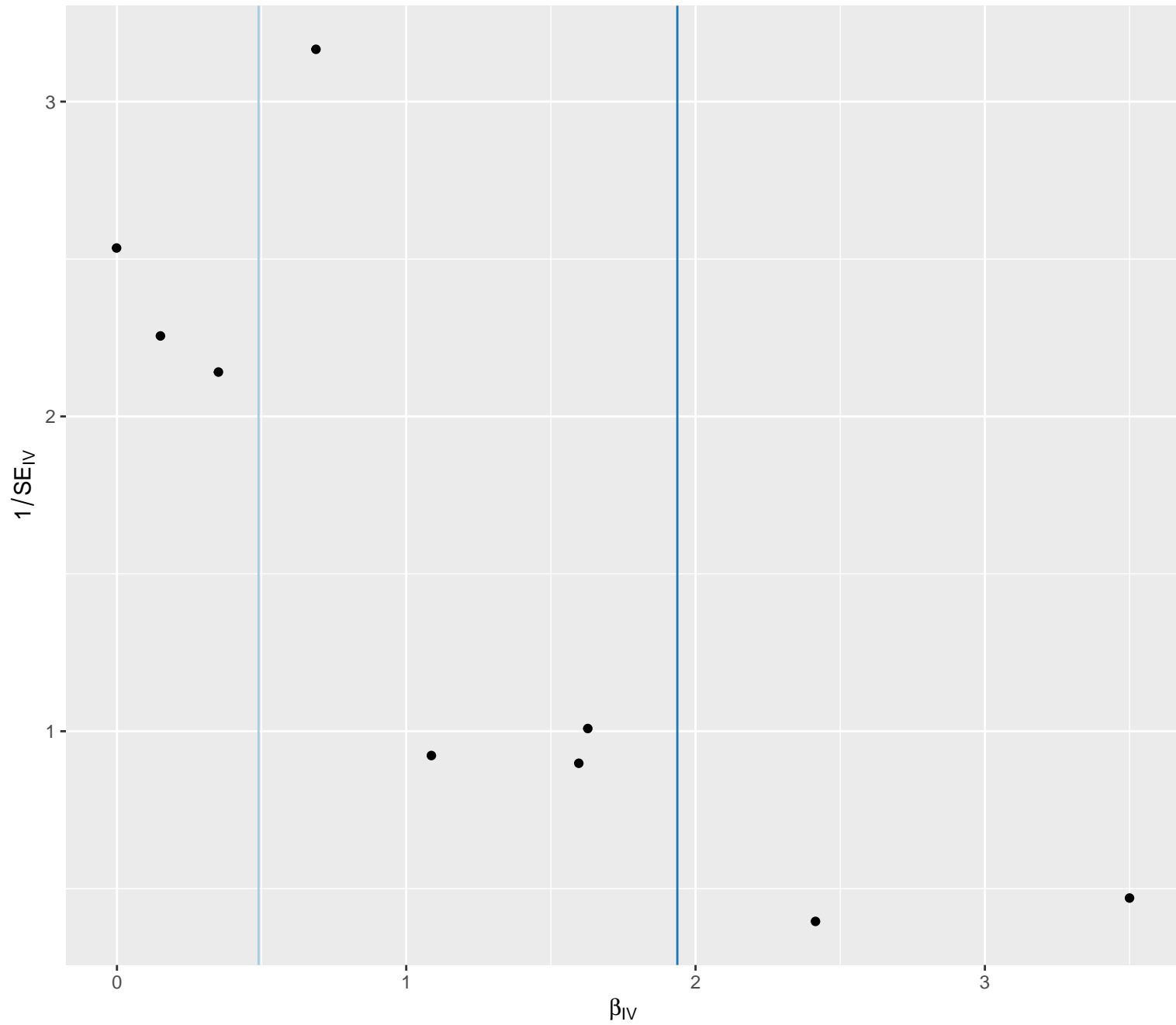

Supplement: Supplementary file 1 [file Data_Sheet_1.zip › Supplementary Materials/MR plots of saliva/Bronchitis/s__Gemella_haemolysans_B_mgs_2903/funnel.pdf]

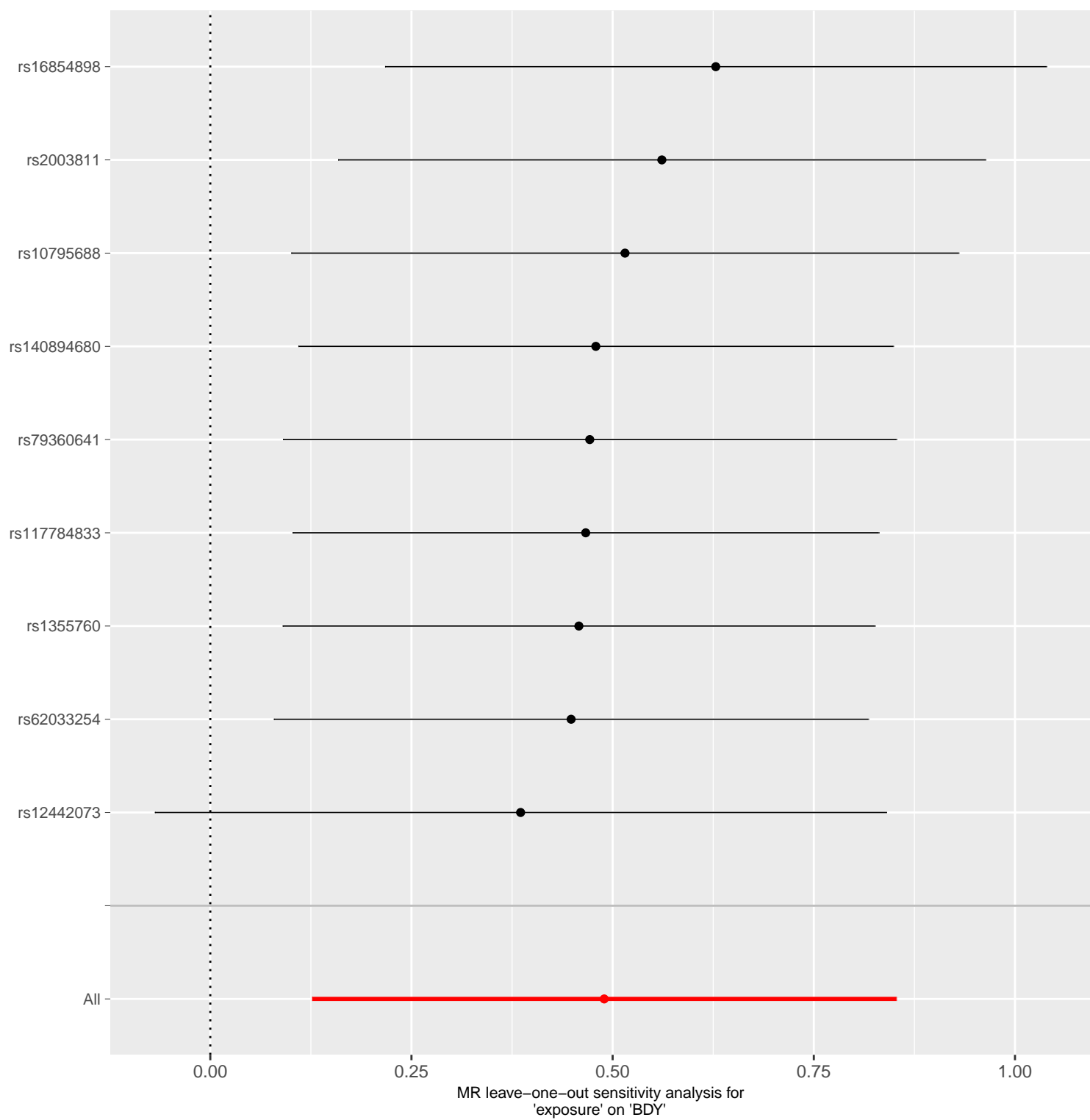

Supplement: Supplementary file 1 [file Data_Sheet_1.zip › Supplementary Materials/MR plots of saliva/Bronchitis/s__Gemella_haemolysans_B_mgs_2903/leave_one_out.pdf]

# MR Test

- Inverse variance weighted
- MR Egger
- Weighted median

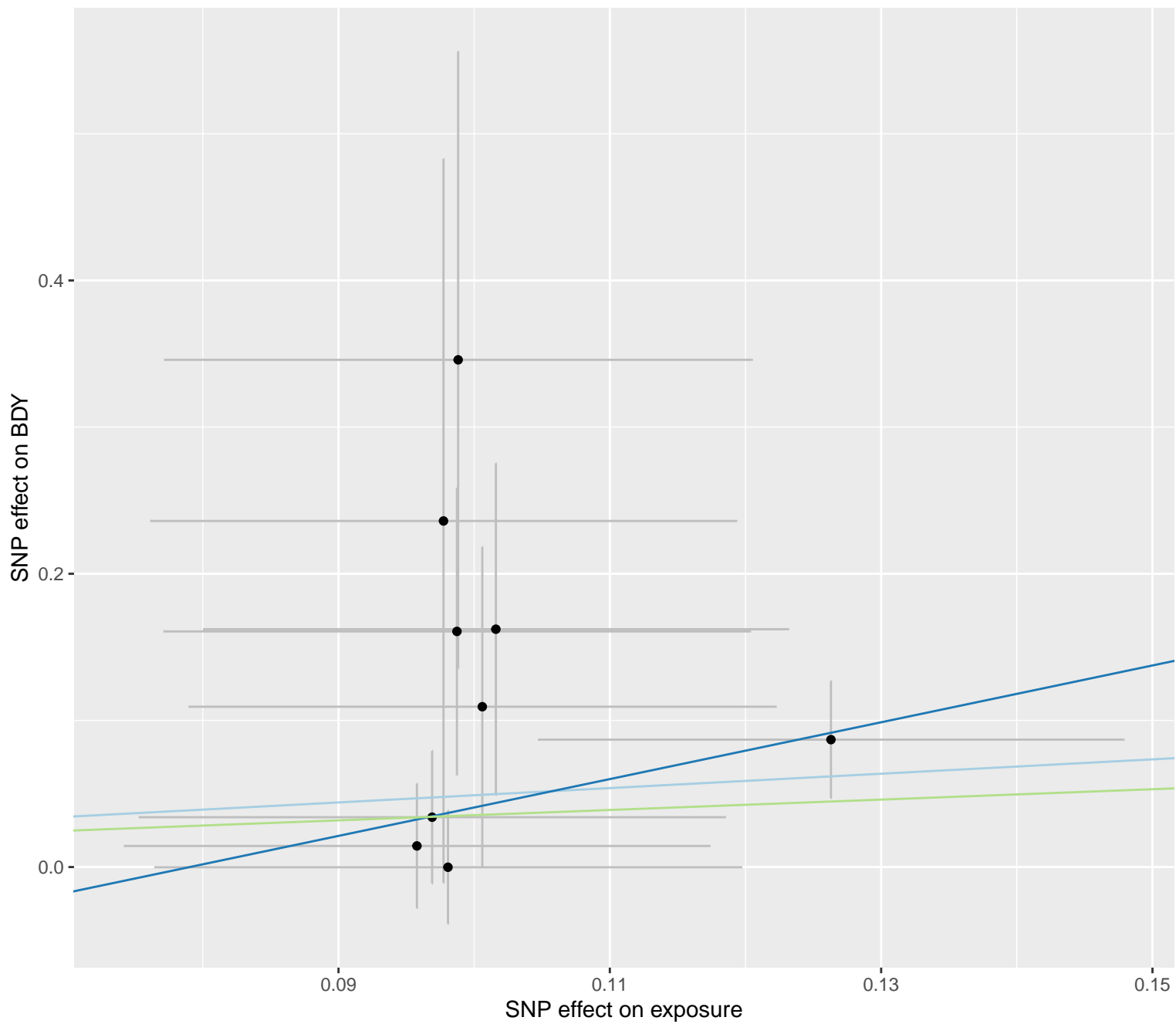

Supplement: Supplementary file 1 [file Data_Sheet_1.zip › Supplementary Materials/MR plots of saliva/Bronchitis/s__Gemella_haemolysans_B_mgs_2903/scatter.pdf]

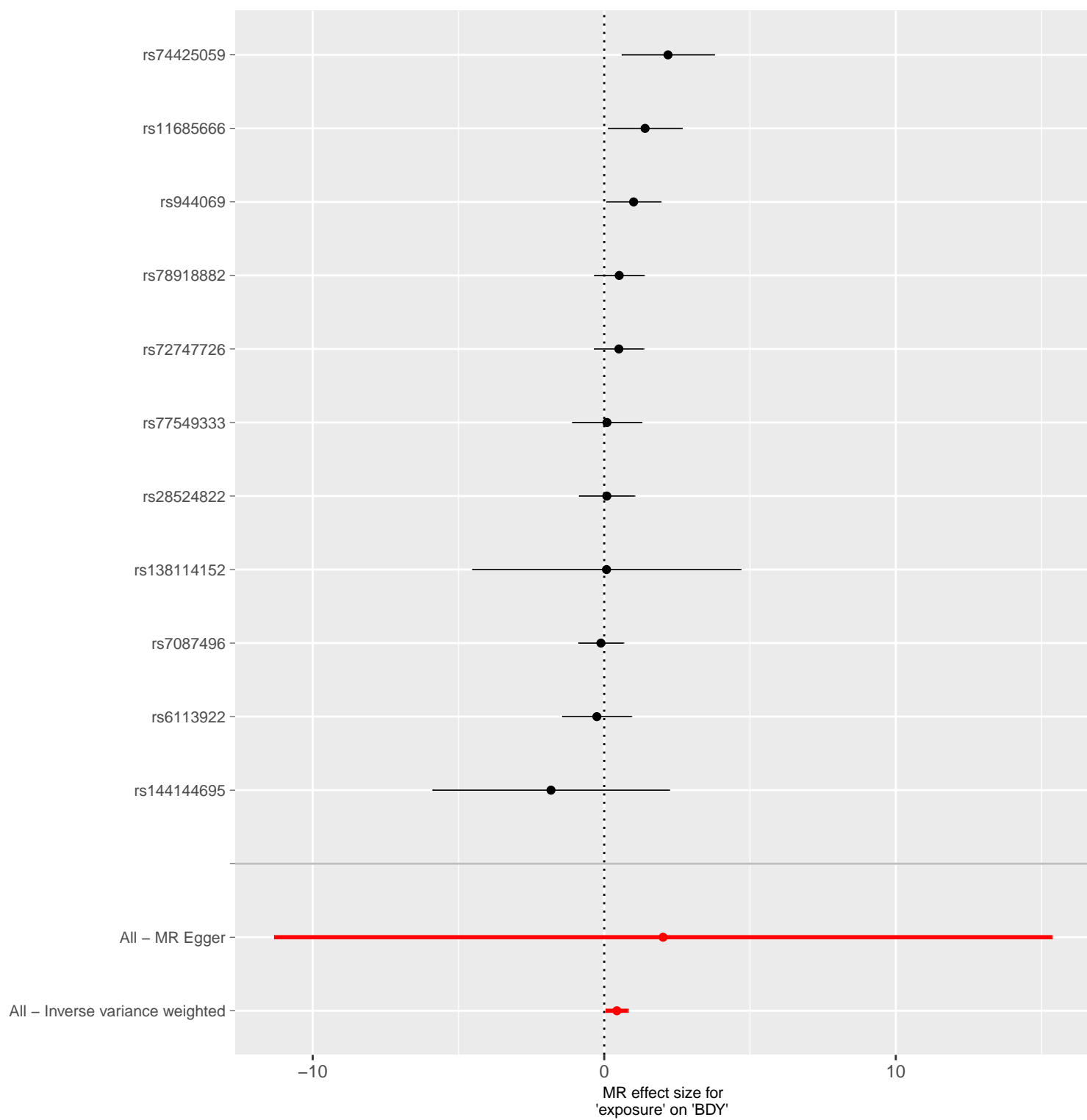

Supplement: Supplementary file 1 [file Data_Sheet_1.zip › Supplementary Materials/MR plots of saliva/Bronchitis/s__Porphyromonas_gingivalis_mgs_77/forest.pdf]

# MR Method

- Inverse variance weighted
- MR Egger

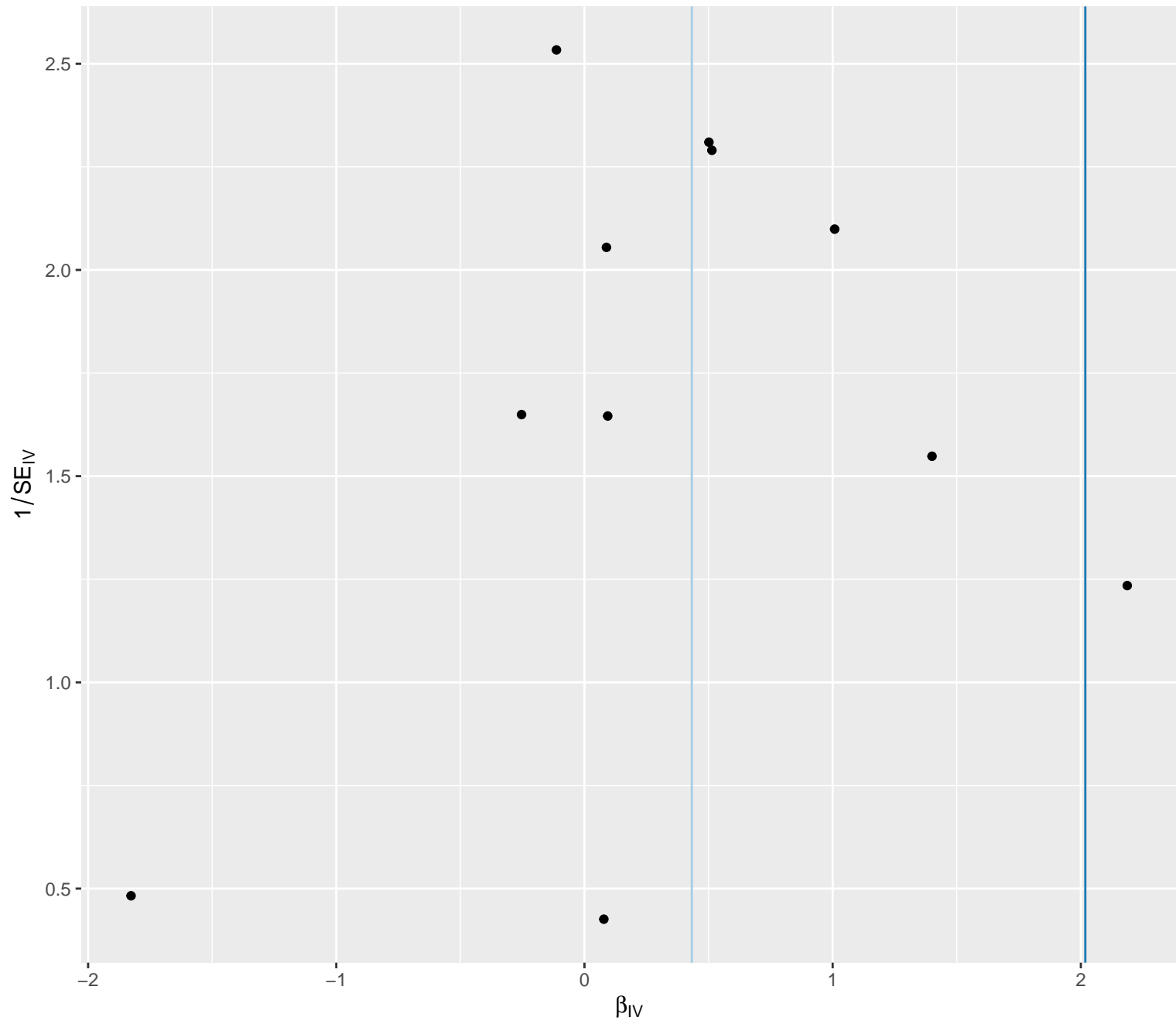

Supplement: Supplementary file 1 [file Data_Sheet_1.zip › Supplementary Materials/MR plots of saliva/Bronchitis/s__Porphyromonas_gingivalis_mgs_77/funnel.pdf]

# MR Test

- Inverse variance weighted
- MR Egger
- Weighted median

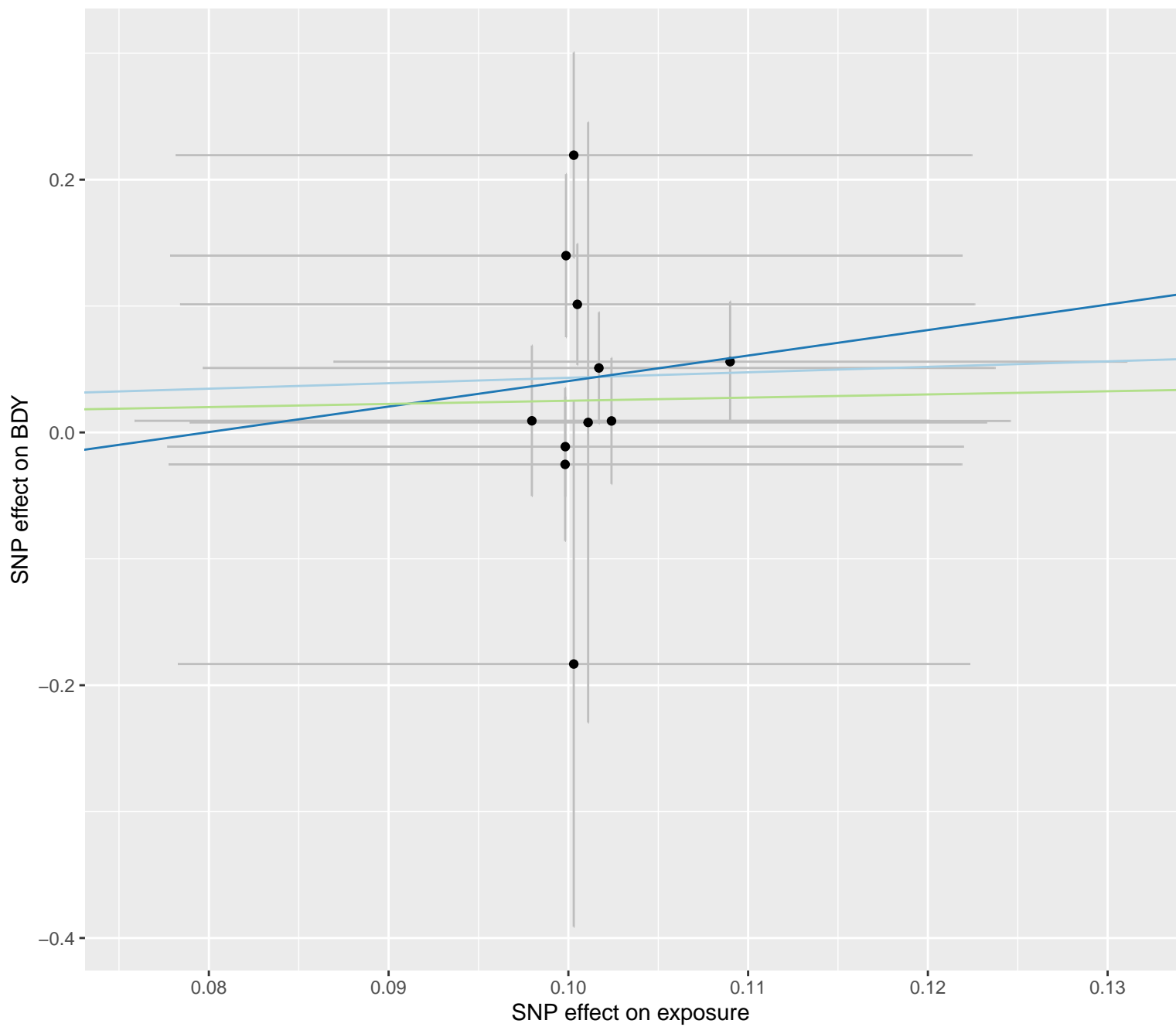

Supplement: Supplementary file 1 [file Data_Sheet_1.zip › Supplementary Materials/MR plots of saliva/Bronchitis/s__Porphyromonas_gingivalis_mgs_77/scatter.pdf]

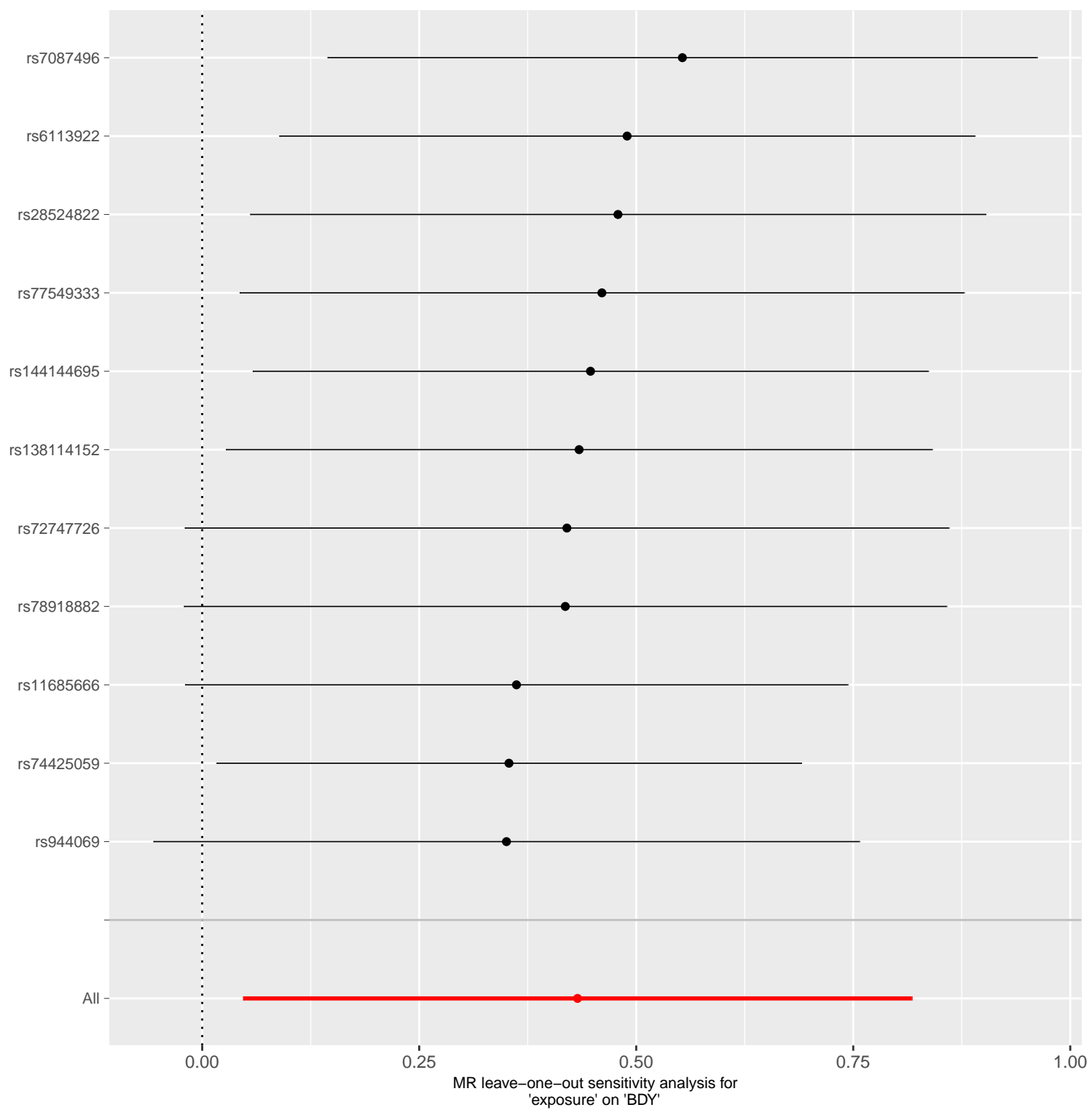

Supplement: Supplementary file 1 [file Data_Sheet_1.zip › Supplementary Materials/MR plots of saliva/Bronchitis/s__Porphyromonas_gingivalis_mgs_77/sensitivity analysis.pdf]
