# Supplementary figures and images for: Association between oral microbiome and five types of respiratory infections: a two-sample Mendelian randomization study in east Asian population (part 1 of 2)
Source: Front Microbiol. 2024 Apr 10;15:1392473. doi: 10.3389/fmicb.2024.1392473 (PMC11039966; doi:10.3389/fmicb.2024.1392473)

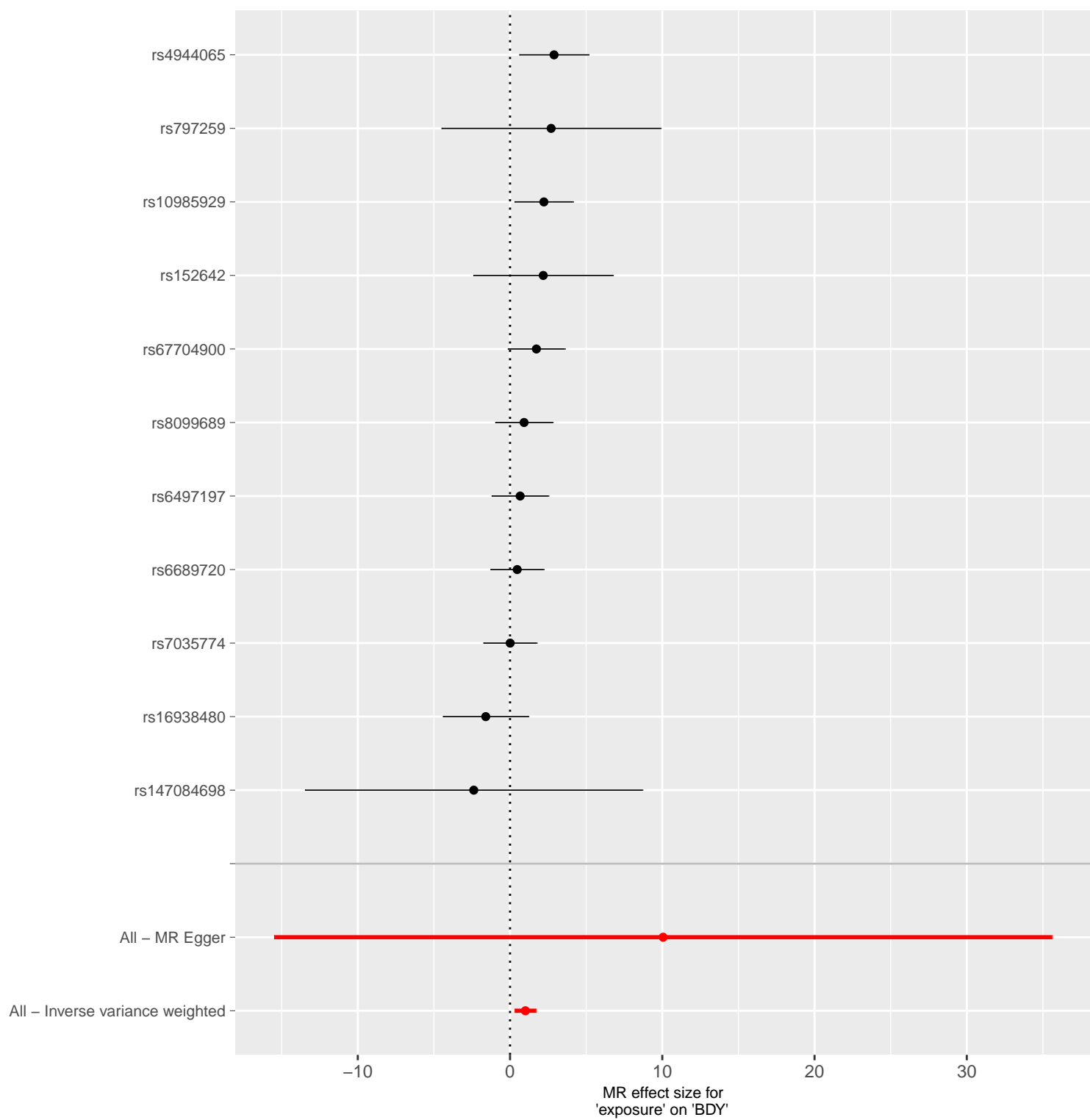

Supplement: Supplementary file 1 [file Data_Sheet_1.zip › Supplementary Materials/MR plots for tongue/Bronchiectasis/s__Eikenella_sp001648475_mgs_3302/forest.pdf]

# MR Method

- Inverse variance weighted
- MR Egger

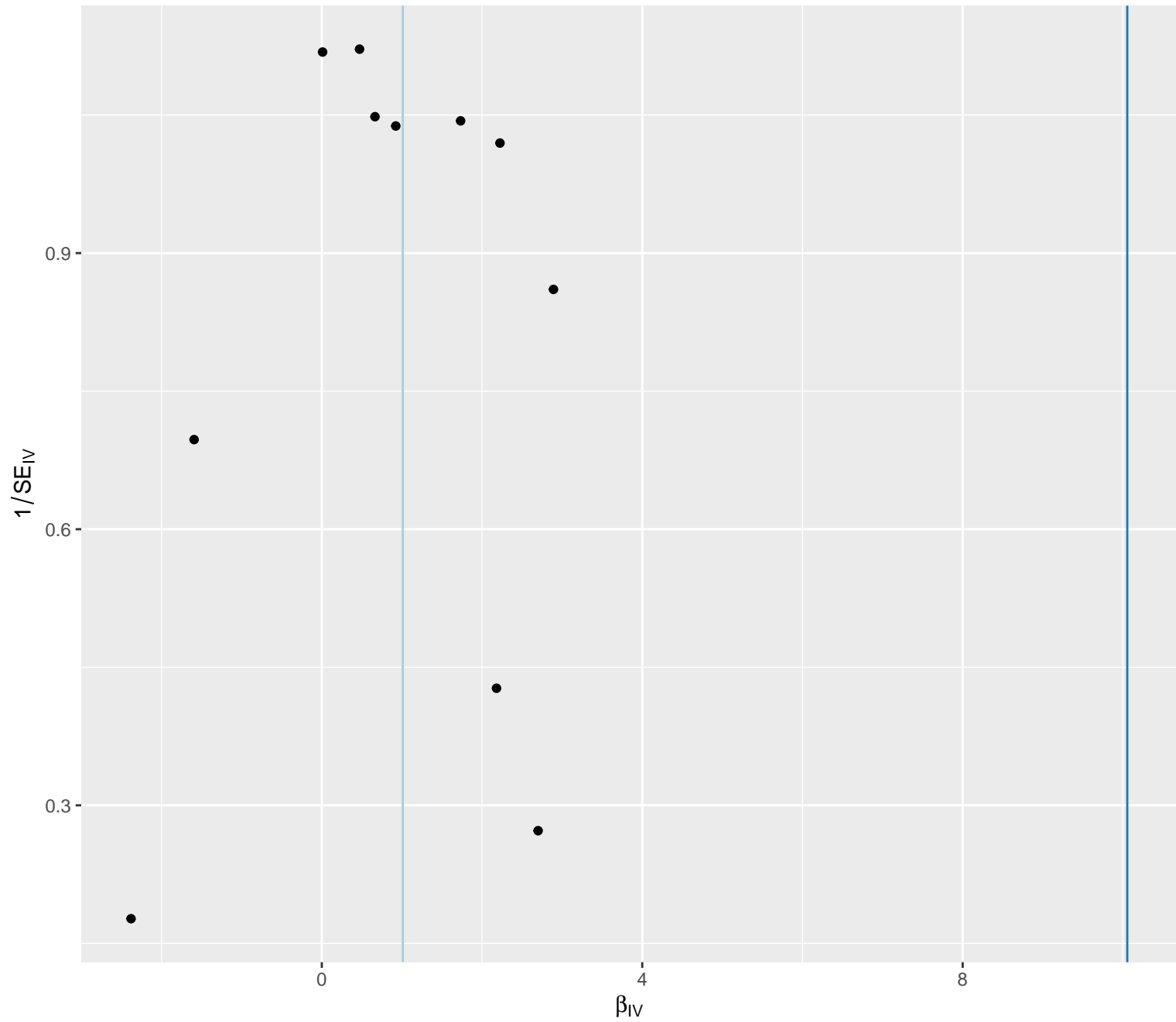

Supplement: Supplementary file 1 [file Data_Sheet_1.zip › Supplementary Materials/MR plots for tongue/Bronchiectasis/s__Eikenella_sp001648475_mgs_3302/funnel.pdf]

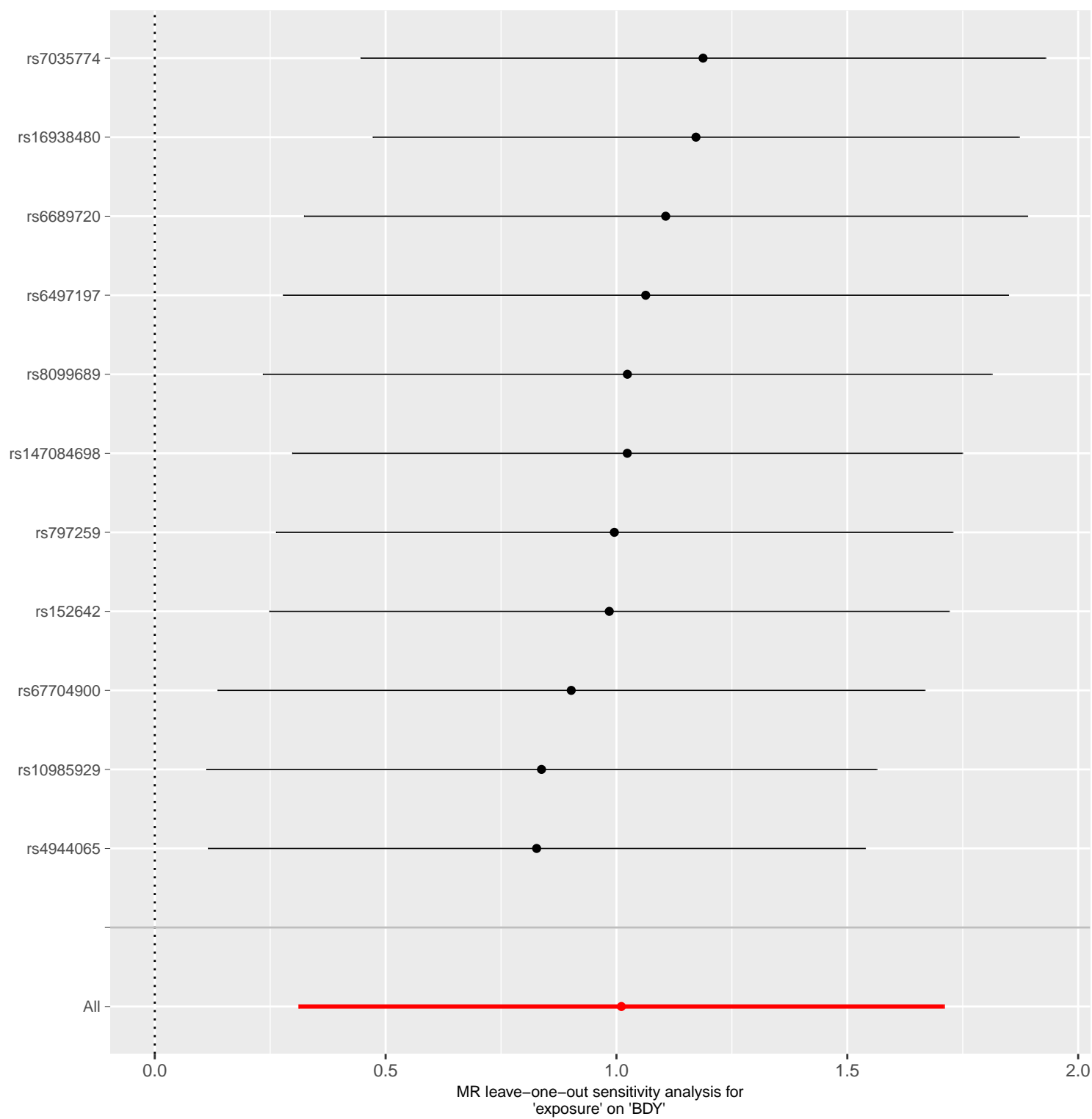

Supplement: Supplementary file 1 [file Data_Sheet_1.zip › Supplementary Materials/MR plots for tongue/Bronchiectasis/s__Eikenella_sp001648475_mgs_3302/leave_one_out.pdf]

# MR Test

- Inverse variance weighted
- MR Egger
- Weighted median

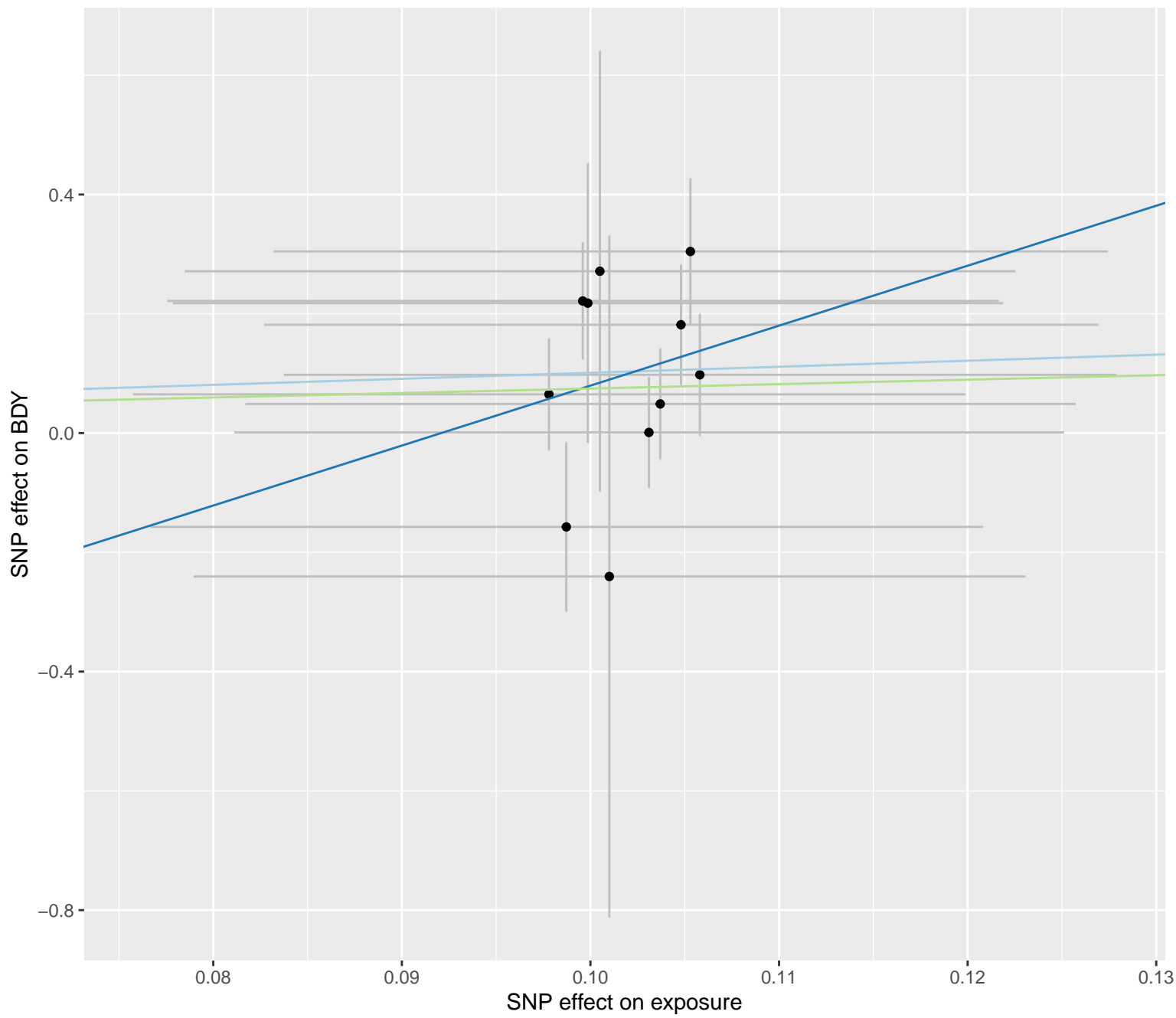

Supplement: Supplementary file 1 [file Data_Sheet_1.zip › Supplementary Materials/MR plots for tongue/Bronchiectasis/s__Eikenella_sp001648475_mgs_3302/scatter.pdf]

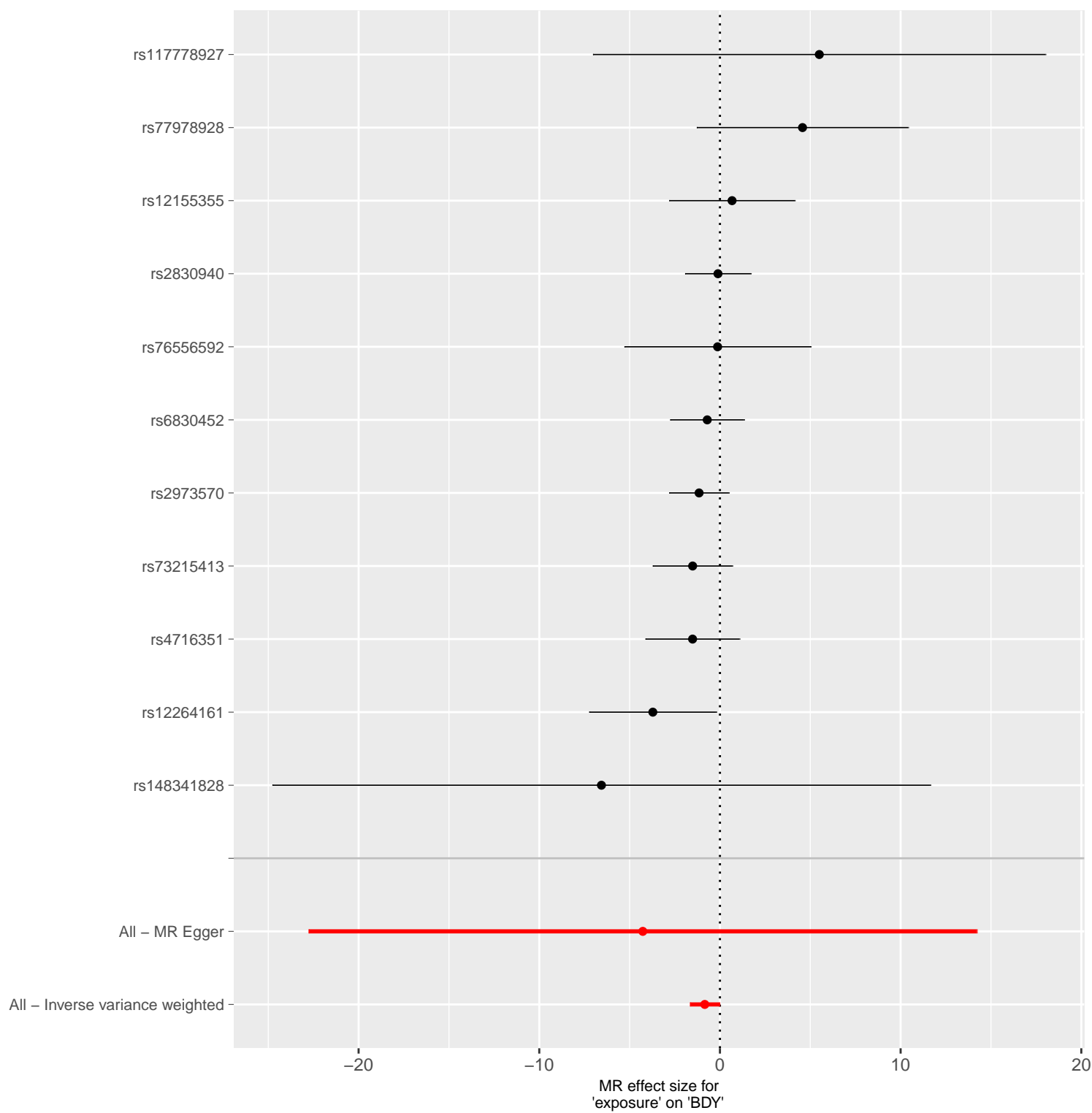

Supplement: Supplementary file 1 [file Data_Sheet_1.zip › Supplementary Materials/MR plots for tongue/Bronchiectasis/s__F0422_sp001553345_mgs_3021/forest.pdf]

# MR Method

- Inverse variance weighted
- MR Egger

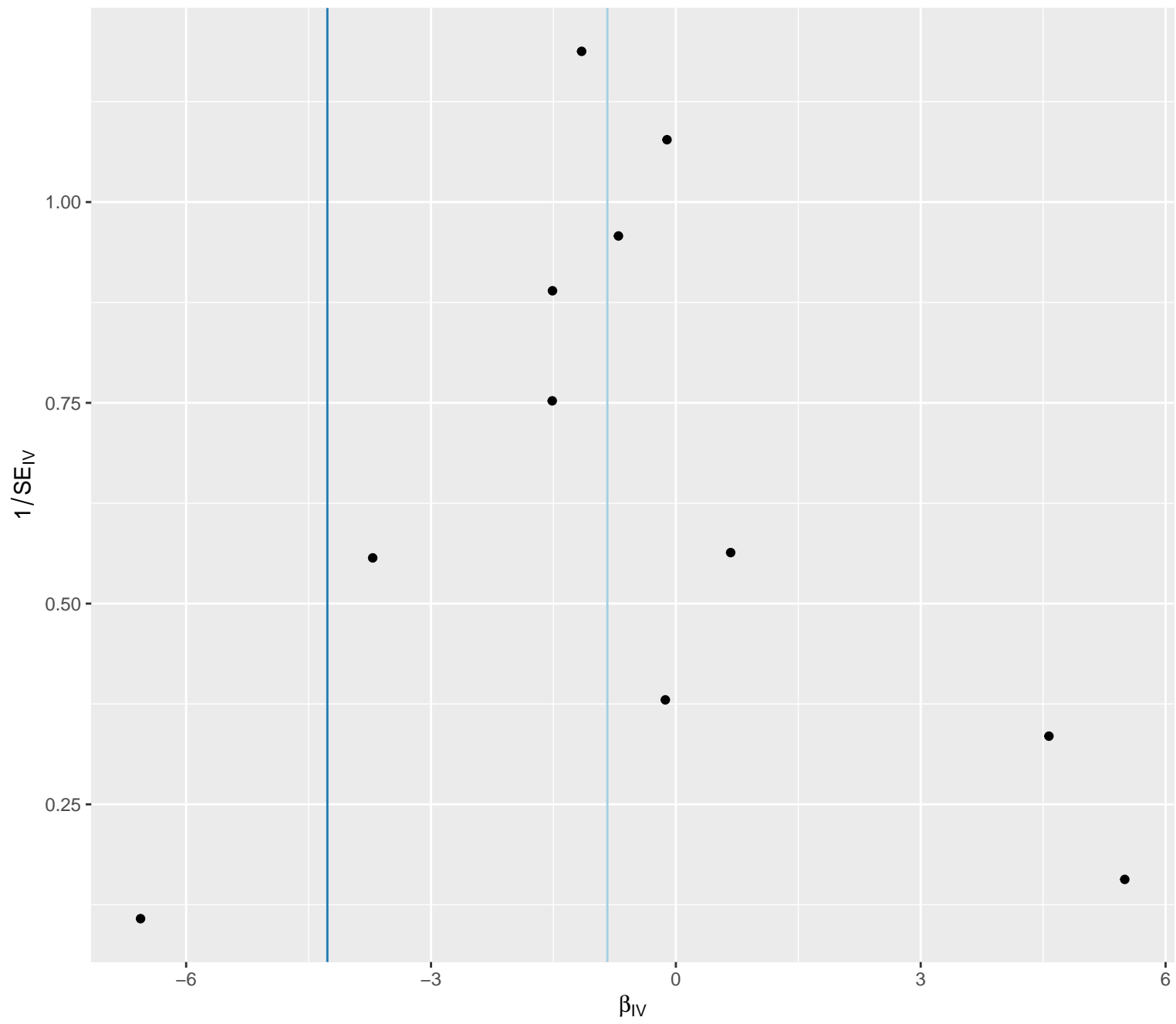

Supplement: Supplementary file 1 [file Data_Sheet_1.zip › Supplementary Materials/MR plots for tongue/Bronchiectasis/s__F0422_sp001553345_mgs_3021/funnel.pdf]

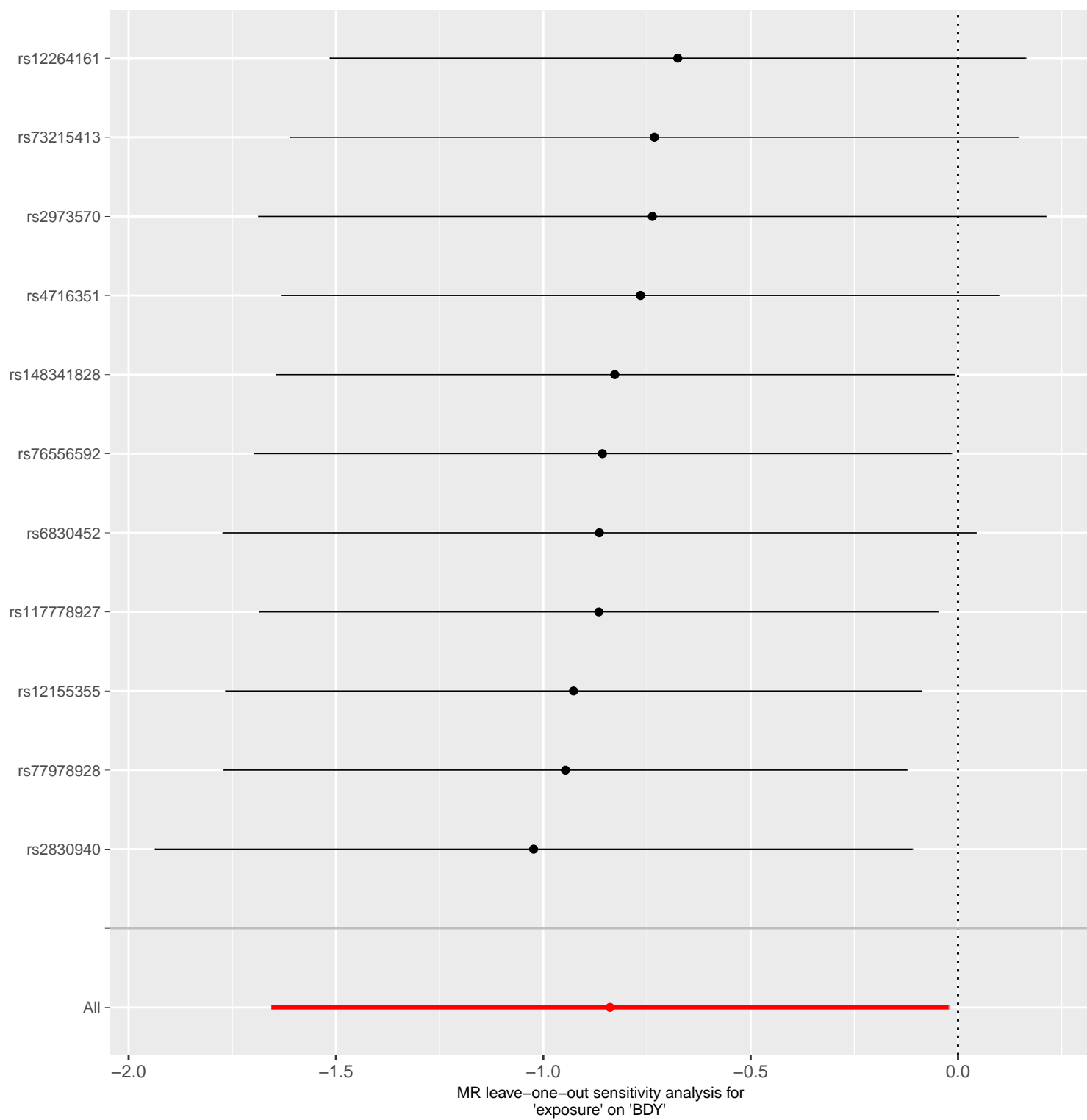

Supplement: Supplementary file 1 [file Data_Sheet_1.zip › Supplementary Materials/MR plots for tongue/Bronchiectasis/s__F0422_sp001553345_mgs_3021/leave_one_out.pdf]

# MR Test

- Inverse variance weighted
- MR Egger
- Weighted median

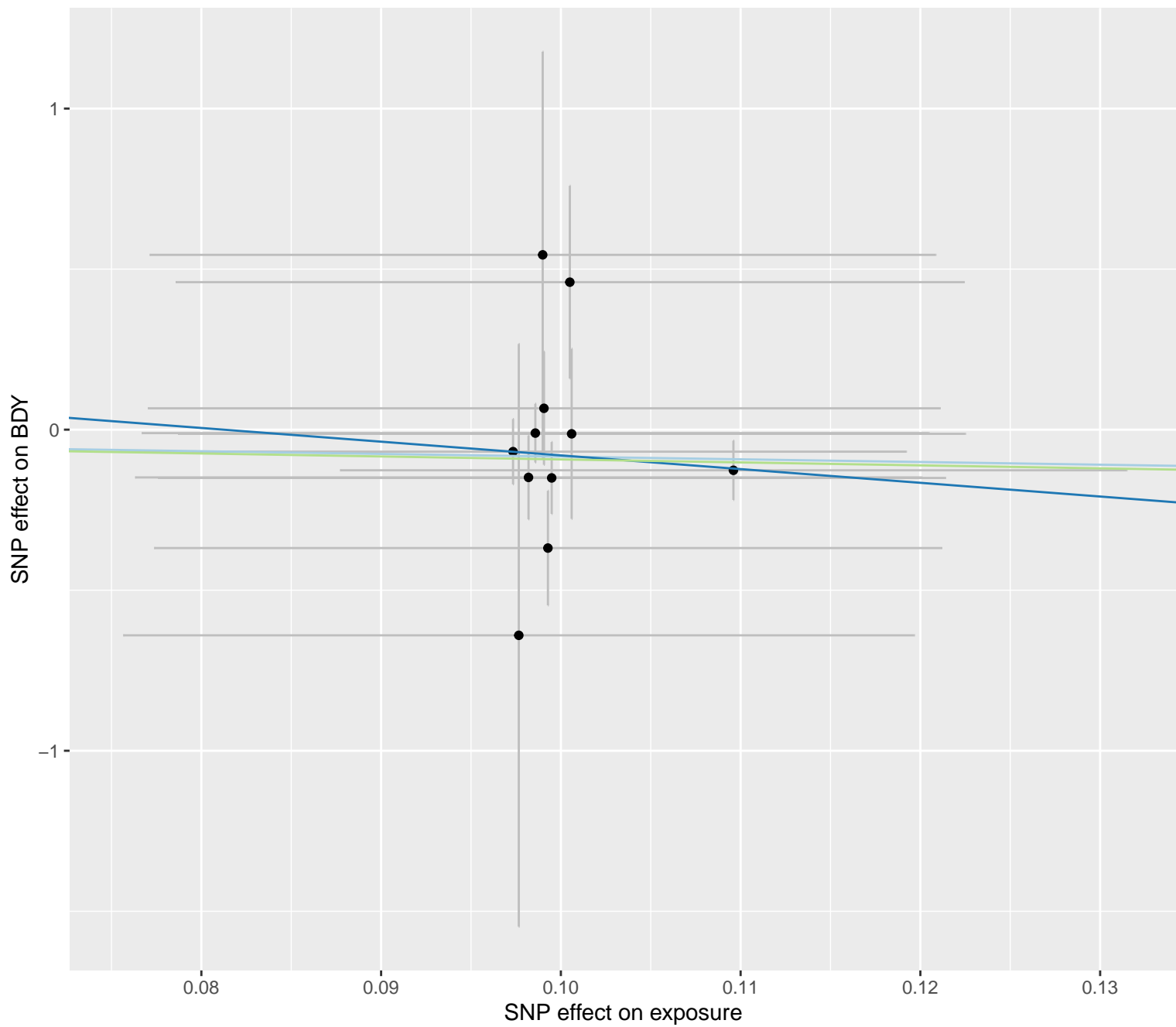

Supplement: Supplementary file 1 [file Data_Sheet_1.zip › Supplementary Materials/MR plots for tongue/Bronchiectasis/s__F0422_sp001553345_mgs_3021/scatter.pdf]

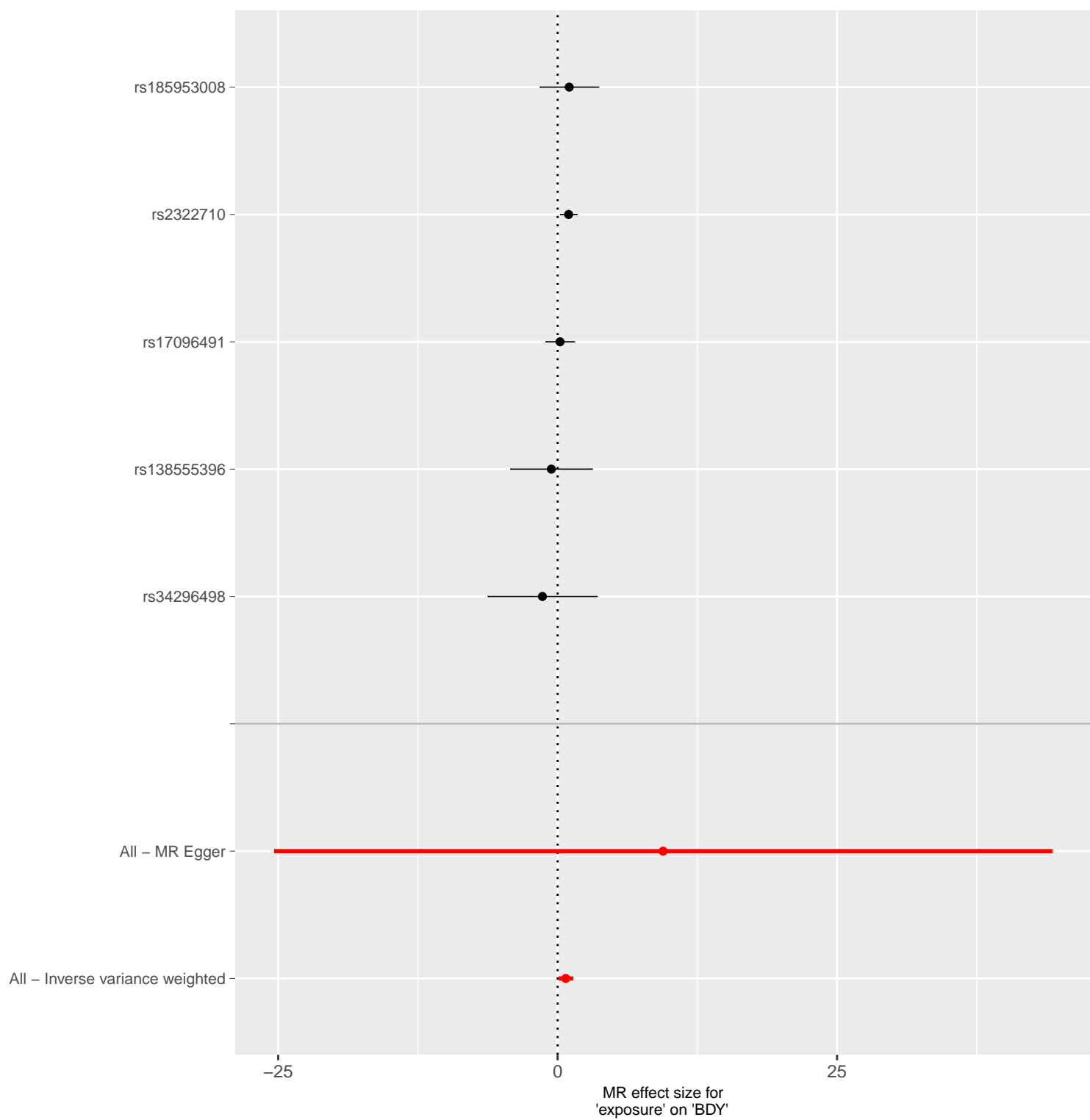

Supplement: Supplementary file 1 [file Data_Sheet_1.zip › Supplementary Materials/MR plots for tongue/Bronchitis/s__Haemophilus_haemolyticus_mgs_1068/forest.pdf]

# MR Method

- Inverse variance weighted
- MR Egger

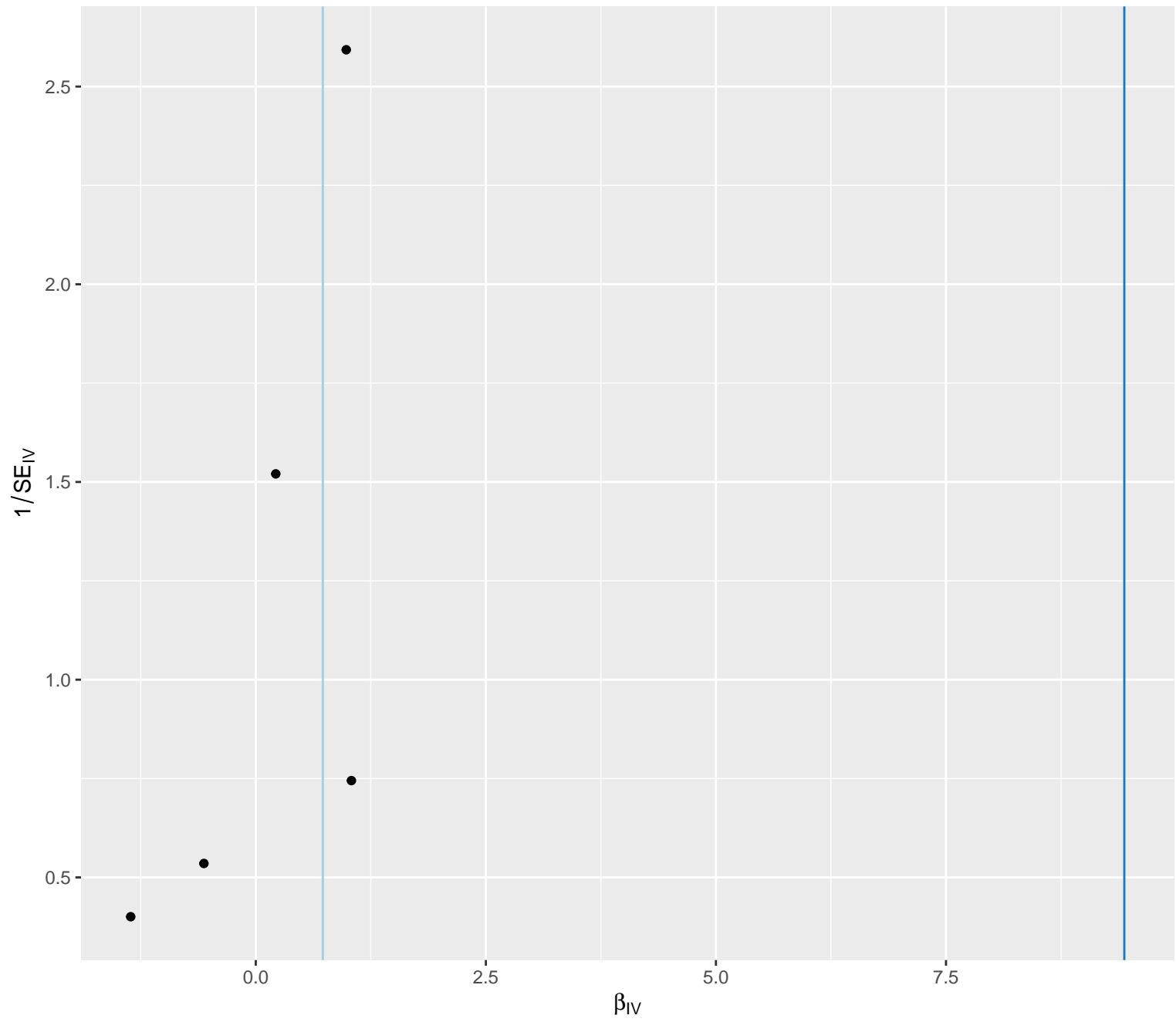

Supplement: Supplementary file 1 [file Data_Sheet_1.zip › Supplementary Materials/MR plots for tongue/Bronchitis/s__Haemophilus_haemolyticus_mgs_1068/funnel.pdf]

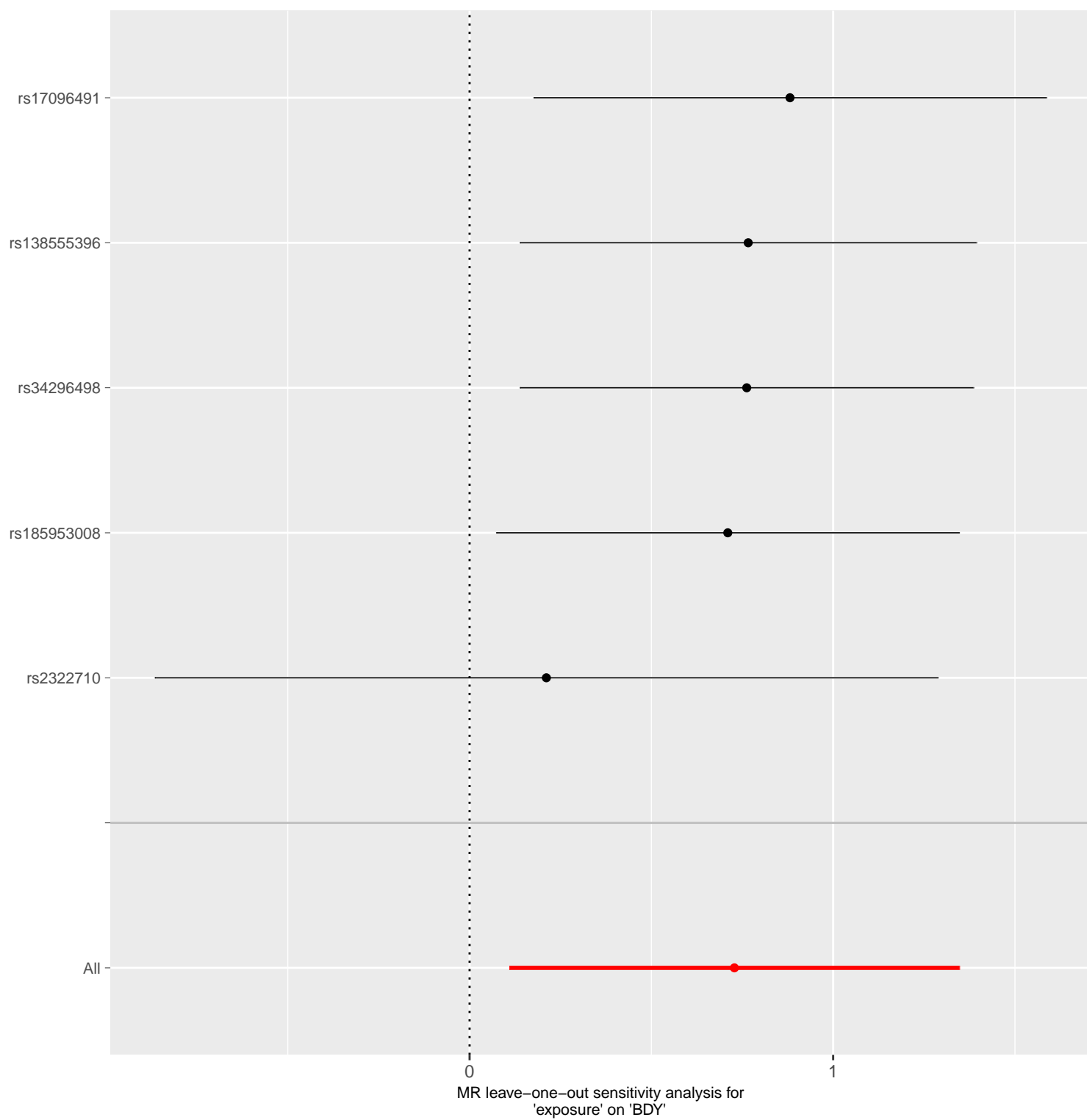

Supplement: Supplementary file 1 [file Data_Sheet_1.zip › Supplementary Materials/MR plots for tongue/Bronchitis/s__Haemophilus_haemolyticus_mgs_1068/leave_one_out.pdf]

# MR Test

- Inverse variance weighted
- MR Egger
- Weighted median

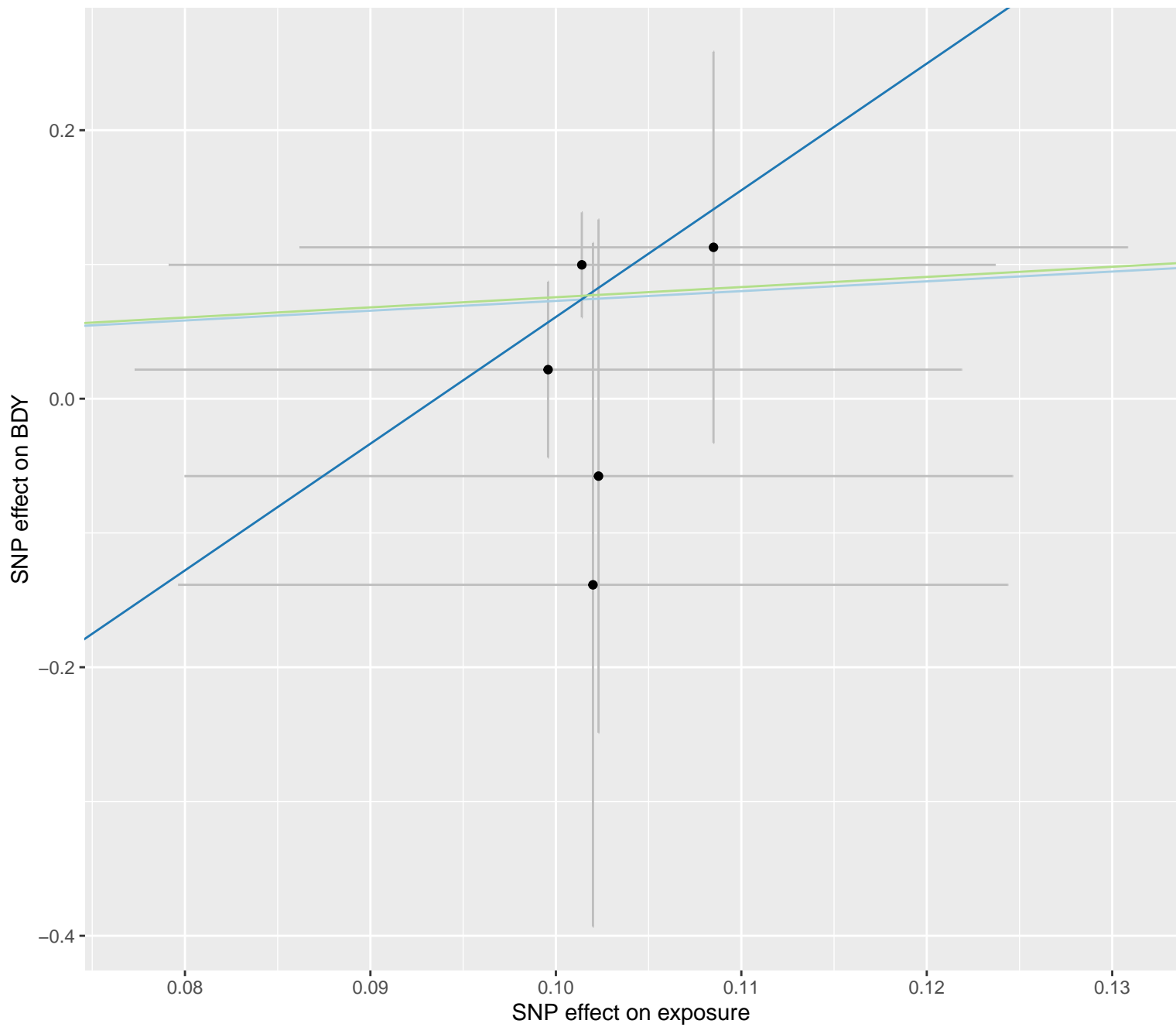

Supplement: Supplementary file 1 [file Data_Sheet_1.zip › Supplementary Materials/MR plots for tongue/Bronchitis/s__Haemophilus_haemolyticus_mgs_1068/scatter.pdf]

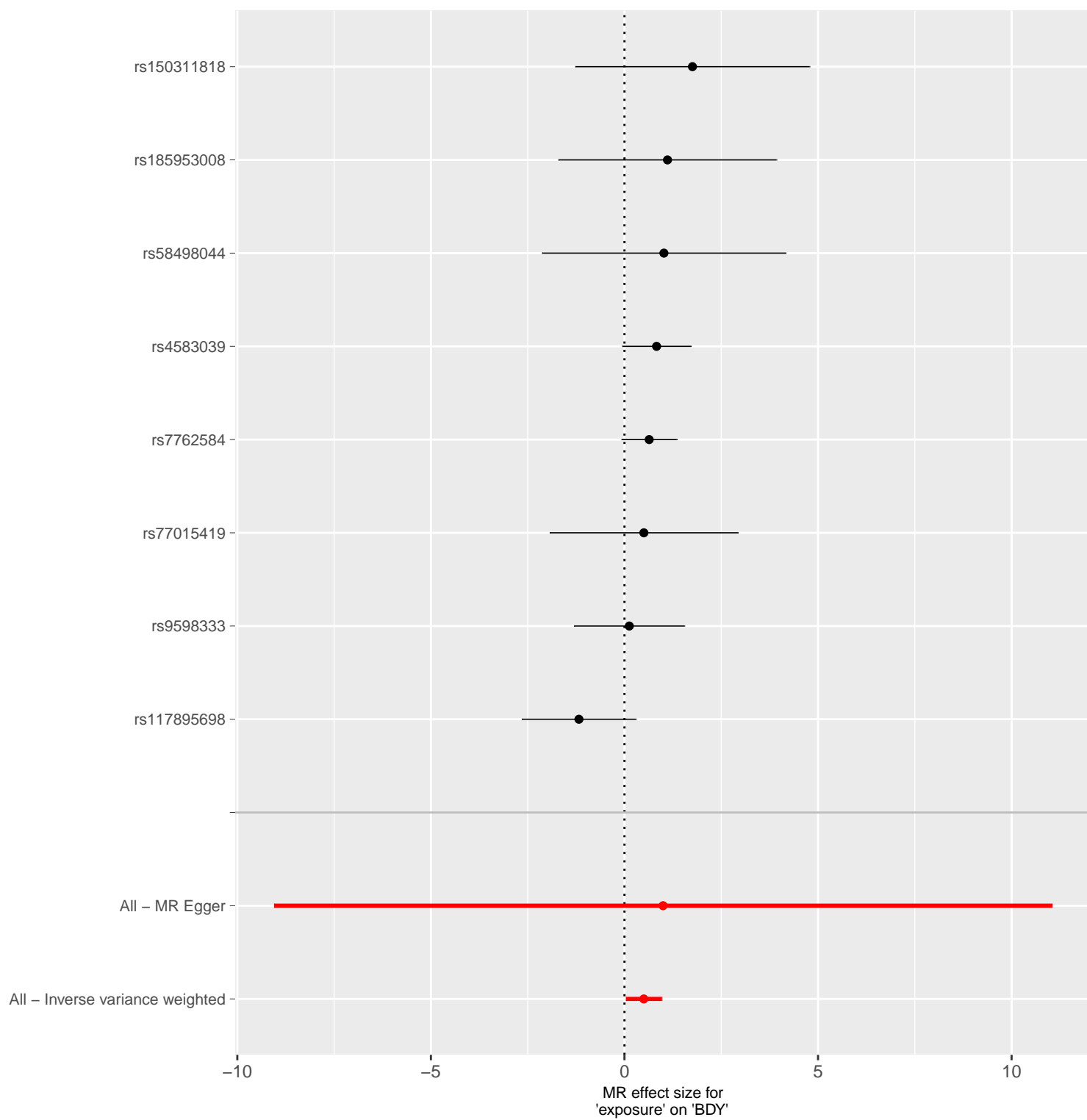

Supplement: Supplementary file 1 [file Data_Sheet_1.zip › Supplementary Materials/MR plots for tongue/Bronchitis/s__Haemophilus_sp002998595_mgs_2250/forest.pdf]

# MR Method

- Inverse variance weighted
- MR Egger

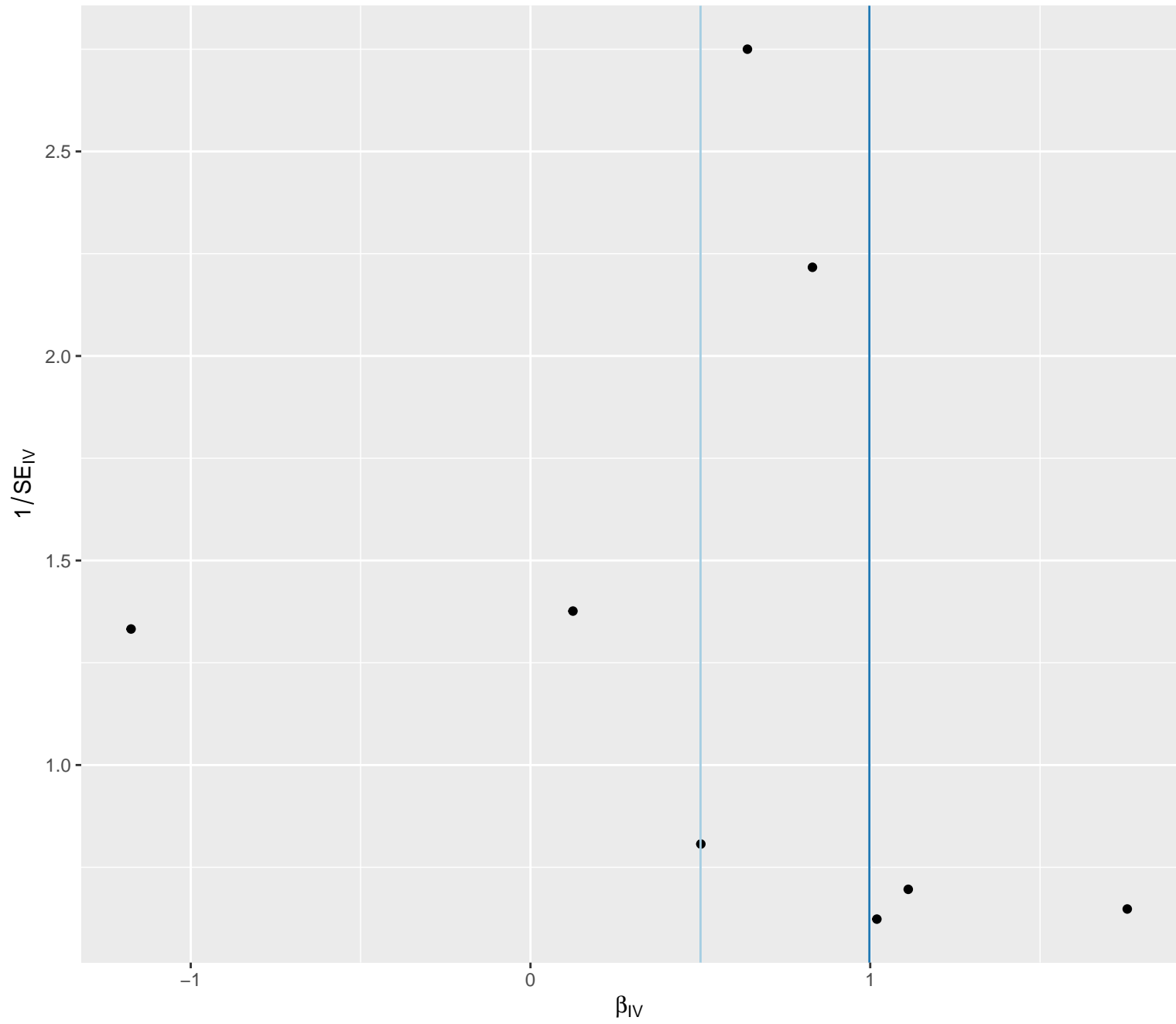

Supplement: Supplementary file 1 [file Data_Sheet_1.zip › Supplementary Materials/MR plots for tongue/Bronchitis/s__Haemophilus_sp002998595_mgs_2250/funnel.pdf]

rs117895698

rs9598333

rs77015419

rs58498044

rs185953008

rs150311818

rs7762584

rs4583039

All

0.0

0.4

0.8

1.2

MR leave-one-out sensitivity analysis for  
'exposure' on 'BDY'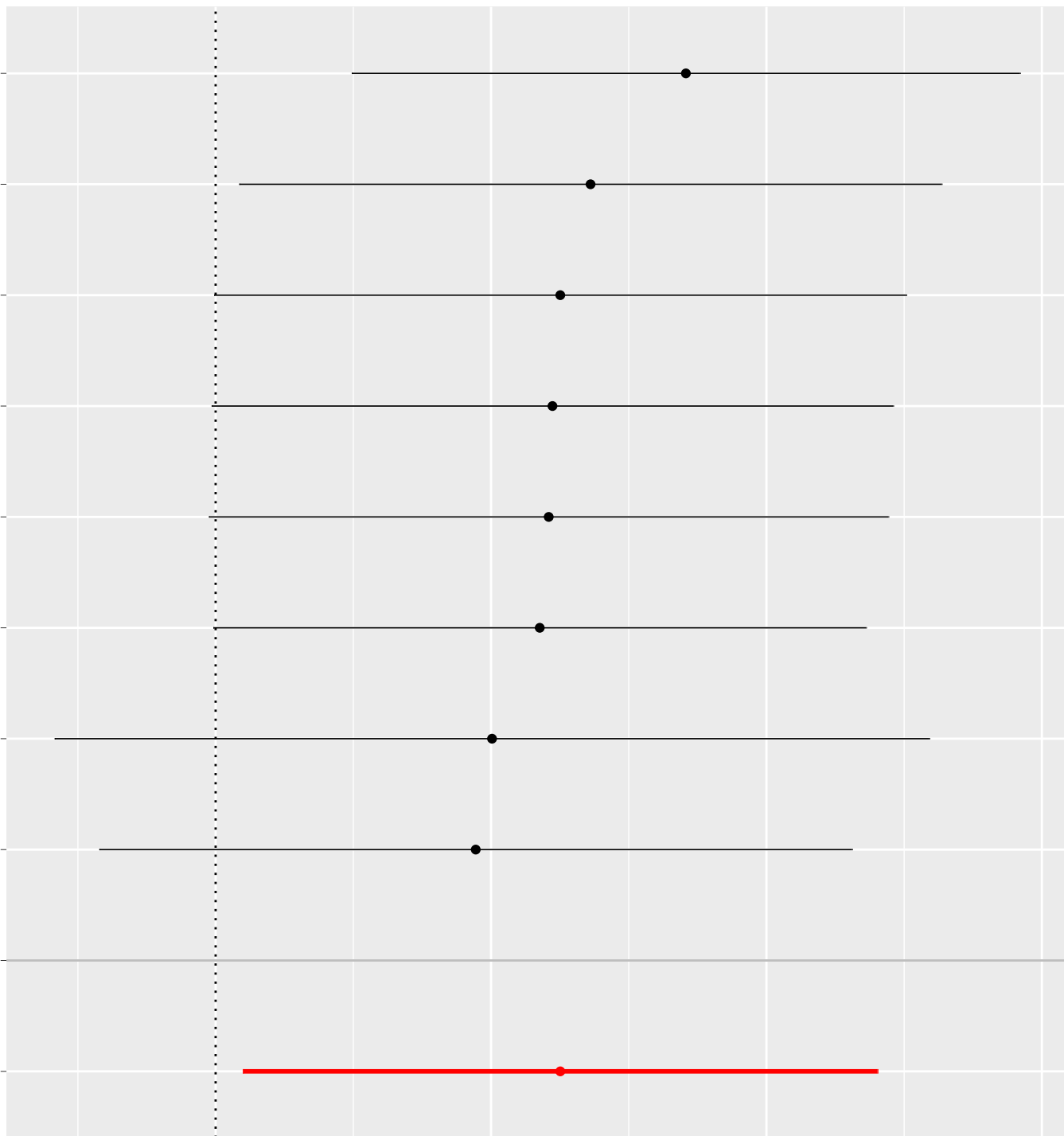

Supplement: Supplementary file 1 [file Data_Sheet_1.zip › Supplementary Materials/MR plots for tongue/Bronchitis/s__Haemophilus_sp002998595_mgs_2250/leave_one_out.pdf]

# MR Test

- Inverse variance weighted
- MR Egger
- Weighted median

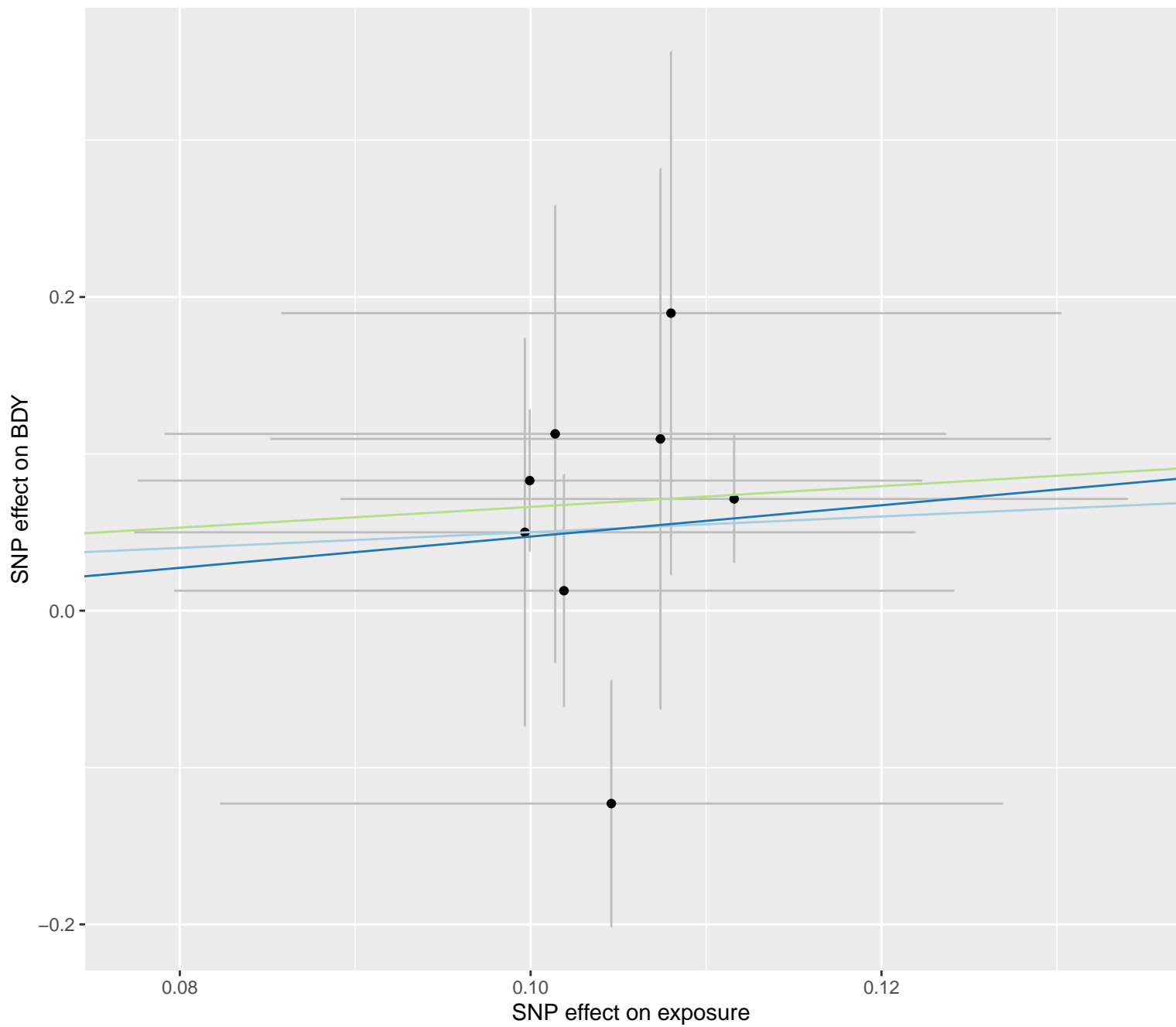

Supplement: Supplementary file 1 [file Data_Sheet_1.zip › Supplementary Materials/MR plots for tongue/Bronchitis/s__Haemophilus_sp002998595_mgs_2250/scatter.pdf]

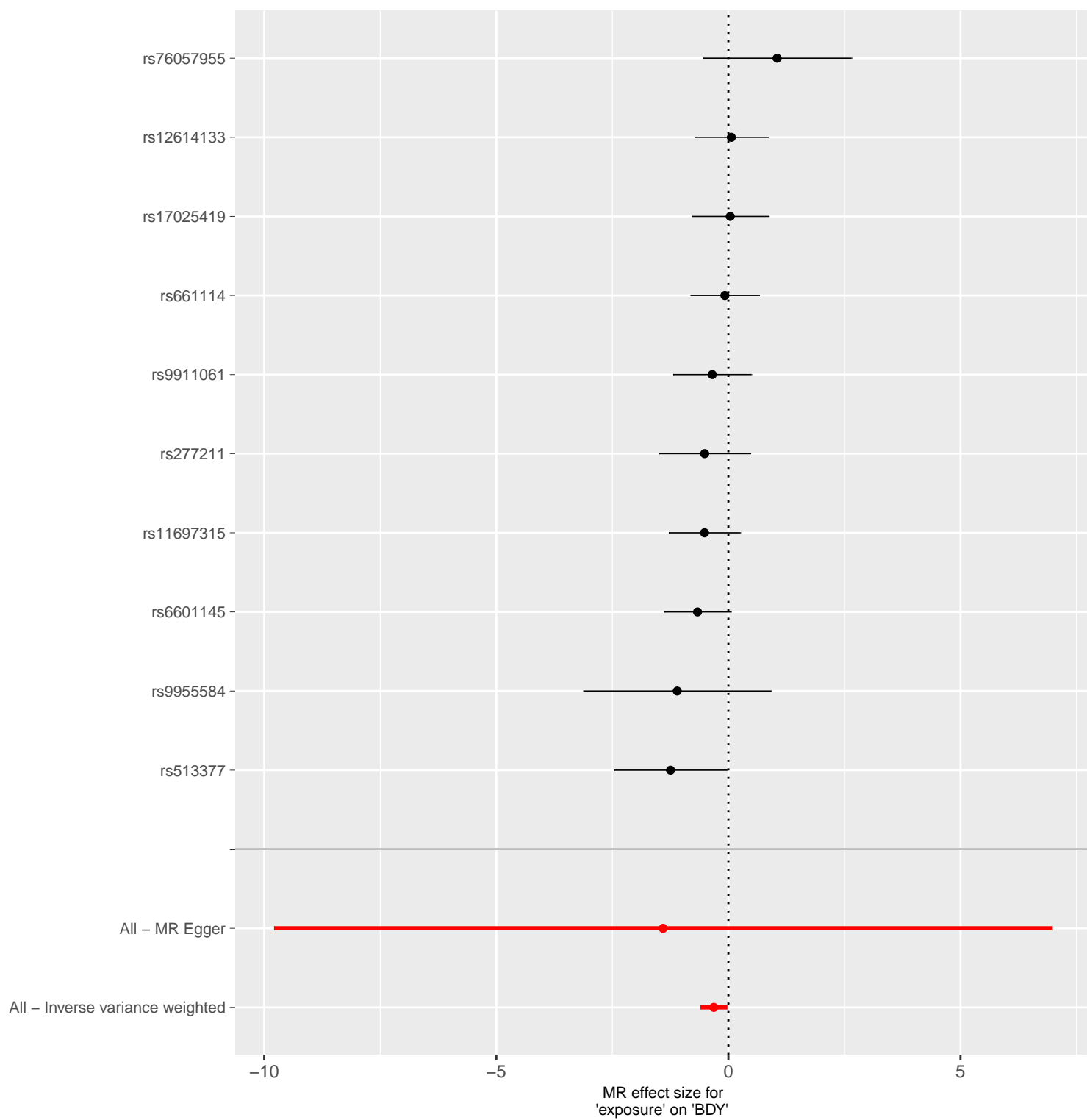

Supplement: Supplementary file 1 [file Data_Sheet_1.zip › Supplementary Materials/MR plots for tongue/Bronchitis/s__Pauljensenia_pyogenes_mgs_3542/forest.pdf]

# MR Method

- Inverse variance weighted
- MR Egger

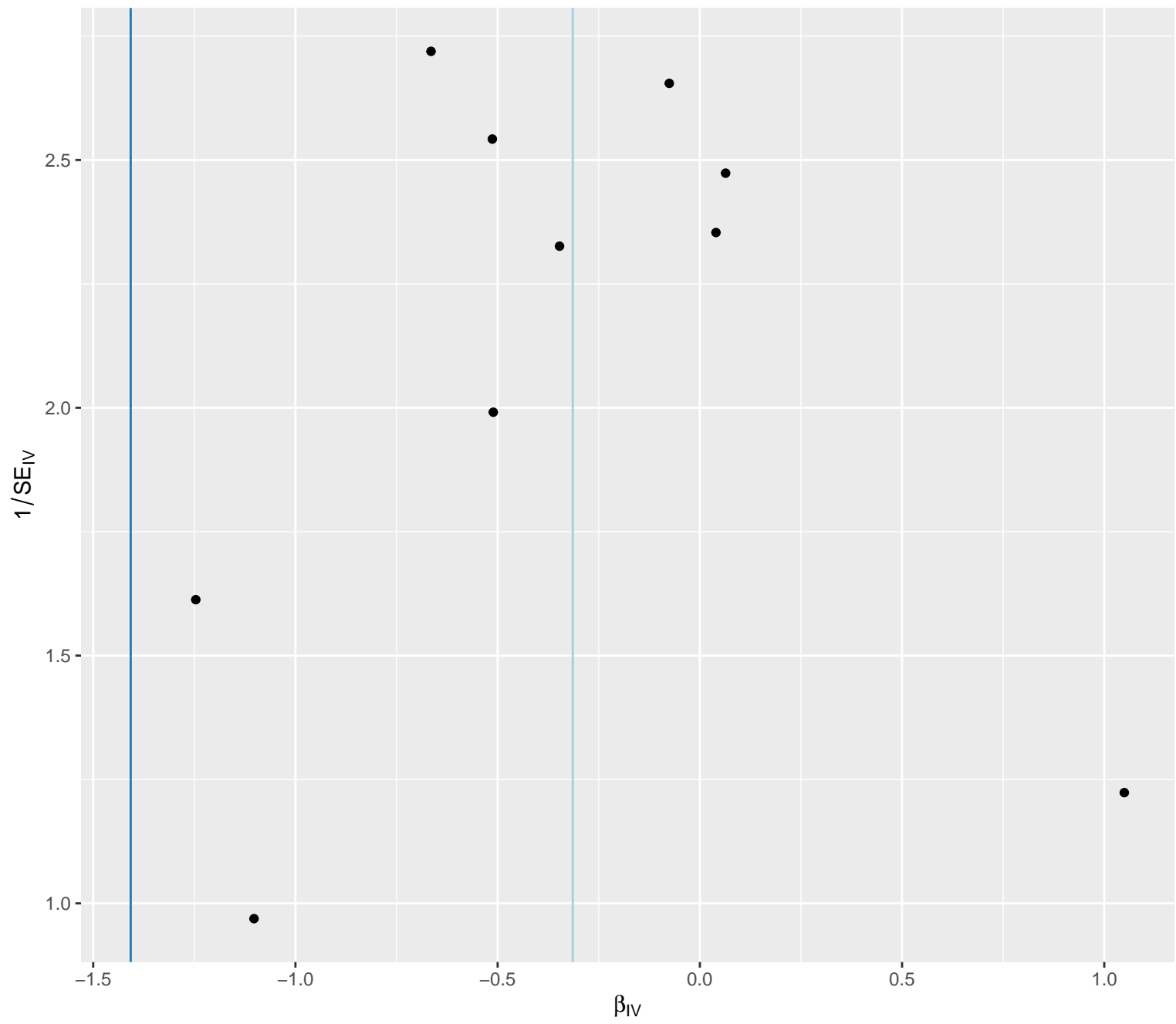

Supplement: Supplementary file 1 [file Data_Sheet_1.zip › Supplementary Materials/MR plots for tongue/Bronchitis/s__Pauljensenia_pyogenes_mgs_3542/funnel.pdf]

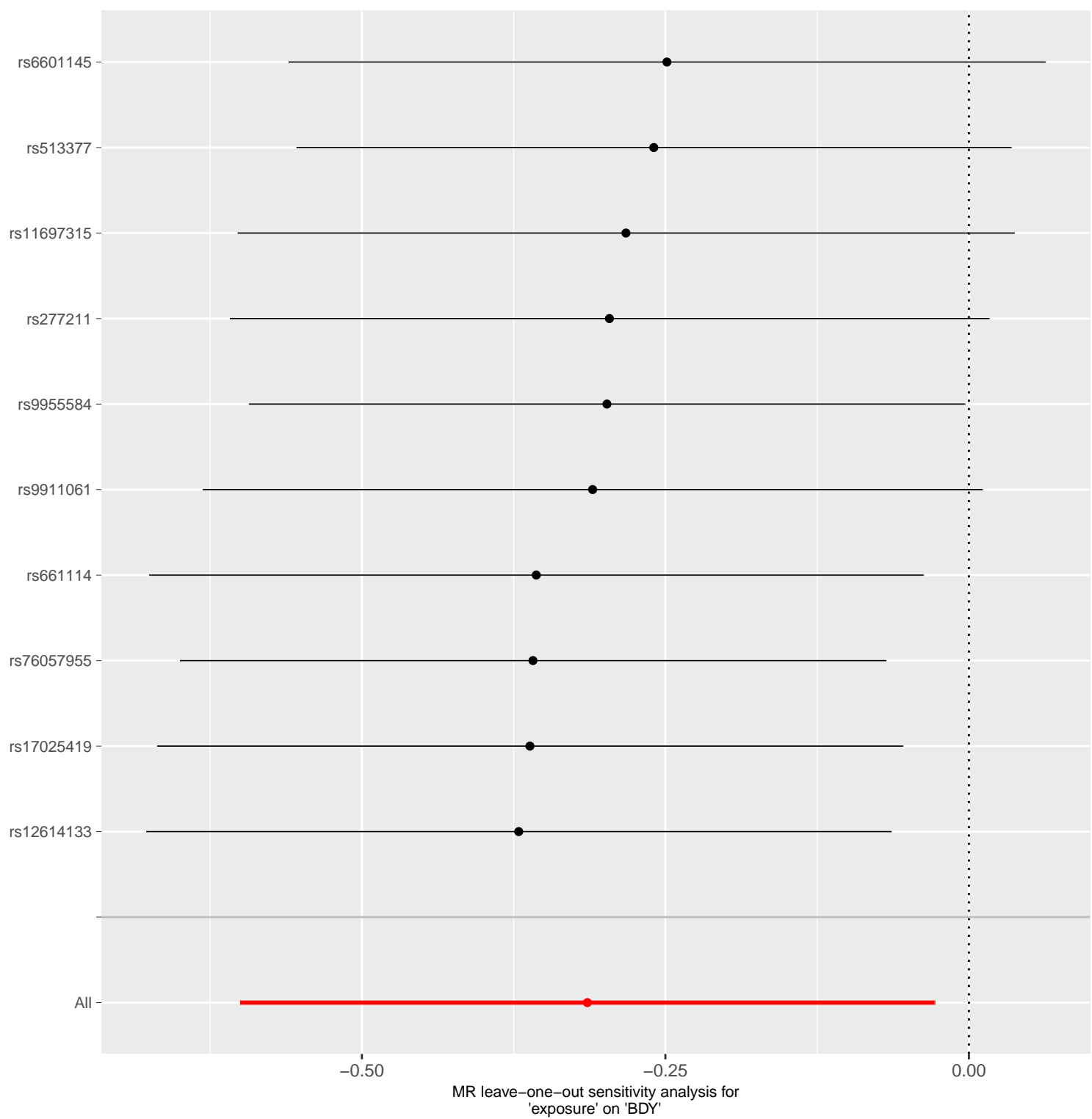

Supplement: Supplementary file 1 [file Data_Sheet_1.zip › Supplementary Materials/MR plots for tongue/Bronchitis/s__Pauljensenia_pyogenes_mgs_3542/leave_one_out.pdf]

# MR Test

- Inverse variance weighted
- MR Egger
- Weighted median

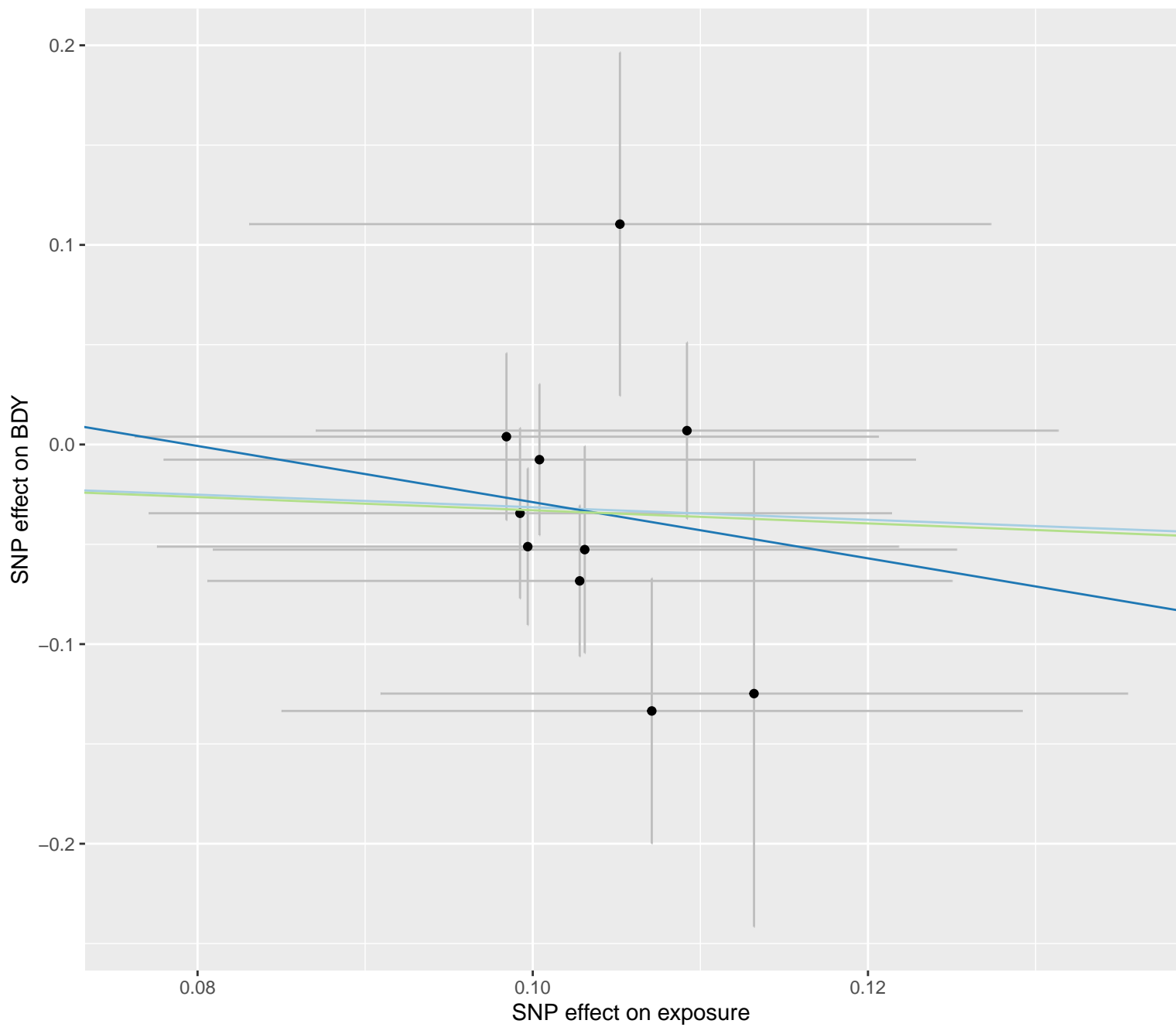

Supplement: Supplementary file 1 [file Data_Sheet_1.zip › Supplementary Materials/MR plots for tongue/Bronchitis/s__Pauljensenia_pyogenes_mgs_3542/scatter.pdf]

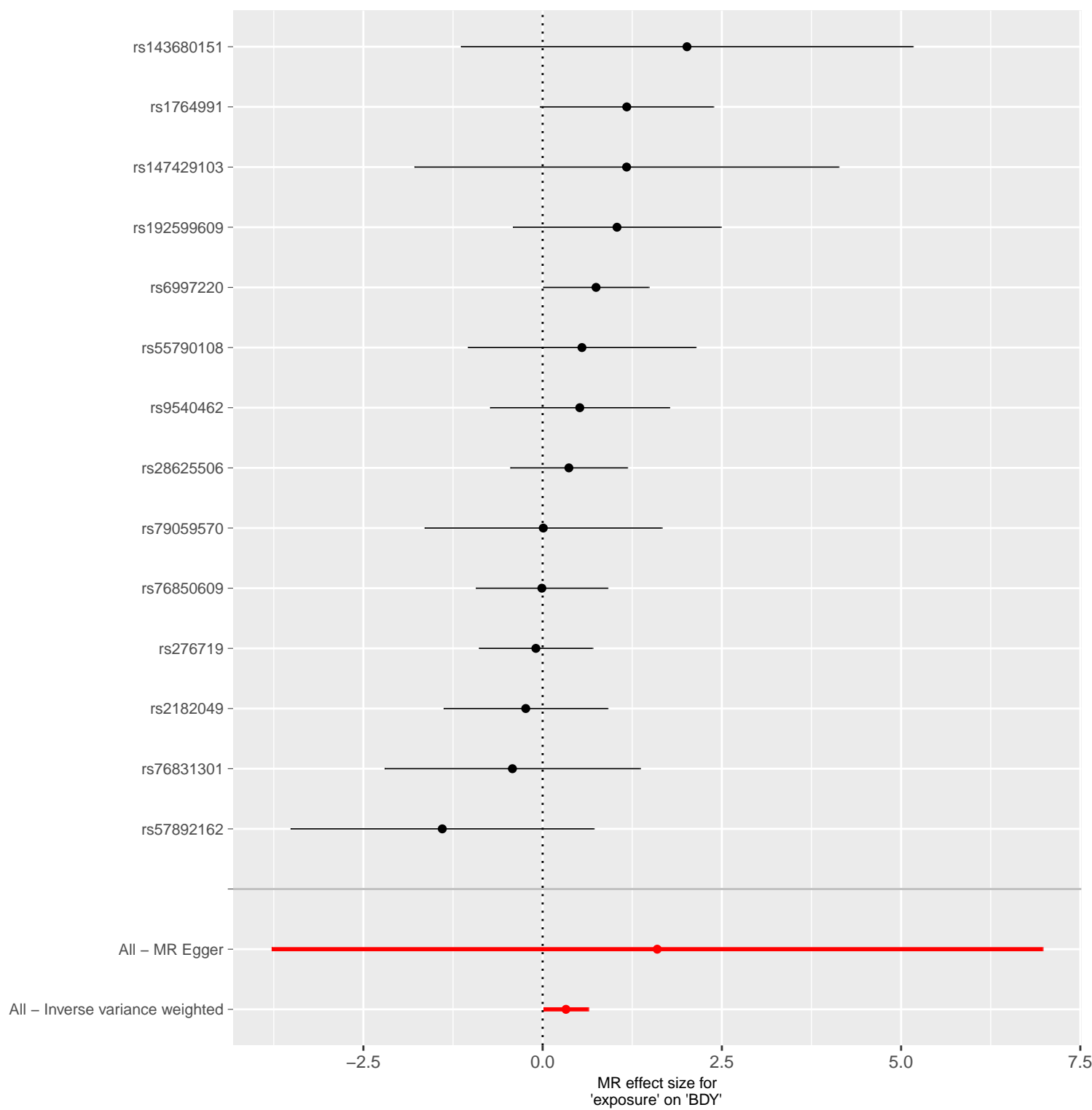

Supplement: Supplementary file 1 [file Data_Sheet_1.zip › Supplementary Materials/MR plots for tongue/Bronchitis/s__Porphyromonas_endodontalis_mgs_3304/forest.pdf]

# MR Method

- Inverse variance weighted
- MR Egger

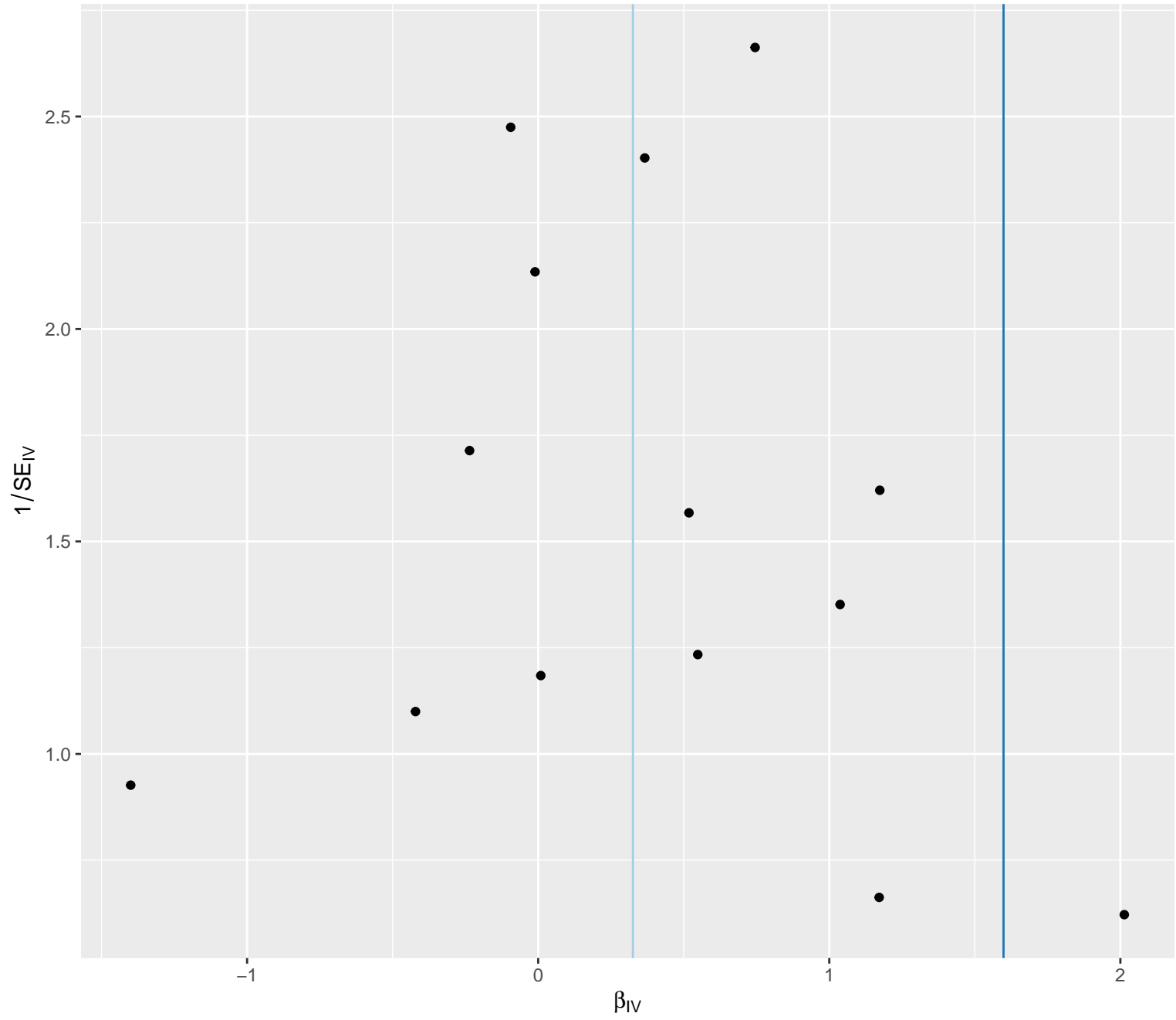

Supplement: Supplementary file 1 [file Data_Sheet_1.zip › Supplementary Materials/MR plots for tongue/Bronchitis/s__Porphyromonas_endodontalis_mgs_3304/funnel.pdf]

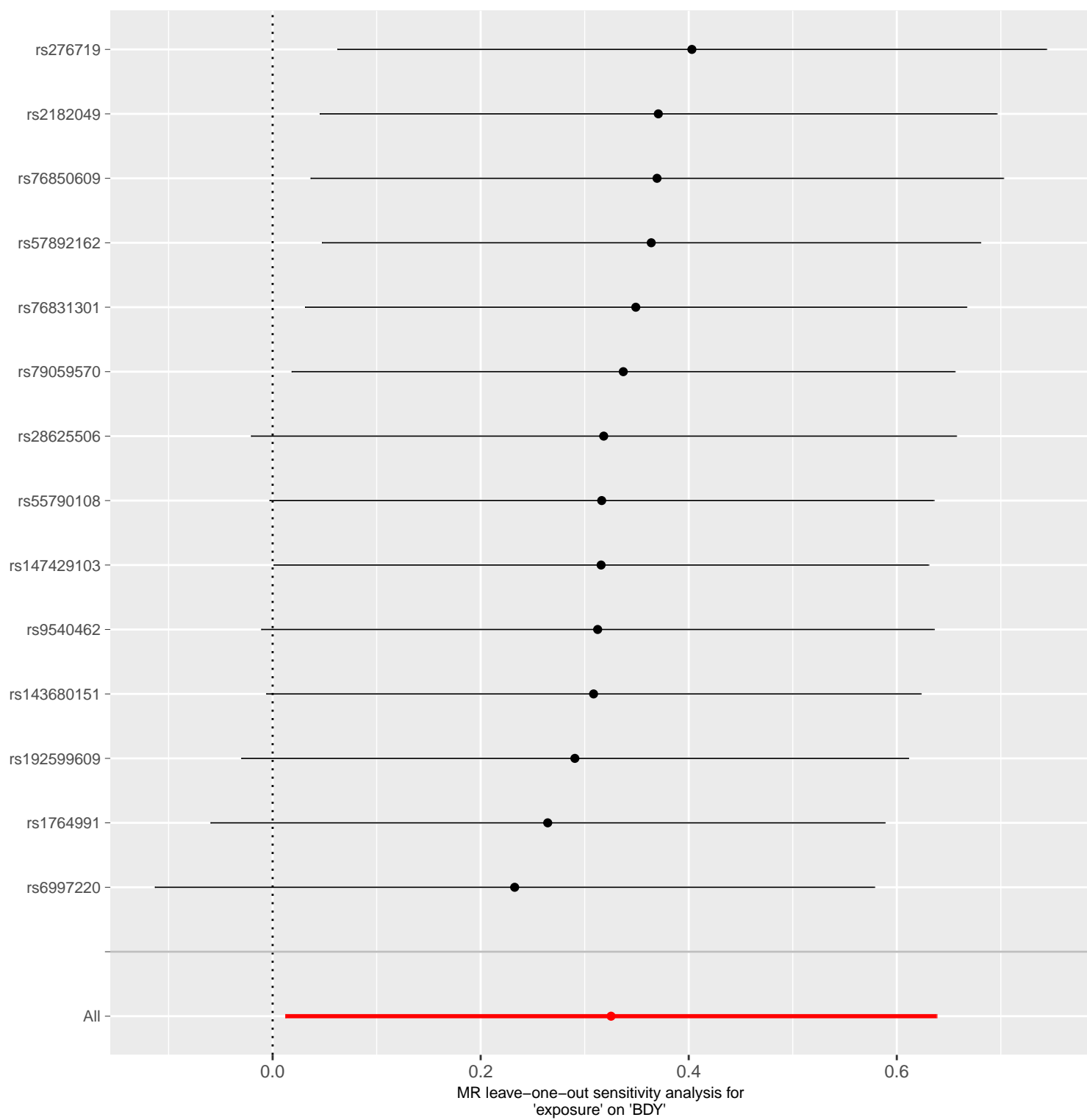

Supplement: Supplementary file 1 [file Data_Sheet_1.zip › Supplementary Materials/MR plots for tongue/Bronchitis/s__Porphyromonas_endodontalis_mgs_3304/leave_one_out.pdf]

# MR Test

- Inverse variance weighted
- MR Egger
- Weighted median

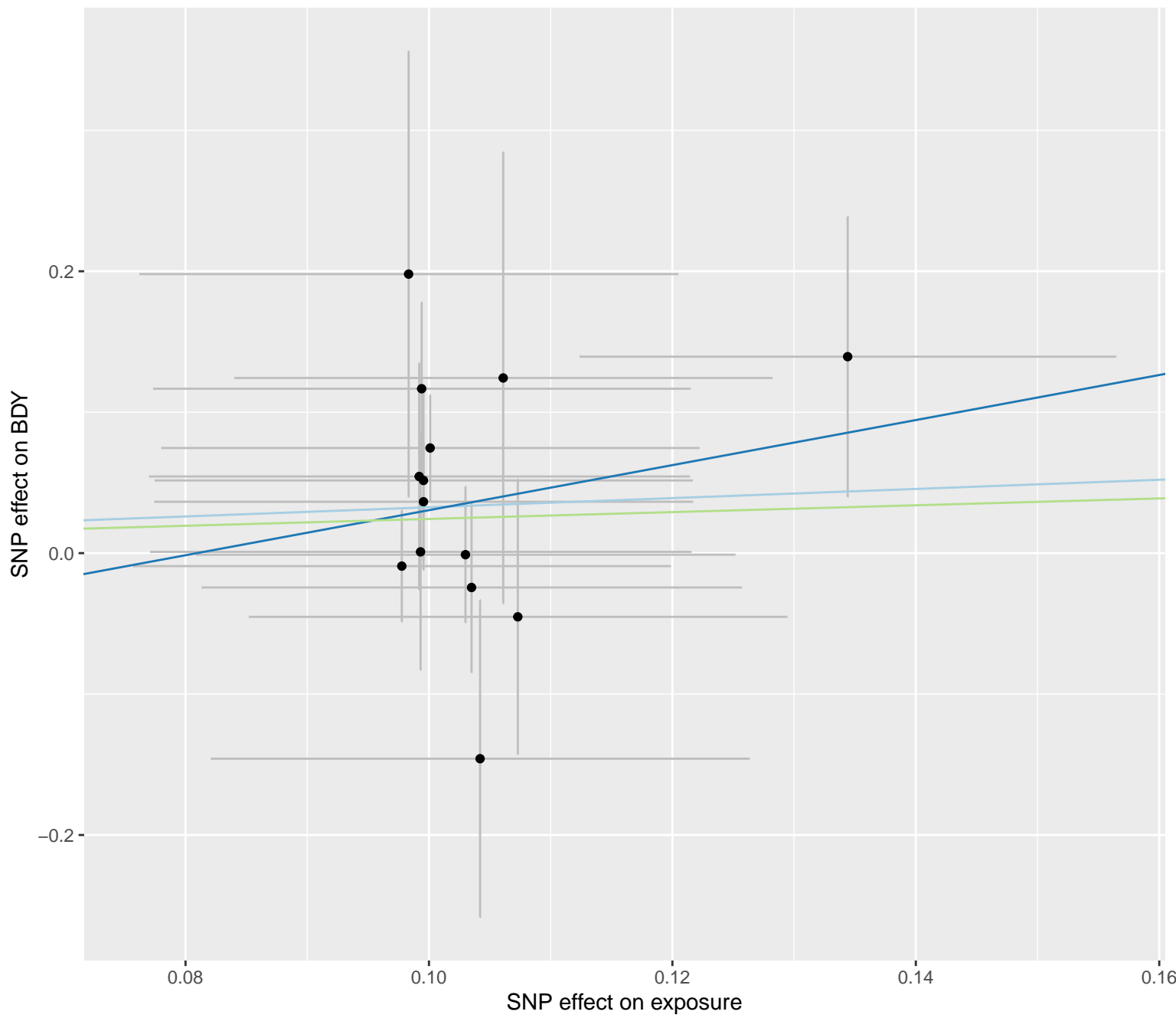

Supplement: Supplementary file 1 [file Data_Sheet_1.zip › Supplementary Materials/MR plots for tongue/Bronchitis/s__Porphyromonas_endodontalis_mgs_3304/scatter.pdf]

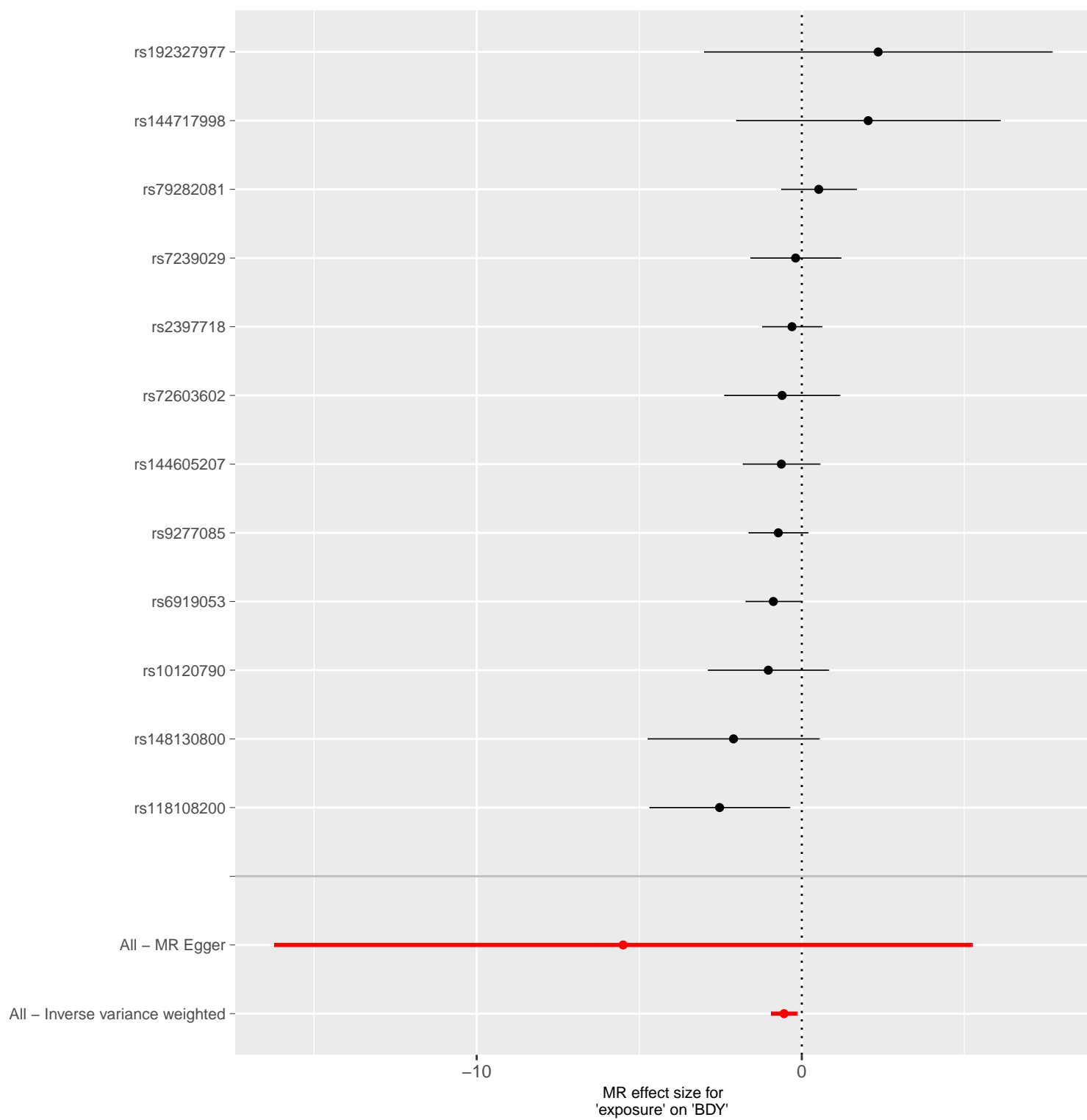

Supplement: Supplementary file 1 [file Data_Sheet_1.zip › Supplementary Materials/MR plots for tongue/Bronchitis/s__Streptococcus_pseudopneumoniae_O_mgs_1112/forest.pdf]

# MR Method

- Inverse variance weighted
- MR Egger

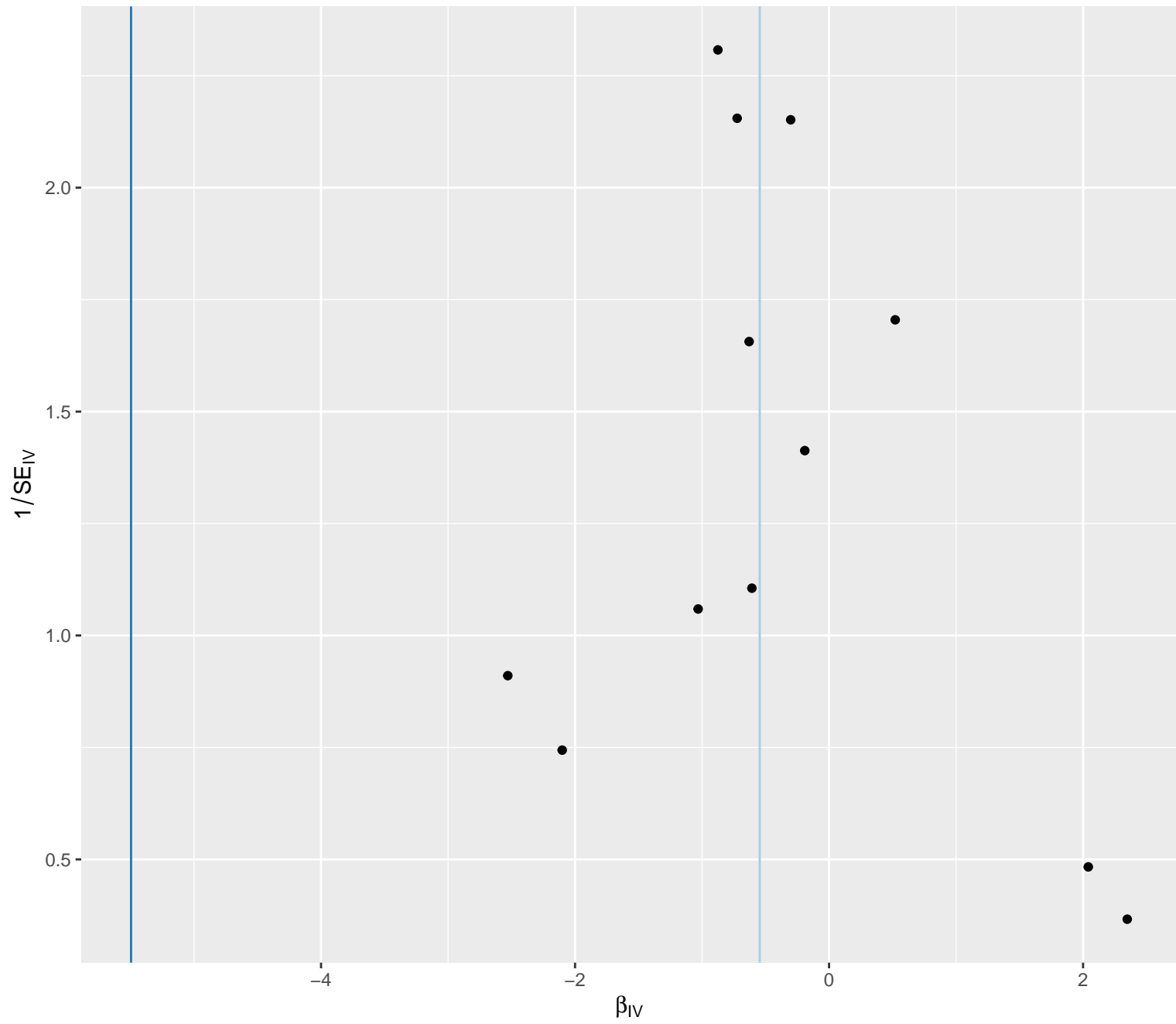

Supplement: Supplementary file 1 [file Data_Sheet_1.zip › Supplementary Materials/MR plots for tongue/Bronchitis/s__Streptococcus_pseudopneumoniae_O_mgs_1112/funnel.pdf]

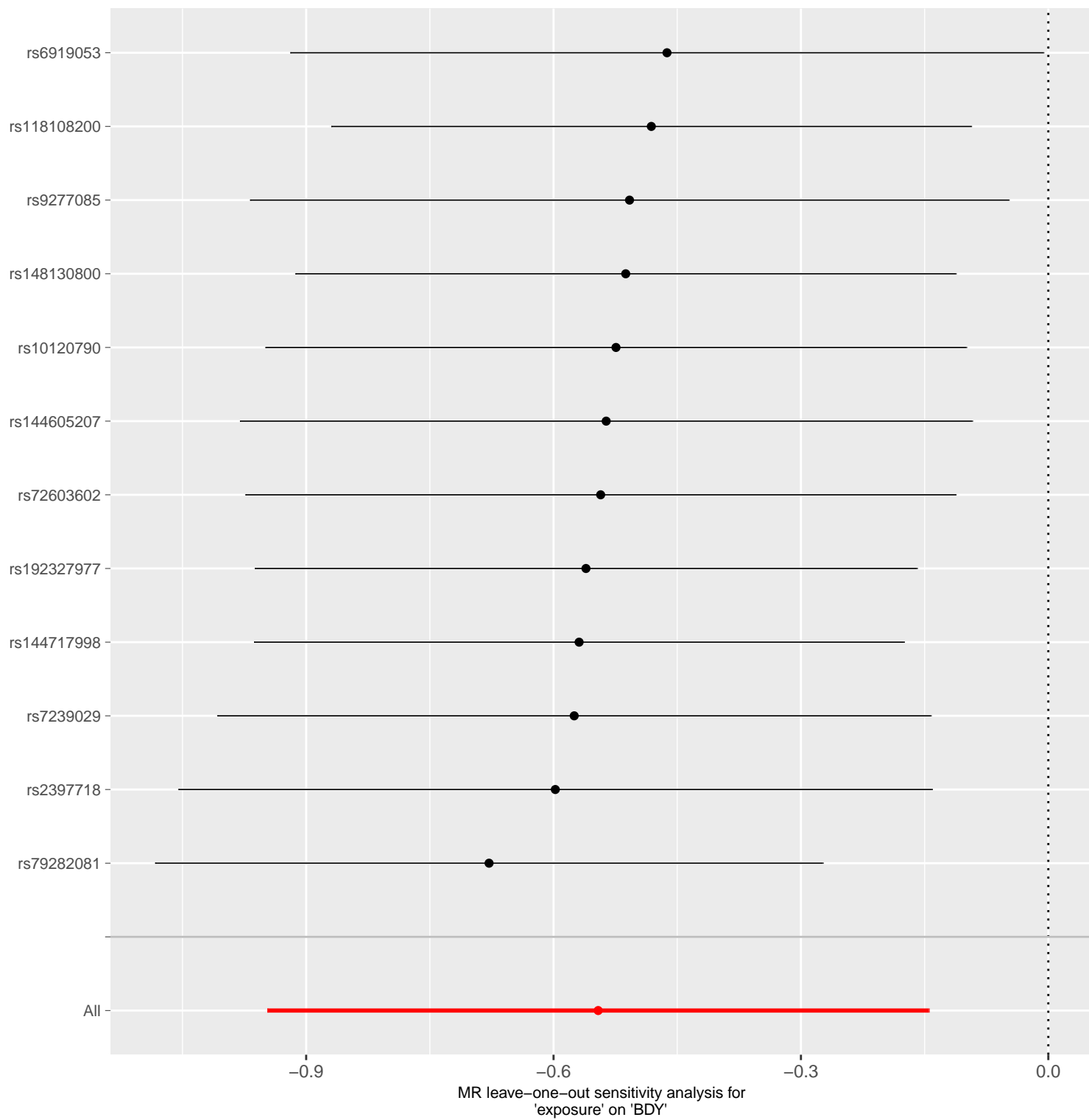

Supplement: Supplementary file 1 [file Data_Sheet_1.zip › Supplementary Materials/MR plots for tongue/Bronchitis/s__Streptococcus_pseudopneumoniae_O_mgs_1112/leave_one_out.pdf]

# MR Test

- Inverse variance weighted
- MR Egger
- Weighted median

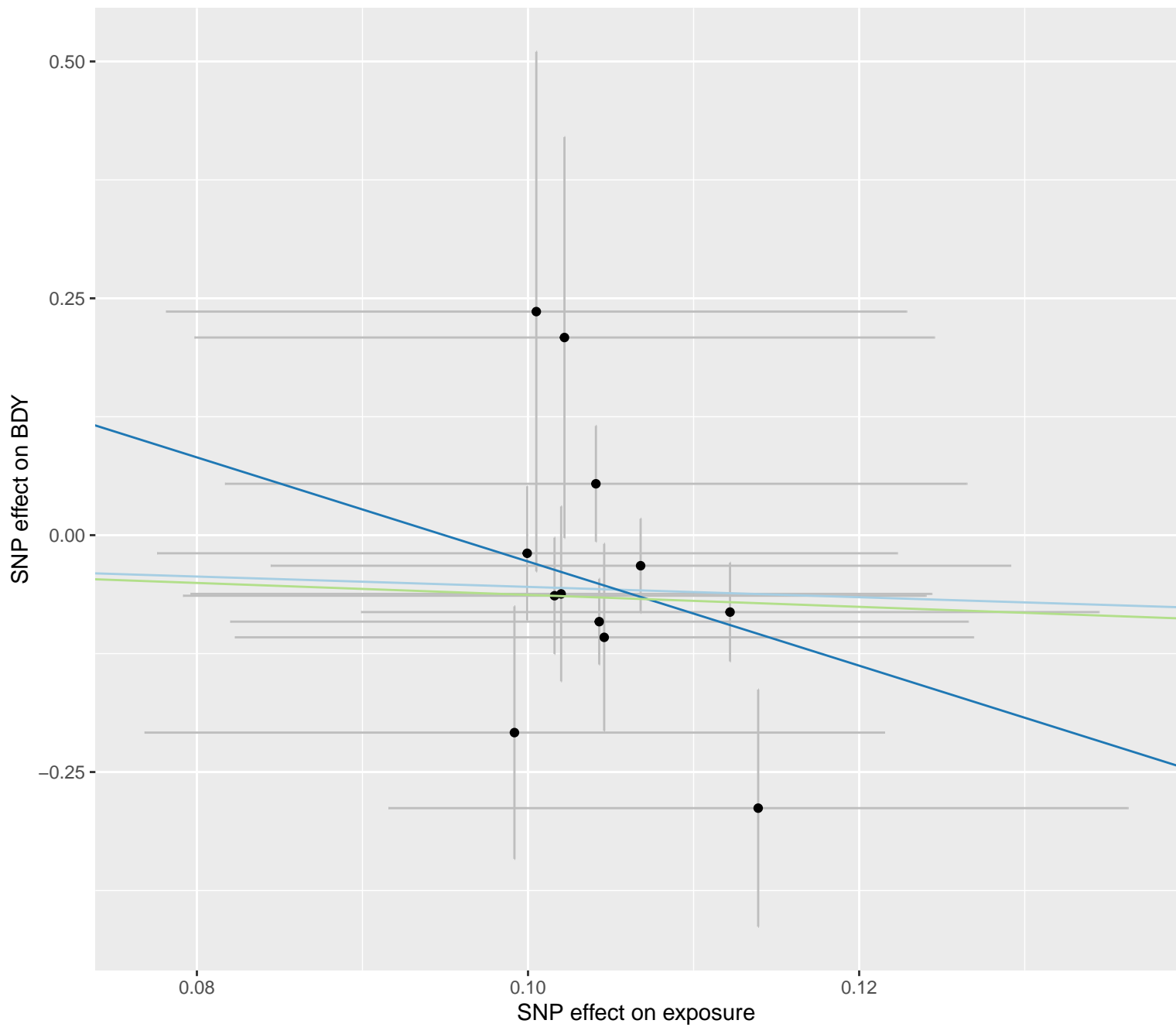

Supplement: Supplementary file 1 [file Data_Sheet_1.zip › Supplementary Materials/MR plots for tongue/Bronchitis/s__Streptococcus_pseudopneumoniae_O_mgs_1112/scatter.pdf]

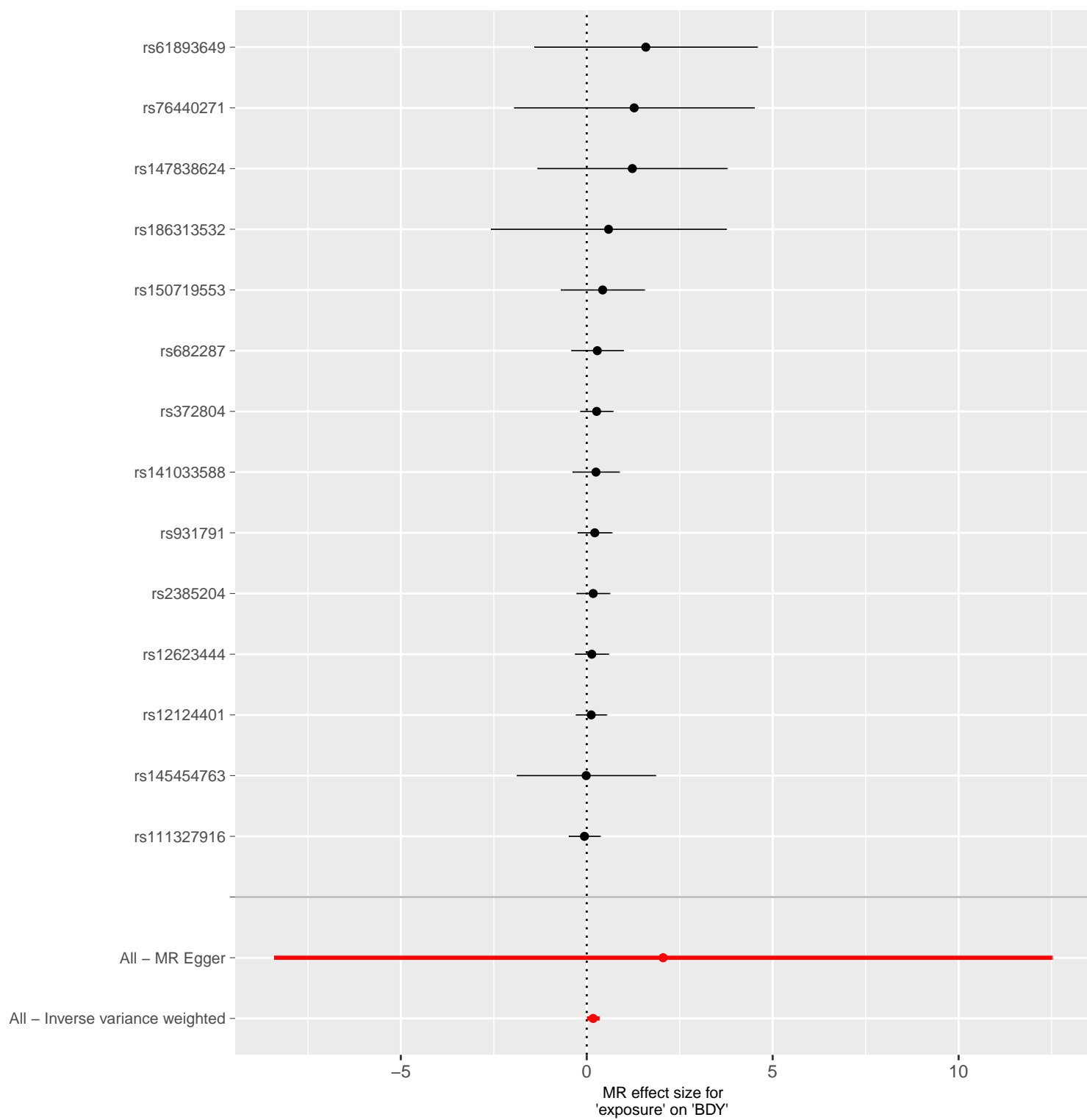

Supplement: Supplementary file 1 [file Data_Sheet_1.zip › Supplementary Materials/MR plots for tongue/Chronic sinusitis/s__F0040_sp900095835_mgs_3541/forest.pdf]

# MR Method

- Inverse variance weighted
- MR Egger

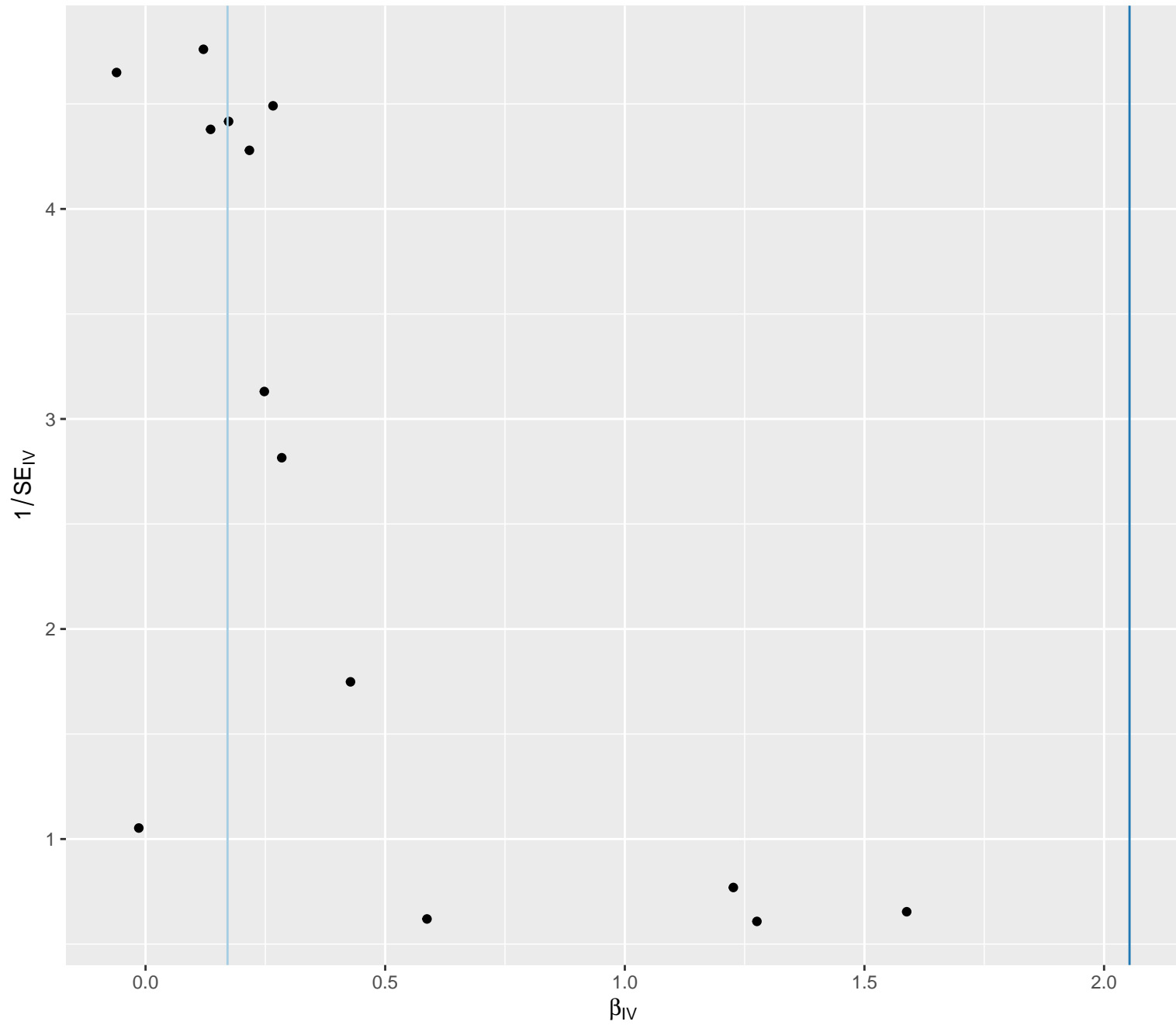

Supplement: Supplementary file 1 [file Data_Sheet_1.zip › Supplementary Materials/MR plots for tongue/Chronic sinusitis/s__F0040_sp900095835_mgs_3541/funnel.pdf]

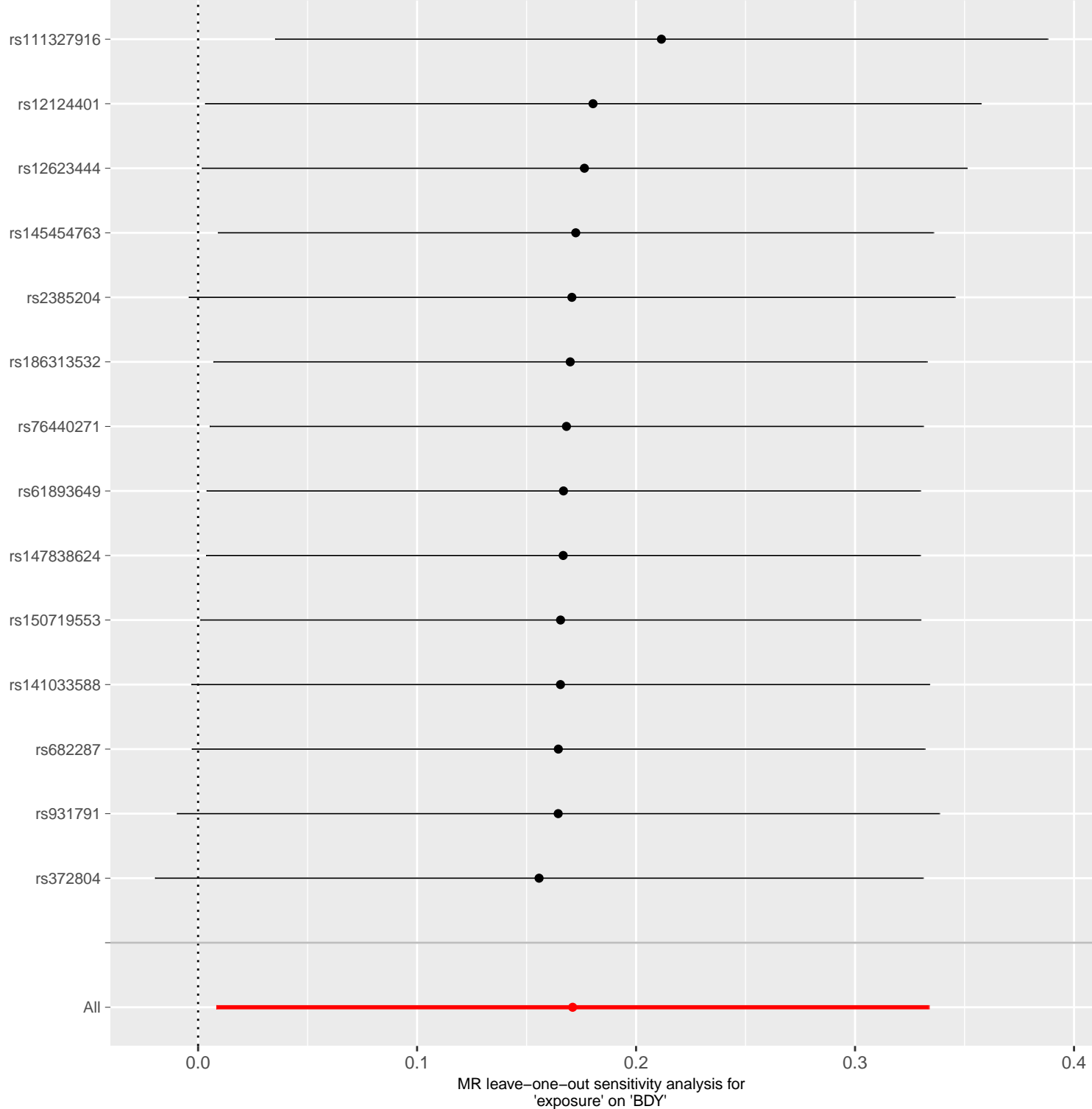

Supplement: Supplementary file 1 [file Data_Sheet_1.zip › Supplementary Materials/MR plots for tongue/Chronic sinusitis/s__F0040_sp900095835_mgs_3541/leave_one_out.pdf]

# MR Test

- Inverse variance weighted
- MR Egger
- Weighted median

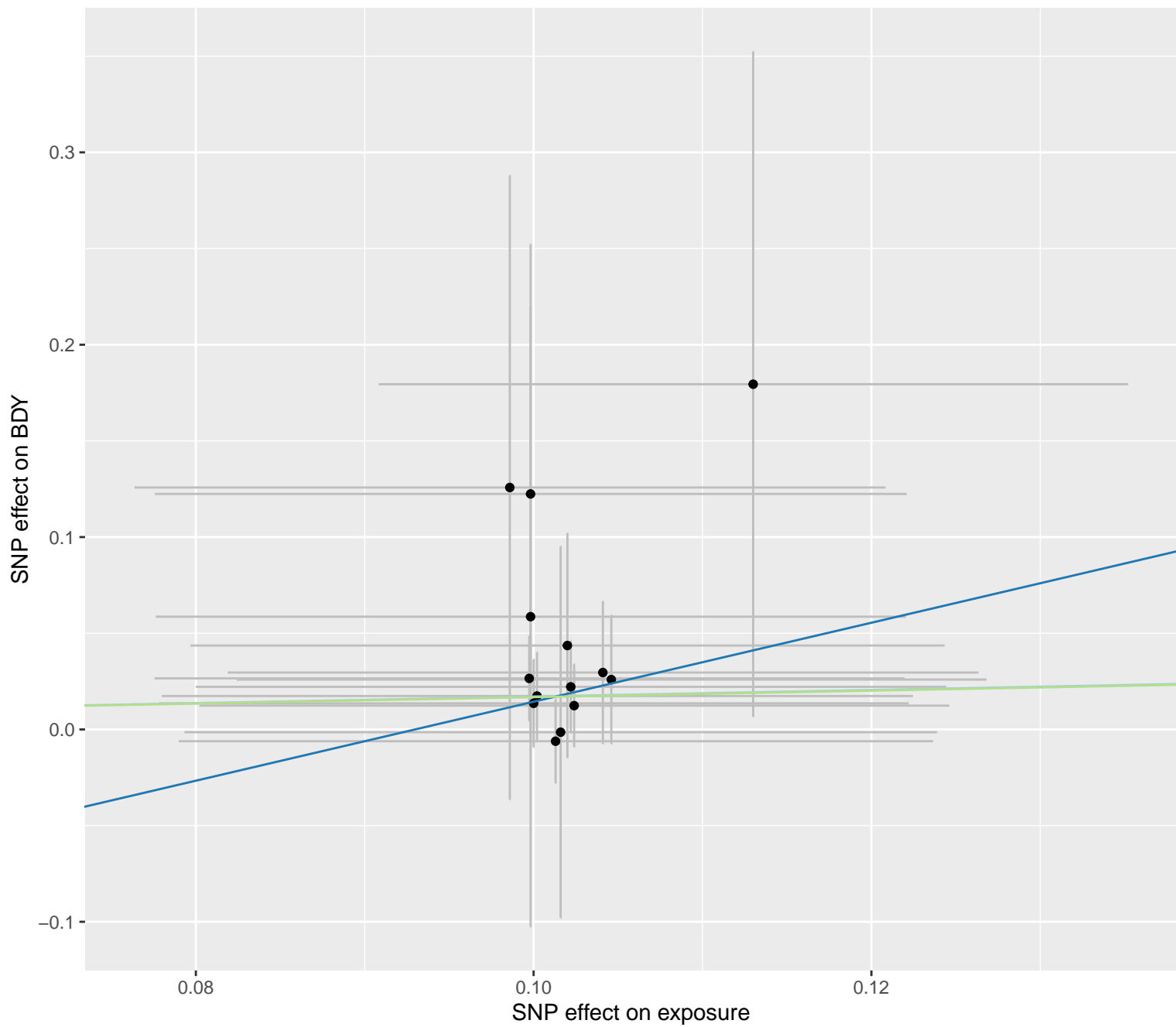

Supplement: Supplementary file 1 [file Data_Sheet_1.zip › Supplementary Materials/MR plots for tongue/Chronic sinusitis/s__F0040_sp900095835_mgs_3541/scatter.pdf]

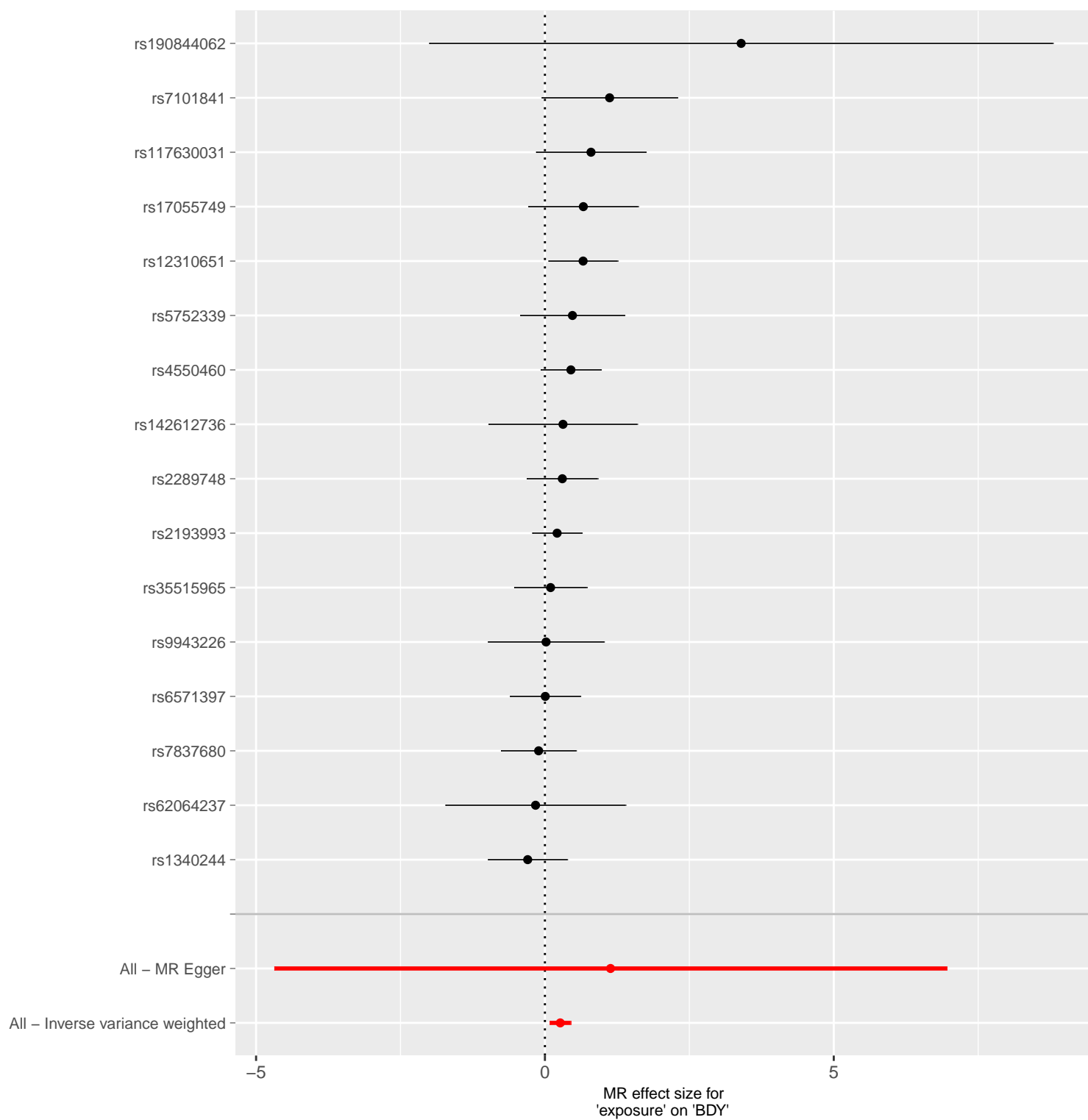

Supplement: Supplementary file 1 [file Data_Sheet_1.zip › Supplementary Materials/MR plots for tongue/Chronic sinusitis/s__Fusobacterium_vincentii_mgs_3469/forest.pdf]

# MR Method

- Inverse variance weighted
- MR Egger

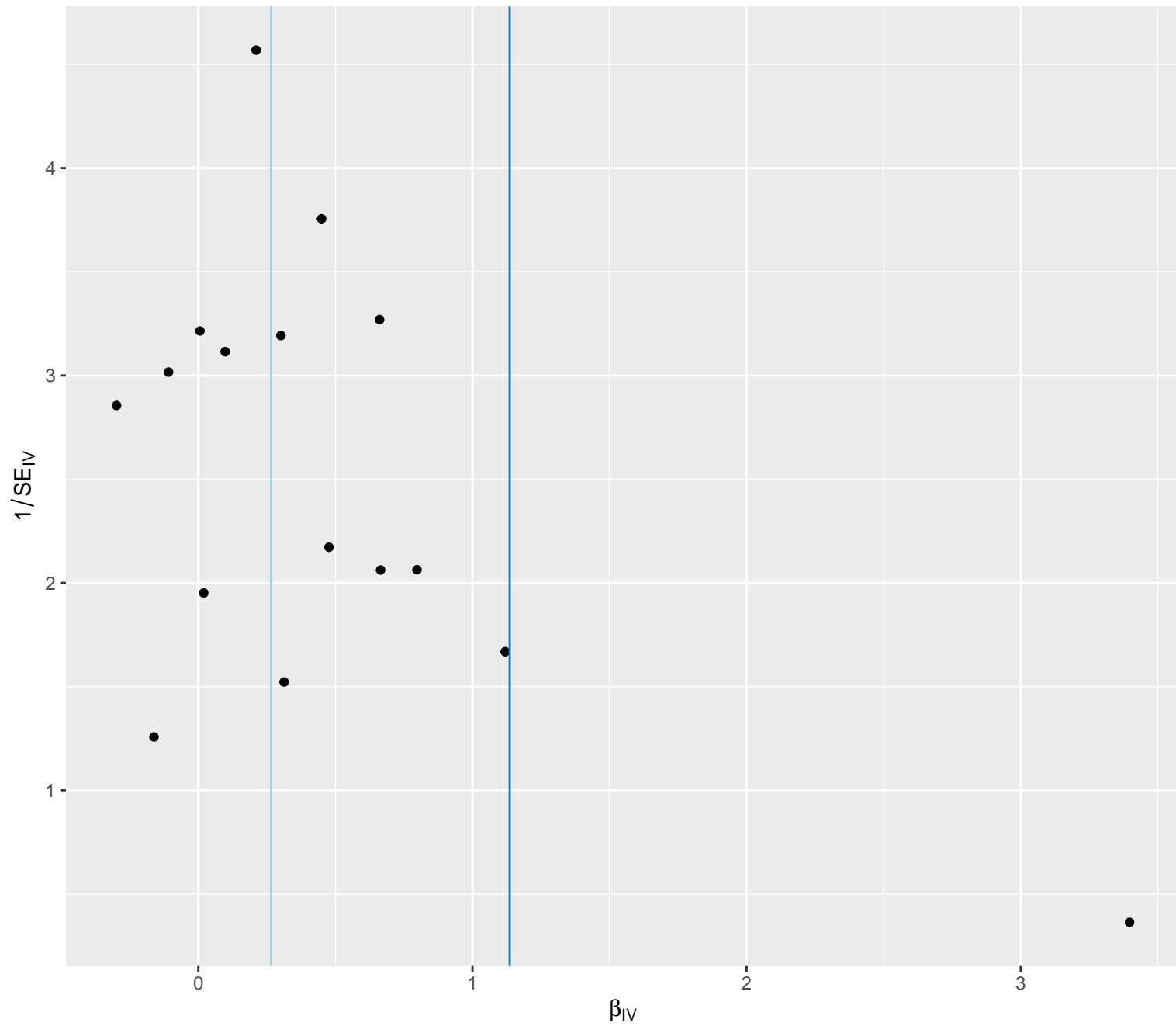

Supplement: Supplementary file 1 [file Data_Sheet_1.zip › Supplementary Materials/MR plots for tongue/Chronic sinusitis/s__Fusobacterium_vincentii_mgs_3469/funnel.pdf]

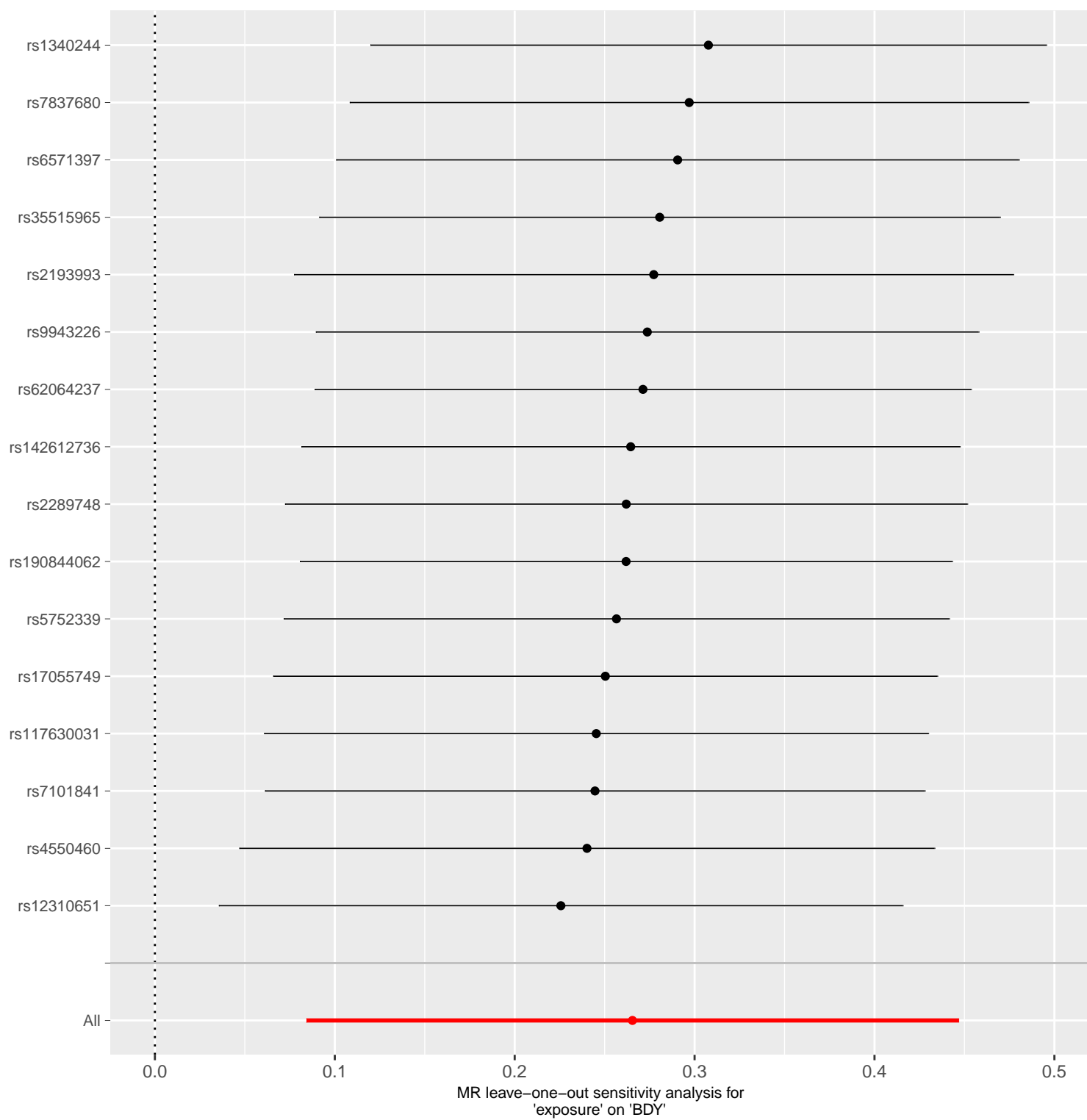

Supplement: Supplementary file 1 [file Data_Sheet_1.zip › Supplementary Materials/MR plots for tongue/Chronic sinusitis/s__Fusobacterium_vincentii_mgs_3469/leave_one_out.pdf]

# MR Test

- Inverse variance weighted
- MR Egger
- Weighted median

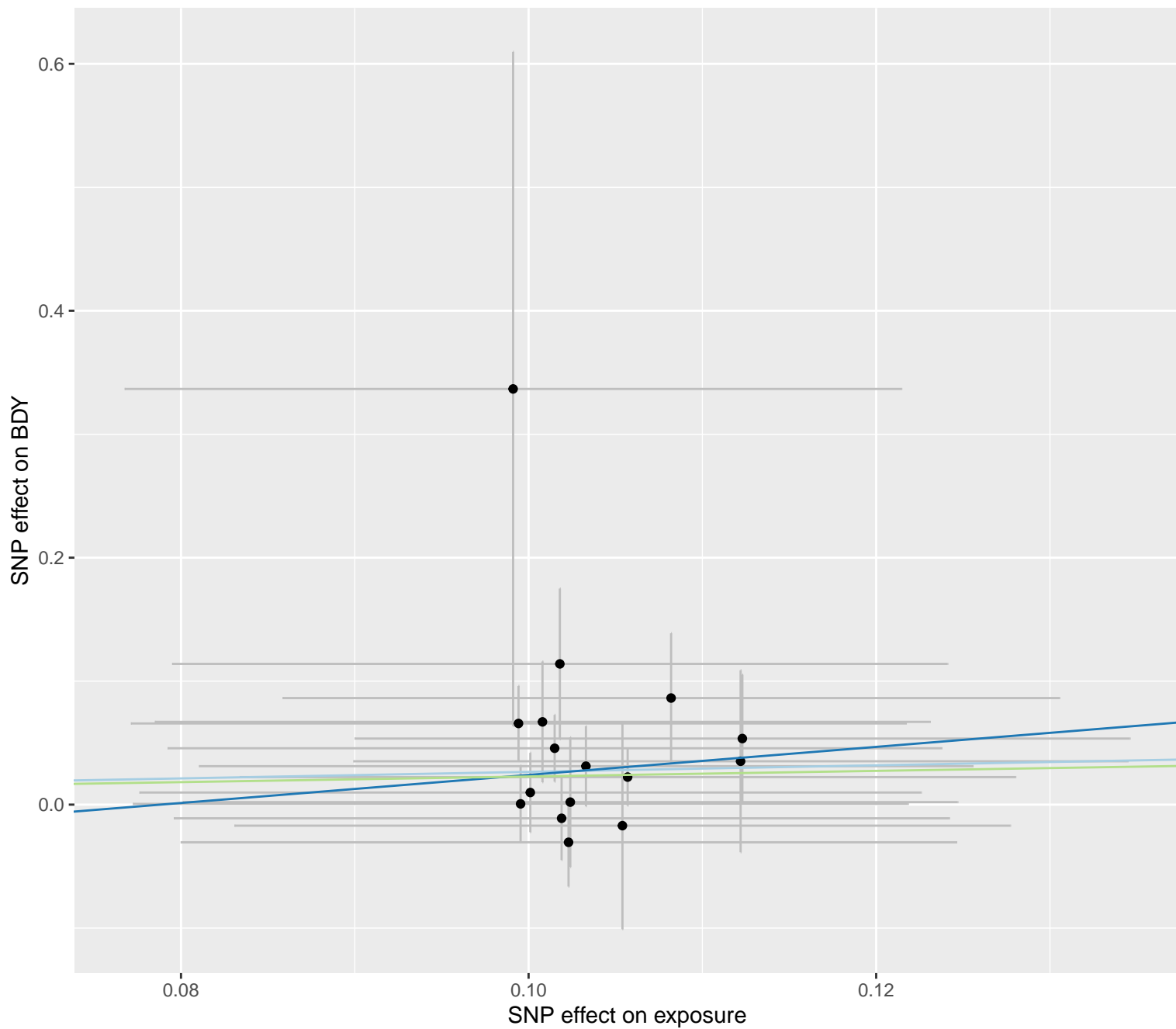

Supplement: Supplementary file 1 [file Data_Sheet_1.zip › Supplementary Materials/MR plots for tongue/Chronic sinusitis/s__Fusobacterium_vincentii_mgs_3469/scatter.pdf]

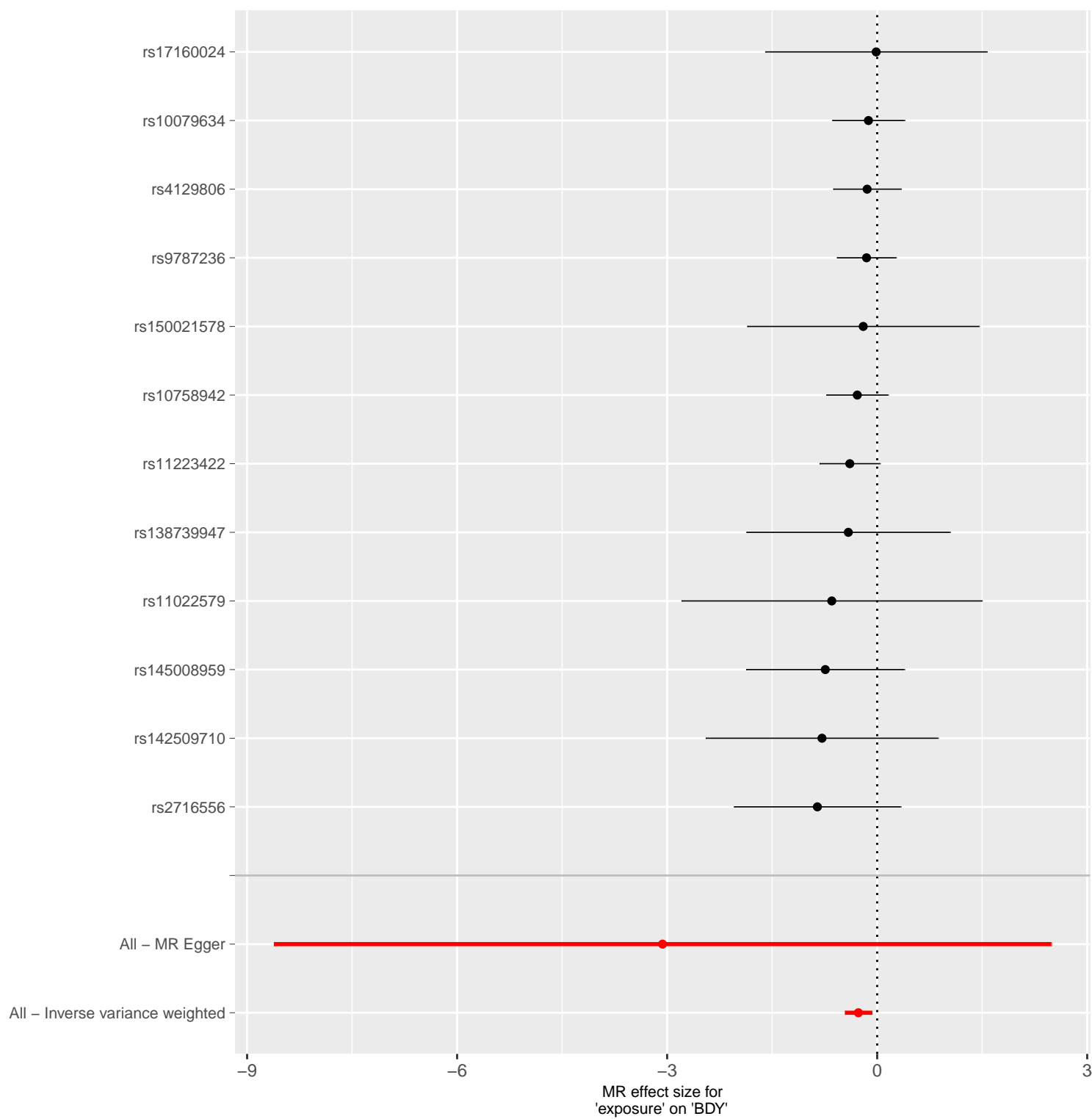

Supplement: Supplementary file 1 [file Data_Sheet_1.zip › Supplementary Materials/MR plots for tongue/Chronic sinusitis/s__Gemella_massiliensis_mgs_238/forest.pdf]

# MR Method

- Inverse variance weighted
- MR Egger

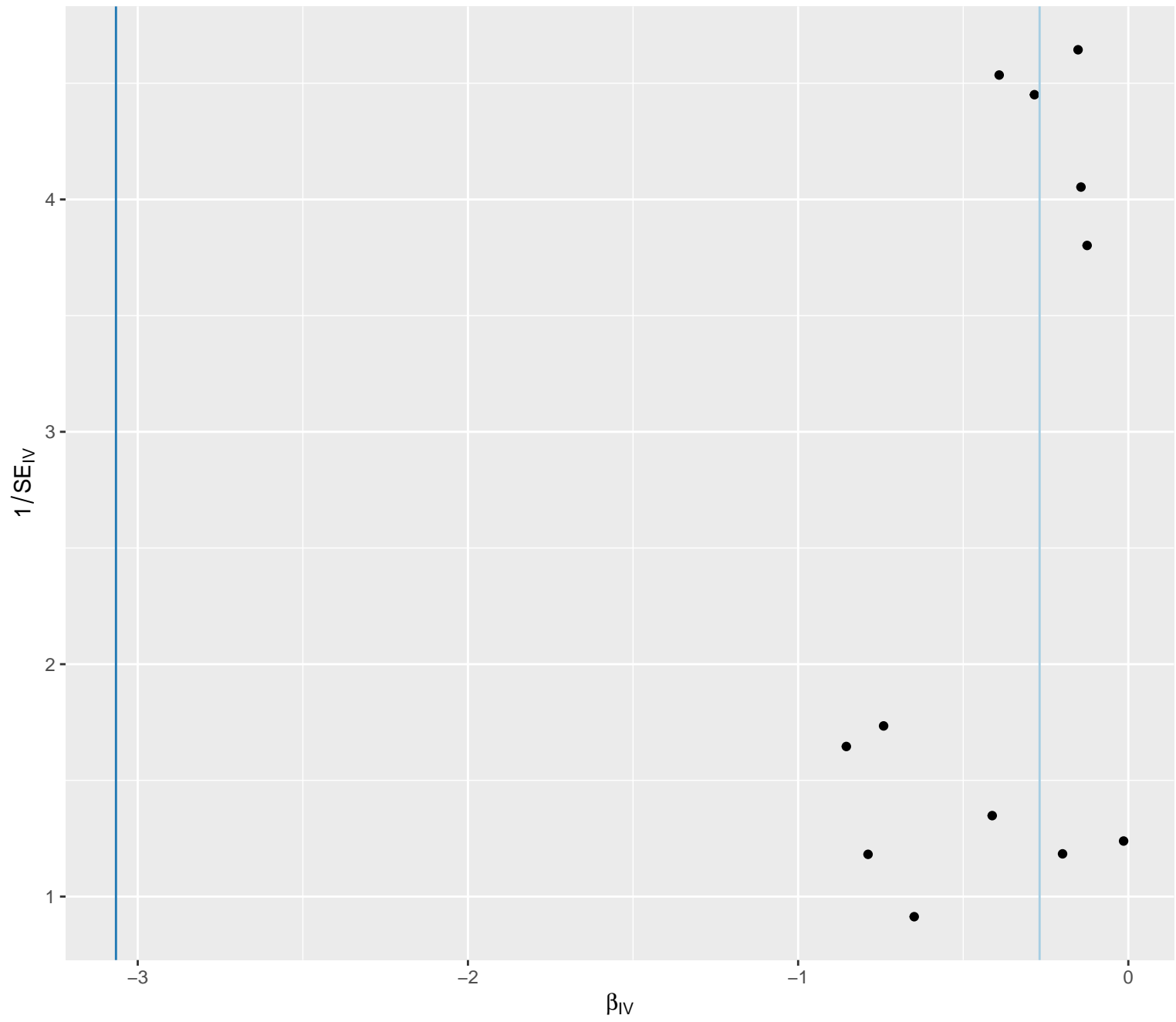

Supplement: Supplementary file 1 [file Data_Sheet_1.zip › Supplementary Materials/MR plots for tongue/Chronic sinusitis/s__Gemella_massiliensis_mgs_238/funnel.pdf]

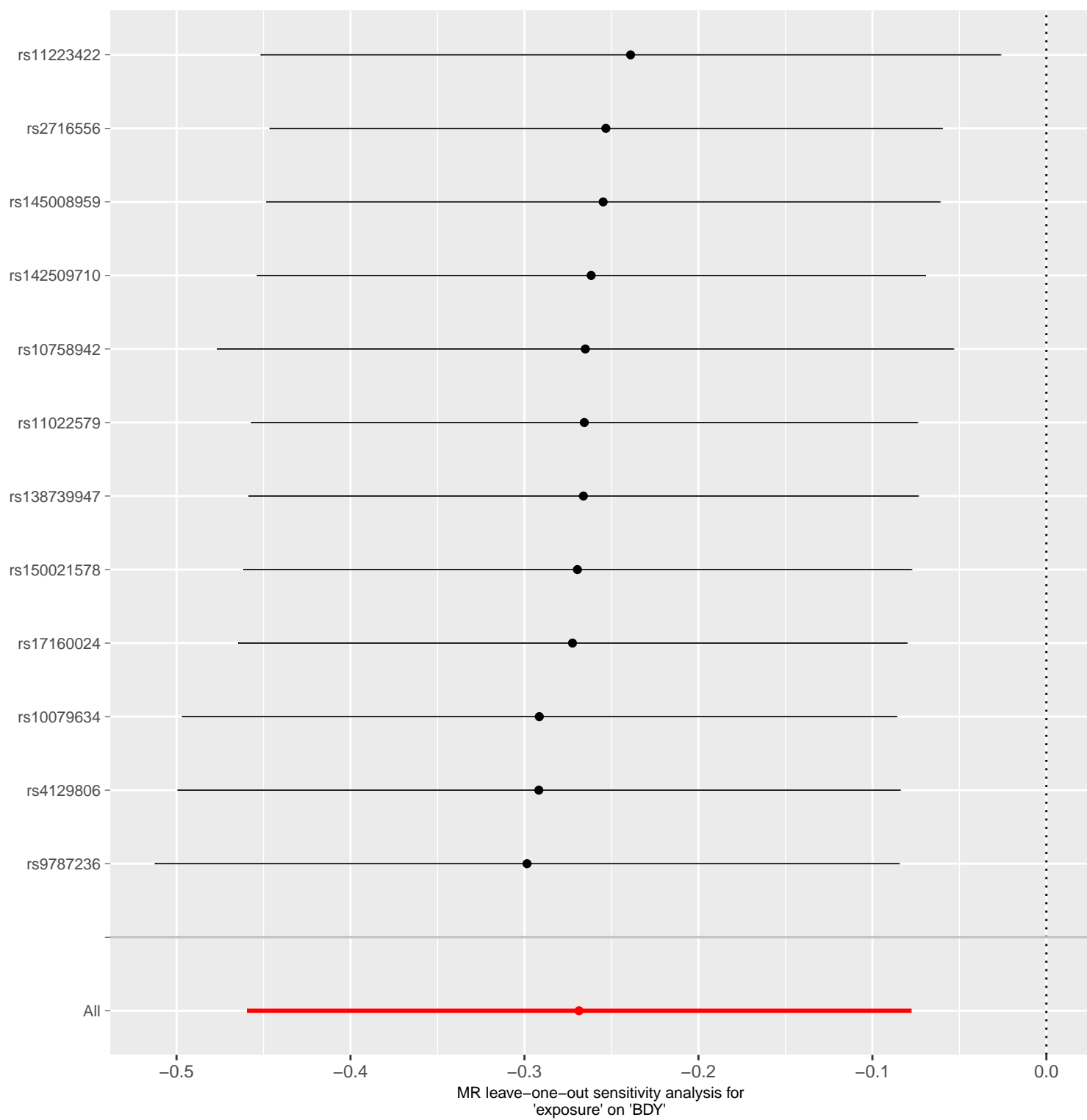

Supplement: Supplementary file 1 [file Data_Sheet_1.zip › Supplementary Materials/MR plots for tongue/Chronic sinusitis/s__Gemella_massiliensis_mgs_238/leave_one_out.pdf]

# MR Test

- Inverse variance weighted
- MR Egger
- Weighted median

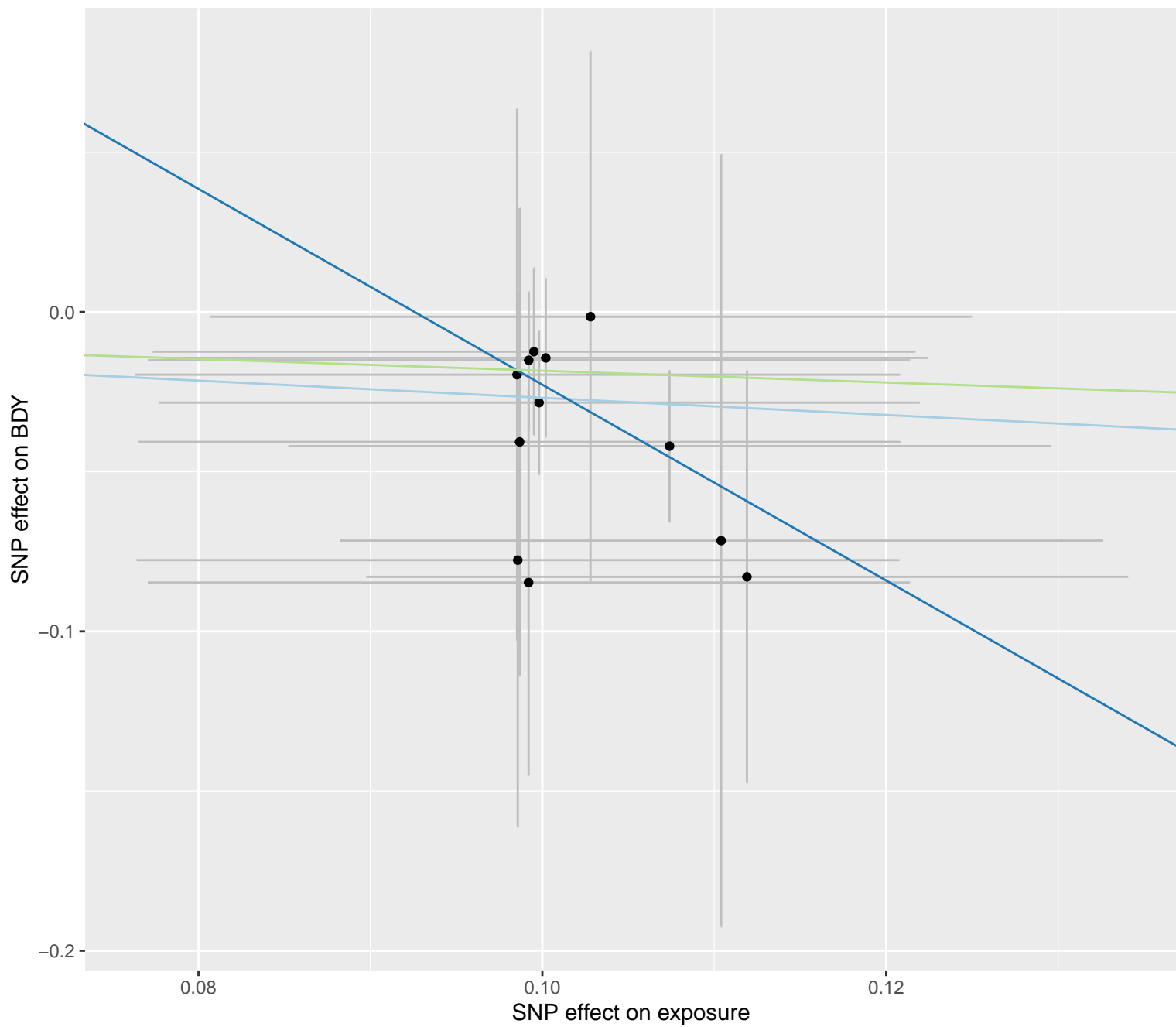

Supplement: Supplementary file 1 [file Data_Sheet_1.zip › Supplementary Materials/MR plots for tongue/Chronic sinusitis/s__Gemella_massiliensis_mgs_238/scatter.pdf]

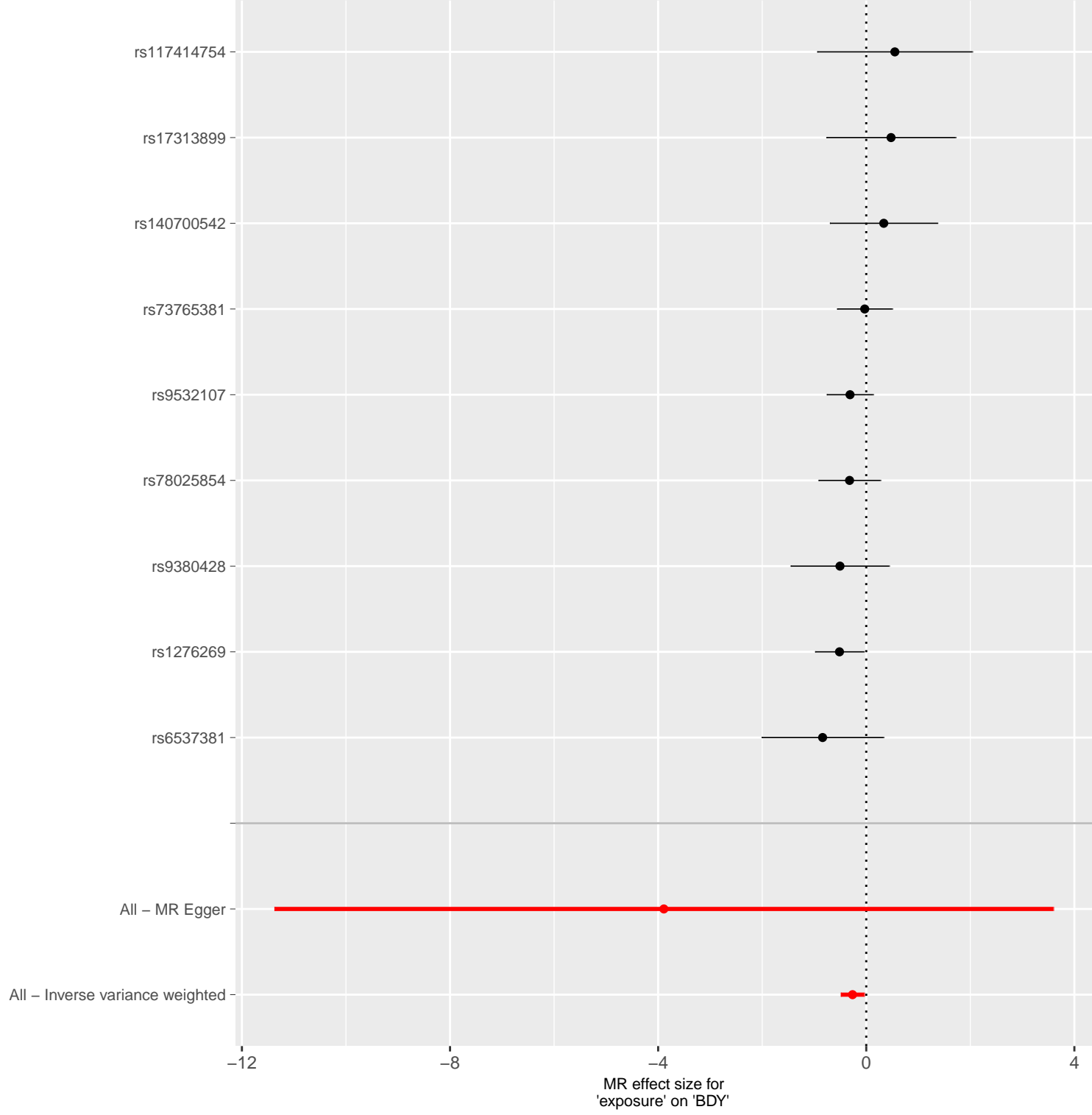

Supplement: Supplementary file 1 [file Data_Sheet_1.zip › Supplementary Materials/MR plots for tongue/Chronic sinusitis/s__Kingella_A_denitrificans_mgs_1934/forest.pdf]

# MR Method

- Inverse variance weighted
- MR Egger

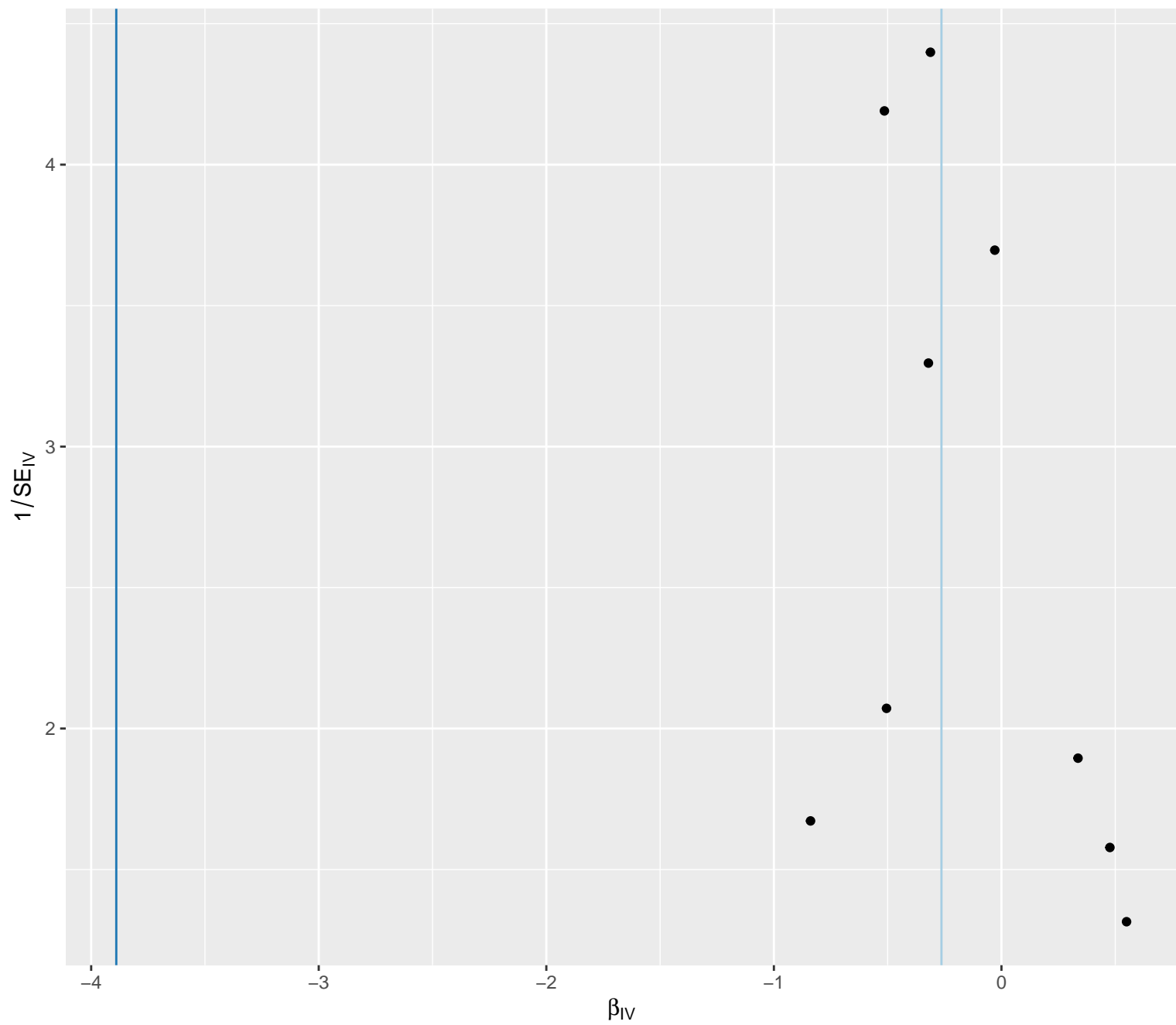

Supplement: Supplementary file 1 [file Data_Sheet_1.zip › Supplementary Materials/MR plots for tongue/Chronic sinusitis/s__Kingella_A_denitrificans_mgs_1934/funnel.pdf]

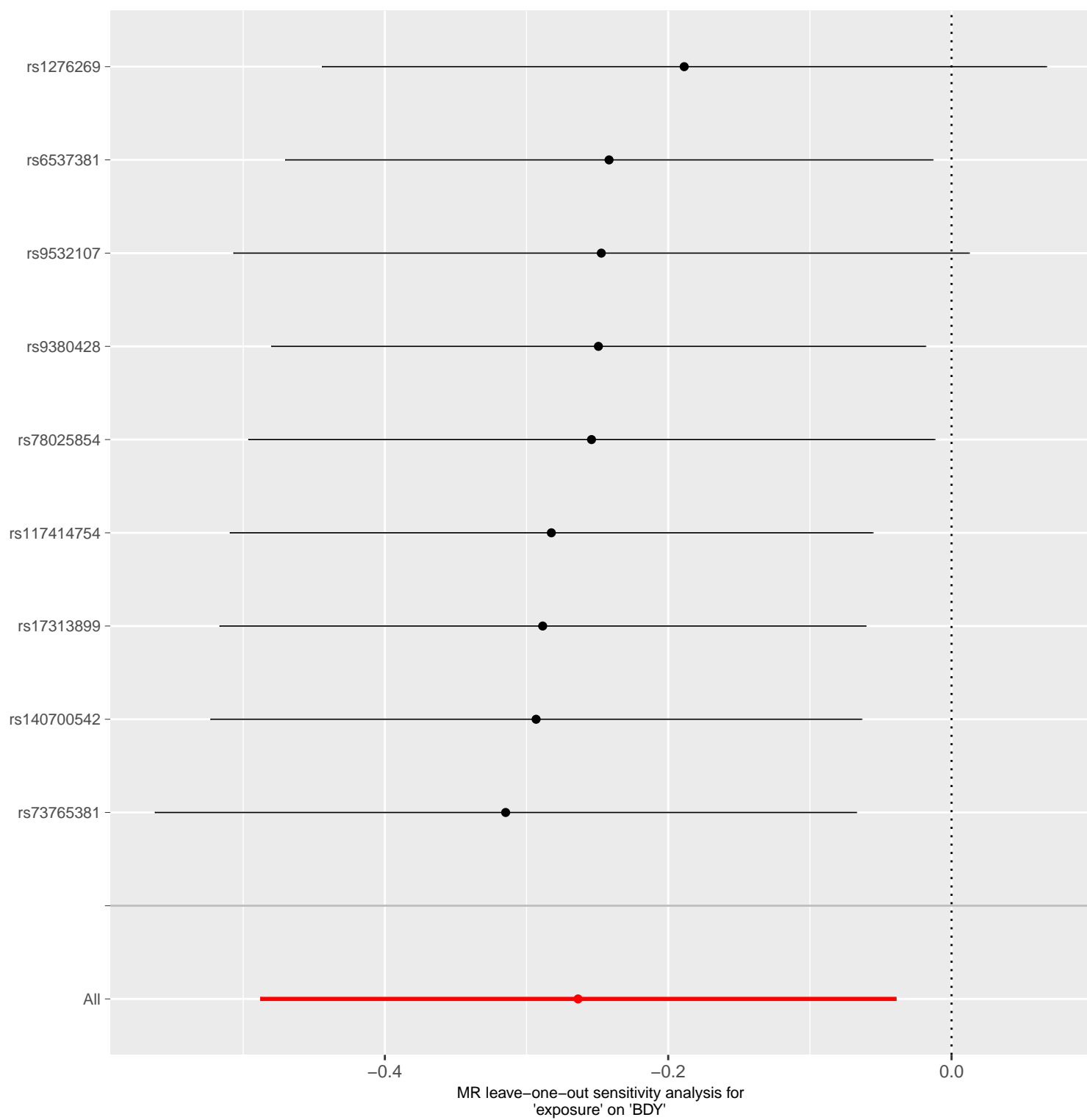

Supplement: Supplementary file 1 [file Data_Sheet_1.zip › Supplementary Materials/MR plots for tongue/Chronic sinusitis/s__Kingella_A_denitrificans_mgs_1934/leave_one_out.pdf]

# MR Test

- Inverse variance weighted
- MR Egger
- Weighted median

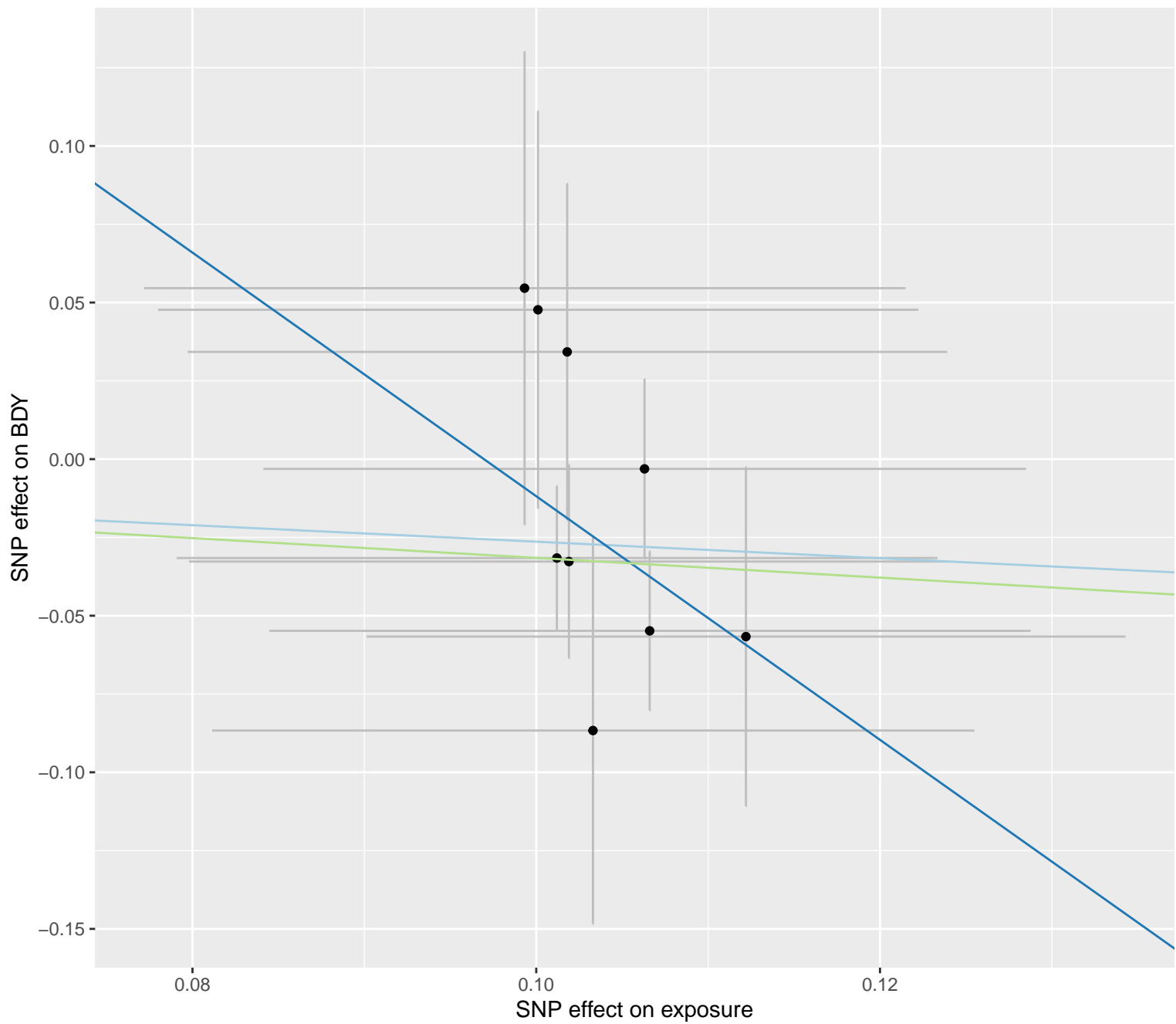

Supplement: Supplementary file 1 [file Data_Sheet_1.zip › Supplementary Materials/MR plots for tongue/Chronic sinusitis/s__Kingella_A_denitrificans_mgs_1934/scatter.pdf]

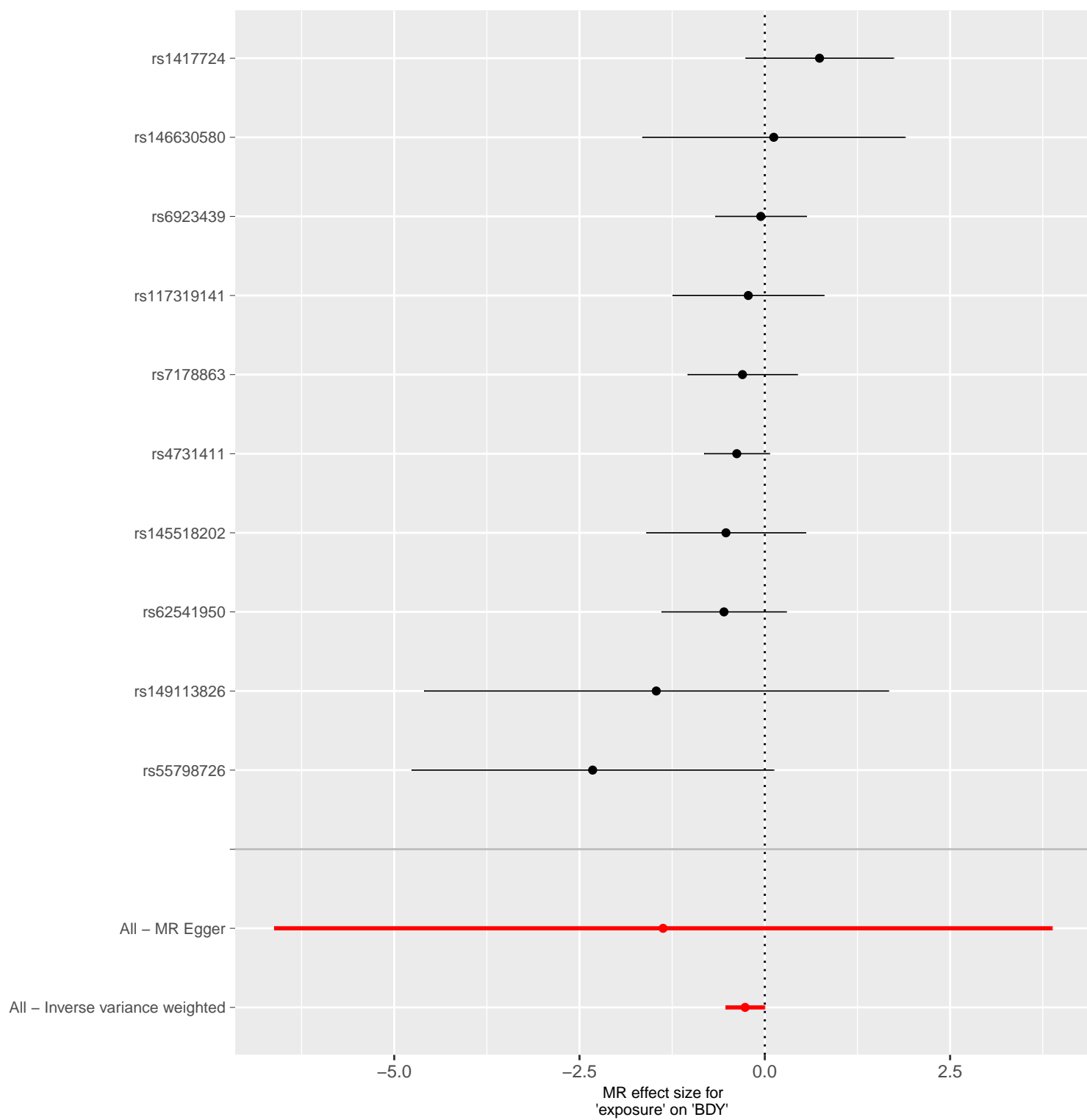

Supplement: Supplementary file 1 [file Data_Sheet_1.zip › Supplementary Materials/MR plots for tongue/Chronic sinusitis/s__Prevotella_conceptionensis_mgs_303/forest.pdf]

# MR Method

- Inverse variance weighted
- MR Egger

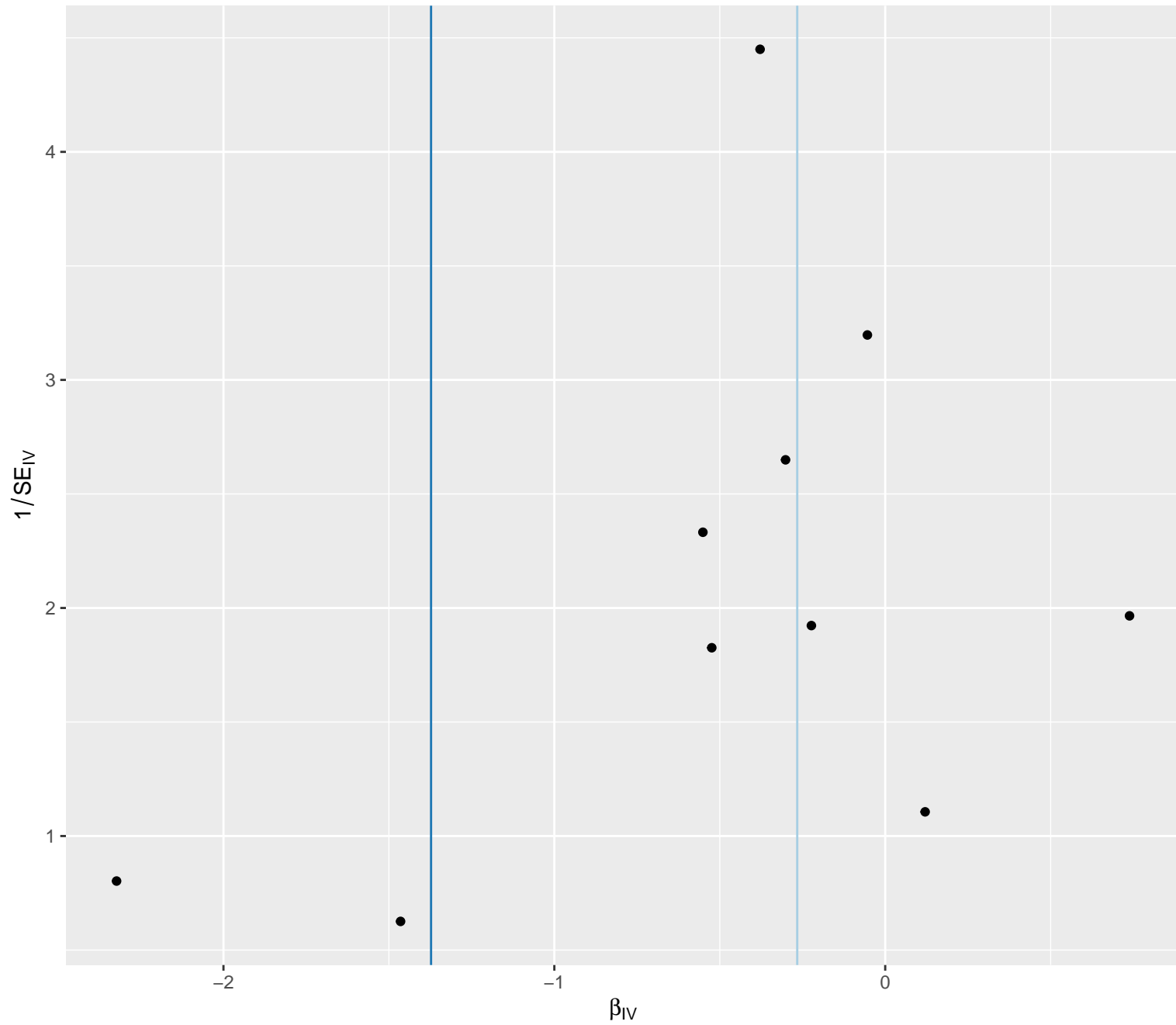

Supplement: Supplementary file 1 [file Data_Sheet_1.zip › Supplementary Materials/MR plots for tongue/Chronic sinusitis/s__Prevotella_conceptionensis_mgs_303/funnel.pdf]

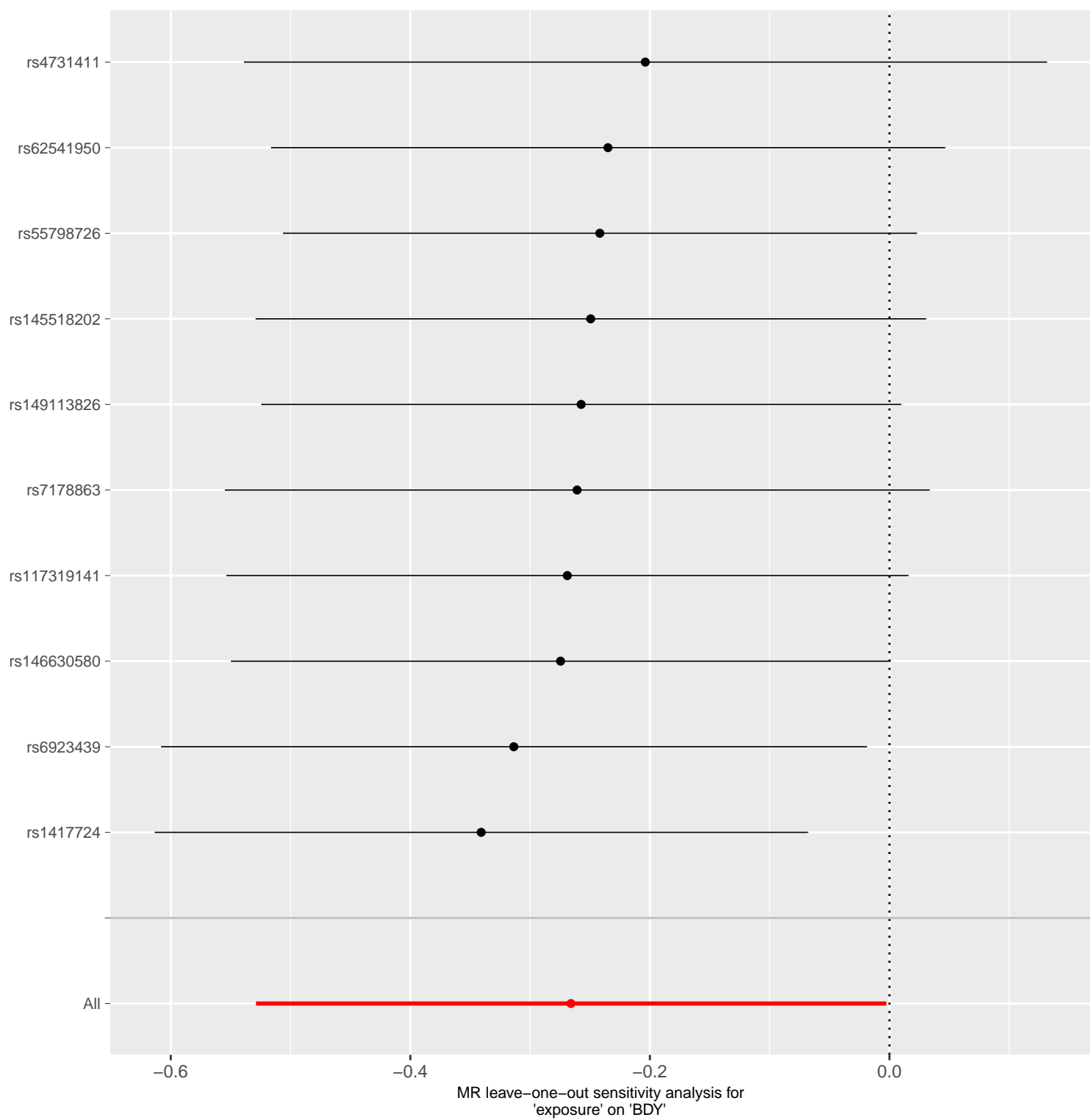

Supplement: Supplementary file 1 [file Data_Sheet_1.zip › Supplementary Materials/MR plots for tongue/Chronic sinusitis/s__Prevotella_conceptionensis_mgs_303/leave_one_out.pdf]

# MR Test

- Inverse variance weighted
- MR Egger
- Weighted median

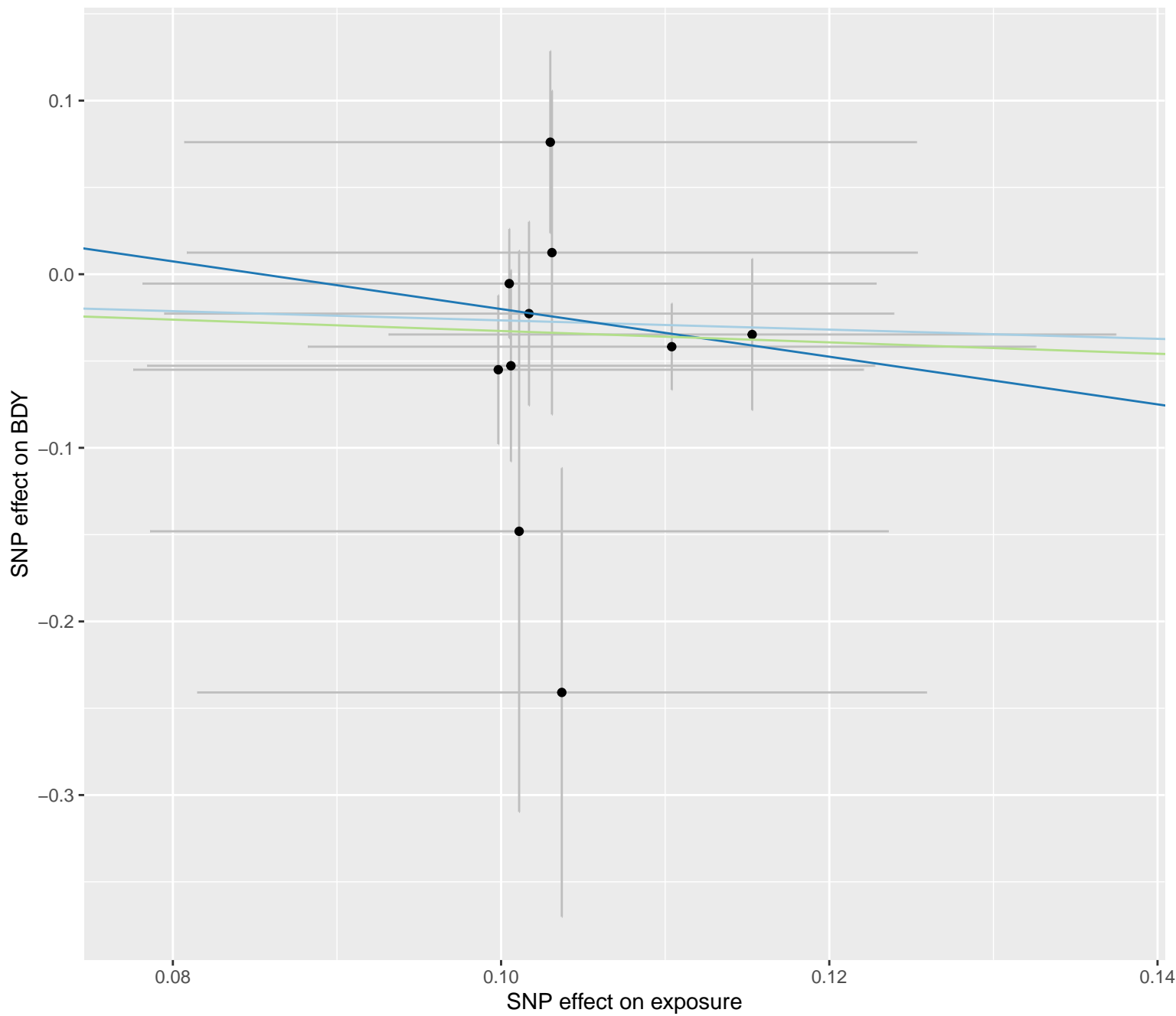

Supplement: Supplementary file 1 [file Data_Sheet_1.zip › Supplementary Materials/MR plots for tongue/Chronic sinusitis/s__Prevotella_conceptionensis_mgs_303/scatter.pdf]

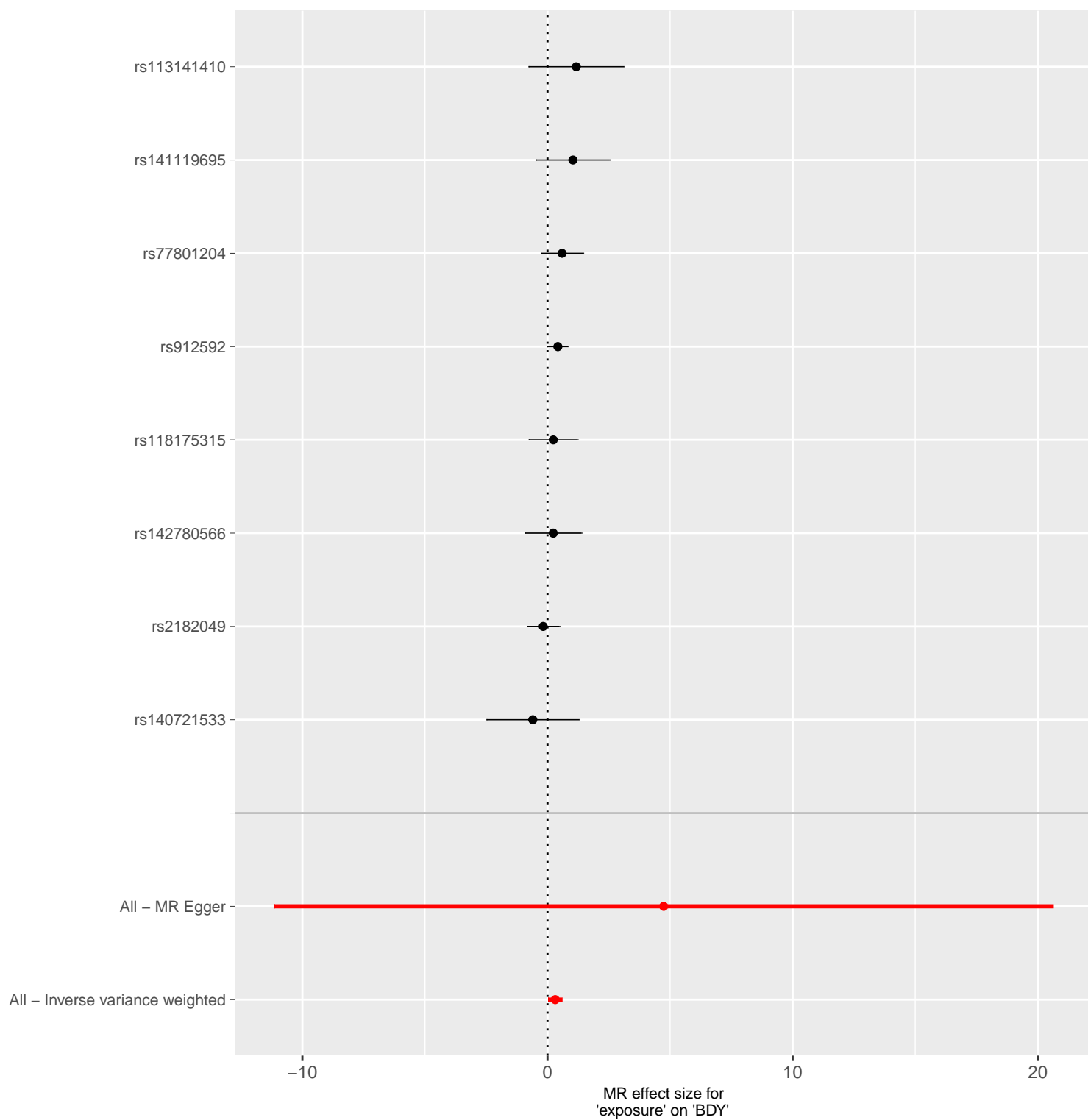

Supplement: Supplementary file 1 [file Data_Sheet_1.zip › Supplementary Materials/MR plots for tongue/Chronic sinusitis/s__Prevotella_nigrescens_mgs_3566/forest.pdf]

# MR Method

- Inverse variance weighted
- MR Egger

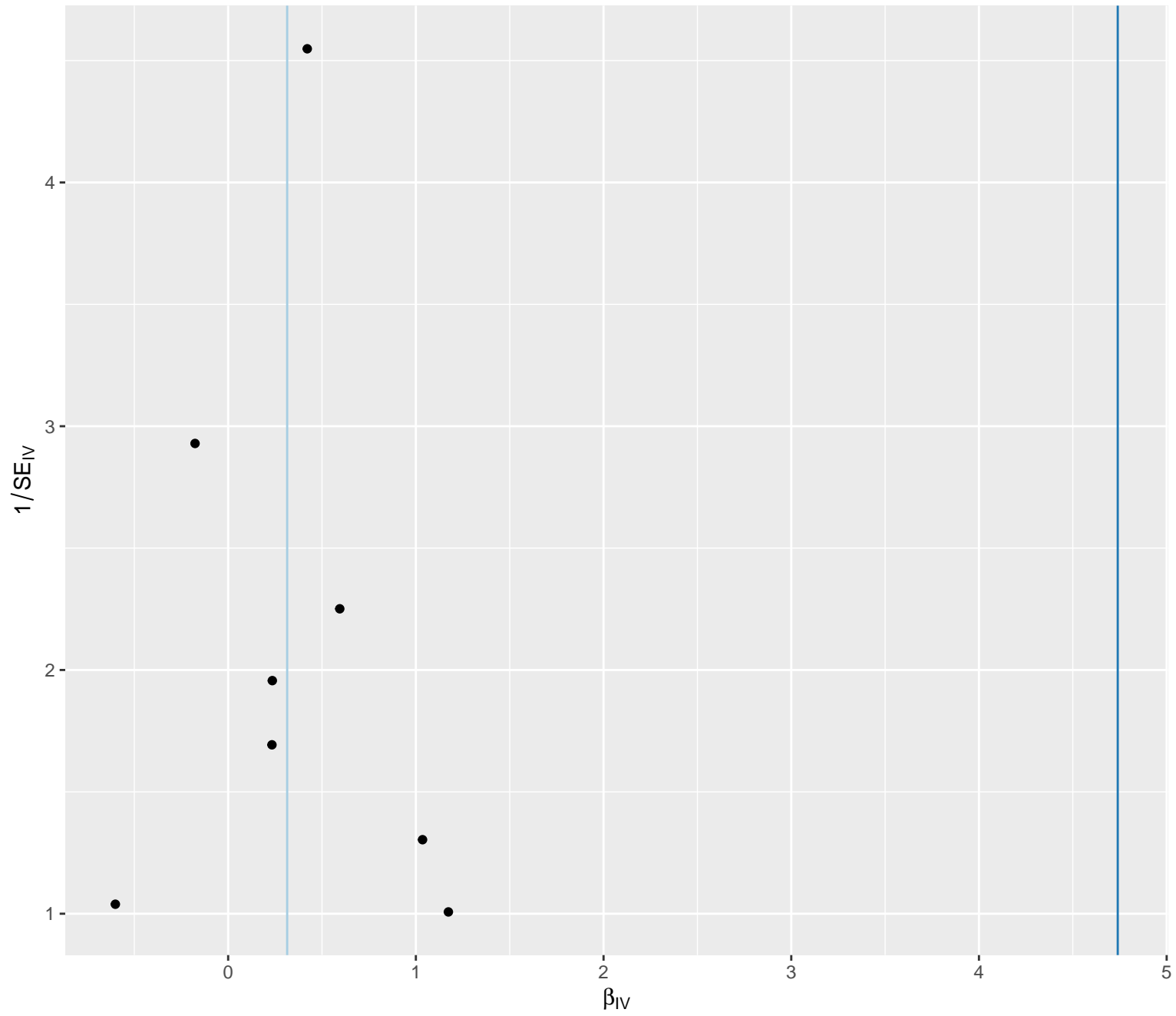

Supplement: Supplementary file 1 [file Data_Sheet_1.zip › Supplementary Materials/MR plots for tongue/Chronic sinusitis/s__Prevotella_nigrescens_mgs_3566/funnel.pdf]

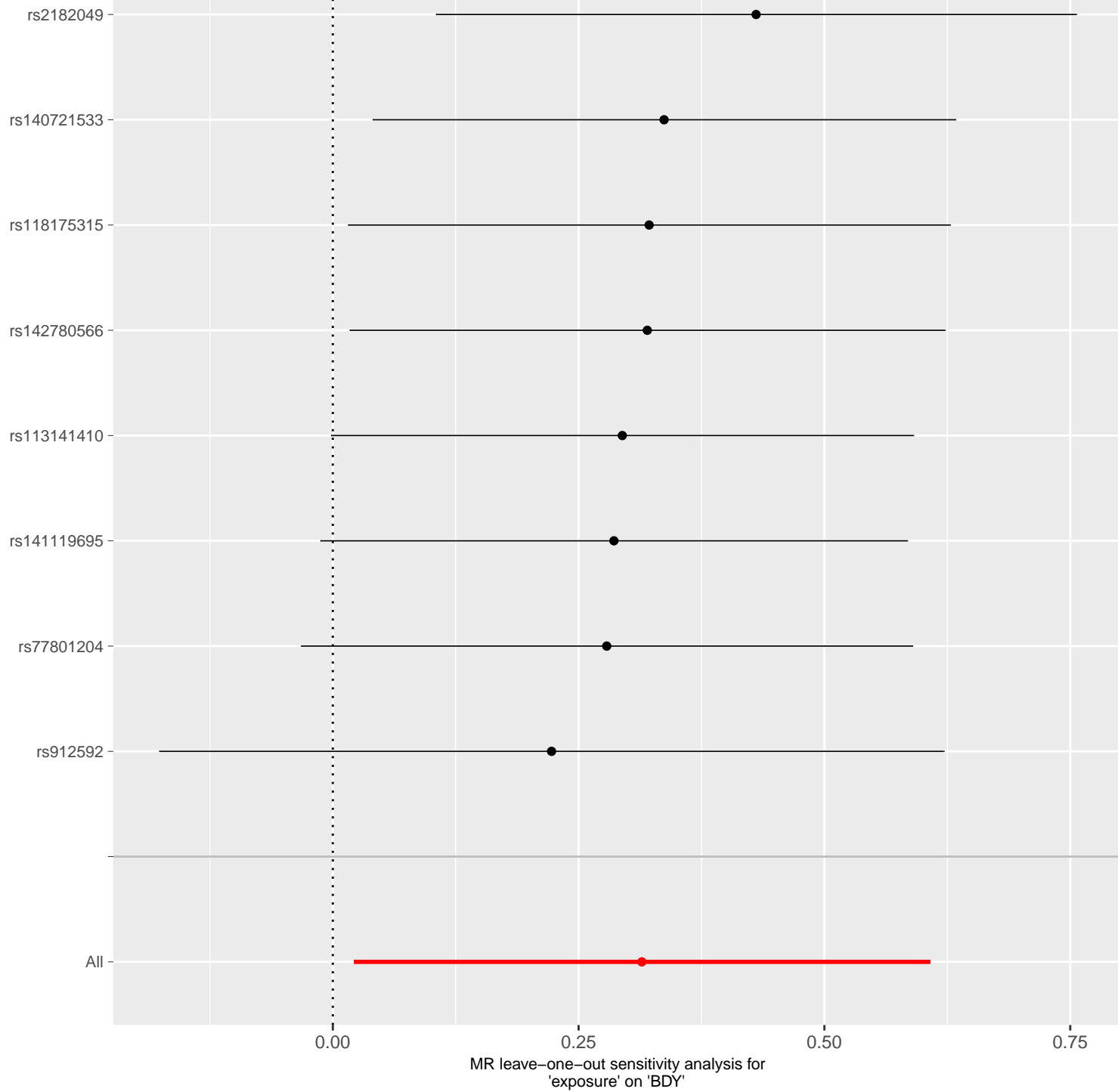

Supplement: Supplementary file 1 [file Data_Sheet_1.zip › Supplementary Materials/MR plots for tongue/Chronic sinusitis/s__Prevotella_nigrescens_mgs_3566/leave_one_out.pdf]

# MR Test

- Inverse variance weighted
- MR Egger
- Weighted median

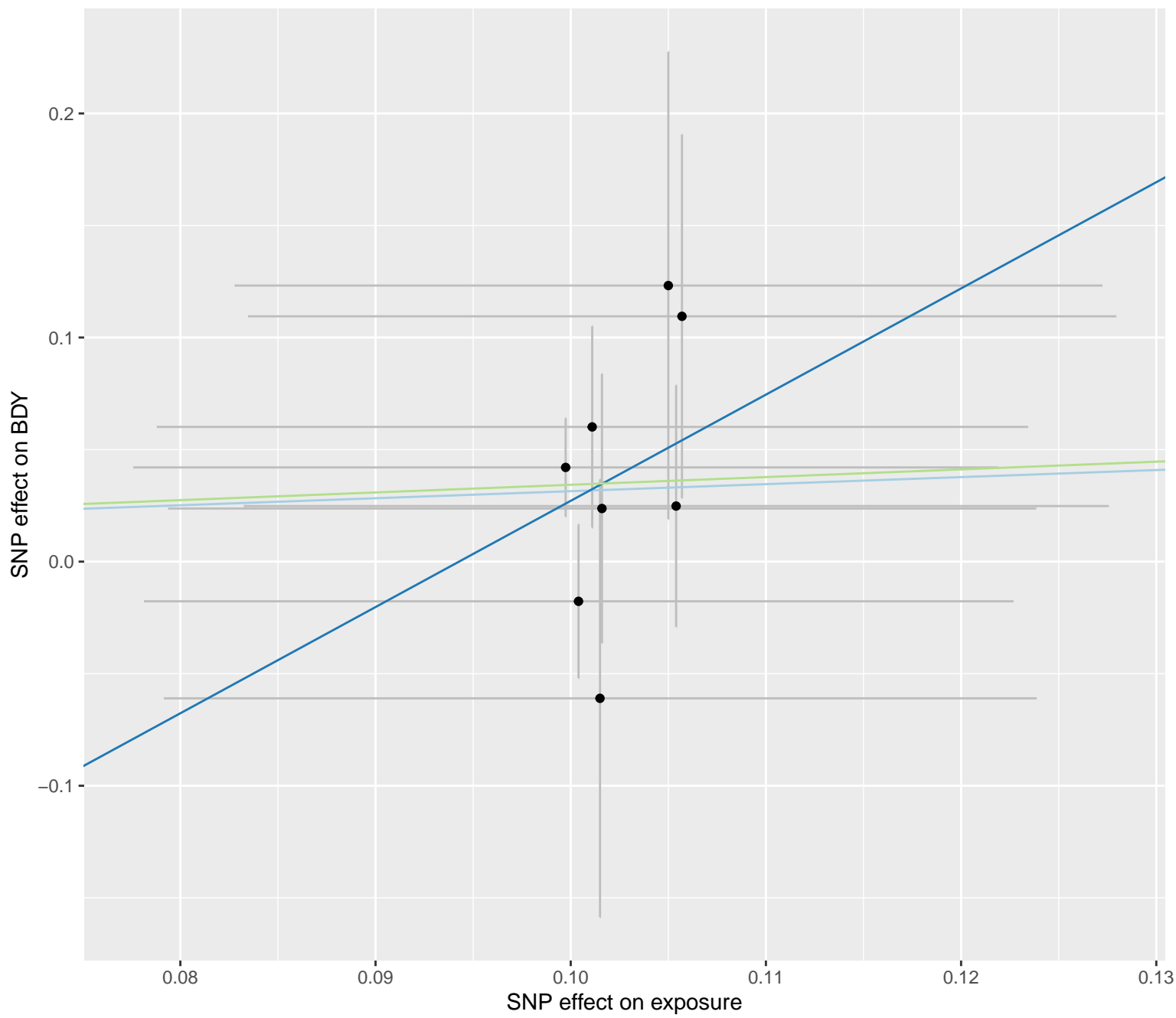

Supplement: Supplementary file 1 [file Data_Sheet_1.zip › Supplementary Materials/MR plots for tongue/Chronic sinusitis/s__Prevotella_nigrescens_mgs_3566/scatter.pdf]

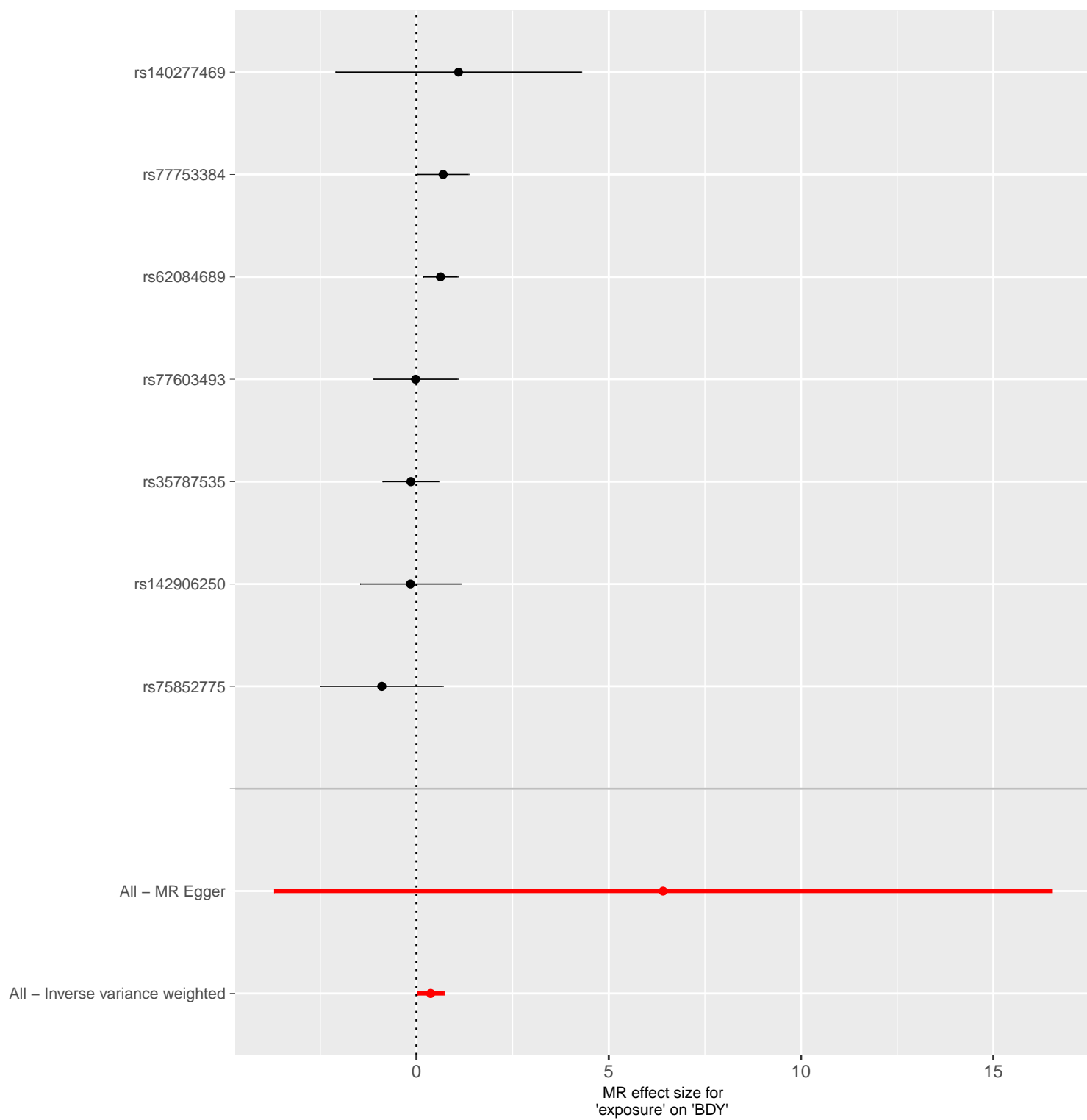

Supplement: Supplementary file 1 [file Data_Sheet_1.zip › Supplementary Materials/MR plots for tongue/Chronic sinusitis/s__Rothia_mucilaginosa_mgs_3219/forest.pdf]

# MR Method

- Inverse variance weighted
- MR Egger

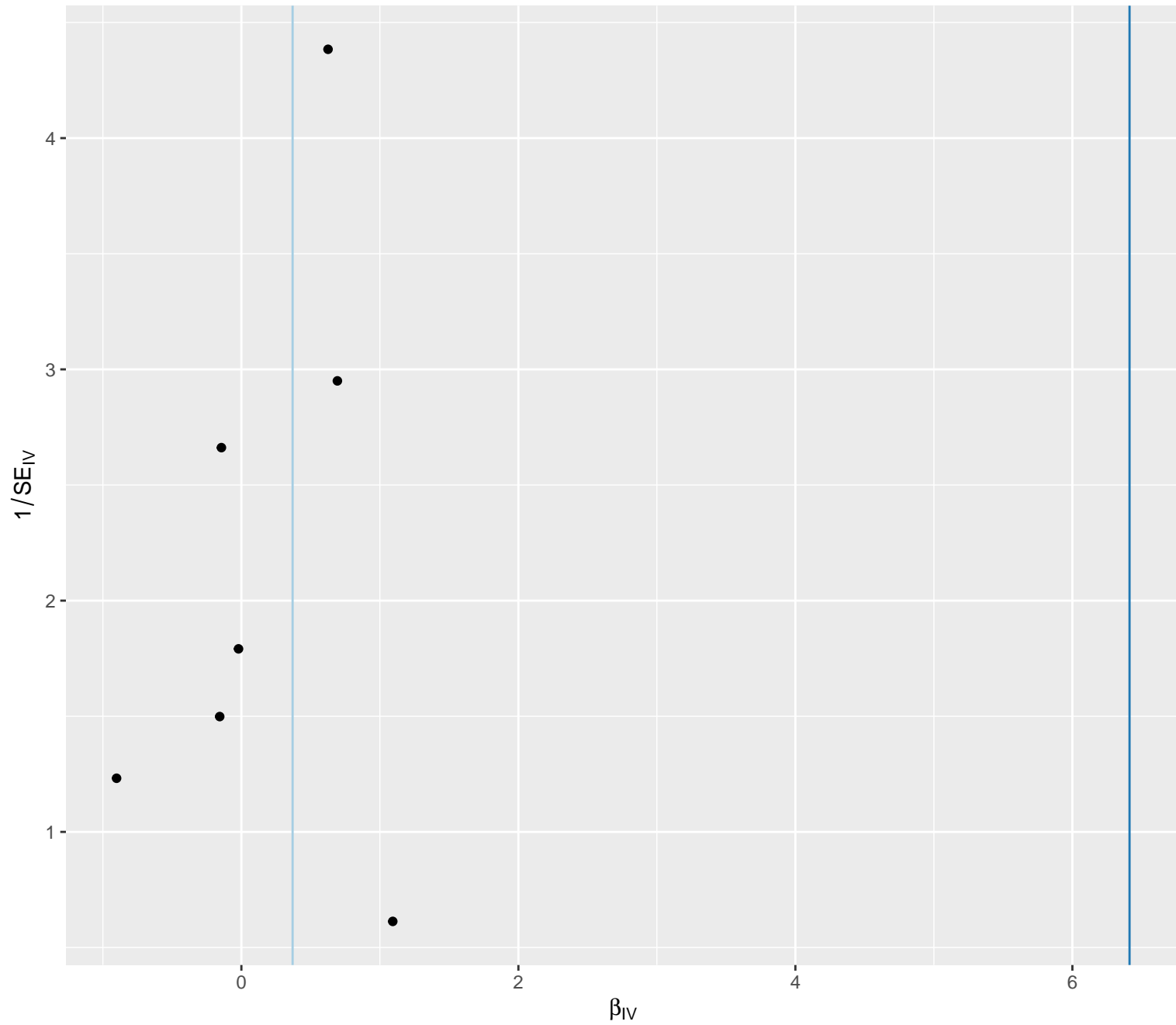

Supplement: Supplementary file 1 [file Data_Sheet_1.zip › Supplementary Materials/MR plots for tongue/Chronic sinusitis/s__Rothia_mucilaginosa_mgs_3219/funnel.pdf]

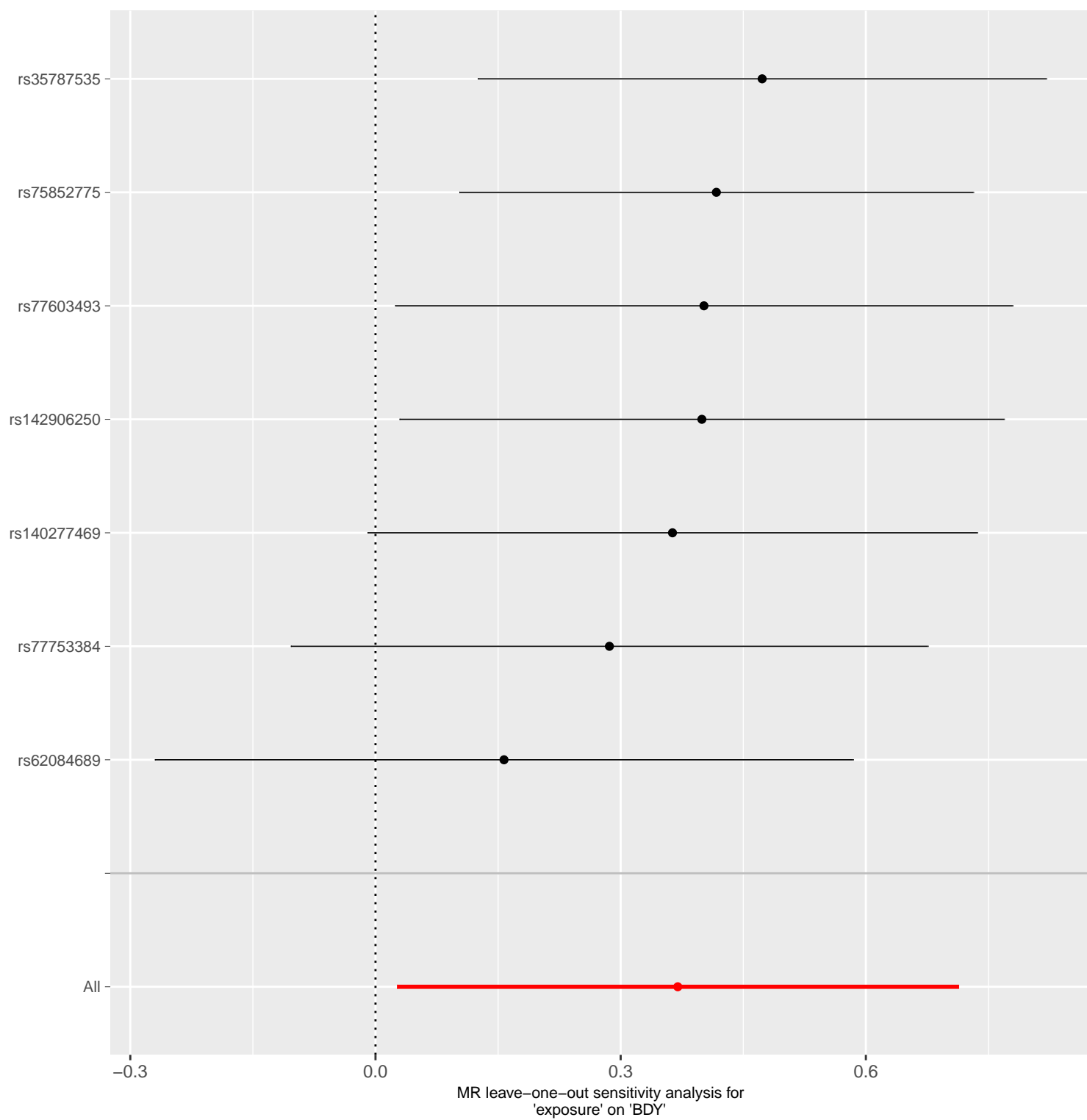

Supplement: Supplementary file 1 [file Data_Sheet_1.zip › Supplementary Materials/MR plots for tongue/Chronic sinusitis/s__Rothia_mucilaginosa_mgs_3219/leave_one_out.pdf]

# MR Test

- Inverse variance weighted
- MR Egger
- Weighted median

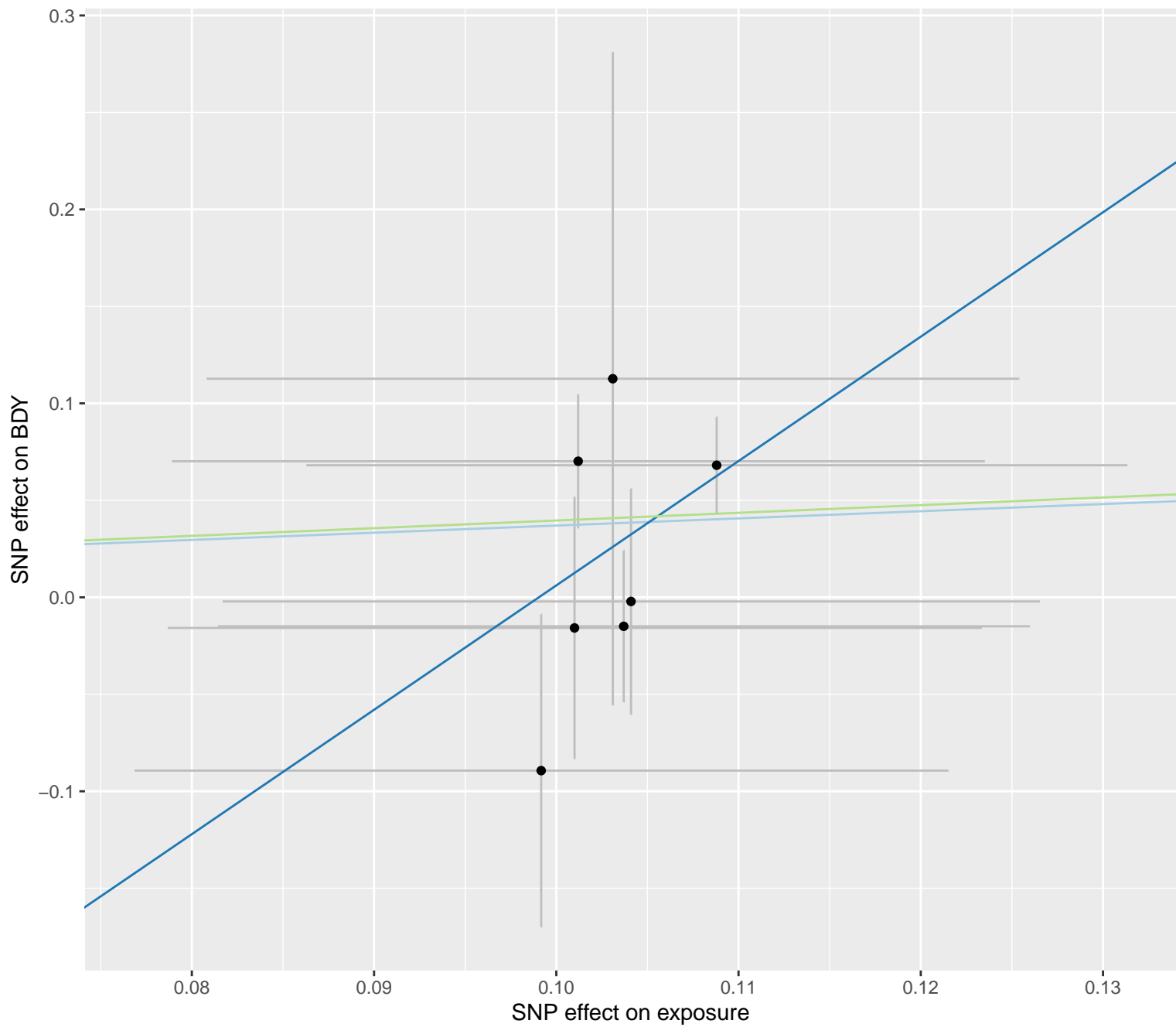

Supplement: Supplementary file 1 [file Data_Sheet_1.zip › Supplementary Materials/MR plots for tongue/Chronic sinusitis/s__Rothia_mucilaginosa_mgs_3219/scatter.pdf]

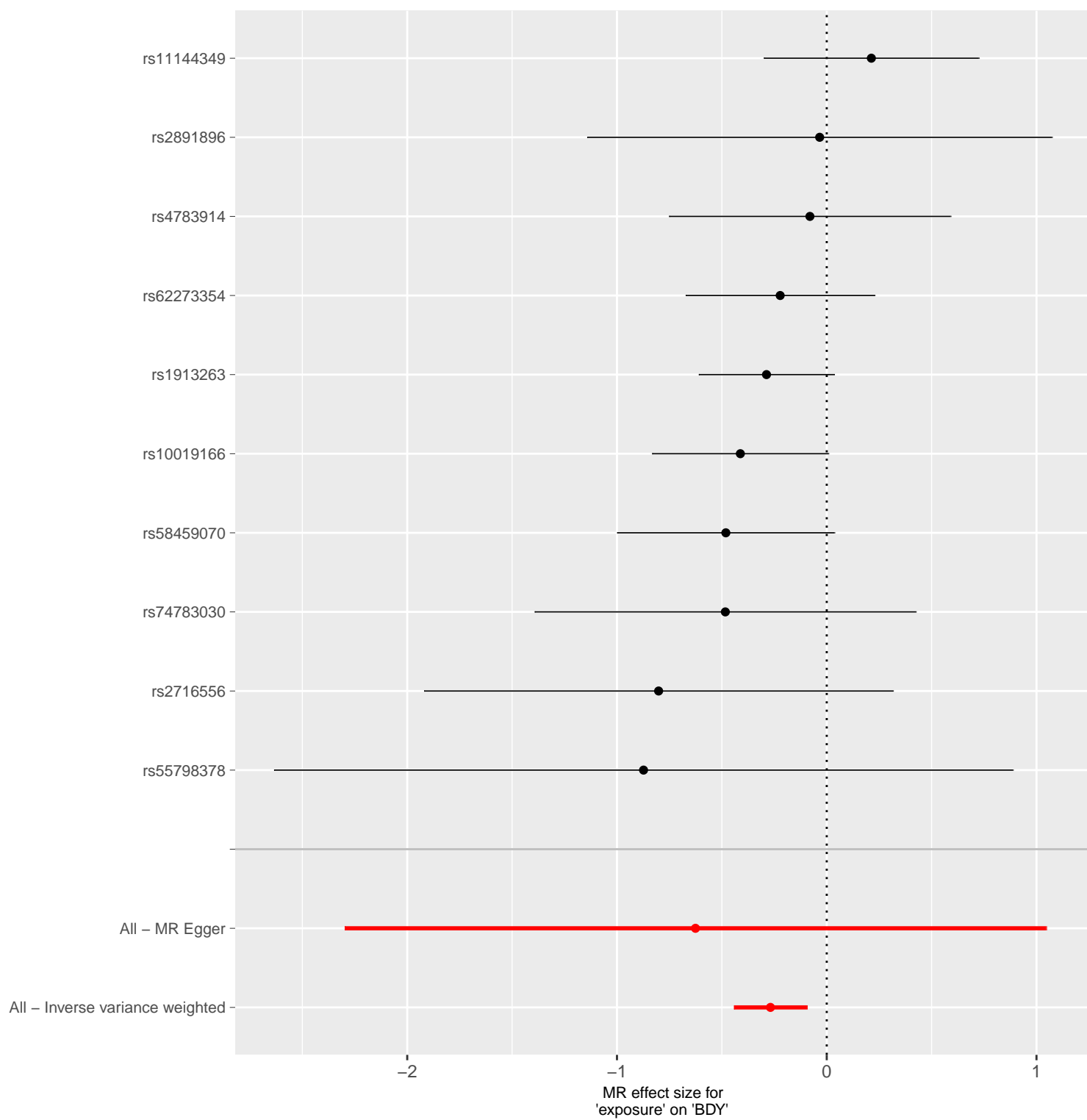

Supplement: Supplementary file 1 [file Data_Sheet_1.zip › Supplementary Materials/MR plots for tongue/Chronic sinusitis/s__Streptococcus_infantis_mgs_1655/forest.pdf]

# MR Method

- Inverse variance weighted
- MR Egger

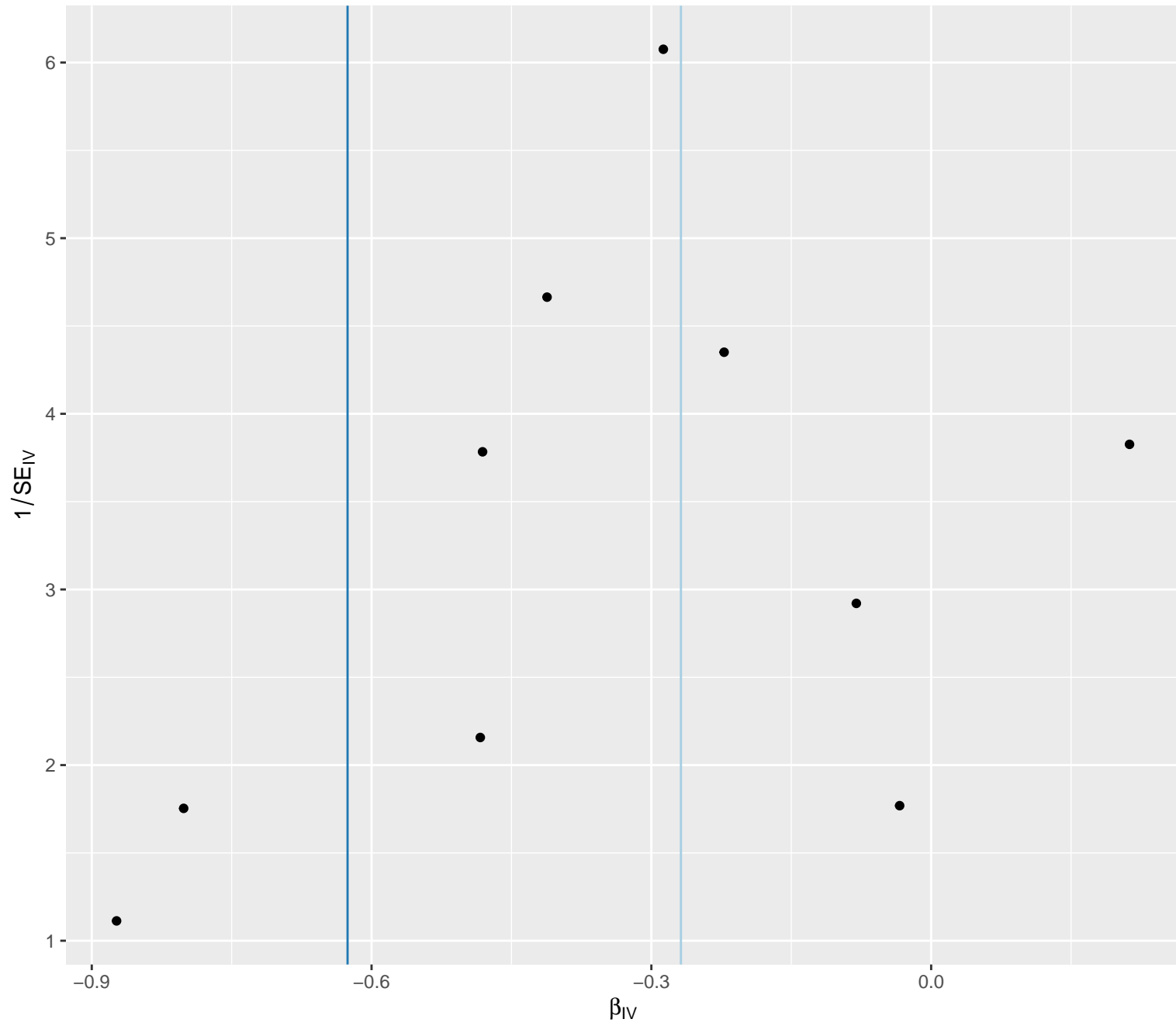

Supplement: Supplementary file 1 [file Data_Sheet_1.zip › Supplementary Materials/MR plots for tongue/Chronic sinusitis/s__Streptococcus_infantis_mgs_1655/funnel.pdf]

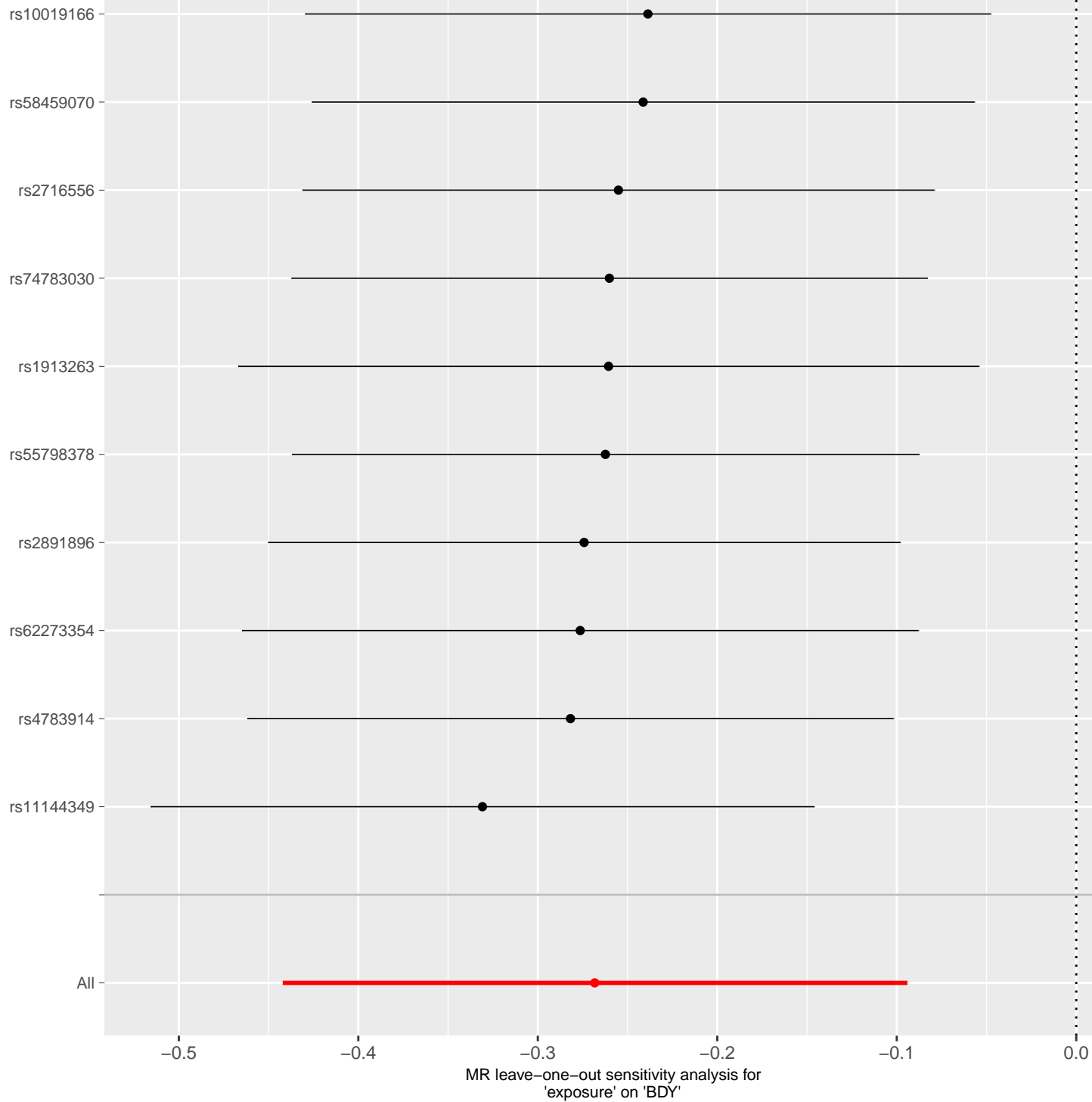

Supplement: Supplementary file 1 [file Data_Sheet_1.zip › Supplementary Materials/MR plots for tongue/Chronic sinusitis/s__Streptococcus_infantis_mgs_1655/leave_one_out.pdf]

# MR Test

- Inverse variance weighted
- MR Egger
- Weighted median

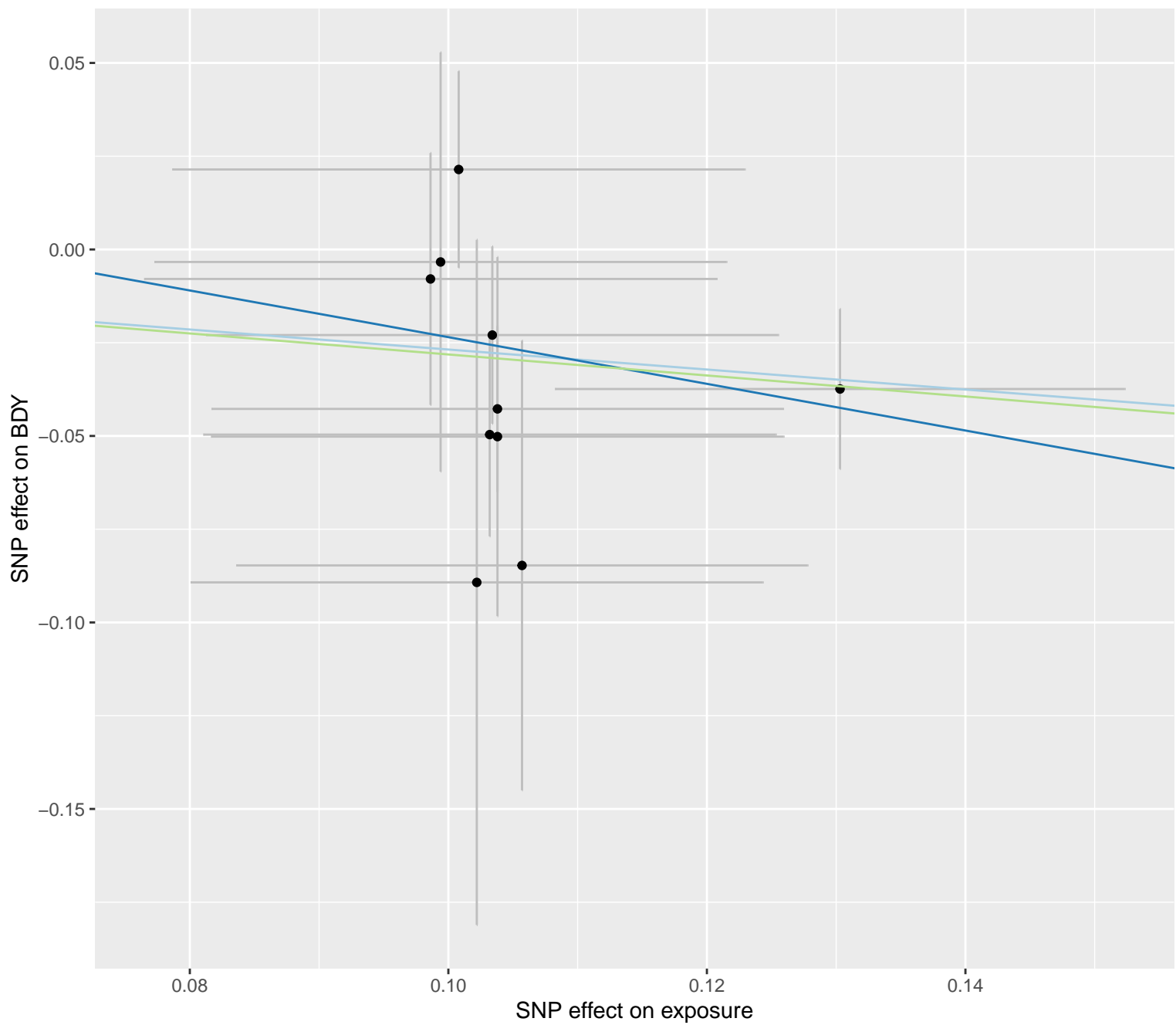

Supplement: Supplementary file 1 [file Data_Sheet_1.zip › Supplementary Materials/MR plots for tongue/Chronic sinusitis/s__Streptococcus_infantis_mgs_1655/scatter.pdf]

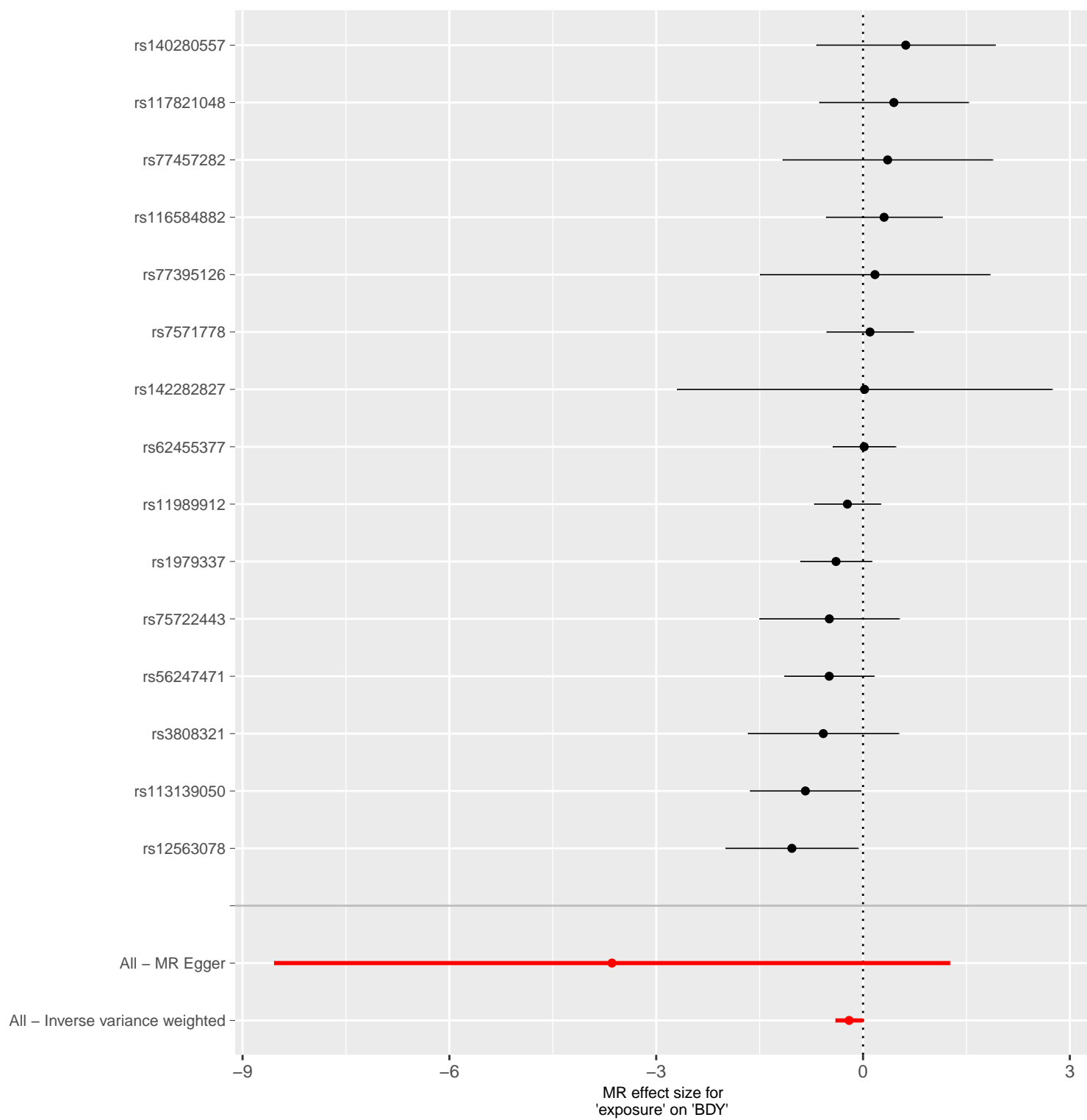

Supplement: Supplementary file 1 [file Data_Sheet_1.zip › Supplementary Materials/MR plots for tongue/Chronic sinusitis/s__Streptococcus_mitis_AT_mgs_2307/forest.pdf]

# MR Method

- Inverse variance weighted
- MR Egger

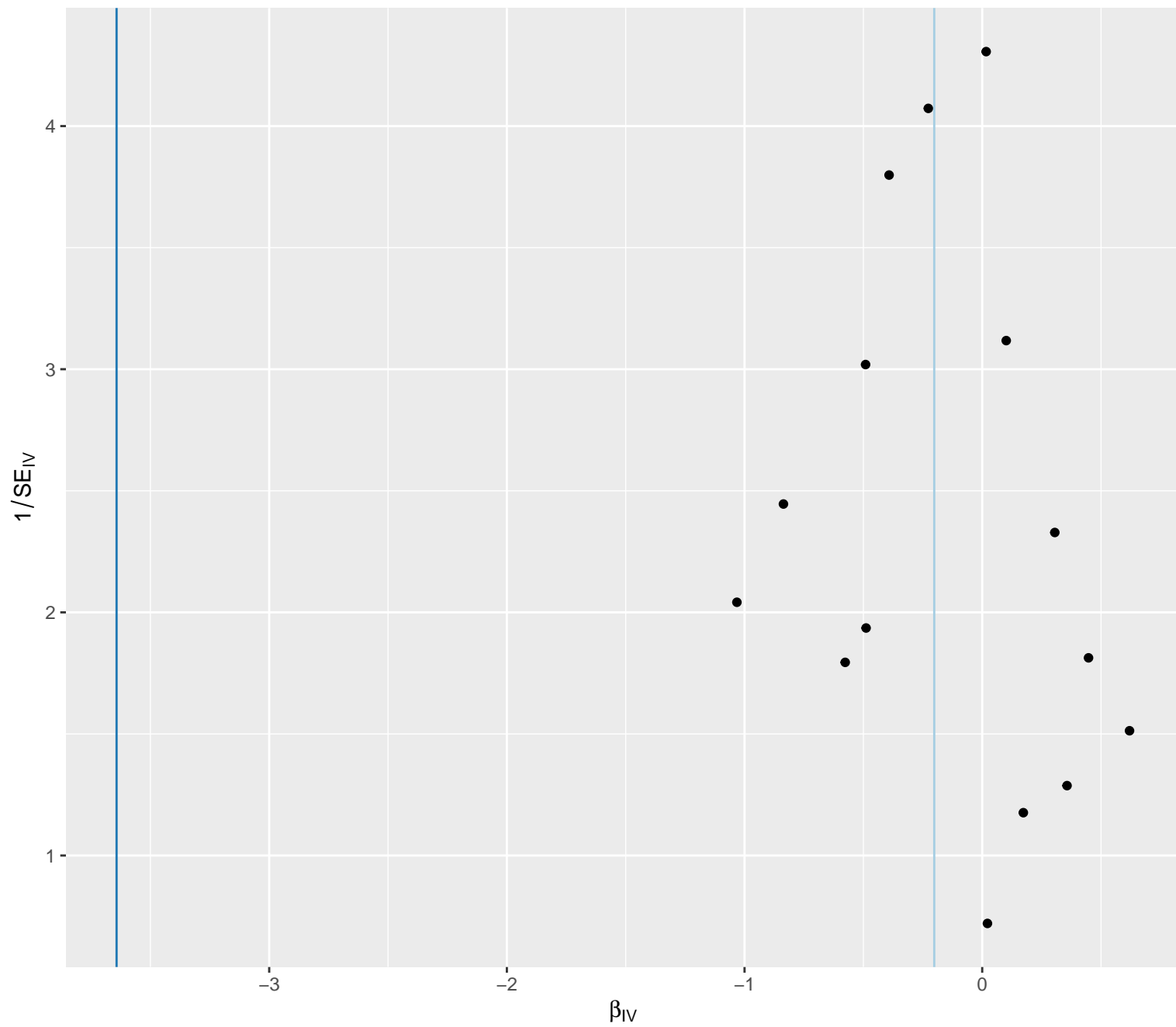

Supplement: Supplementary file 1 [file Data_Sheet_1.zip › Supplementary Materials/MR plots for tongue/Chronic sinusitis/s__Streptococcus_mitis_AT_mgs_2307/funnel.pdf]

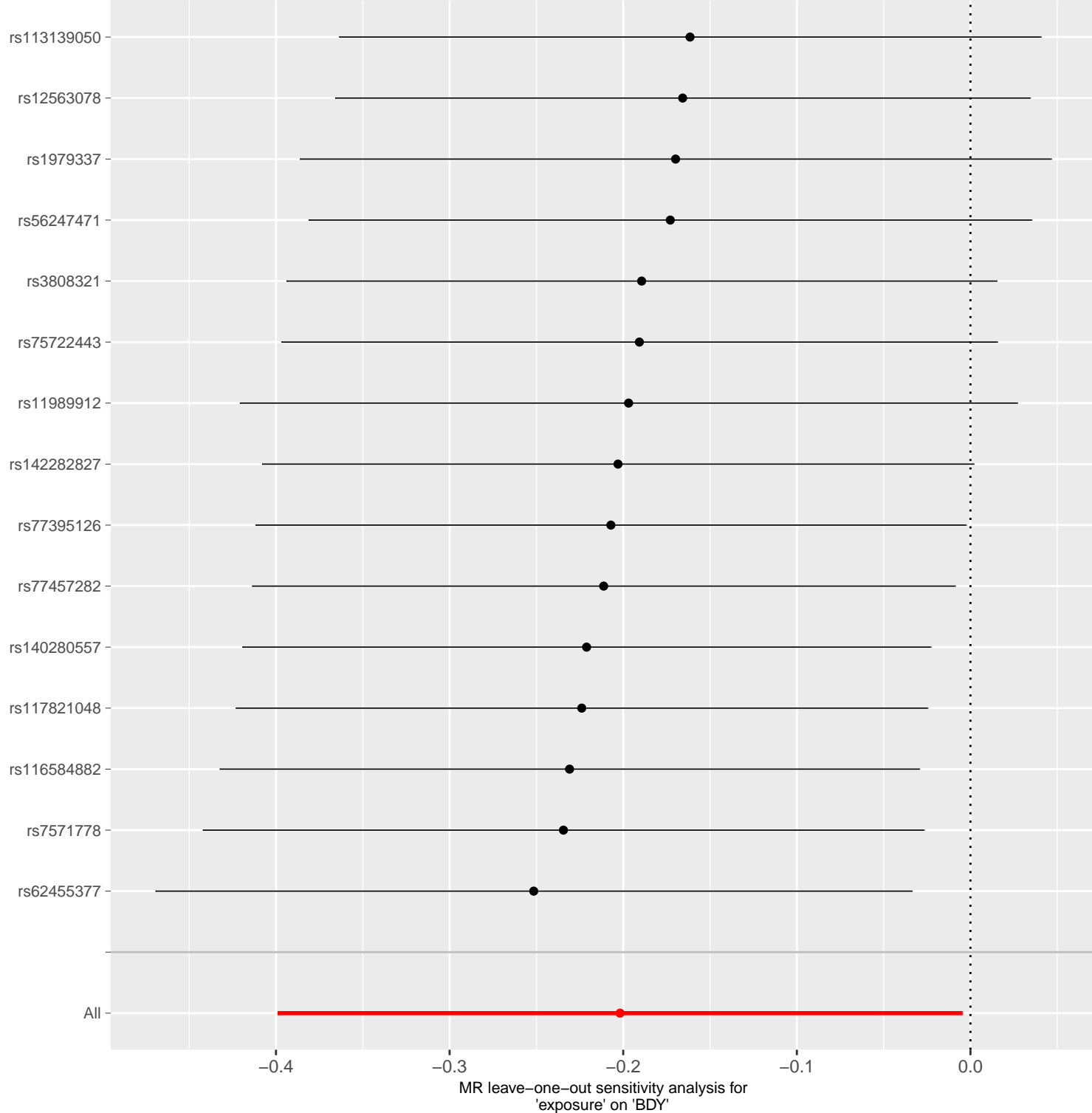

Supplement: Supplementary file 1 [file Data_Sheet_1.zip › Supplementary Materials/MR plots for tongue/Chronic sinusitis/s__Streptococcus_mitis_AT_mgs_2307/leave_one_out.pdf]

# MR Test

- Inverse variance weighted
- MR Egger
- Weighted median

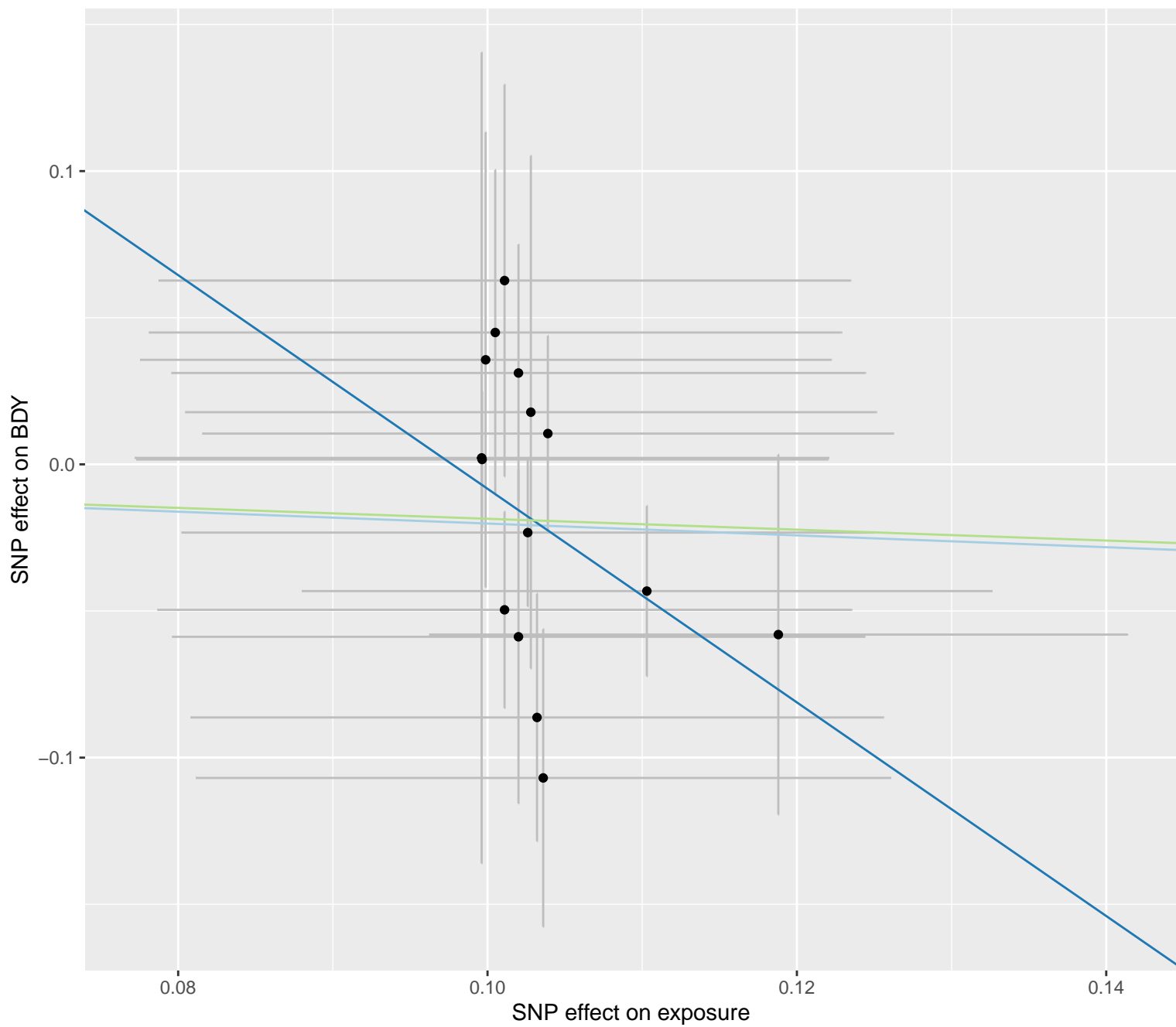

Supplement: Supplementary file 1 [file Data_Sheet_1.zip › Supplementary Materials/MR plots for tongue/Chronic sinusitis/s__Streptococcus_mitis_AT_mgs_2307/scatter.pdf]

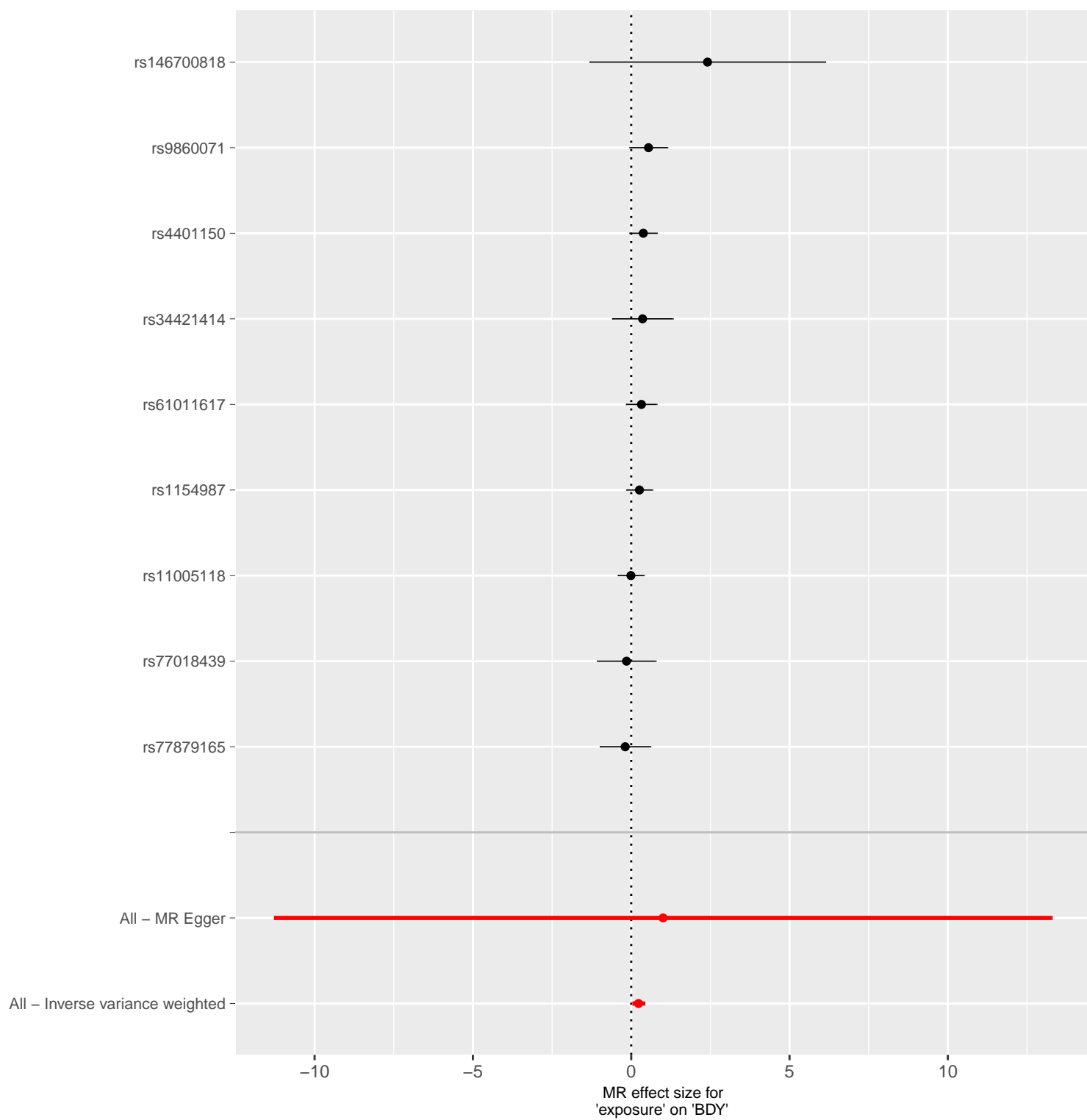

Supplement: Supplementary file 1 [file Data_Sheet_1.zip › Supplementary Materials/MR plots for tongue/Chronic sinusitis/s__Streptococcus_mitis_I_mgs_2661/forest.pdf]

# MR Method

- Inverse variance weighted
- MR Egger

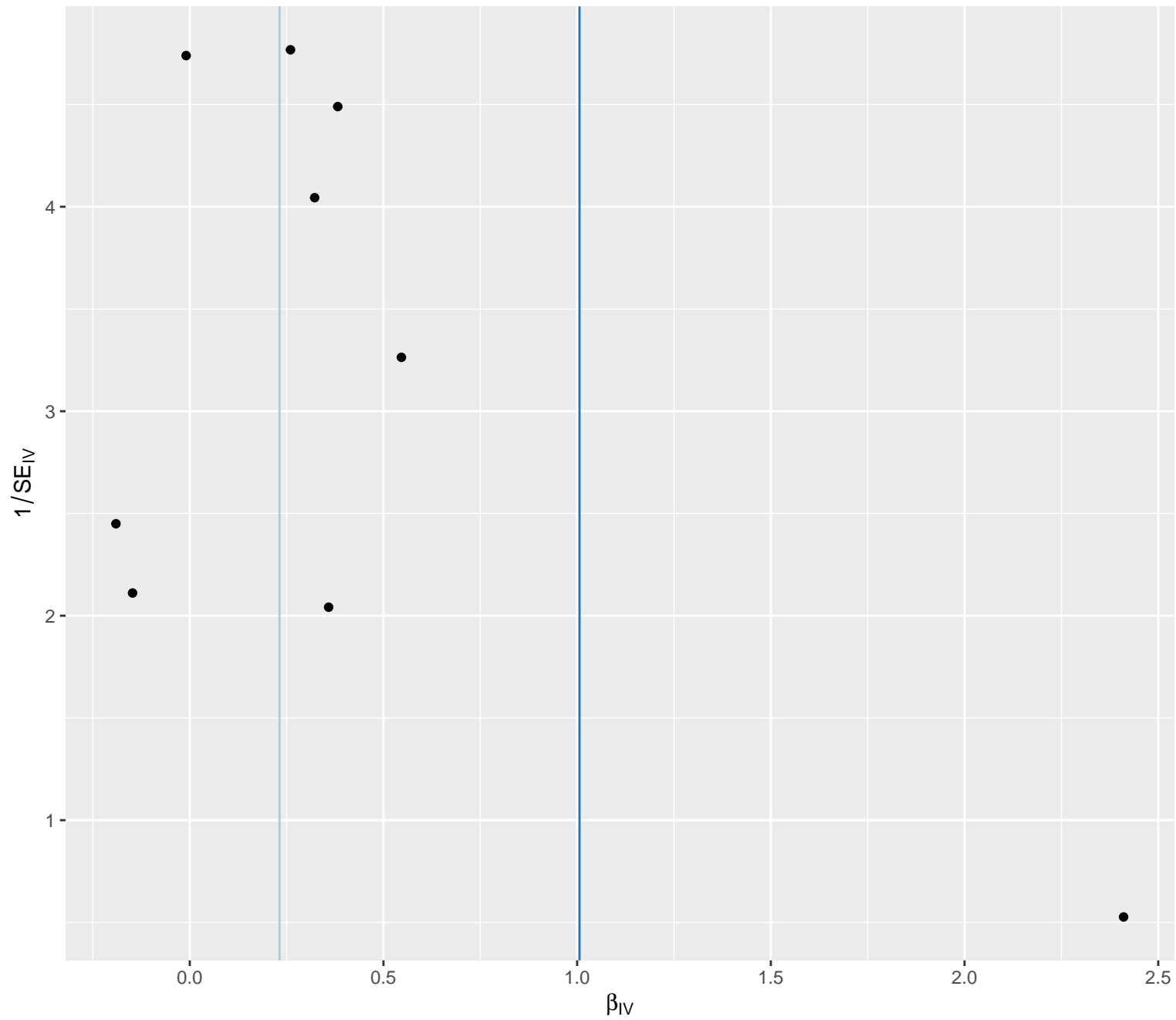

Supplement: Supplementary file 1 [file Data_Sheet_1.zip › Supplementary Materials/MR plots for tongue/Chronic sinusitis/s__Streptococcus_mitis_I_mgs_2661/funnel.pdf]

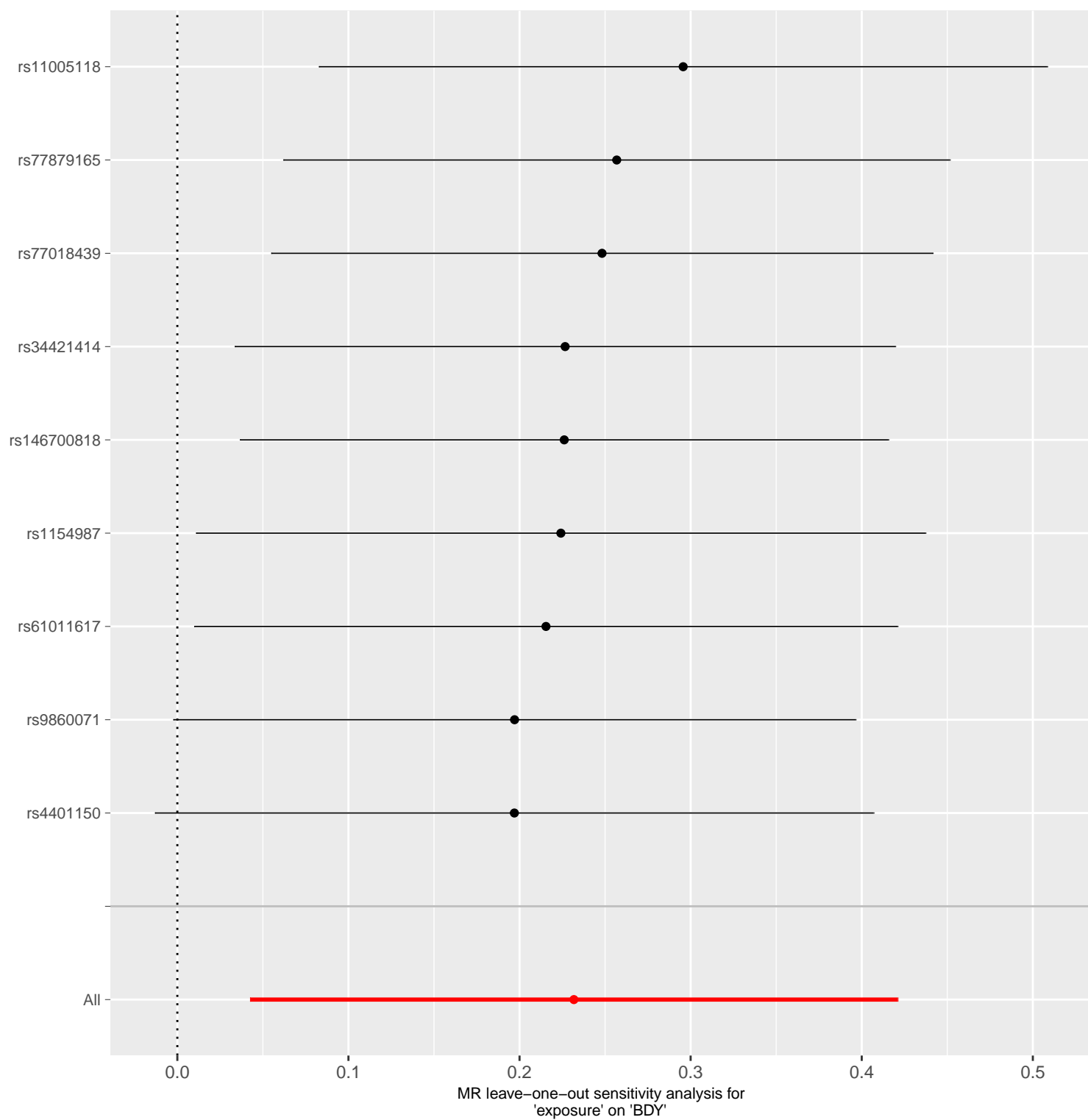

Supplement: Supplementary file 1 [file Data_Sheet_1.zip › Supplementary Materials/MR plots for tongue/Chronic sinusitis/s__Streptococcus_mitis_I_mgs_2661/leave_one_out.pdf]

# MR Test

- Inverse variance weighted
- MR Egger
- Weighted median

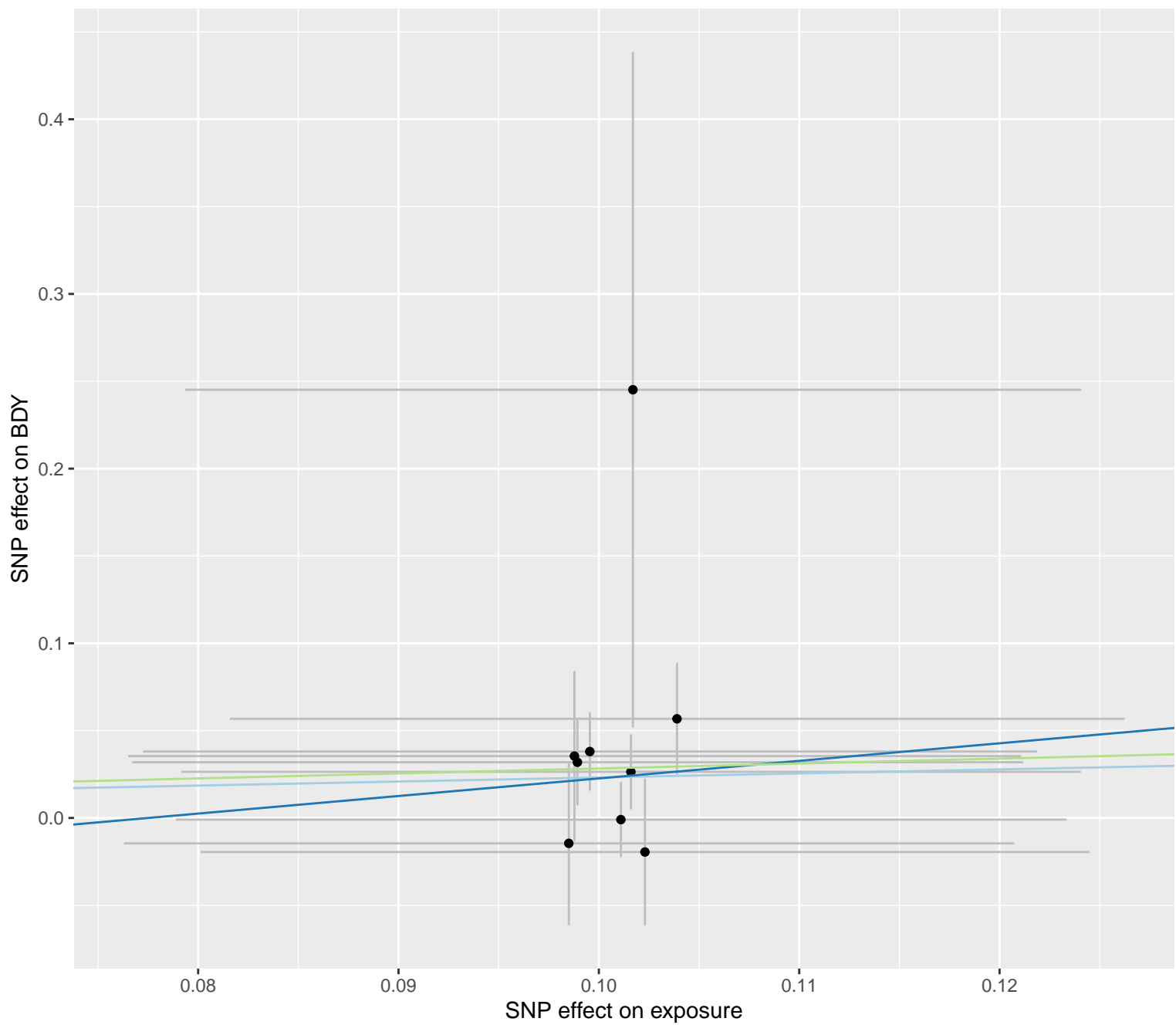

Supplement: Supplementary file 1 [file Data_Sheet_1.zip › Supplementary Materials/MR plots for tongue/Chronic sinusitis/s__Streptococcus_mitis_I_mgs_2661/scatter.pdf]

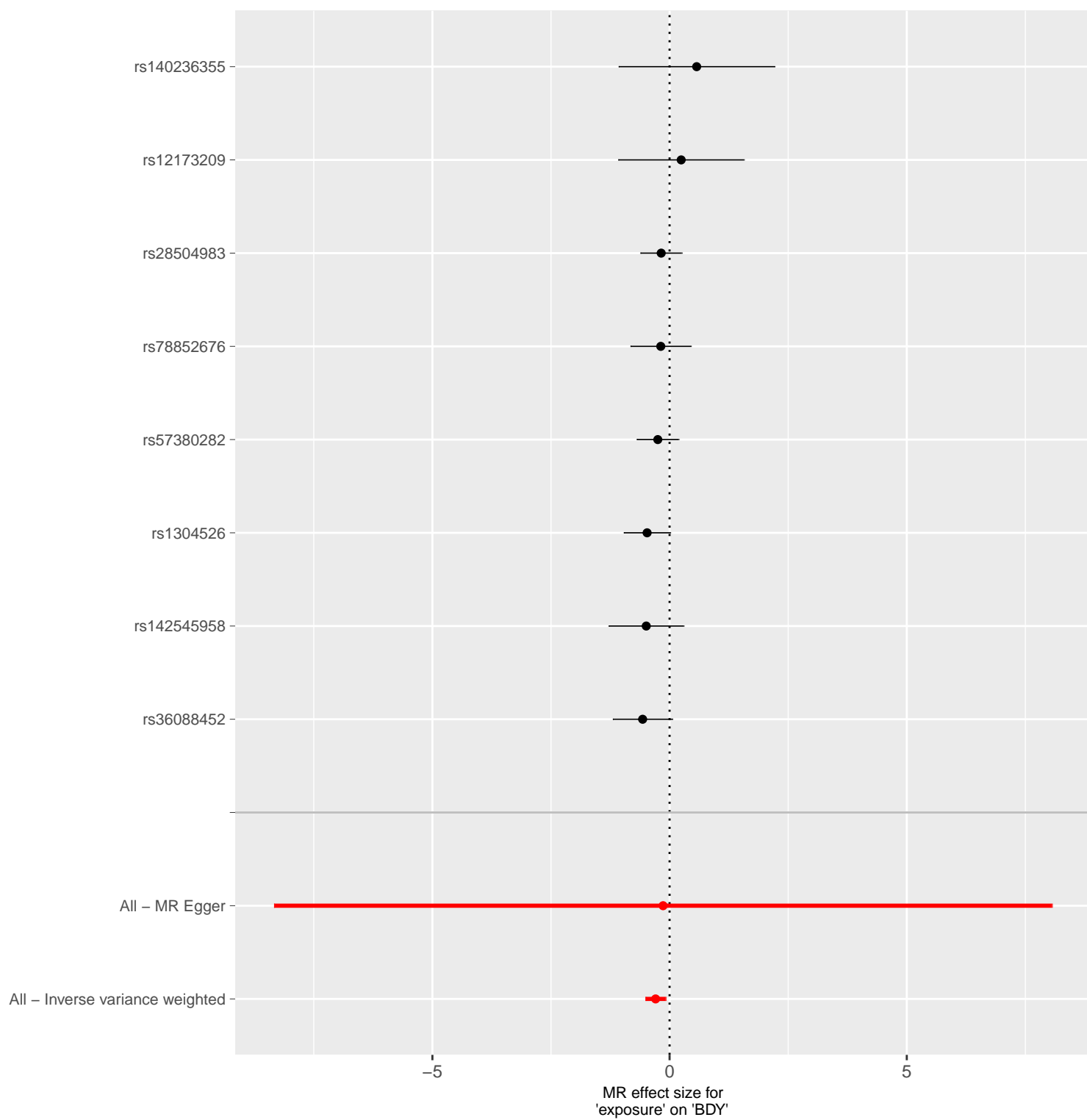

Supplement: Supplementary file 1 [file Data_Sheet_1.zip › Supplementary Materials/MR plots for tongue/Chronic sinusitis/s__Streptococcus_sp000187745_mgs_3504/forest.pdf]

# MR Method

- Inverse variance weighted
- MR Egger

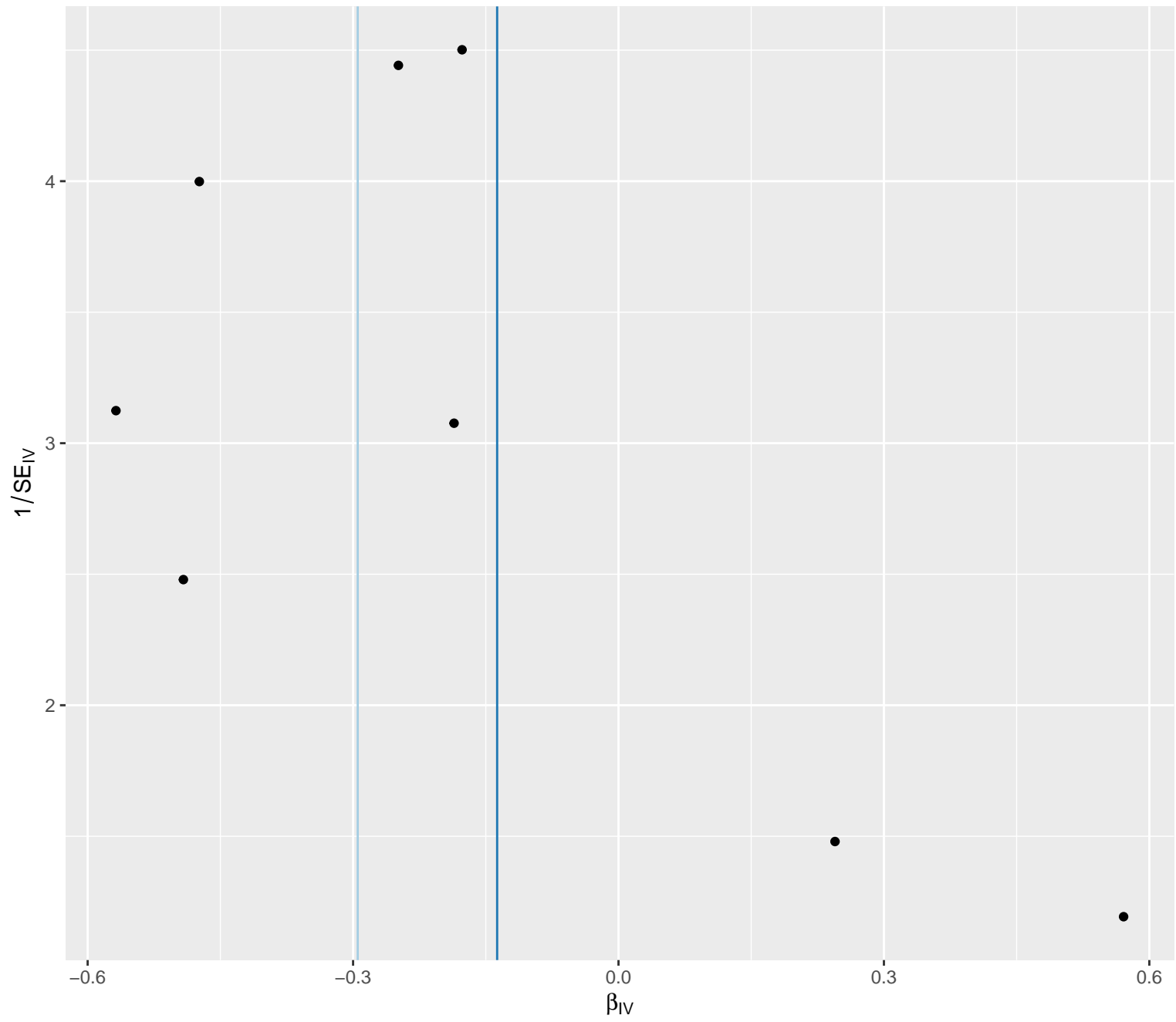

Supplement: Supplementary file 1 [file Data_Sheet_1.zip › Supplementary Materials/MR plots for tongue/Chronic sinusitis/s__Streptococcus_sp000187745_mgs_3504/funnel.pdf]

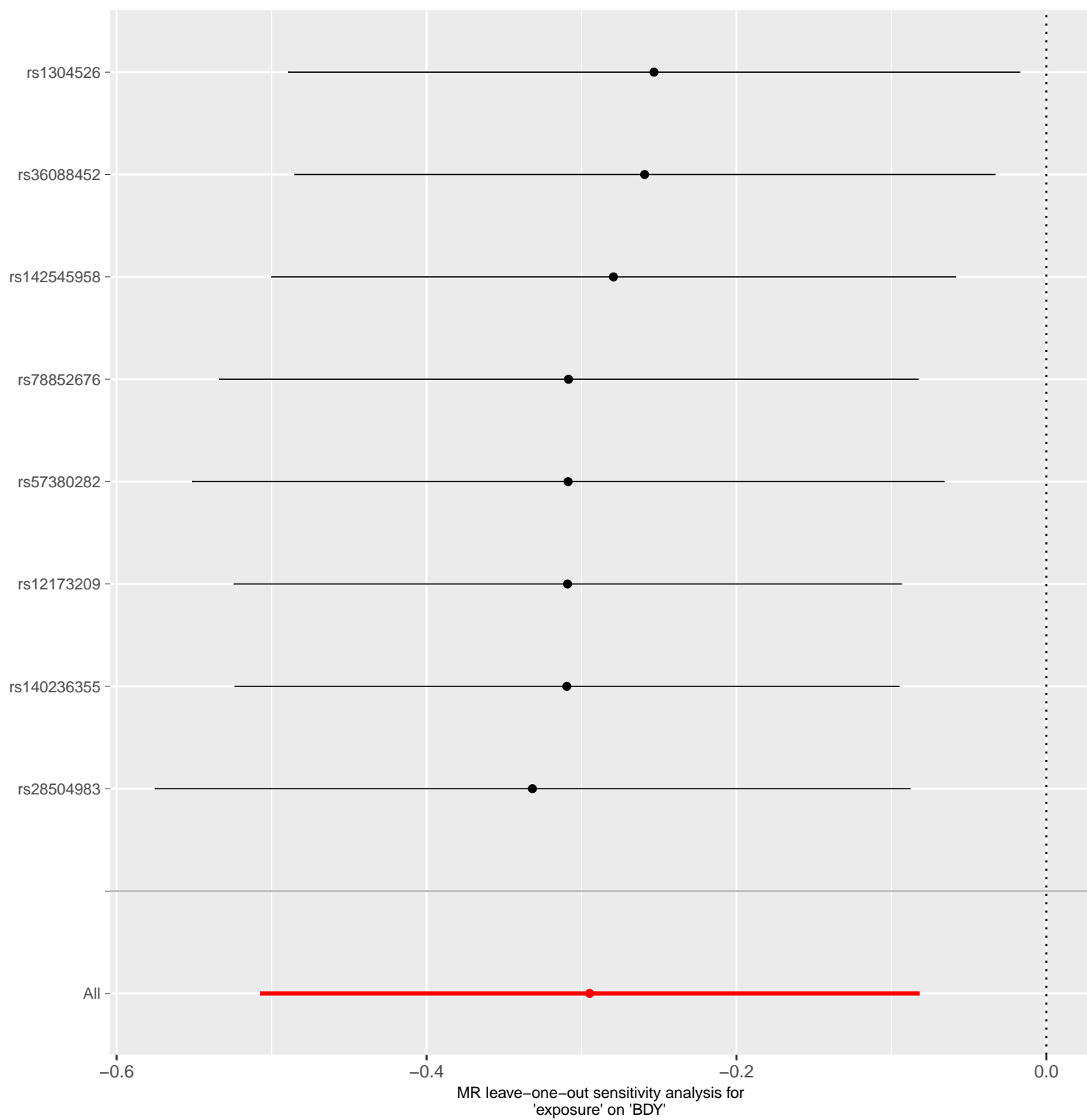

Supplement: Supplementary file 1 [file Data_Sheet_1.zip › Supplementary Materials/MR plots for tongue/Chronic sinusitis/s__Streptococcus_sp000187745_mgs_3504/leave_one_out.pdf]

# MR Test

- Inverse variance weighted
- MR Egger
- Weighted median

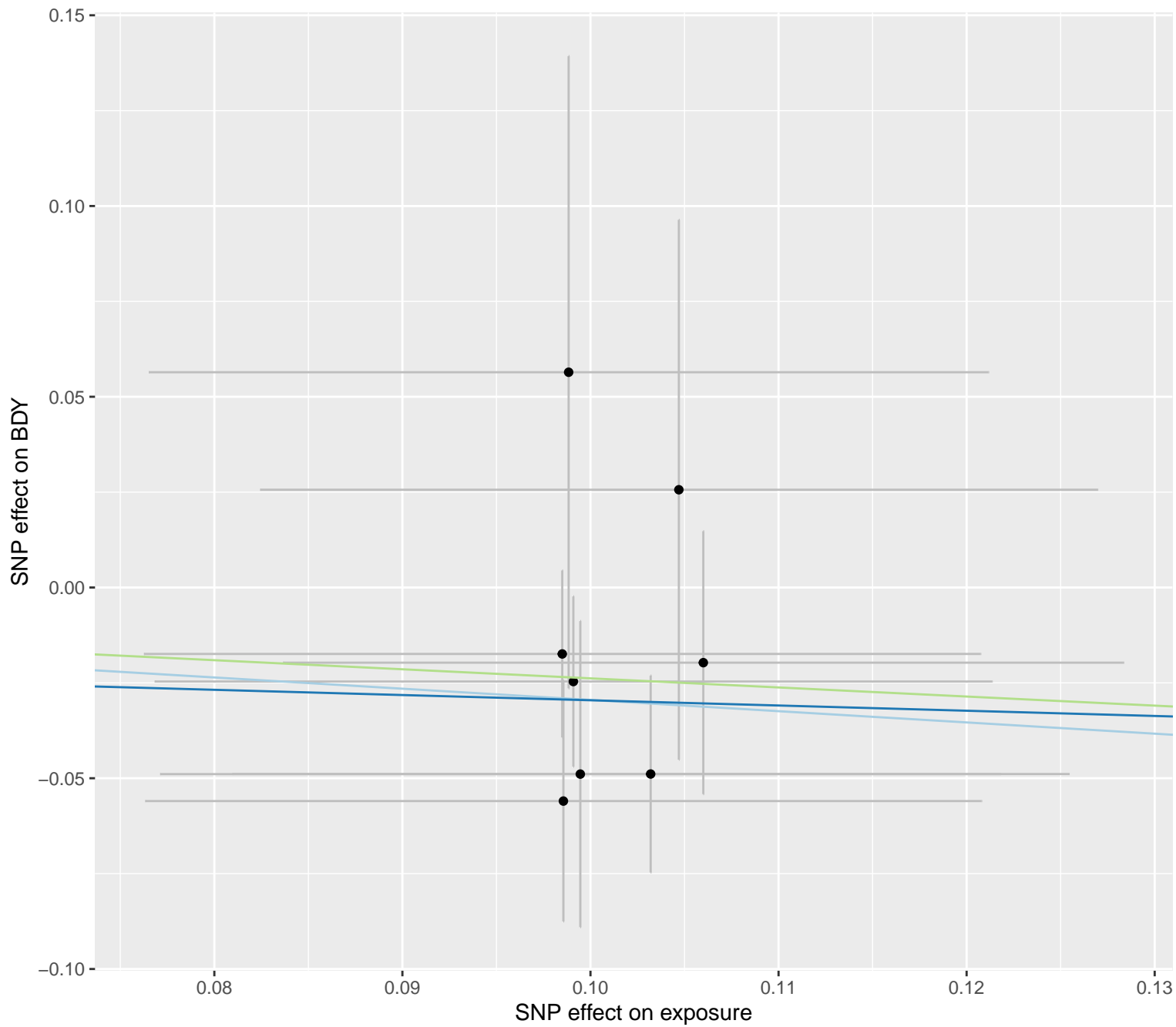

Supplement: Supplementary file 1 [file Data_Sheet_1.zip › Supplementary Materials/MR plots for tongue/Chronic sinusitis/s__Streptococcus_sp000187745_mgs_3504/scatter.pdf]

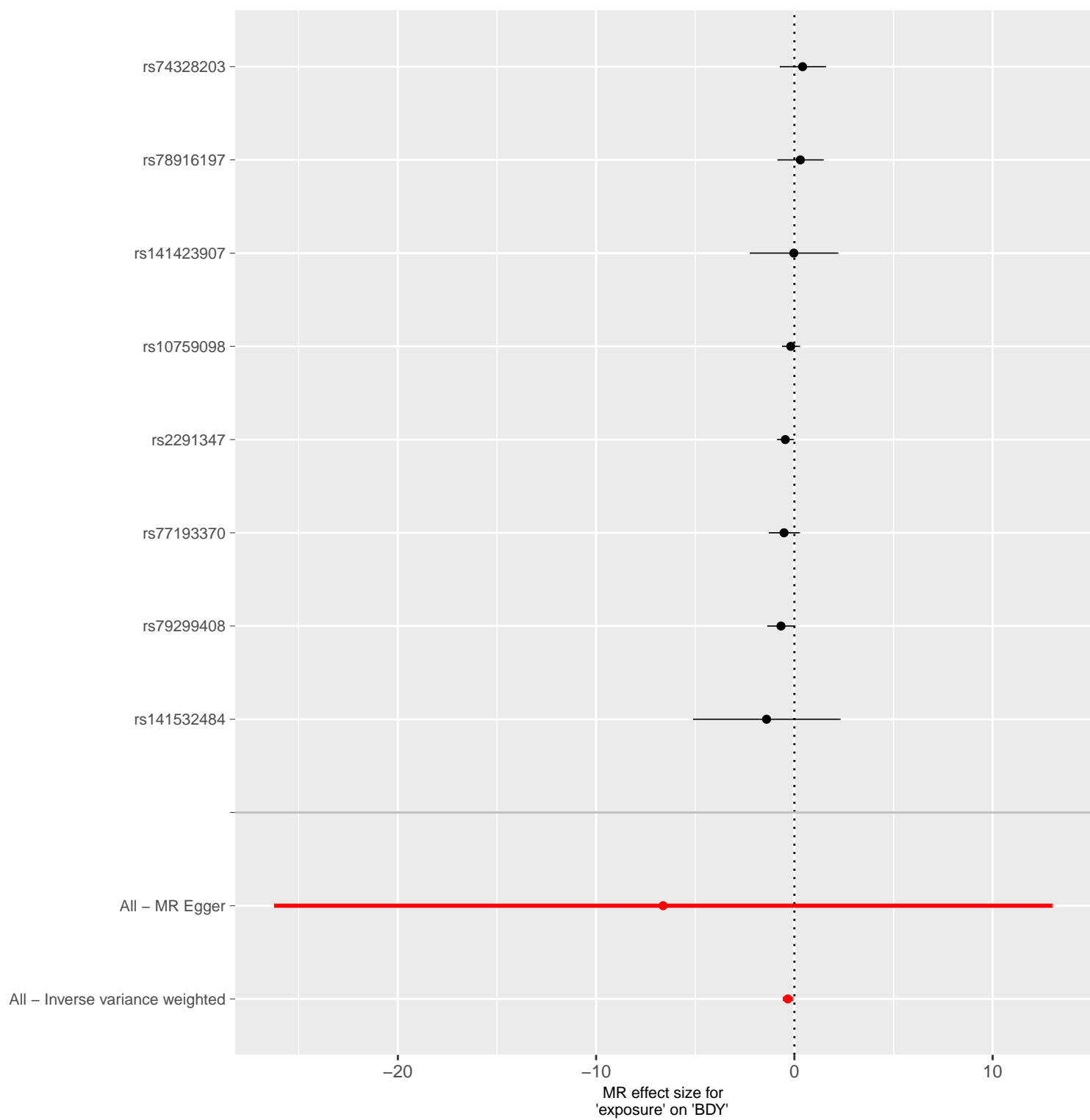

Supplement: Supplementary file 1 [file Data_Sheet_1.zip › Supplementary Materials/MR plots for tongue/Chronic sinusitis/s__Tannerella_forsythia_mgs_3235/forest.pdf]

# MR Method

- Inverse variance weighted
- MR Egger

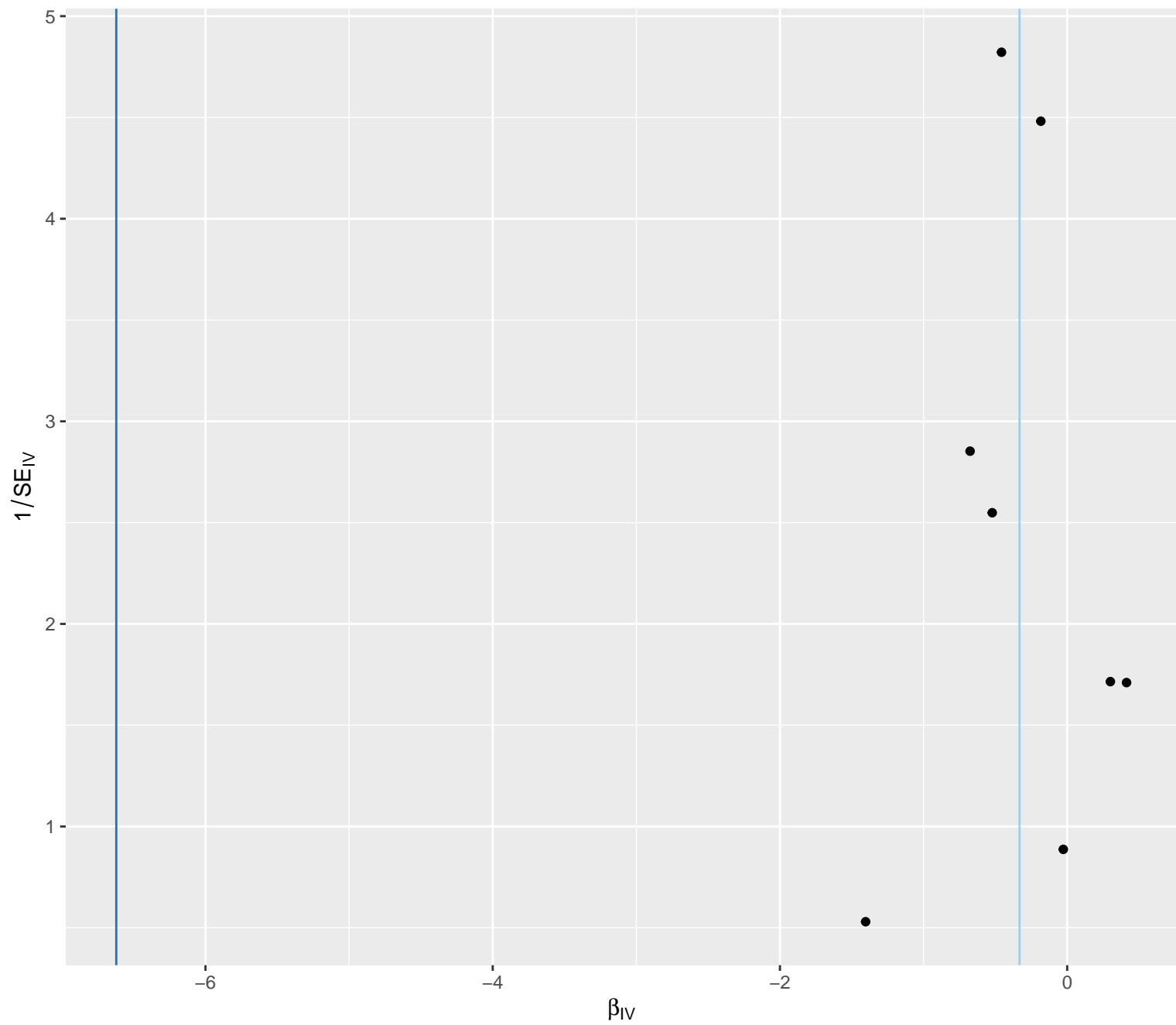

Supplement: Supplementary file 1 [file Data_Sheet_1.zip › Supplementary Materials/MR plots for tongue/Chronic sinusitis/s__Tannerella_forsythia_mgs_3235/funnel.pdf]

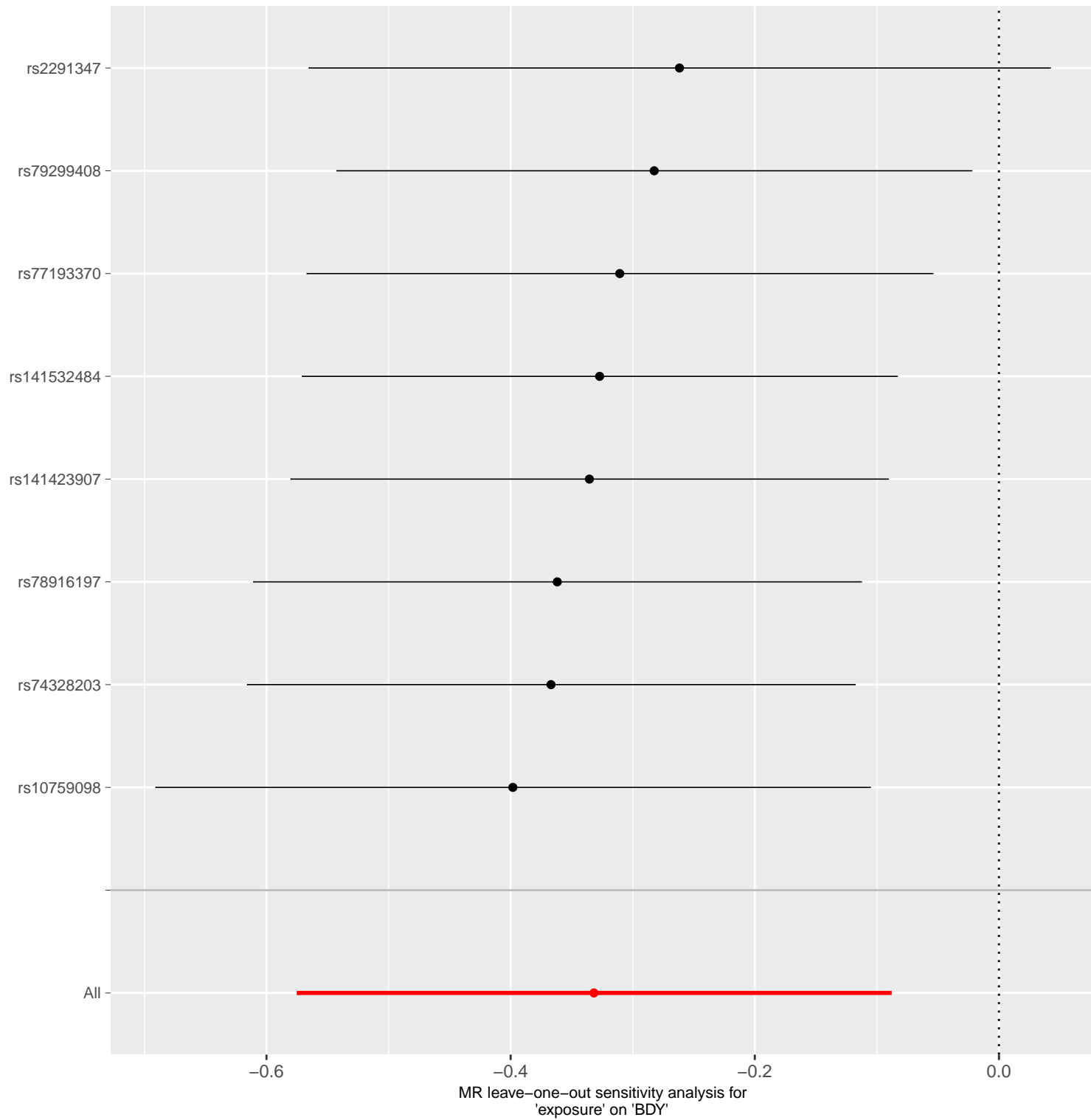

Supplement: Supplementary file 1 [file Data_Sheet_1.zip › Supplementary Materials/MR plots for tongue/Chronic sinusitis/s__Tannerella_forsythia_mgs_3235/leave_one_out.pdf]

# MR Test

- Inverse variance weighted
- MR Egger
- Weighted median

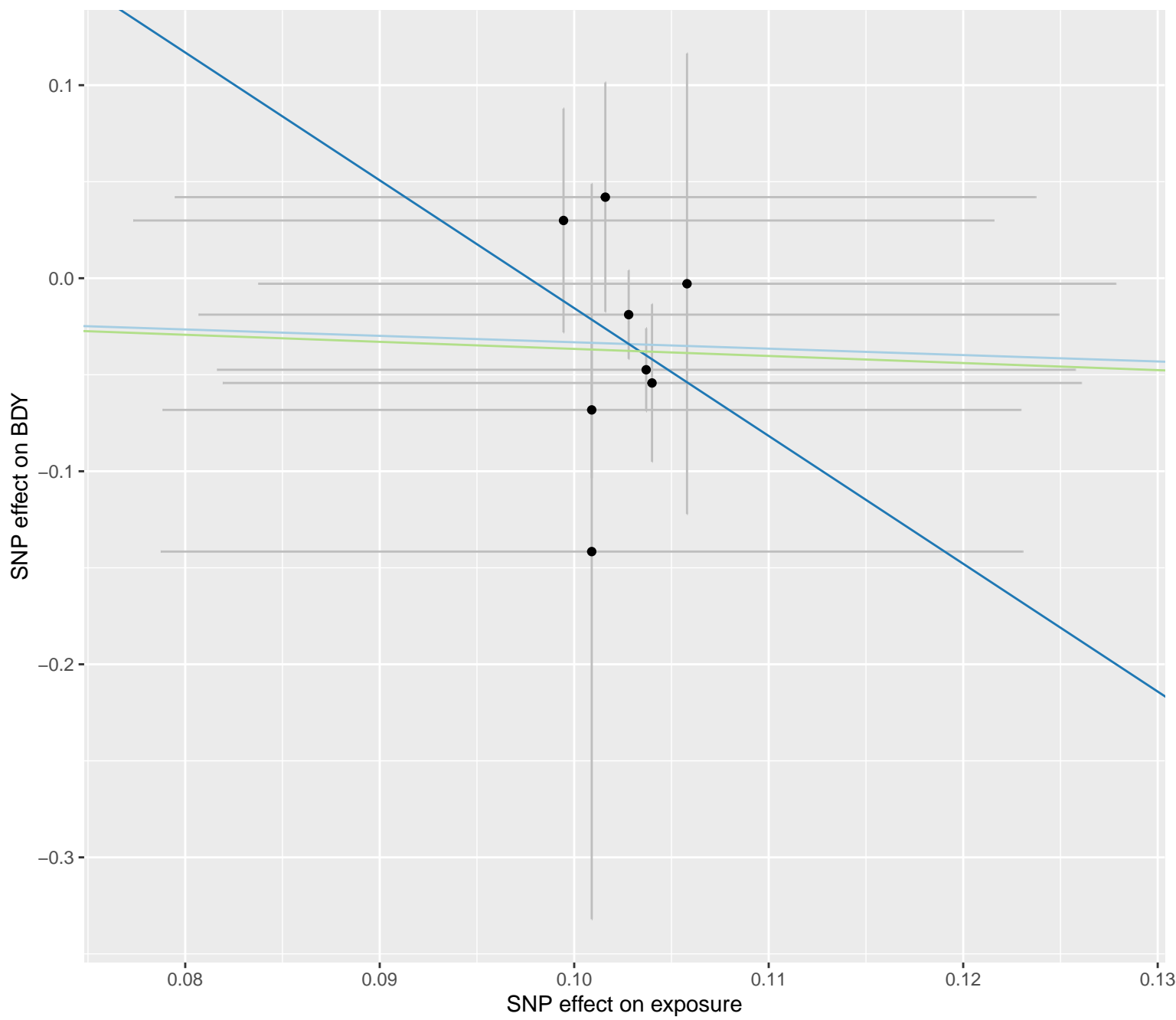

Supplement: Supplementary file 1 [file Data_Sheet_1.zip › Supplementary Materials/MR plots for tongue/Chronic sinusitis/s__Tannerella_forsythia_mgs_3235/scatter.pdf]

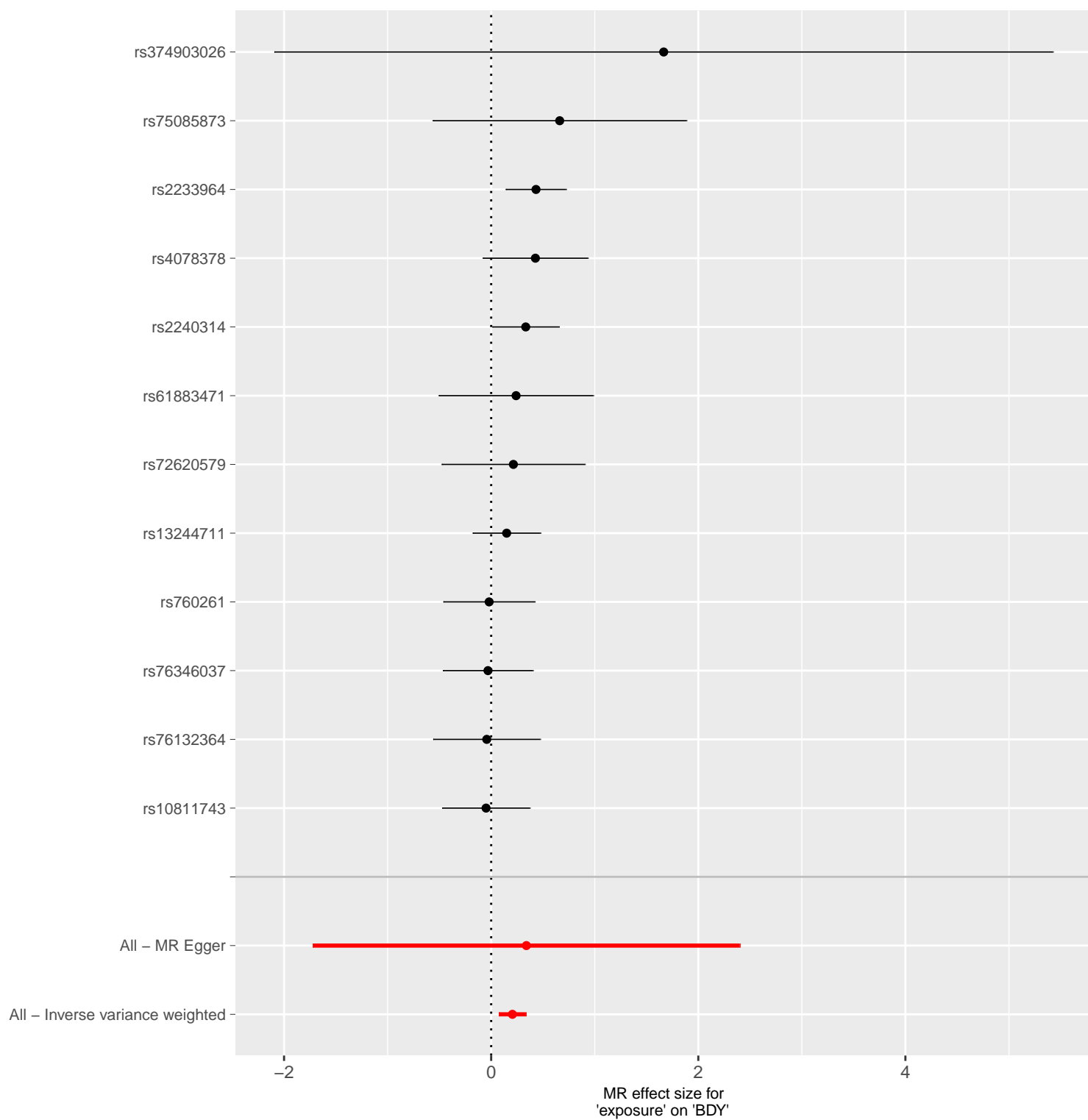

Supplement: Supplementary file 1 [file Data_Sheet_1.zip › Supplementary Materials/MR plots for tongue/Pneumonia/g__unclassified_mgs_1254/forest.pdf]

# MR Method

- Inverse variance weighted
- MR Egger

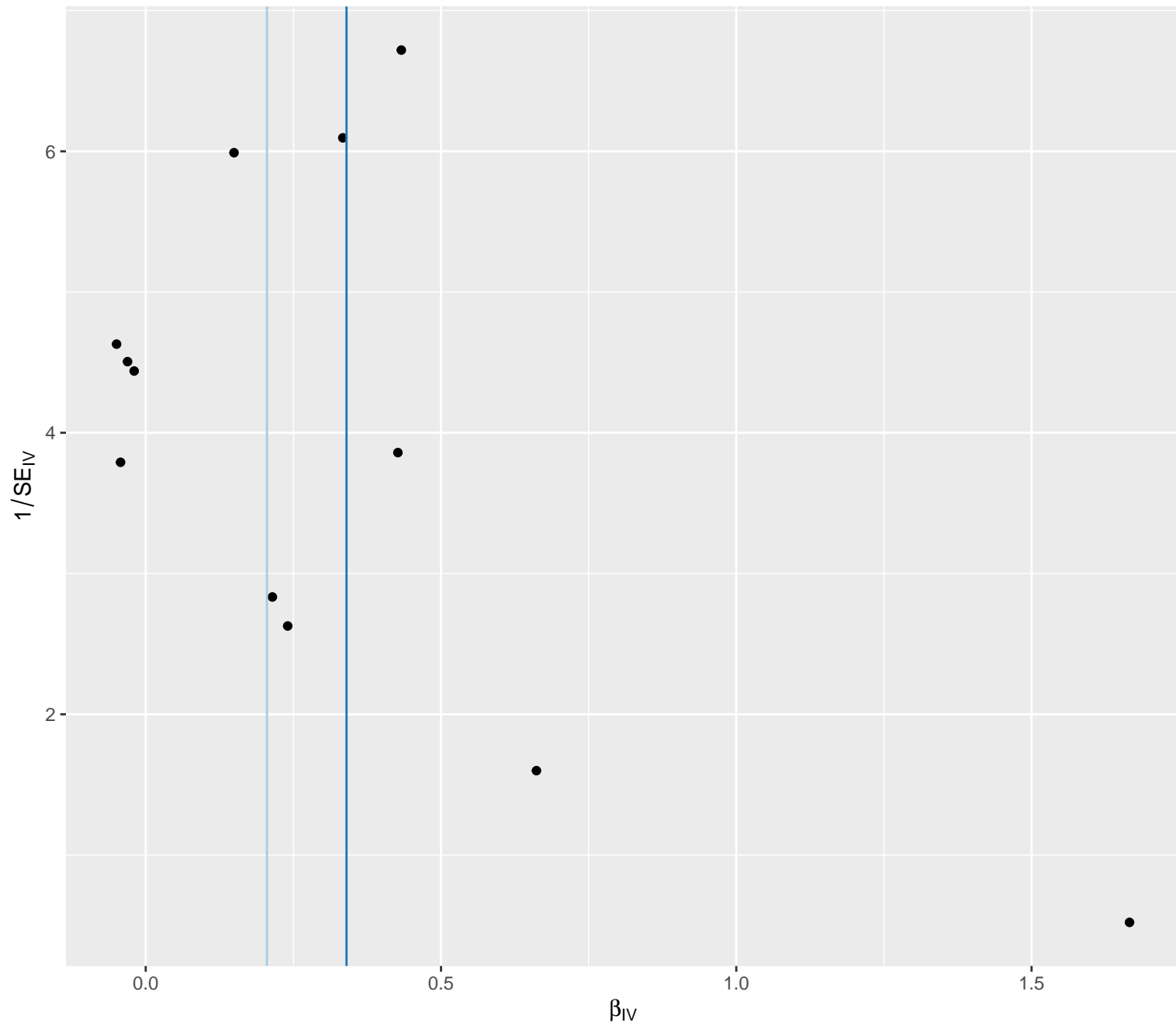

Supplement: Supplementary file 1 [file Data_Sheet_1.zip › Supplementary Materials/MR plots for tongue/Pneumonia/g__unclassified_mgs_1254/funnel.pdf]

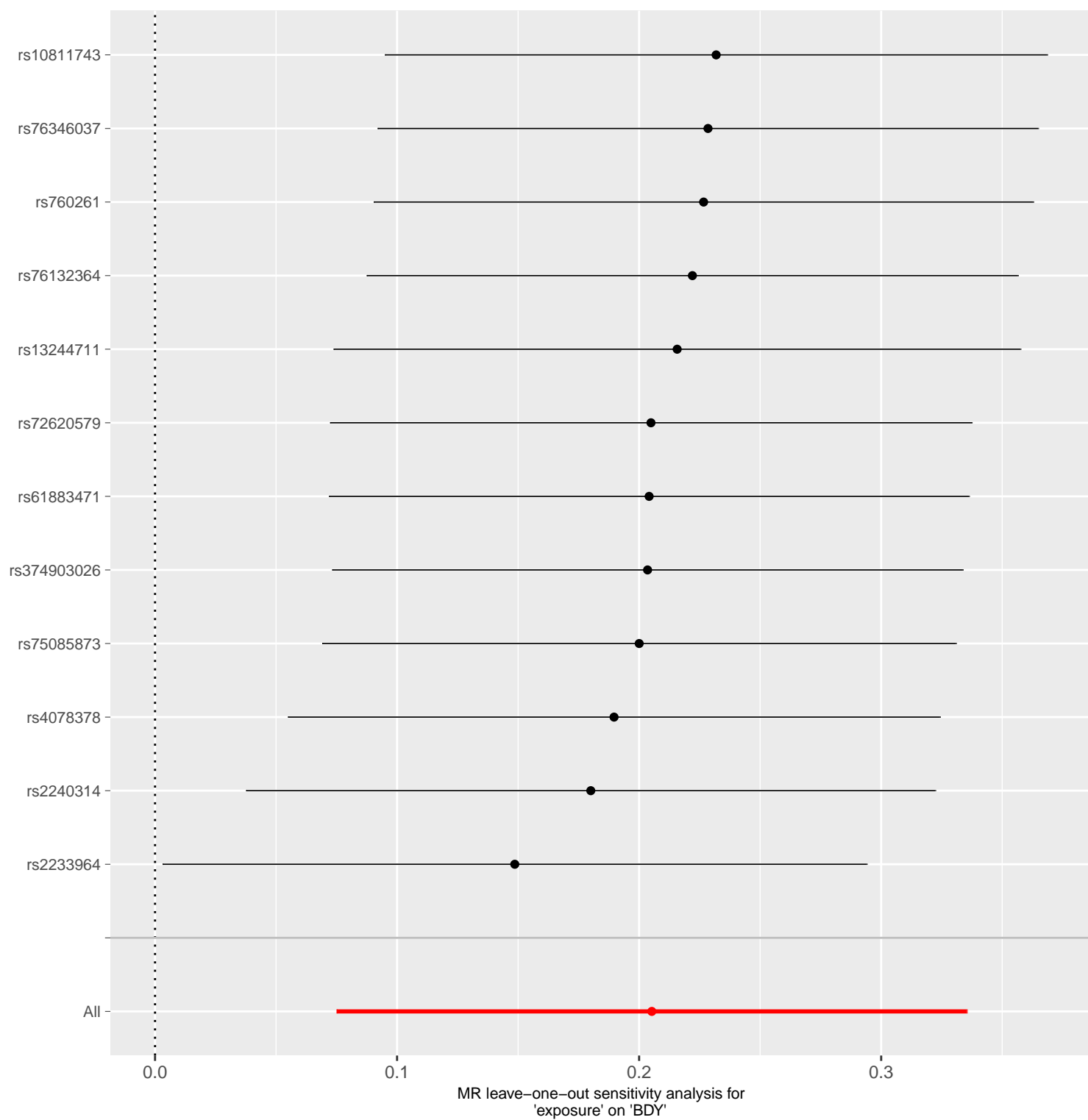

Supplement: Supplementary file 1 [file Data_Sheet_1.zip › Supplementary Materials/MR plots for tongue/Pneumonia/g__unclassified_mgs_1254/leave_one_out.pdf]

# MR Test

- Inverse variance weighted
- MR Egger
- Weighted median

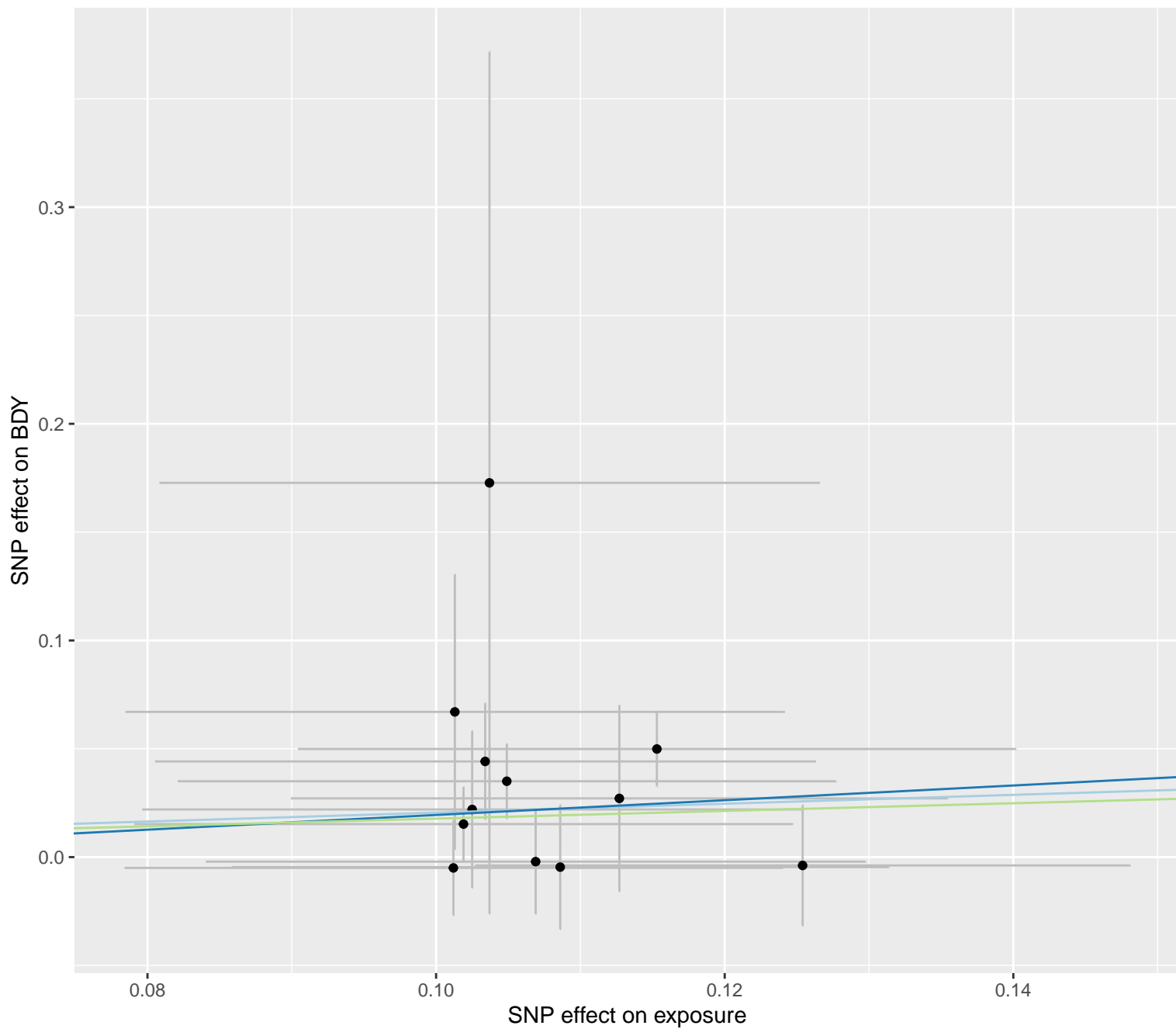

Supplement: Supplementary file 1 [file Data_Sheet_1.zip › Supplementary Materials/MR plots for tongue/Pneumonia/g__unclassified_mgs_1254/scatter.pdf]

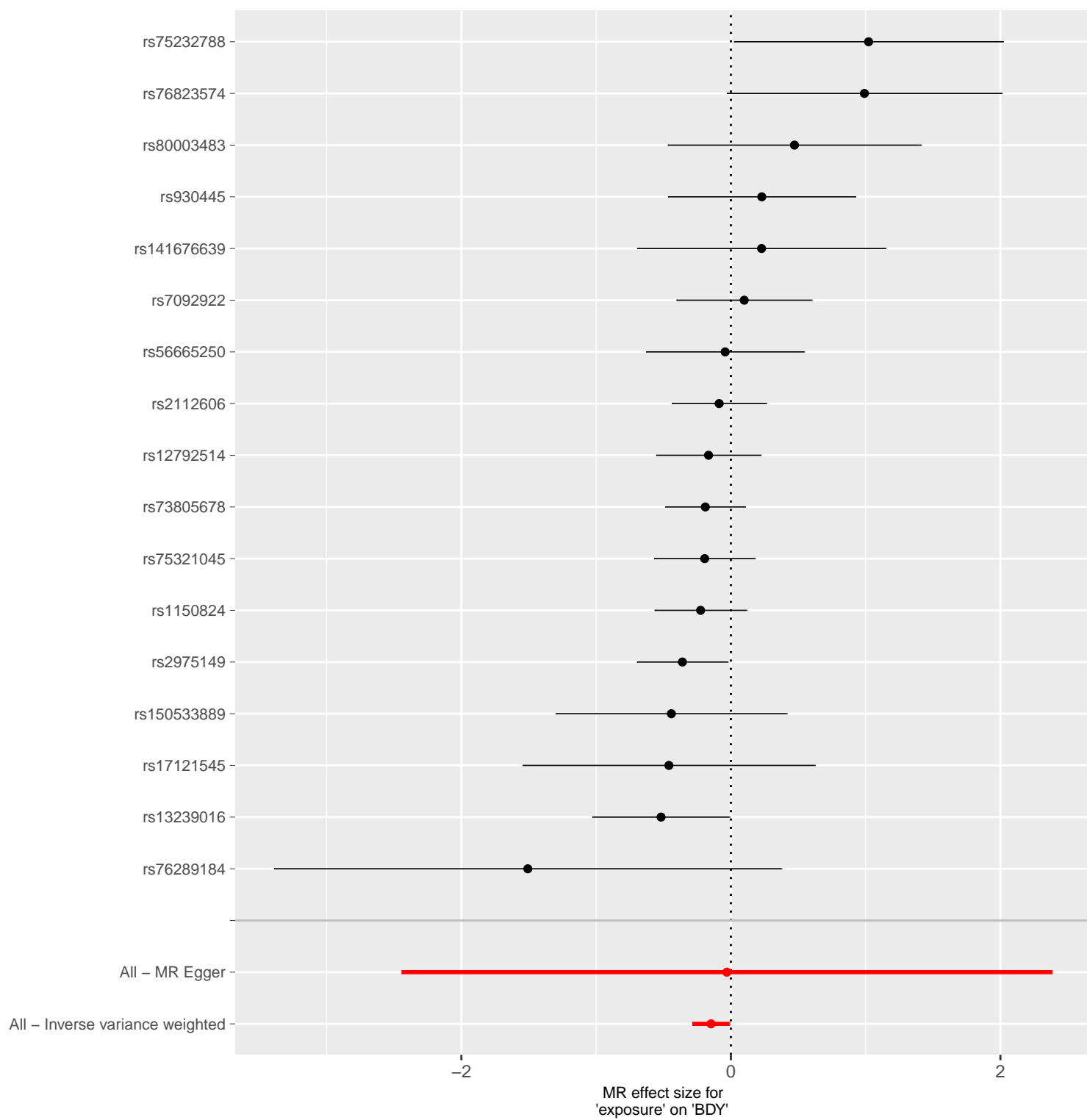

Supplement: Supplementary file 1 [file Data_Sheet_1.zip › Supplementary Materials/MR plots for tongue/Pneumonia/s__Actinomyces_oris_A_mgs_3282/forest.pdf]

# MR Method

Inverse variance weighted  
MR Egger

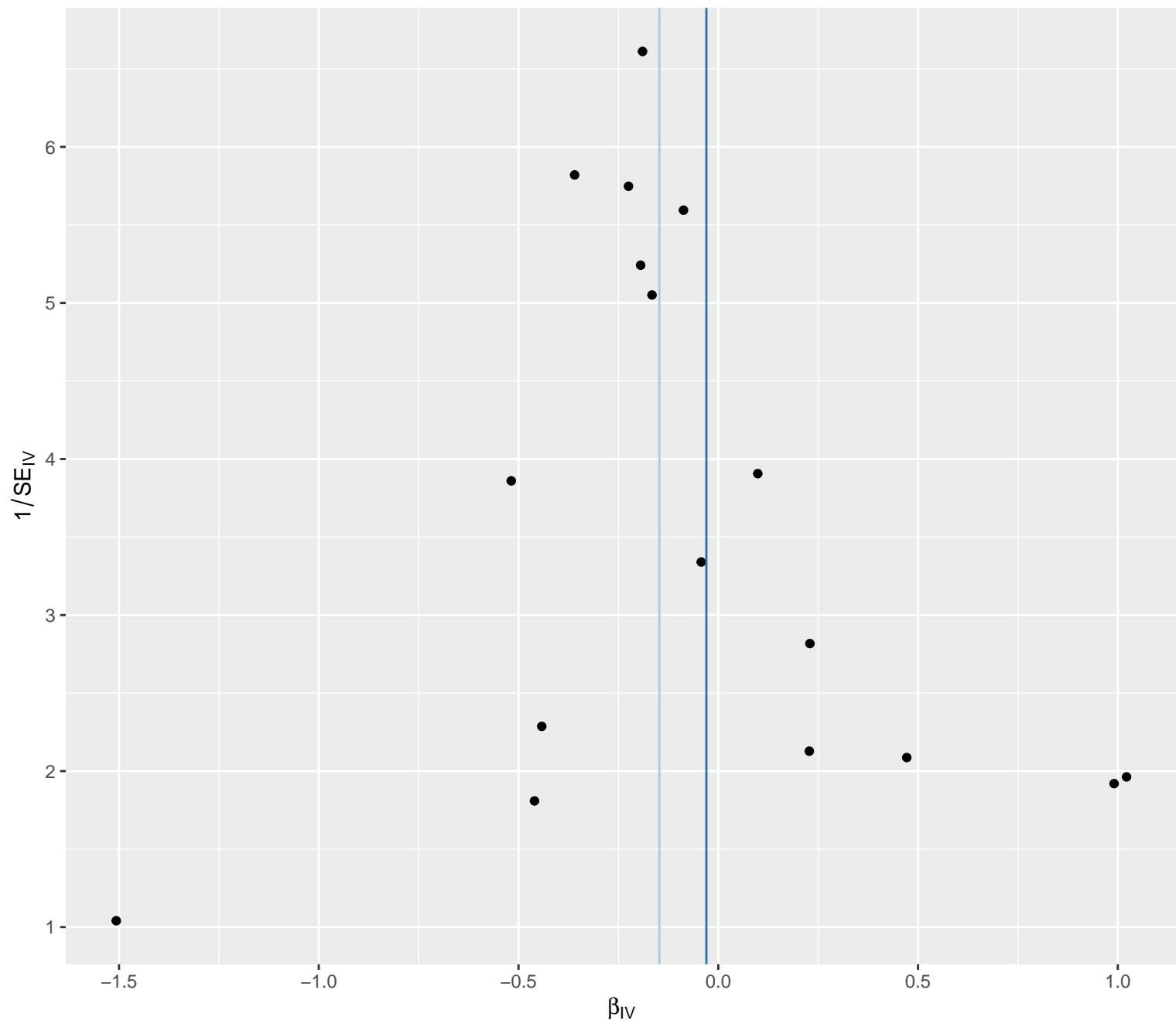

Supplement: Supplementary file 1 [file Data_Sheet_1.zip › Supplementary Materials/MR plots for tongue/Pneumonia/s__Actinomyces_oris_A_mgs_3282/funnel.pdf]

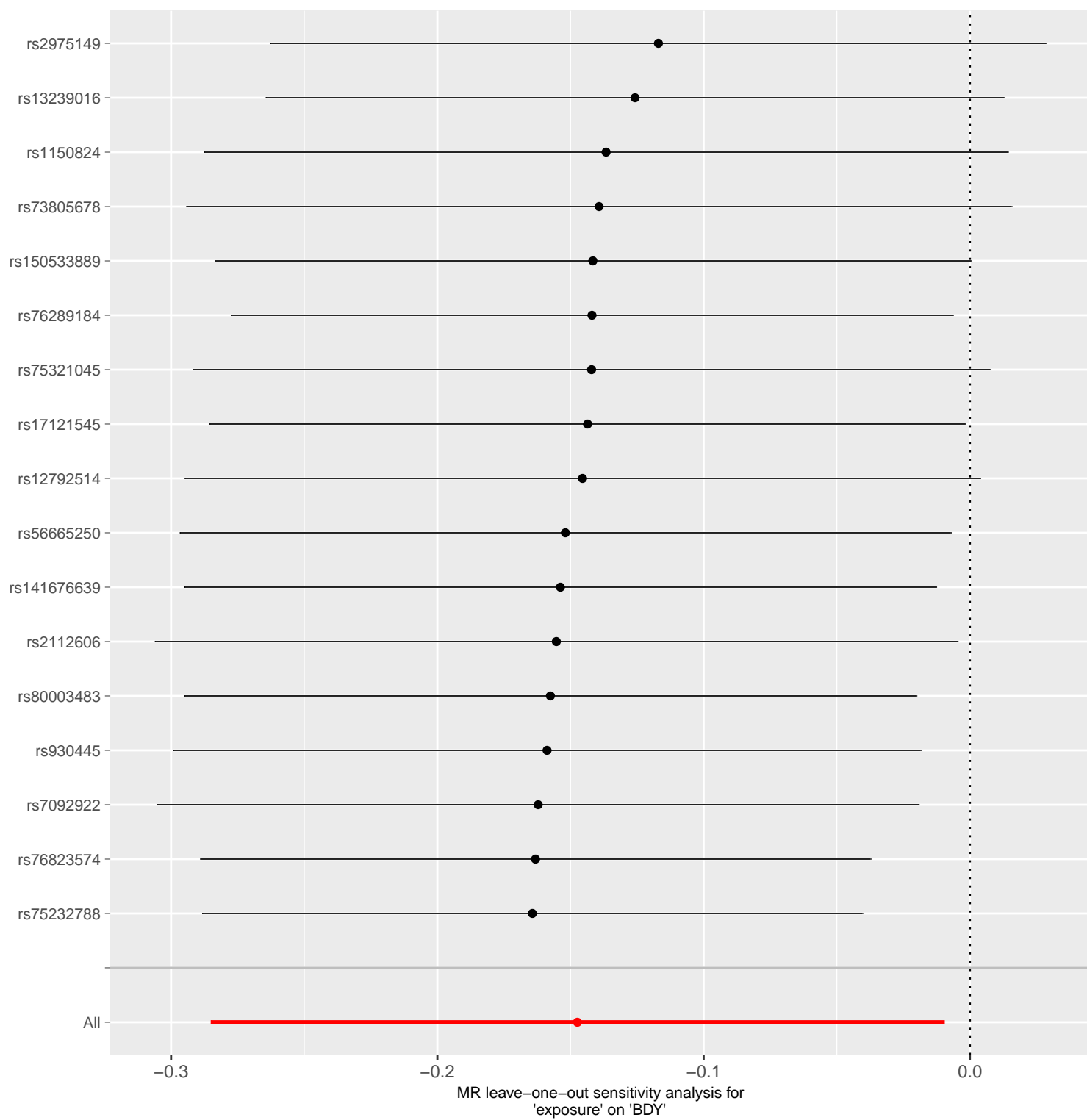

Supplement: Supplementary file 1 [file Data_Sheet_1.zip › Supplementary Materials/MR plots for tongue/Pneumonia/s__Actinomyces_oris_A_mgs_3282/leave_one_out.pdf]

# MR Test

- Inverse variance weighted
- MR Egger
- Weighted median

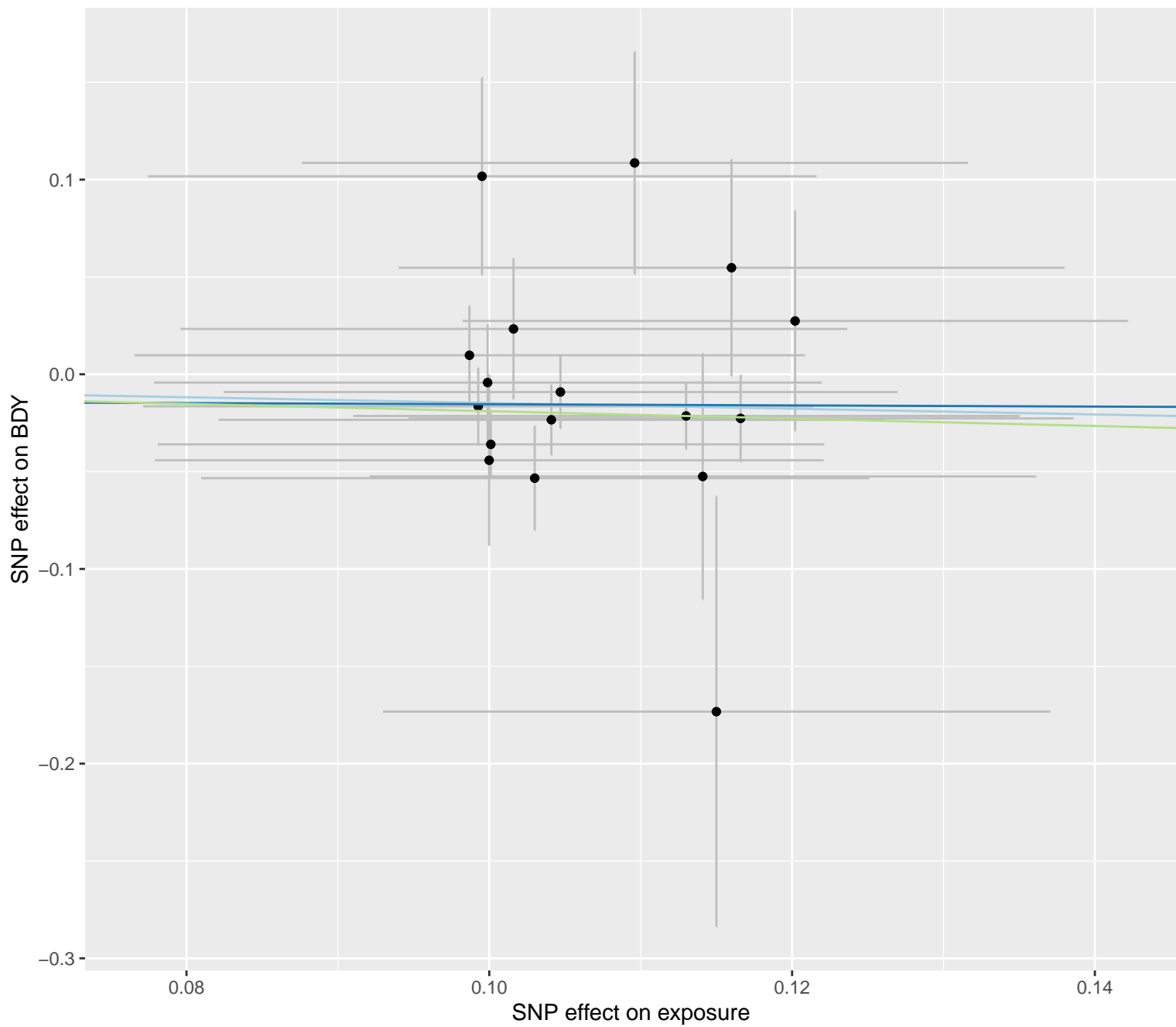

Supplement: Supplementary file 1 [file Data_Sheet_1.zip › Supplementary Materials/MR plots for tongue/Pneumonia/s__Actinomyces_oris_A_mgs_3282/scatter.pdf]

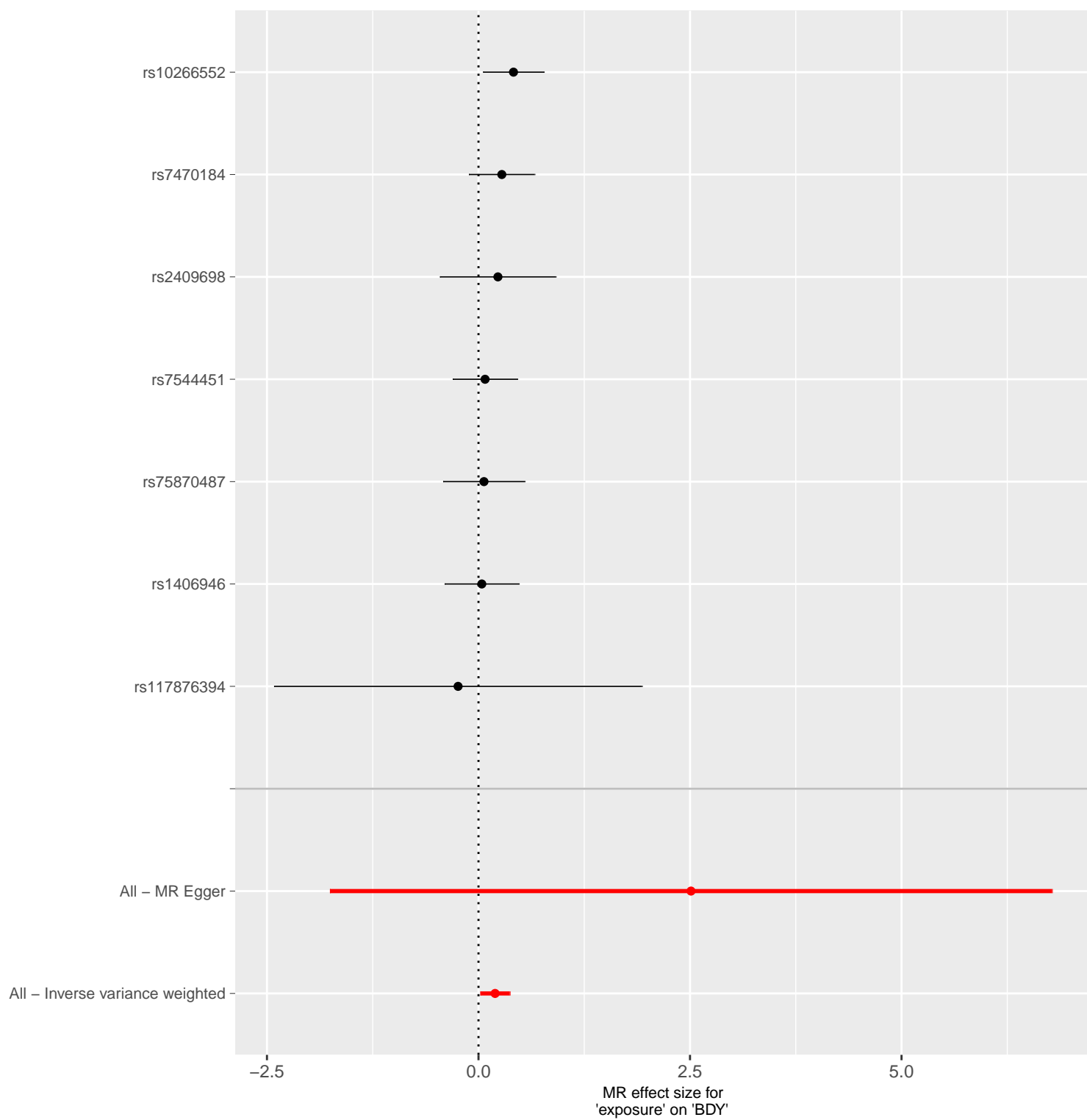

Supplement: Supplementary file 1 [file Data_Sheet_1.zip › Supplementary Materials/MR plots for tongue/Pneumonia/s__Alloprevotella_rava_mgs_541/forest.pdf]

# MR Method

- Inverse variance weighted
- MR Egger

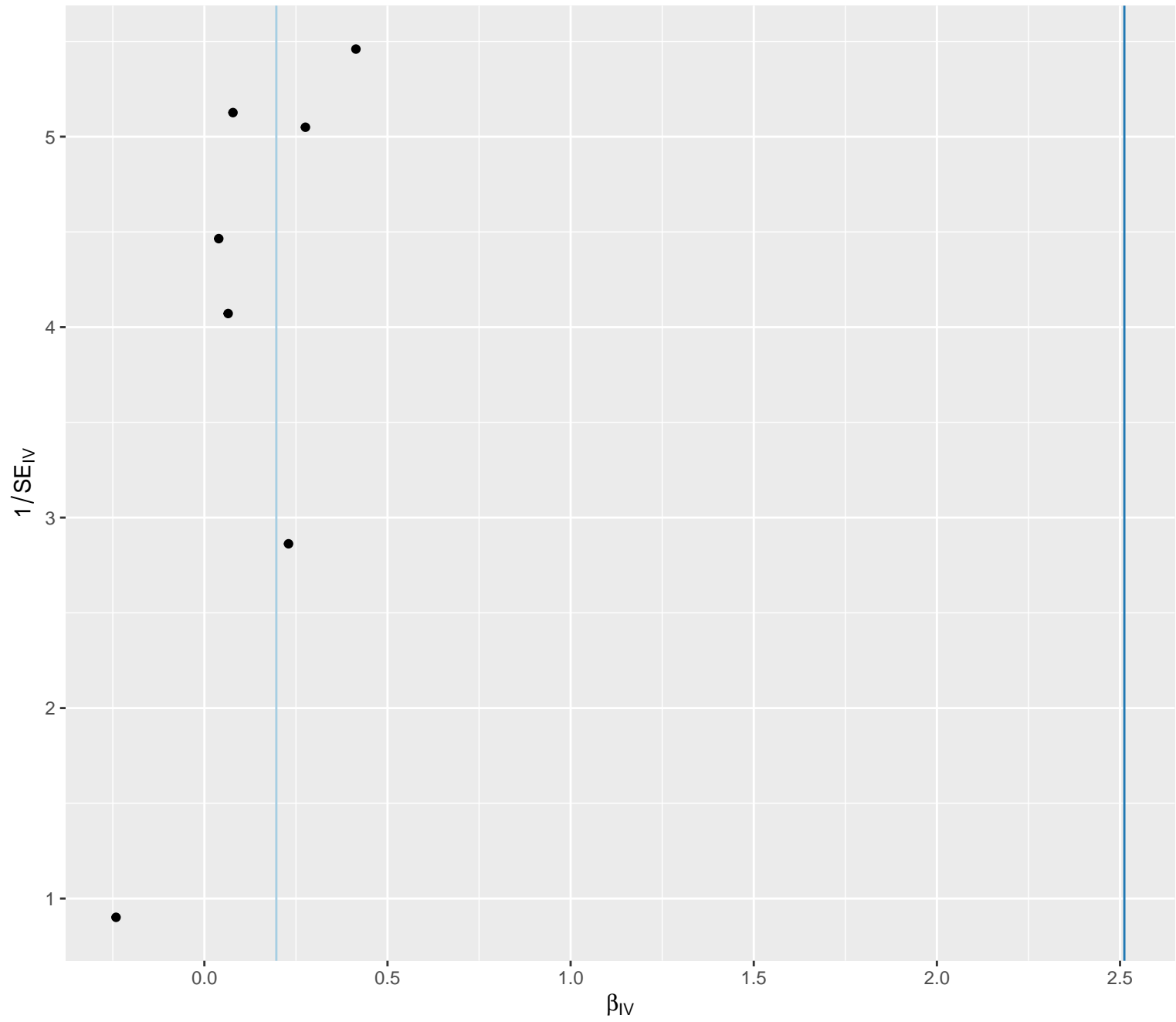

Supplement: Supplementary file 1 [file Data_Sheet_1.zip › Supplementary Materials/MR plots for tongue/Pneumonia/s__Alloprevotella_rava_mgs_541/funnel.pdf]

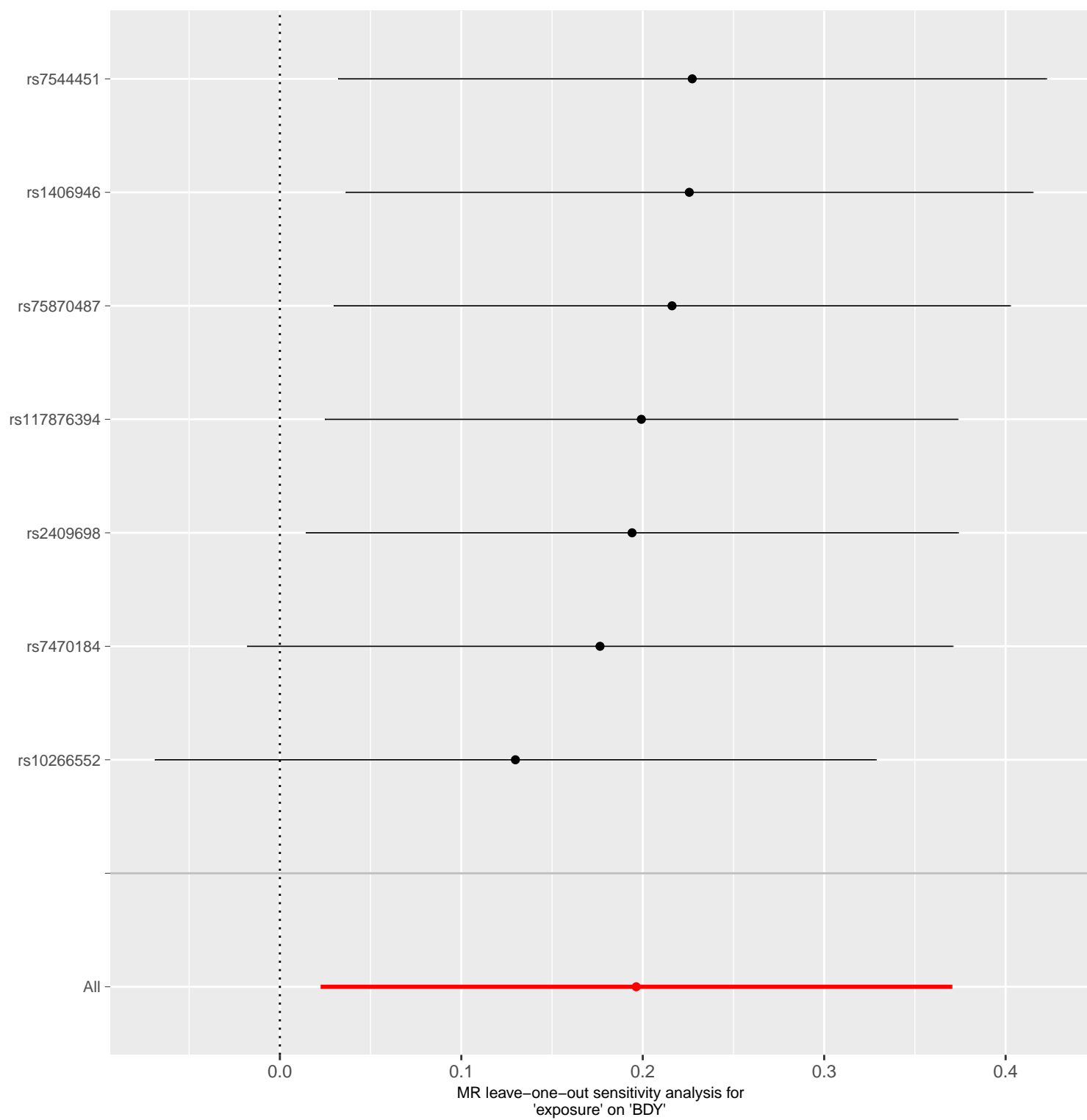

Supplement: Supplementary file 1 [file Data_Sheet_1.zip › Supplementary Materials/MR plots for tongue/Pneumonia/s__Alloprevotella_rava_mgs_541/leave_one_out.pdf]

# MR Test

- Inverse variance weighted
- Weighted median
- MR Egger

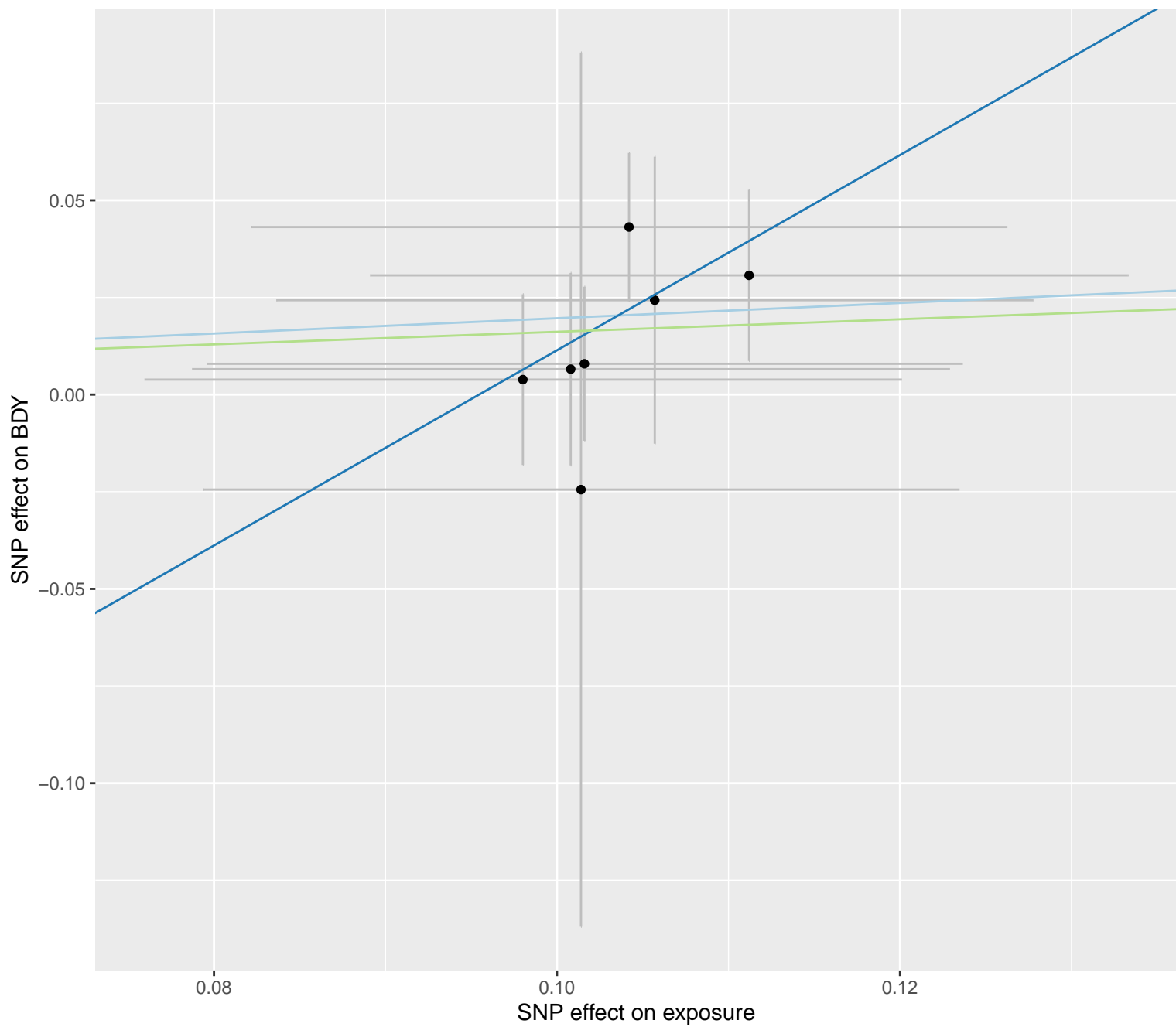

Supplement: Supplementary file 1 [file Data_Sheet_1.zip › Supplementary Materials/MR plots for tongue/Pneumonia/s__Alloprevotella_rava_mgs_541/scatter.pdf]

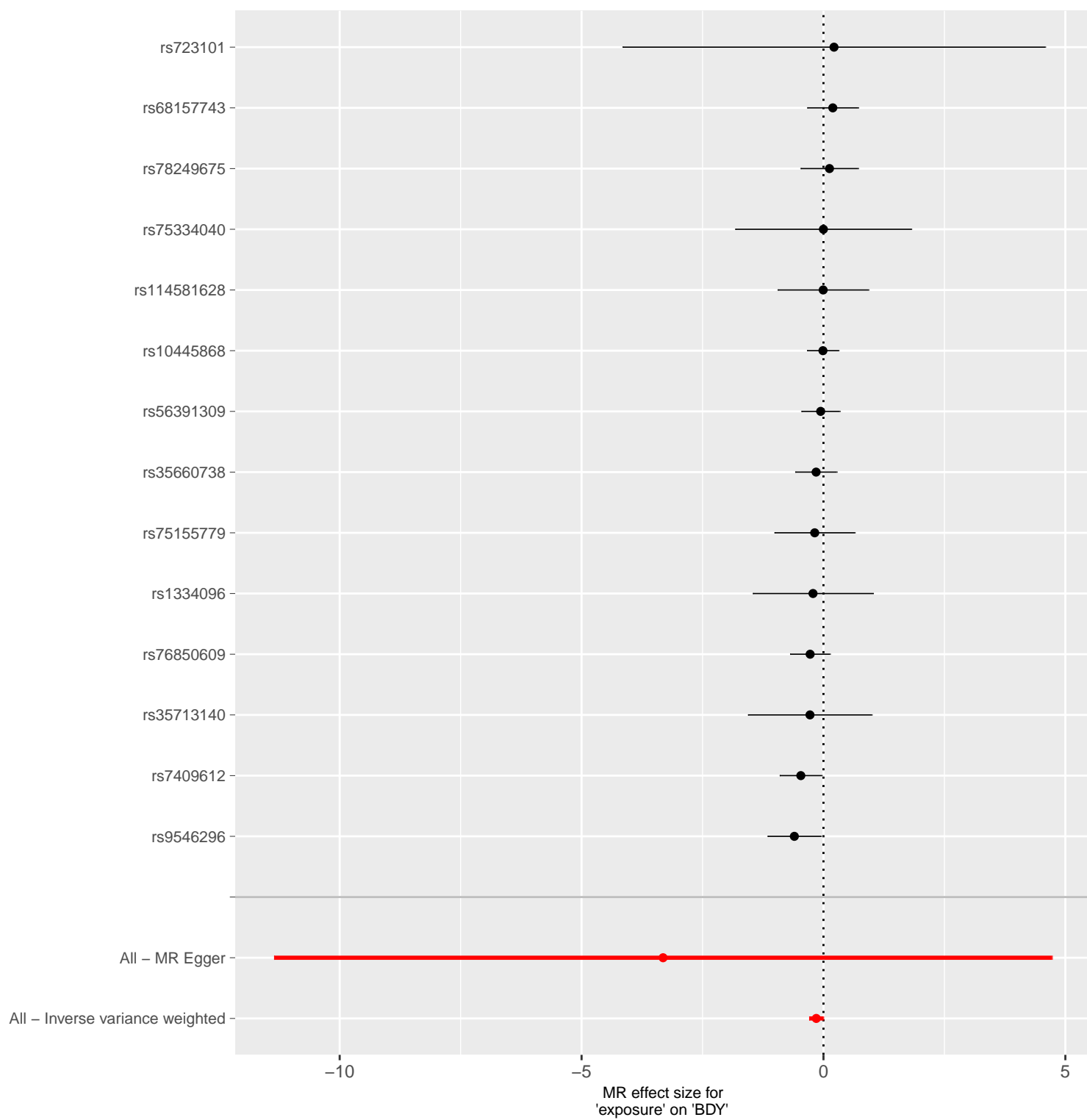

Supplement: Supplementary file 1 [file Data_Sheet_1.zip › Supplementary Materials/MR plots for tongue/Pneumonia/s__Campylobacter_A_rectus_mgs_2542/forest.pdf]

# MR Method

- Inverse variance weighted
- MR Egger

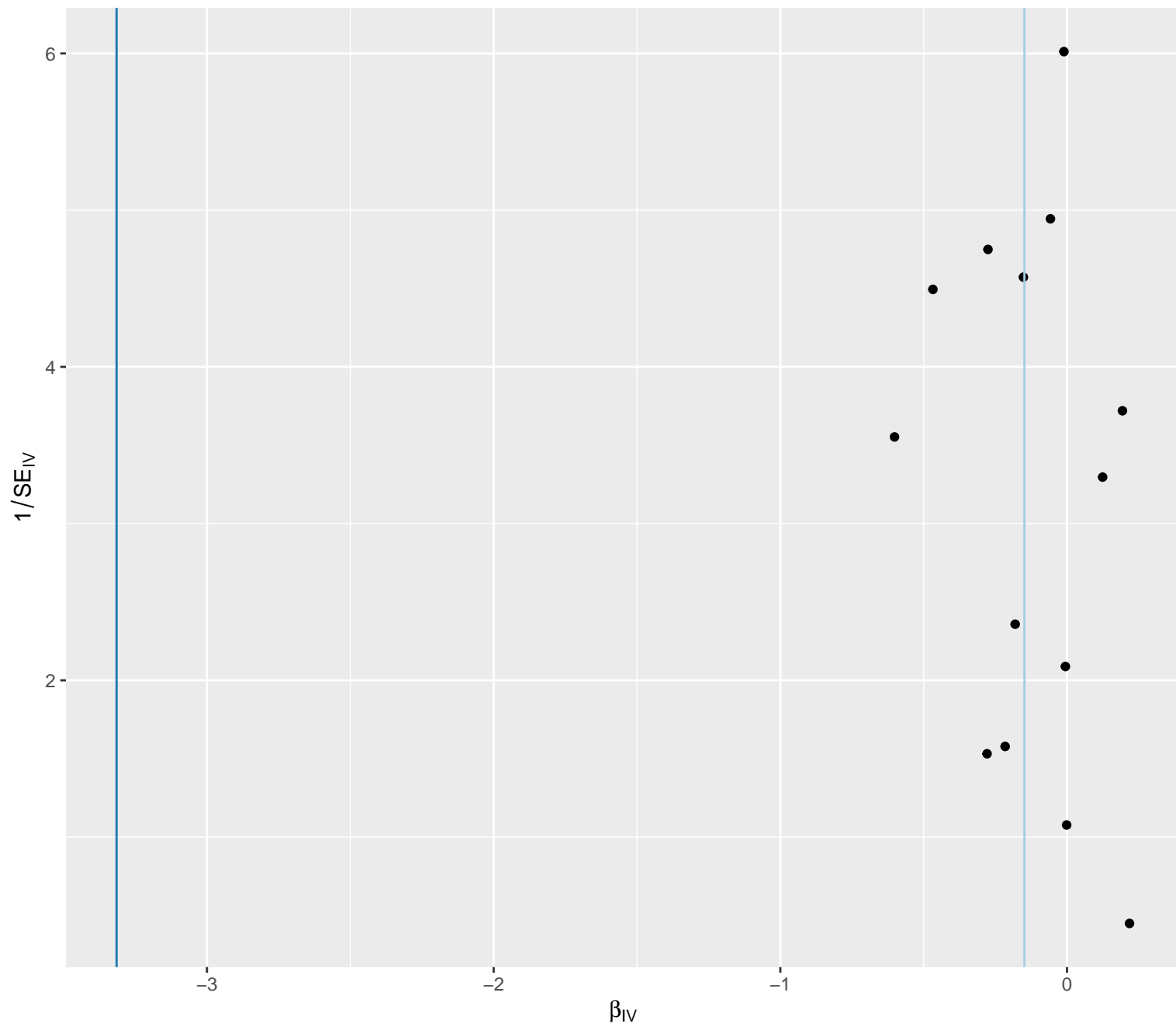

Supplement: Supplementary file 1 [file Data_Sheet_1.zip › Supplementary Materials/MR plots for tongue/Pneumonia/s__Campylobacter_A_rectus_mgs_2542/funnel.pdf]

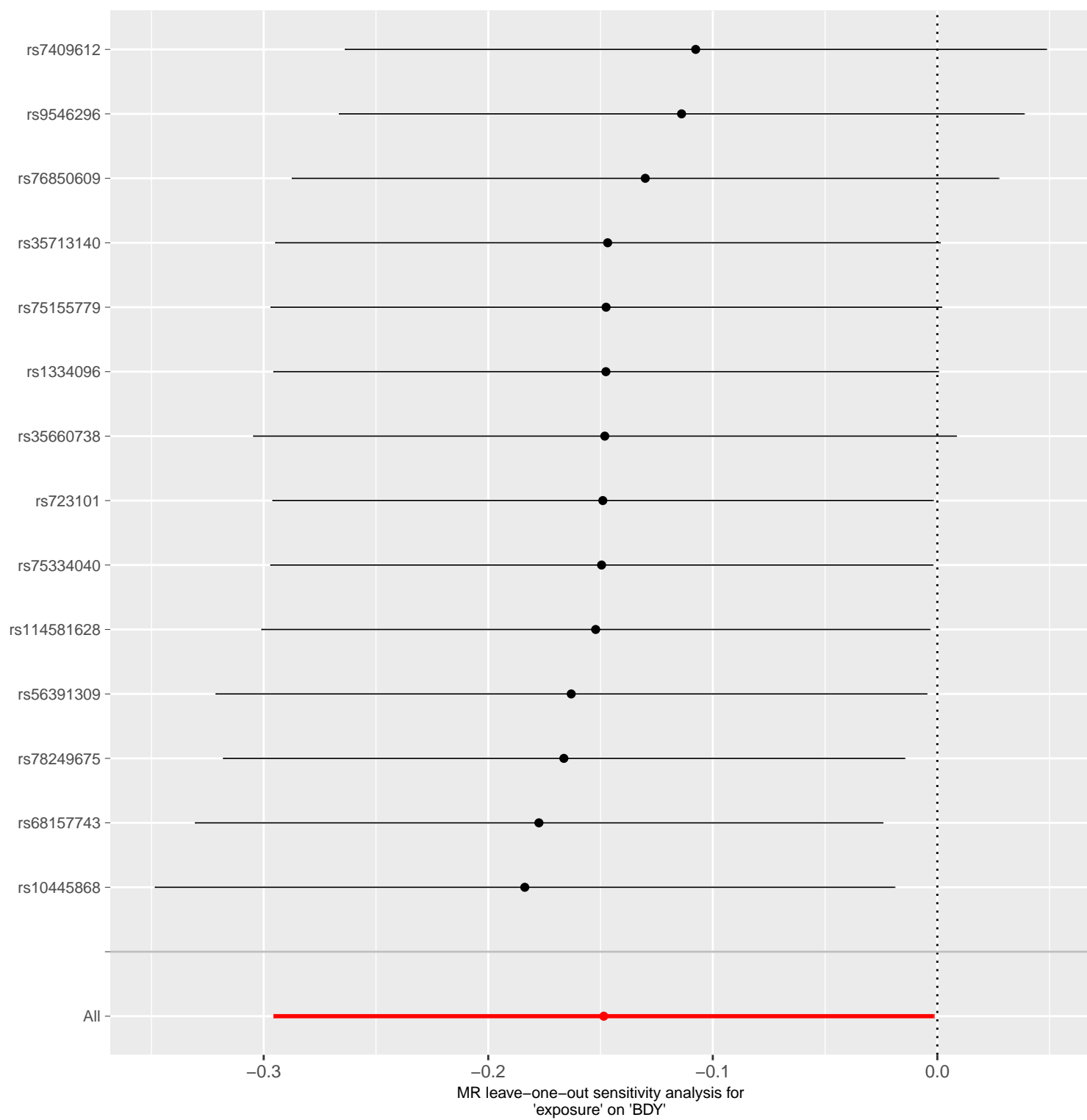

Supplement: Supplementary file 1 [file Data_Sheet_1.zip › Supplementary Materials/MR plots for tongue/Pneumonia/s__Campylobacter_A_rectus_mgs_2542/leave_one_out.pdf]

# MR Test

- Inverse variance weighted
- MR Egger
- Weighted median

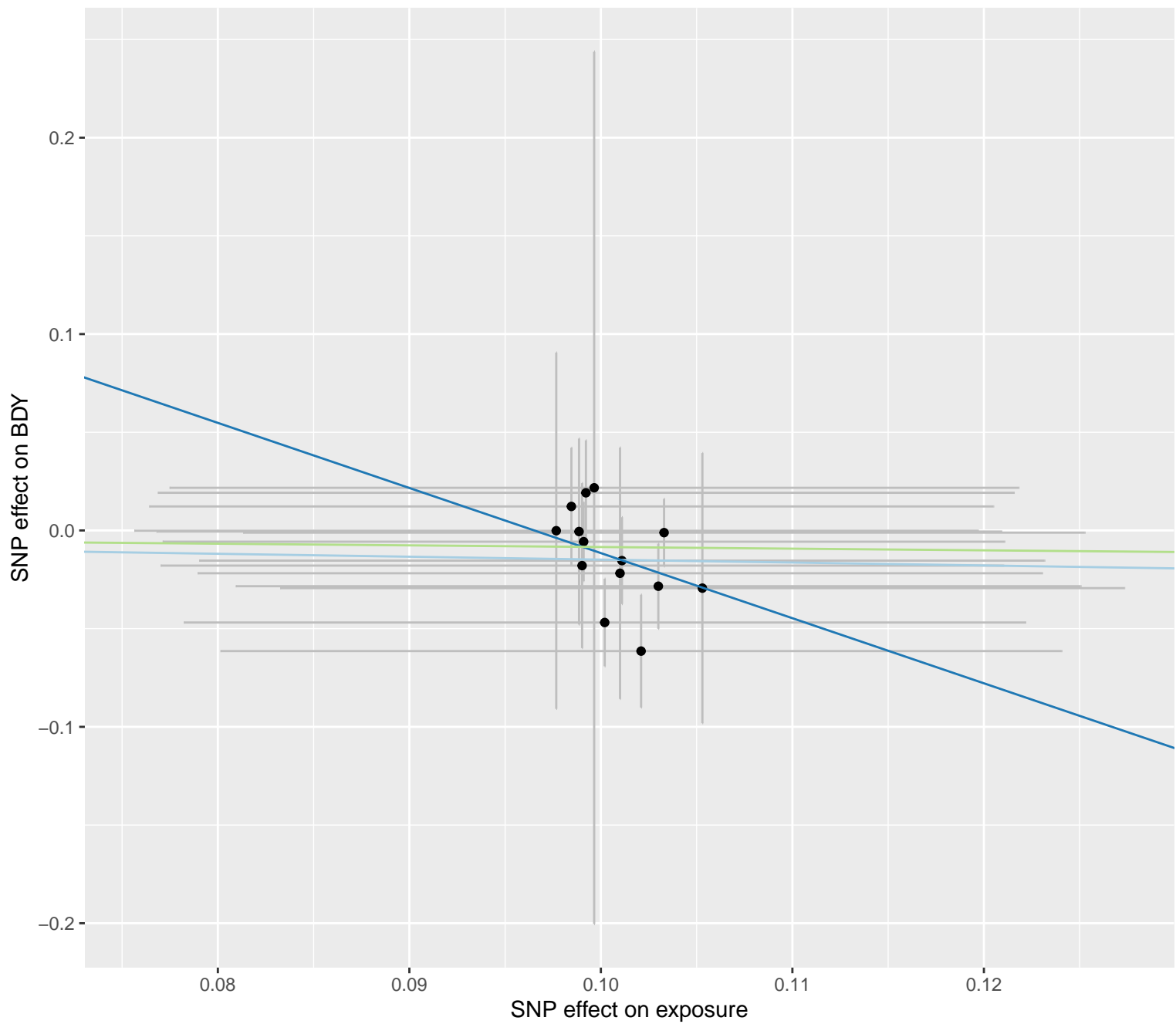

Supplement: Supplementary file 1 [file Data_Sheet_1.zip › Supplementary Materials/MR plots for tongue/Pneumonia/s__Campylobacter_A_rectus_mgs_2542/scatter.pdf]

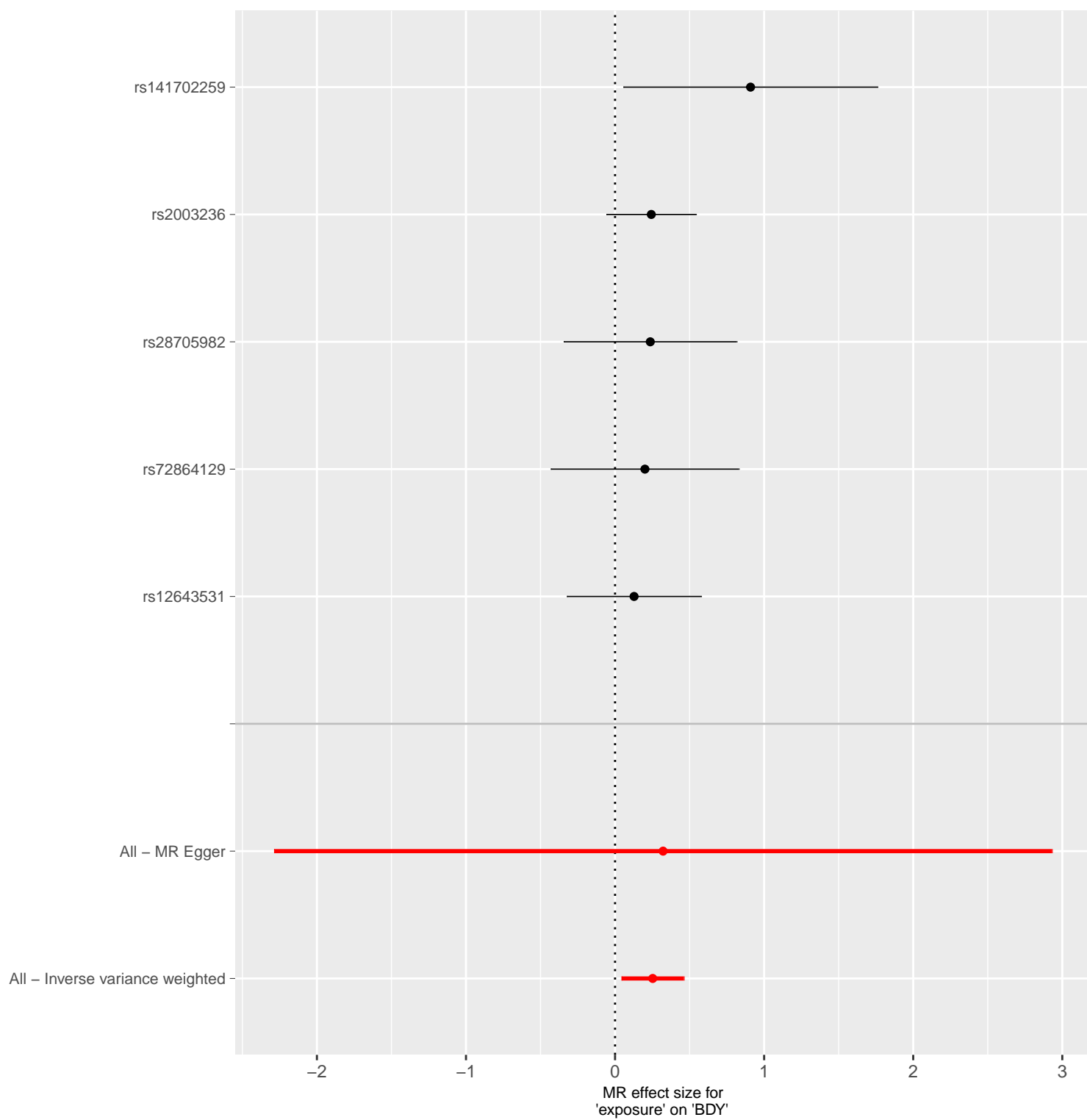

Supplement: Supplementary file 1 [file Data_Sheet_1.zip › Supplementary Materials/MR plots for tongue/Pneumonia/s__Centipeda_noxia_mgs_3198/forest.pdf]

# MR Method

- Inverse variance weighted
- MR Egger

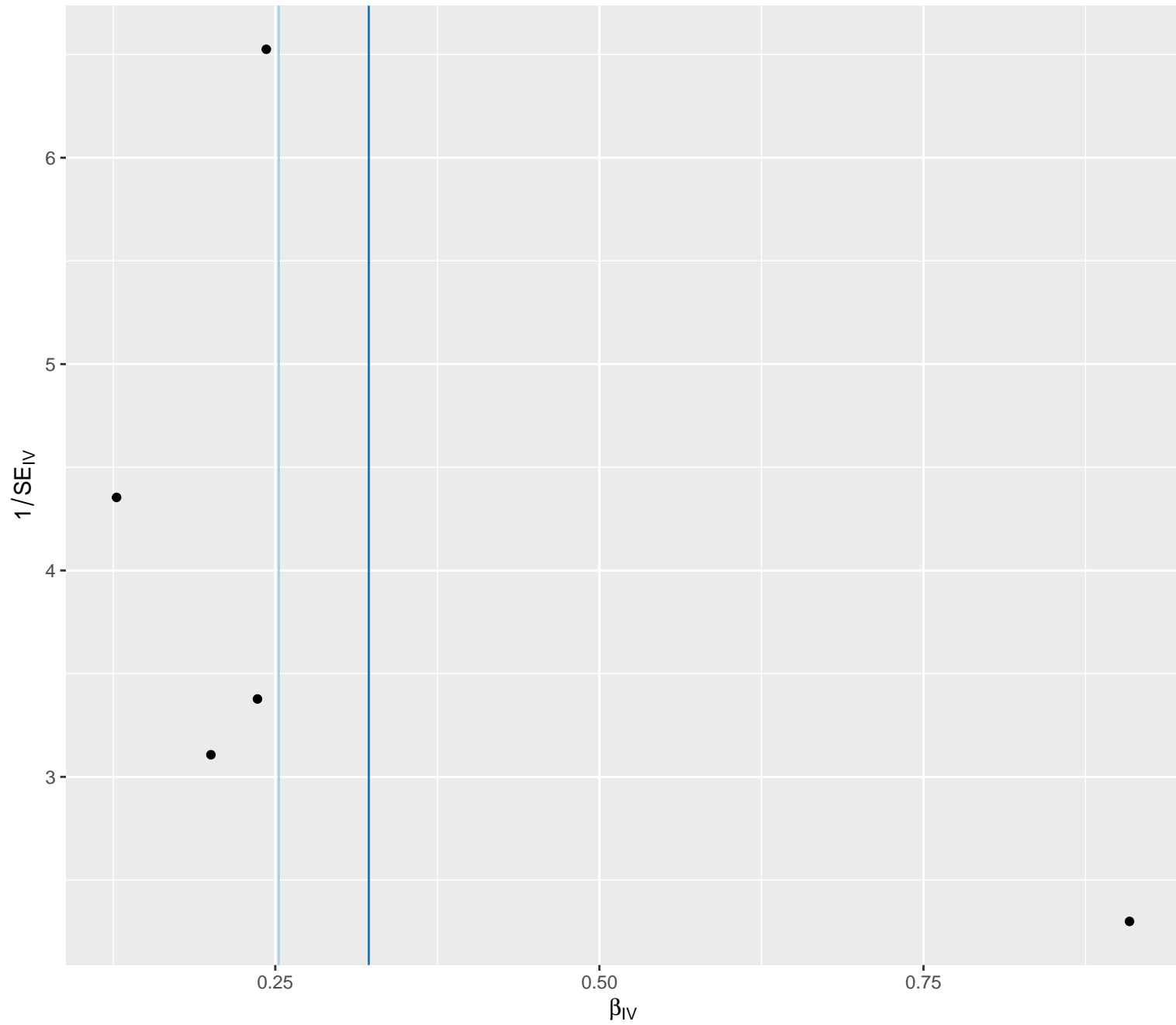

Supplement: Supplementary file 1 [file Data_Sheet_1.zip › Supplementary Materials/MR plots for tongue/Pneumonia/s__Centipeda_noxia_mgs_3198/funnel.pdf]

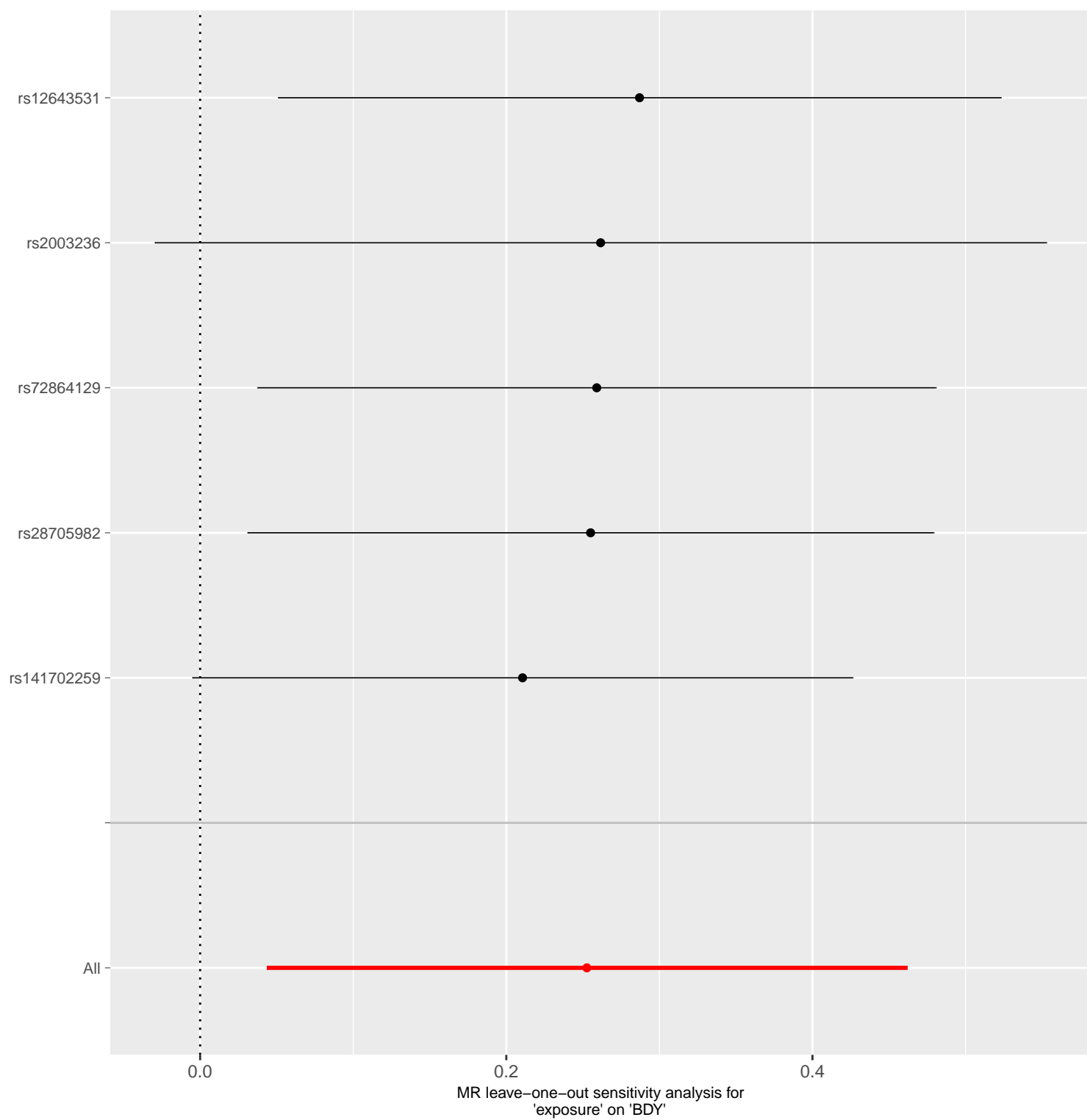

Supplement: Supplementary file 1 [file Data_Sheet_1.zip › Supplementary Materials/MR plots for tongue/Pneumonia/s__Centipeda_noxia_mgs_3198/leave_one_out.pdf]

# MR Test

- Inverse variance weighted
- MR Egger
- Weighted median

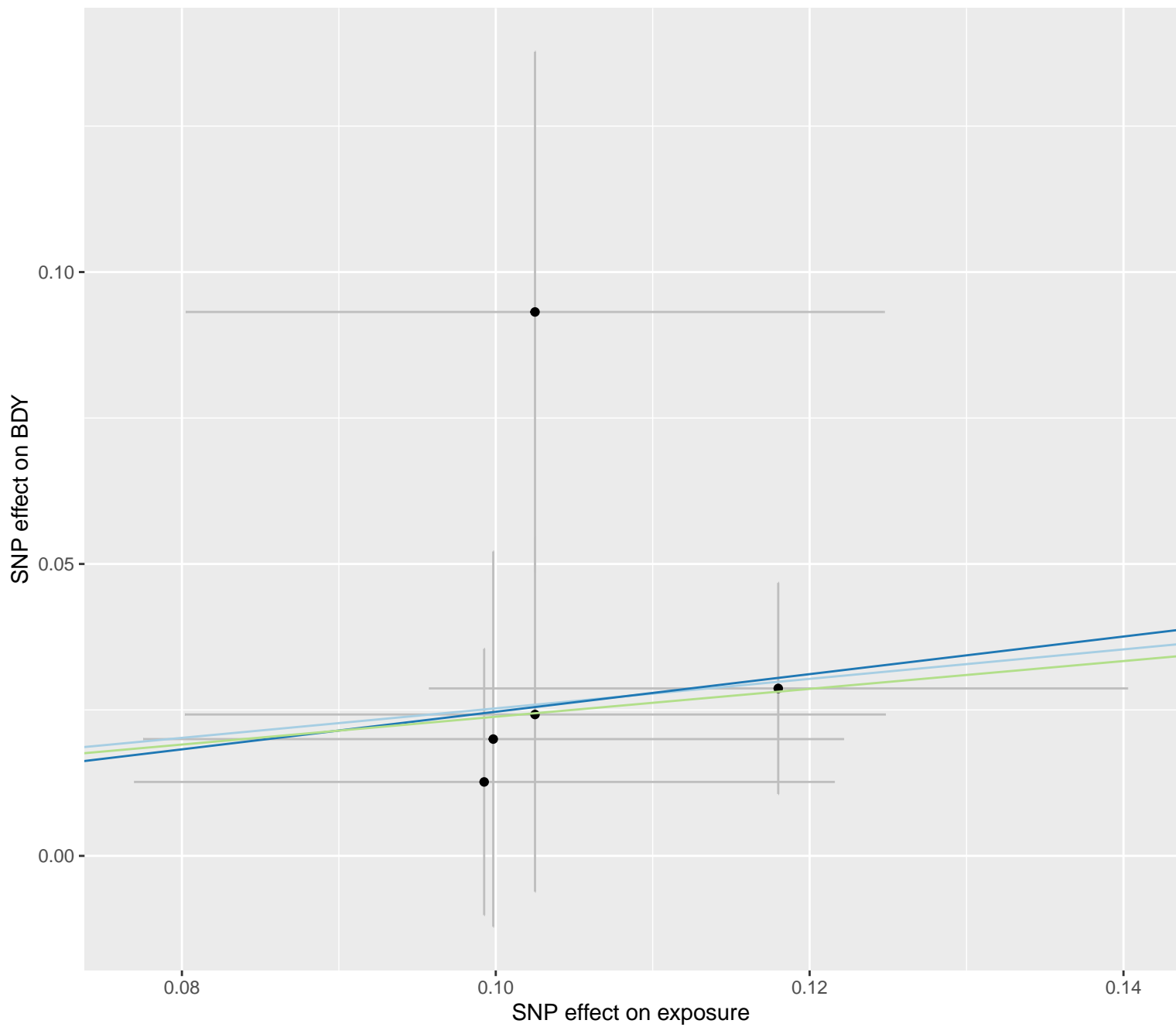

Supplement: Supplementary file 1 [file Data_Sheet_1.zip › Supplementary Materials/MR plots for tongue/Pneumonia/s__Centipeda_noxia_mgs_3198/scatter.pdf]

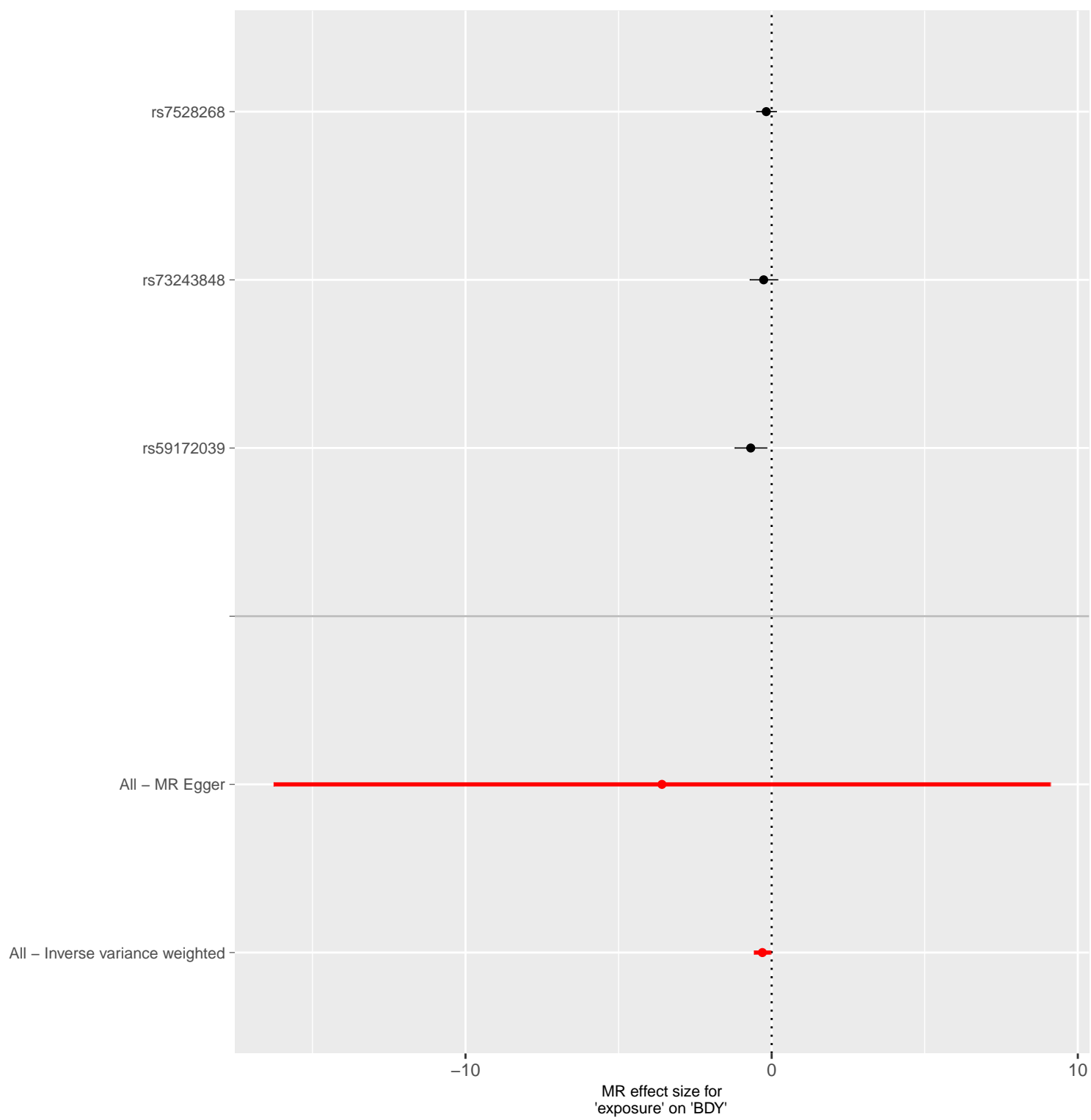

Supplement: Supplementary file 1 [file Data_Sheet_1.zip › Supplementary Materials/MR plots for tongue/Pneumonia/s__Eikenella_corrodens_mgs_3538/forest.pdf]

# MR Method

- Inverse variance weighted
- MR Egger

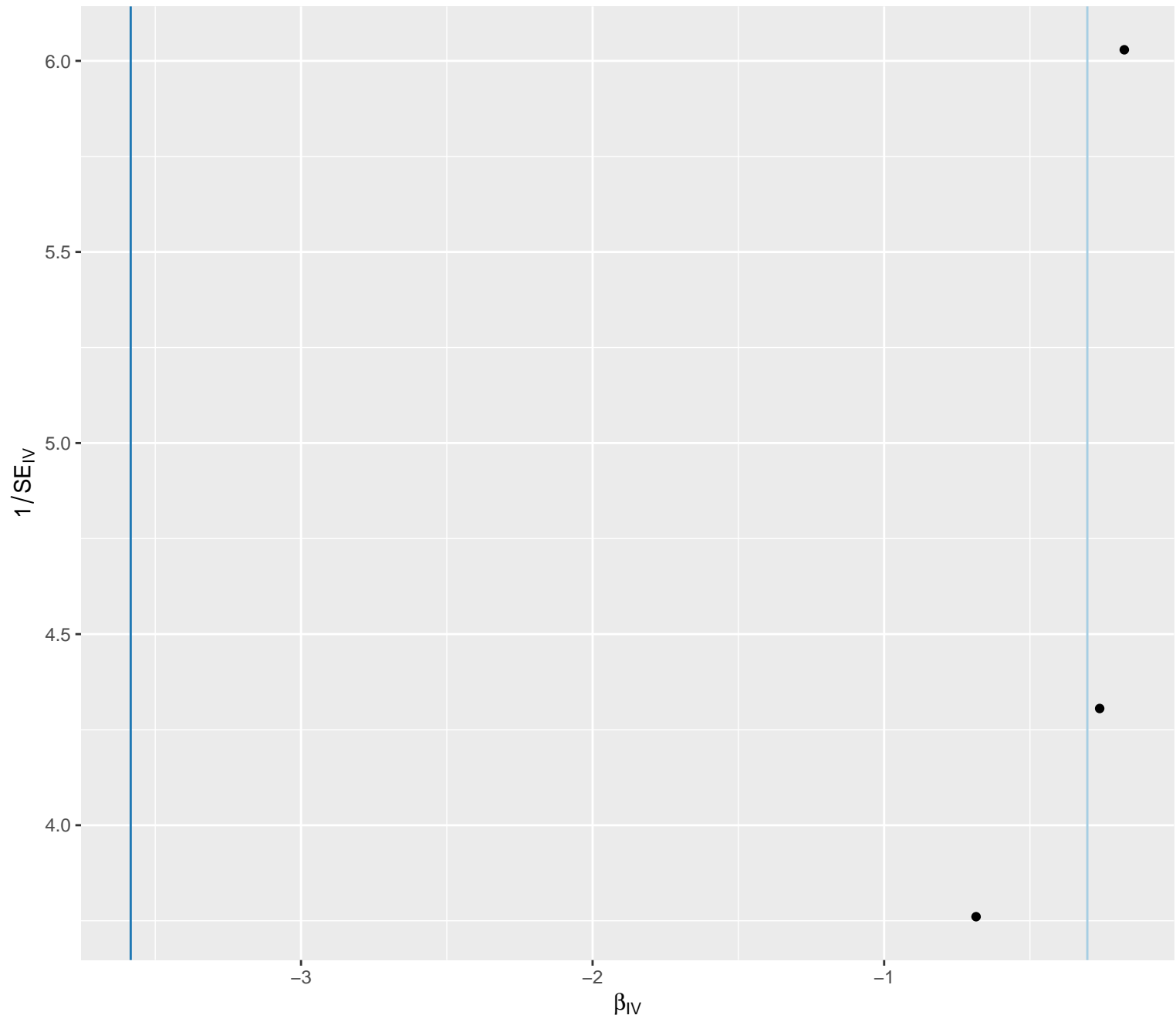

Supplement: Supplementary file 1 [file Data_Sheet_1.zip › Supplementary Materials/MR plots for tongue/Pneumonia/s__Eikenella_corrodens_mgs_3538/funnel.pdf]

rs59172039

rs73243848

rs7528268

All

-0.9

-0.6

-0.3

0.0

MR leave-one-out sensitivity analysis for  
'exposure' on 'BDY'

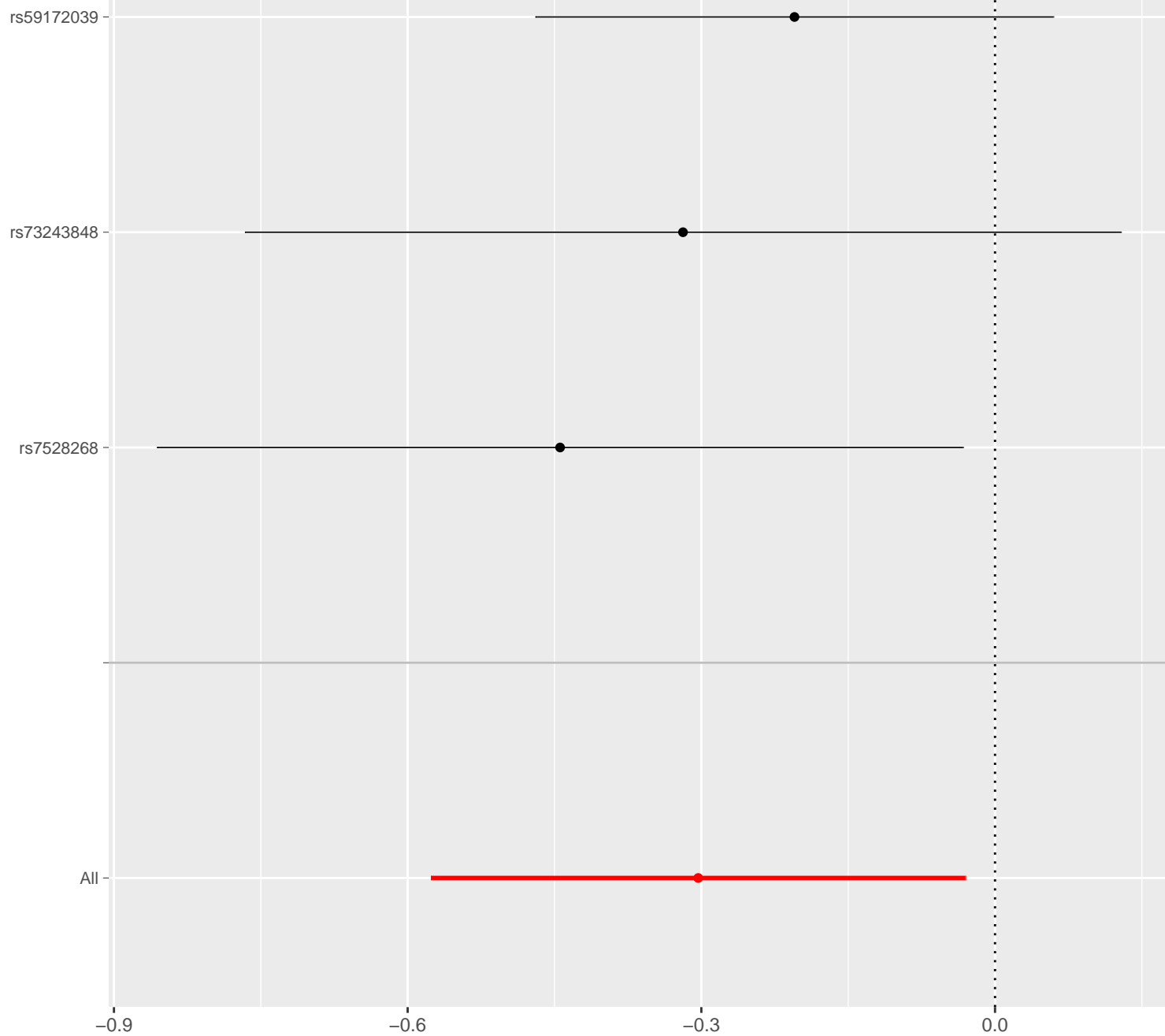

Supplement: Supplementary file 1 [file Data_Sheet_1.zip › Supplementary Materials/MR plots for tongue/Pneumonia/s__Eikenella_corrodens_mgs_3538/leave_one_out.pdf]

# MR Test

- Inverse variance weighted
- Weighted median
- MR Egger

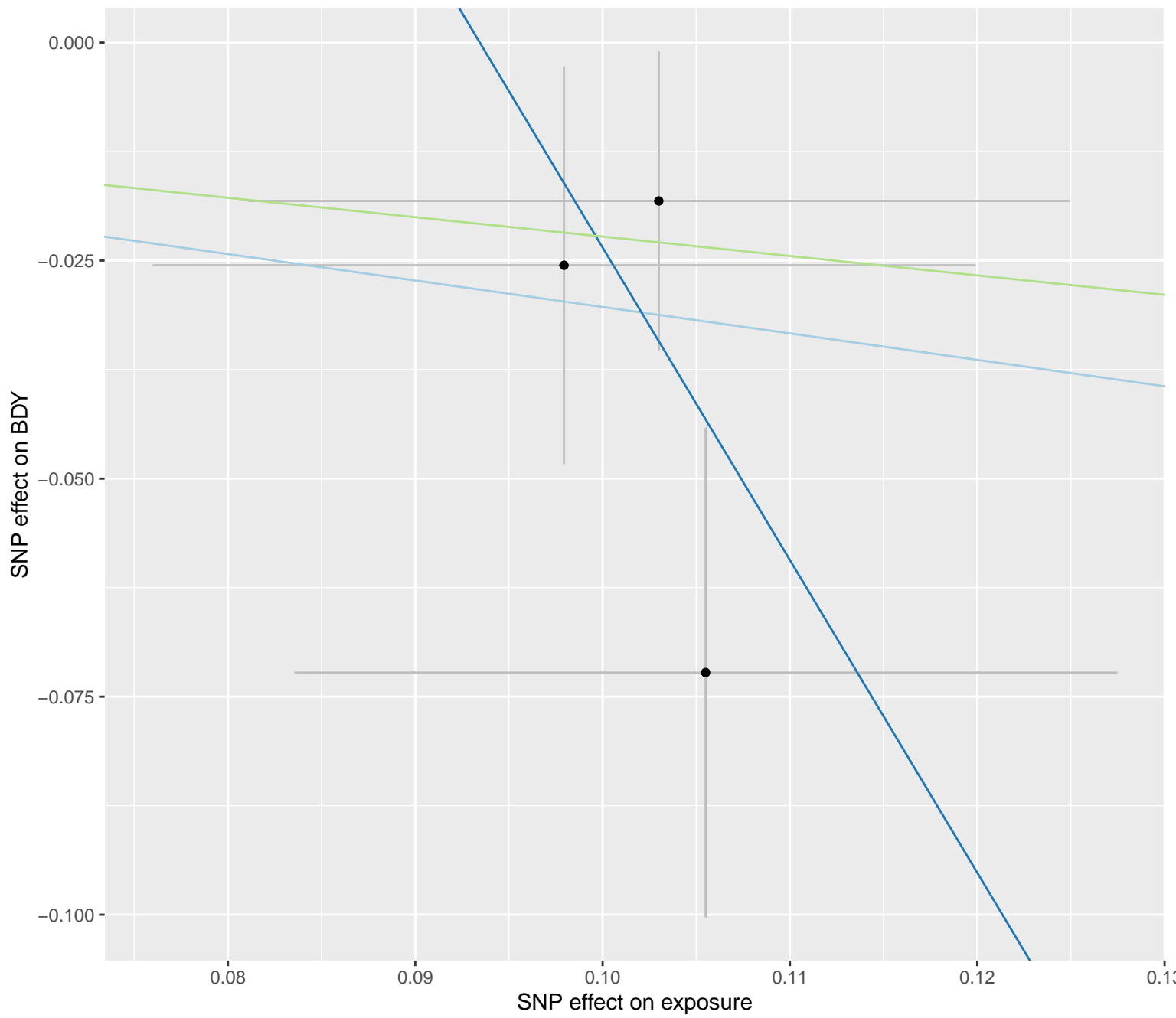

Supplement: Supplementary file 1 [file Data_Sheet_1.zip › Supplementary Materials/MR plots for tongue/Pneumonia/s__Eikenella_corrodens_mgs_3538/scatter.pdf]
